# PeGAS: a versatile bioinformatics pipeline for antimicrobial resistance, virulence and pangenome analysis

**DOI:** 10.1093/bioadv/vbaf165

**Published:** 2025-07-09

**Authors:** Liviu-Iulian Rotaru, Marius Surleac

**Affiliations:** Department of Anatomy, Animal Physiology and Biophysics, Faculty of Biology, University of Bucharest, Bucharest 050663, Romania; Research Institute of the University of Bucharest, University of Bucharest, Bucharest 050663, Romania; Department of Molecular Genetics, National Institute for Infectious Diseases, “Matei Bals”, Bucharest 021105, Romania

## Abstract

**Motivation:**

Antimicrobial resistance is increasingly recognized as one of the most significant global health threats, with profound implications for human, animal, and environmental health. Genome analysis represents a very useful tool that provides accurate and reproducible results allowing for the advancement of knowledge regarding antimicrobial resistance diagnosis, therapeutics, surveillance, transmission, and evolution. However, due to increasing complexity of bacterial genome analysis and computational power required for genomic approaches, there is a continuous need for comprehensive, user-friendly tools for data analysis. We developed Pangenome and Genomic Analysis Suite (PeGAS), to address some of these challenges by offering an all-in-one pipeline that performs a range of analyses.

**Results:**

PeGAS integrates key genomic analysis features of bacteria whole genome sequencing, including the prediction of antimicrobial resistance profiles, sorted by various categories of antibiotics, VF detection, and plasmid replicon assignment. The pipeline also performs pangenome analysis, multilocus sequence typing, genome assembly quality control (by reporting statistics such as GC content, contig length, the number of contigs, as well as variation from certain GC thresholds) providing a comprehensive genomic overview. PeGAS also offers the ability to restart seamlessly from any sporadic interruptions that might occur during long or resource-intensive runs.

**Availability and implementation:**

PeGAS is available at: https://github.com/liviurotiul/PeGAS

## 1 Introduction

Antimicrobial resistance has emerged as one of the most pressing global health challenges of the twenty-first century (GBD 2021 Antimicrobial Resistance Collaborators 2024). With the rise of resistant bacterial strains, many of which exhibit high levels of resistance and even pan-resistance and virulence, the effectiveness of traditional antibiotics is rapidly waning. In some cases, these treatments are rendered completely ineffective, posing significant challenges to managing even routine infections. The spread of antimicrobial resistance (AMR) coupled with continuous changes in bacterial behavior (e.g. quorum sensing, biofilm formation) had added complexity to these systems and could ultimately lead to pan-resistance (Krishna et al. 2025). The emergence and spread of AMR are not confined only on human contact; this occurs across diverse ecosystems—it disseminates through environmental vectors, reaches wild & domestic animals, flows through natural and polluted water & soil systems, creating a continuous cycle of transmission (Jian et al. 2021). To fully understand the extent of AMR spread, it is essential to approach the issue from a One Health perspective, integrating human, animal and environmental health considerations (Velazquez-Meza et al. 2022, Liu and Pandey 2024). Predicting AMR profiles, identifying potential virulence factors (VF) and the circulating plasmids carrying antibiotic resistance genes (ARG), are essential to better understand the AMR evolution and inform targeted treatments (Jian et al. 2021, Wang et al. 2023).

The rise of high-throughput sequencing technologies has led to an explosion of genomic data, necessitating robust, scalable tools to manage, analyze, and interpret this data efficiently. In this context, bioinformatics pipelines have become indispensable, and effective surveillance requires fast and reliable tools readily accessible to researchers and clinicians, even to those without a background in computer science (Gil-Gil et al. 2021).

PeGAS reports crucial assembly statistics (e.g. contigs length, N50 average coverage, N50 average length), and it also provides insights into the quality of the sequencing, evaluating GC content, and identifying isolates that may have undergone issues during or prior to sequencing.

PeGAS features a user-friendly installation guide, the ability to resume interrupted runs, and an eye-catching easy to follow HTML output report, making it a practical solution for researchers handling large datasets.

## 2 Methods

### 2.1 Pipeline overview

**Figure 1.**
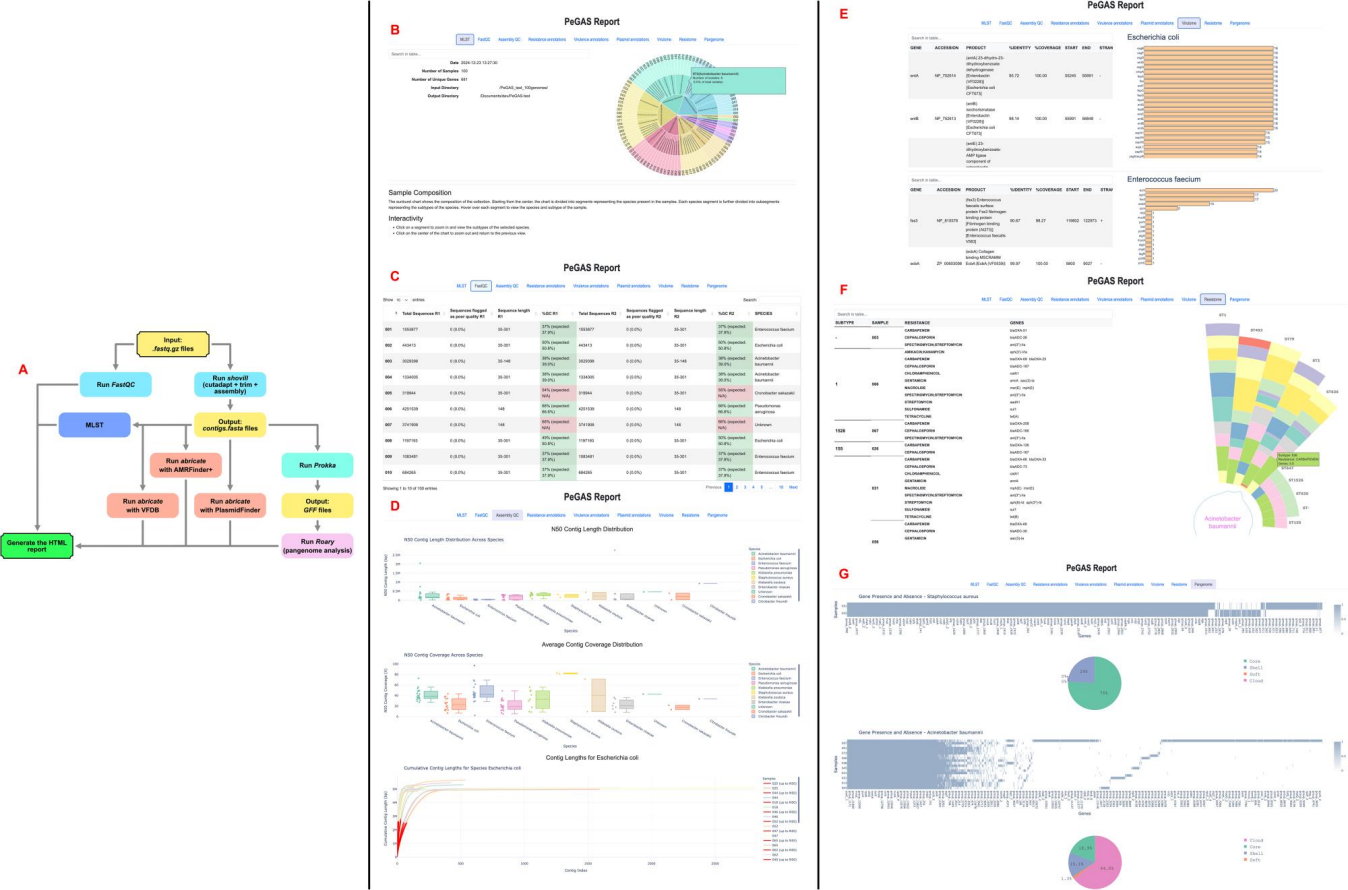
PeGAS pipeline overview: (A) Workflow diagram; (B) MLST, (C) FastQC, (D) Assembly QC, (E) Virulence, (F) Resistance, and (G) Pangenome reports, respectively.

PeGAS integrates multiple analysis steps into a single streamlined workflow, as illustrated in Fig. 1A. This workflow includes raw data QC, de novo genome assembly, multilocus sequence typing (MLST), and downstream analyses for AMR, VF and plasmidic replicon predictions, annotations and pangenome analysis. The pipeline was developed using open-source tools and it is accessible through a simple Conda-based installation, ensuring compatibility on Unix systems (though it can be used on other systems by virtualization). Additionally, PeGAS has the capability to resume from where it left off in the event of a run break, making it robust solution for handling long or resource-intensive processes.

### 2.2 Prerequisites and use. Pipeline implementation and availability

PeGAS is implemented in Python and leverages Snakemake (Mölder et al. 2021) to manage workflow dependencies, ensuring reproducibility and scalability. One important note is that Snakemake makes use of Mamba, a Conda package manager implementation that can only be installed alongside a Miniconda implementation and not Anaconda (https://github.com/anaconda) (Anaconda Software Distribution 2016). To install the prerequisites, the users should follow the guidelines: https://github.com/conda-forge/miniforge, https://docs.anaconda.com/miniconda/miniconda-install/ and then proceed with Mamba and PeGAS installation in the command line, following the guidelines from the page on Github: https://github.com/liviurotiul/PeGAS. PeGAS can then be ran with the command “pegas -d path/to/input/folder -o path/to/output/folder” where the input folder contains only fastq.gz files, and the results will be saved to the output folder specified by the user.

The PeGAS pipeline is distributed through Conda, on the Bioconda channel, allowing for a seamless installation process on Unix systems. Guidance on how to install and use PeGAS can be found on the Github page; short guidelines for interpreting the results can be found at the bottom of specific tabs in the HTML report.

PeGAS allows customization of resource usage through the –cores argument (specifying how many jobs can be run in parallel). Additionally, the number of cores or threads used by tools like shovill (Seemann 2020b), Prokka (Seemann 2014), and Roary (Page et al. 2015), can further be specified. While Snakemake is designed to scale down when overloaded, it is recommended to configure resources conservatively to prevent system instability.

PeGAS identifies and subsequently tracks each sample/isolate by removing the ‘_R1’/‘_R2’ suffix from the fastq.gz files. The resulting HTML report can then be accessed by opening ‘output_folder/report/report.html’ and a CSV file containing all the resulted annotations can be found in ‘output_folder/dataframe.’

### 2.3 The test dataset

The input dataset is made of raw reads that were randomly selected from another set of approximately one thousand genomes previously sequenced in our laboratory. The dataset selection is purely used for underlining the features of PeGAS, and not for a detailed contextual research analysis. To showcase the full functionality of PeGAS, we included raw data from ten different species. The isolates were assigned simple numerical identifiers ranging from ‘001’ to ‘100’ for clarity and consistency.

### 2.4 Quality control and genome assembly

Before performing any downstream analysis, raw reads undergo a QC check using FastQC (Andrews 2010). The reads are then processed through Shovill, a powerful prokaryotic genome assembly pipeline to clean reads, cut adapters and assemble them into contigs. After assembly, key metrics such as GC content, contig length, and the number of contigs are calculated and reported. These metrics are critical to assess the quality of the genome assembly and identifying potential issues that could affect downstream analyses (Fig. 1C and D).

### 2.5 Antimicrobial resistance, virulence factors and plasmid prediction

PeGAS employs the NCBI AMRFinderPlus (Feldgarden et al. 2021) database, integrated with Abricate (Seemann 2020a), for AMR prediction. The pipeline aligns the assembled genome to the corresponding genes database, classifying AMR profiles based on antibiotic categories, providing detailed insights into the resistance mechanisms. In parallel, VF prediction is performed using the VFDB (Chen et al. 2016) and PlasmidFinder (Carattoli et al. 2014) is used to assign the replicons associated with certain plasmids from various species (Fig. 1E and F).

### 2.6 Pangenome analysis

To capture the diversity within a bacterial species, PeGAS includes pangenome analysis, performed with Roary after the assembled genomes have been previously annotated with Prokka. This analysis identifies the core and accessory genes present across multiple isolates, offering insights into the evolutionary dynamics of bacterial species (Fig. 1G). Pangenome analysis enhances the understanding of genetic diversity and highlights potential adaptive traits.

### 2.7 MLST prediction

PeGAS includes MLST prediction (Fig. 1B) using the traditional PubMLST typing schemes (Jolley et al. 2018, Seemann 2018). Using a predefined set of housekeeping genes, the pipeline assigns a sequence type (ST) to the assembled genome, facilitating epidemiological studies and comparisons between isolates. MLST results are crucial for identifying bacterial lineages and understanding the evolutionary relationships among isolates. This information is particularly valuable, as certain STs are often more pathogenic than others, providing insights into the potential risk associated with specific bacterial strains.

### 2.8 Data visualization and reporting

At the end of the run, PeGAS generates a detailed HTML summary report that consolidates all the results, filtered according to the already discussed traits. The results include plots of species distribution among the selected isolates; data tables for ARGs, VFs, and plasmidic replicons; plots for ST and antibiotic categories distribution; assembly details plots; and pangenome composition summaries. These results are presented in both graphical and tabular formats, allowing for easy interpretation and actionable insights.

## 3 Results

We present a comprehensive report generated by PeGAS on 100 bacterial isolates belonging to the ESKAPE group of pathogens (Miller and Arias 2024). The report generated by PeGAS contains nine tabs presented in the following order: MLST, FastQC, Assembly QC, Resistance annotations, Virulence annotations, Plasmid annotations, Virulome, Resistome and Pangenome (Fig. 1B-G).

The ‘MLST’ tab also serves as welcome page (Fig. 1B). On the left side, it provides general information about the sequencing run, while on the right side, an interactive chart visually represents the isolates’ collection. The chart is organized hierarchically with the predicted Species at the core level of the pie chart, followed by Subtypes and finally Isolates’ names/IDs. Hovering over a subtype or species segment displays the number of isolates and their percentage contribution to the total collection. For isolates missing the complete set of genes required to assign MLST or those containing entirely new mutations, the system designates Unknown subtype and Species classification (e.g. isolate 007 in Fig. 1B).

The ‘FastQC’ tab (Fig. 1C) provides a summary table offering a concise overview of key sequencing quality metrics, enabling rapid assessment of raw data integrity prior to downstream analysis. A key aspect here is the implementation of %GC content, shown as actual versus expected values. The expected values are based on values reported throughout the literature. To aid interpretation, the cells containing %GC in the summary table are color-coded for quick visual assessment: green indicates that the actual %GC is <6% of the expected value, suggesting consistency with the species’ genomic characteristics, while red highlights represent deviations >6%, potentially suggesting contamination, sequencing bias or natural genomic variation. A complete description of how to interpret the table is provided at the bottom of the tab, allowing users to reference the information as needed.

The ‘Assembly QC’ tab (Fig. 1D) focuses on contig assembly quality, providing plots that assess assembly performance and identify potential issues. Similarly, a complete explanation of the plots is provided at the bottom of the ‘Assembly QC’ tab.

The ‘Resistance,’ ‘Virulence,’ and ‘Plasmid annotations’ tabs provide detailed tabled information on gene annotations (see supplementary ‘PeGAS_Report.html’ file). The table’s display settings are customizable, allowing users to adjust the number of rows displayed, perform string-based searches, and sort data for easier exploration and interpretation.

The ‘Virulome’ tab (Fig. 1E) serves as a summary of virulence gene diversity across species, and it summarizes the ‘Virulence annotations’ tab. By examining both the bar plot and the table, users can gain insights into the diversity and distribution of virulence genes.

Similarly to the ‘Virulome,’ the ‘Resistome’ tab (Fig. 1F) provides an overview of the resistance genes’ distribution across subtypes within a species. It integrates an interactive polar chart and a corresponding annotation table. The polar chart shows the average number of resistance genes per sample for each ST. The bars are ordered by descending average gene count, with each bar’s height reflecting the average number of genes. Colors are used to indicate distinct drug resistance categories, making it easy to interpret resistance patterns across subtypes. A central circular line plot shows the number of samples per subtype, enabling users to assess representativeness within the dataset. Hovering over a gene in the table, displays gene percentage identity when compared to its reference sequence. By correlating the insights from the polar chart with the details in the table, users can identify patterns in resistance gene distribution, detect dominant resistance types, and pinpoint unusual or interesting profiles. This representation is applied to all species found in each set of isolates submitted for analysis.

The ‘Pangenome’ tab (Fig. 1G) presents pangenome distributions for each species, generally employing pie charts and heatmaps to visualize core and accessory gene sets. These visualizations enable users to examine patterns of gene presence and absence, compare the genomic diversity across species, and explore the relationships between core and accessory genes.

## 4 Discussion and conclusion

There are multiple complementary methods that address similar prediction features. Some focus on different aspects of AMR while others are designed to solve specific problems, such as annotating genomes. Certain tools are cross-platform compatible, while others are accessible only through online interfaces (Le et al. 2024, Seeman et al. 2015, Quijada et al. 2019, Petit and Read 2020, Argimón et al. 2021). Compared to other tools, we hope to provide a more accessible way to do fast, scalable, and visually impactful WGS AMR analysis.

PeGAS is an easily usable yet flexible bioinformatics pipeline for bacterial genome analysis encompassing AMR, VF prediction, plasmid replicon assignment, MLST, and pangenome exploration. Its installation process is straightforward, with all dependencies automatically managed through a Conda-based approach. By integrating all essential steps from raw reads to comprehensive visual reports, PeGAS simplifies the analysis process and ensures accessibility to a wide range of users.

The pipeline covers all key steps for bacterial genome analysis while keeping the number of required parameters to a minimum, ensuring ease of use. This user-friendly approach makes PeGAS a valuable resource for bacterial genomics, with applications ranging from routine laboratory data analysis to surveillance and research on emerging antimicrobial resistance threats.

## 5 Future work

There is a continuous need of tools for antimicrobial analysis that would cover as many details as possible that could help in understanding the resistance mechanisms. To support further the needs of researchers on this topic we are already working on underlining the context in which the elements of pathogenicity are found in respect to one another in the genome. Similarly, we want to implement the possibility to highlight the non-synonymous mutations in each predicted gene that could impact its function.

## Supplementary Material

vbaf165_Supplementary_Data

## Data Availability

The data underlying this article will be shared on reasonable request to the corresponding author. https://github.com/liviurotiul/PeGAS.
