# Supplementary material for: PeGAS: a versatile bioinformatics pipeline for antimicrobial resistance, virulence and pangenome analysis
Source: Bioinform Adv. 2025 Jul 9;5(1):vbaf165. doi: 10.1093/bioadv/vbaf165 (PMC12308278; doi:10.1093/bioadv/vbaf165)
Supplement: vbaf165_Supplementary_Data [file vbaf165_supplementary_data.zip › PeGAS_Report.html]

# PeGAS Report


- MLST
- FastQC
- Assembly QC
- Resistance annotations
- Virulence annotations
- Plasmid annotations
- Virulome
- Resistome
- Pangenome

|  |  |
| --- | --- |
| Date | 2024-12-23 13:27:30 |
| Number of Samples | 100 |
| Number of Unique Genes | 681 |
| Input Directory | /PeGAS\_test\_100genomes/ |
| Output Directory | /Documents/dev/PeGAS-test |

---


---

### Sample Composition

The sunburst chart shows the composition of the collection. Starting from the center, the chart is divided into segments representing the species present in the samples. Each species segment is further divided into subsegments representing the subtypes of the species.
Hover over each segment to view the species and subtype of the sample.

### Interactivity

- Click on a segment to zoom in and view the subtypes of the selected species.
- Click on the center of the chart to zoom out and return to the previous view.

|  | Total Sequences R1 | Sequences flagged as poor quality R1 | Sequence length R1 | %GC R1 | Total Sequences R2 | Sequences flagged as poor quality R2 | Sequence length R2 | %GC R2 | SPECIES |
| --- | --- | --- | --- | --- | --- | --- | --- | --- | --- |
| 001 | 1553877 | 0 (0.0%) | 35-301 | 37% (expected: 37.9%) | 1553877 | 0 (0.0%) | 35-301 | 37% (expected: 37.9%) | Enterococcus faecium |
| 002 | 443413 | 0 (0.0%) | 35-301 | 50% (expected: 50.8%) | 443413 | 0 (0.0%) | 35-301 | 50% (expected: 50.8%) | Escherichia coli |
| 003 | 3029398 | 0 (0.0%) | 35-148 | 38% (expected: 39.0%) | 3029398 | 0 (0.0%) | 35-148 | 38% (expected: 39.0%) | Acinetobacter baumannii |
| 004 | 1334005 | 0 (0.0%) | 35-301 | 38% (expected: 39.0%) | 1334005 | 0 (0.0%) | 35-301 | 38% (expected: 39.0%) | Acinetobacter baumannii |
| 005 | 319944 | 0 (0.0%) | 35-301 | 54% (expected: N/A) | 319944 | 0 (0.0%) | 35-301 | 55% (expected: N/A) | Cronobacter sakazakii |
| 006 | 4251539 | 0 (0.0%) | 148 | 66% (expected: 66.6%) | 4251539 | 0 (0.0%) | 148 | 66% (expected: 66.6%) | Pseudomonas aeruginosa |
| 007 | 3741908 | 0 (0.0%) | 148 | 66% (expected: N/A) | 3741908 | 0 (0.0%) | 148 | 66% (expected: N/A) | Unknown |
| 008 | 1197193 | 0 (0.0%) | 35-301 | 49% (expected: 50.8%) | 1197193 | 0 (0.0%) | 35-301 | 50% (expected: 50.8%) | Escherichia coli |
| 009 | 1083481 | 0 (0.0%) | 35-301 | 37% (expected: 37.9%) | 1083481 | 0 (0.0%) | 35-301 | 37% (expected: 37.9%) | Enterococcus faecium |
| 010 | 684265 | 0 (0.0%) | 35-301 | 37% (expected: 37.9%) | 684265 | 0 (0.0%) | 35-301 | 37% (expected: 37.9%) | Enterococcus faecium |
| 011 | 1181470 | 0 (0.0%) | 35-301 | 36% (expected: 37.9%) | 1181470 | 0 (0.0%) | 35-301 | 36% (expected: 37.9%) | Enterococcus faecium |
| 012 | 1018047 | 0 (0.0%) | 35-301 | 37% (expected: 37.9%) | 1018047 | 0 (0.0%) | 35-301 | 37% (expected: 37.9%) | Enterococcus faecium |
| 013 | 754406 | 0 (0.0%) | 35-301 | 39% (expected: 39.0%) | 754406 | 0 (0.0%) | 35-301 | 39% (expected: 39.0%) | Acinetobacter baumannii |
| 014 | 371267 | 0 (0.0%) | 35-301 | 50% (expected: 50.8%) | 371267 | 0 (0.0%) | 35-301 | 50% (expected: 50.8%) | Escherichia coli |
| 015 | 1138814 | 0 (0.0%) | 35-301 | 65% (expected: 66.6%) | 1138814 | 0 (0.0%) | 35-301 | 66% (expected: 66.6%) | Pseudomonas aeruginosa |
| 016 | 1567126 | 0 (0.0%) | 35-301 | 66% (expected: 66.6%) | 1567126 | 0 (0.0%) | 35-301 | 67% (expected: 66.6%) | Pseudomonas aeruginosa |
| 017 | 1679425 | 0 (0.0%) | 35-301 | 54% (expected: 57.2%) | 1679425 | 0 (0.0%) | 35-301 | 54% (expected: 57.2%) | Klebsiella pneumoniae |
| 018 | 449050 | 0 (0.0%) | 35-301 | 50% (expected: 50.8%) | 449050 | 0 (0.0%) | 35-301 | 50% (expected: 50.8%) | Escherichia coli |
| 019 | 9574247 | 0 (0.0%) | 35-36 | 36% (expected: 37.9%) | 9574247 | 0 (0.0%) | 35-36 | 37% (expected: 37.9%) | Enterococcus faecium |
| 020 | 757445 | 0 (0.0%) | 35-301 | 39% (expected: 39.0%) | 757445 | 0 (0.0%) | 35-301 | 39% (expected: 39.0%) | Acinetobacter baumannii |
| 021 | 1017698 | 0 (0.0%) | 35-301 | 39% (expected: 39.0%) | 1017698 | 0 (0.0%) | 35-301 | 39% (expected: 39.0%) | Acinetobacter baumannii |
| 022 | 745602 | 0 (0.0%) | 35-301 | 32% (expected: 32.7%) | 745602 | 0 (0.0%) | 35-301 | 32% (expected: 32.7%) | Staphylococcus aureus |
| 023 | 1475215 | 0 (0.0%) | 35-301 | 56% (expected: 57.2%) | 1475215 | 0 (0.0%) | 35-301 | 56% (expected: 57.2%) | Klebsiella pneumoniae |
| 024 | 1166271 | 0 (0.0%) | 35-301 | 54% (expected: 55.1%) | 1166271 | 0 (0.0%) | 35-301 | 55% (expected: 55.1%) | Enterobacter cloacae |
| 025 | 8773493 | 0 (0.0%) | 36 | 49% (expected: 50.8%) | 8773493 | 0 (0.0%) | 36 | 49% (expected: 50.8%) | Escherichia coli |
| 026 | 610690 | 0 (0.0%) | 35-301 | 39% (expected: 39.0%) | 610690 | 0 (0.0%) | 35-301 | 39% (expected: 39.0%) | Acinetobacter baumannii |
| 027 | 3052298 | 0 (0.0%) | 148 | 53% (expected: 55.1%) | 3052298 | 0 (0.0%) | 148 | 54% (expected: 55.1%) | Enterobacter cloacae |
| 028 | 5648168 | 0 (0.0%) | 35-36 | 54% (expected: 55.1%) | 5648168 | 0 (0.0%) | 35-36 | 54% (expected: 55.1%) | Enterobacter cloacae |
| 029 | 705151 | 0 (0.0%) | 35-301 | 37% (expected: 37.9%) | 705151 | 0 (0.0%) | 35-301 | 37% (expected: 37.9%) | Enterococcus faecium |
| 030 | 652954 | 0 (0.0%) | 35-301 | 50% (expected: 50.8%) | 652954 | 0 (0.0%) | 35-301 | 50% (expected: 50.8%) | Escherichia coli |
| 031 | 1302240 | 0 (0.0%) | 35-301 | 39% (expected: 39.0%) | 1302240 | 0 (0.0%) | 35-301 | 39% (expected: 39.0%) | Acinetobacter baumannii |
| 032 | 4309870 | 0 (0.0%) | 36 | 65% (expected: 66.6%) | 4309870 | 0 (0.0%) | 36 | 65% (expected: 66.6%) | Pseudomonas aeruginosa |
| 033 | 718970 | 0 (0.0%) | 35-301 | 65% (expected: 66.6%) | 718970 | 0 (0.0%) | 35-301 | 66% (expected: 66.6%) | Pseudomonas aeruginosa |
| 034 | 709670 | 0 (0.0%) | 35-301 | 36% (expected: 37.9%) | 709670 | 0 (0.0%) | 35-301 | 37% (expected: 37.9%) | Enterococcus faecium |
| 035 | 3432997 | 0 (0.0%) | 35 | 65% (expected: 66.6%) | 3432997 | 0 (0.0%) | 35 | 65% (expected: 66.6%) | Pseudomonas aeruginosa |
| 036 | 3279550 | 0 (0.0%) | 35-148 | 38% (expected: 39.0%) | 3279550 | 0 (0.0%) | 35-148 | 38% (expected: 39.0%) | Acinetobacter baumannii |
| 037 | 863615 | 0 (0.0%) | 35-301 | 65% (expected: 66.6%) | 863615 | 0 (0.0%) | 35-301 | 65% (expected: 66.6%) | Pseudomonas aeruginosa |
| 038 | 746755 | 0 (0.0%) | 35-301 | 56% (expected: 57.2%) | 746755 | 0 (0.0%) | 35-301 | 57% (expected: 57.2%) | Klebsiella pneumoniae |
| 039 | 806431 | 0 (0.0%) | 35-301 | 65% (expected: 66.6%) | 806431 | 0 (0.0%) | 35-301 | 65% (expected: 66.6%) | Pseudomonas aeruginosa |
| 040 | 1183600 | 0 (0.0%) | 35-301 | 64% (expected: 66.6%) | 1183600 | 0 (0.0%) | 35-301 | 65% (expected: 66.6%) | Pseudomonas aeruginosa |
| 041 | 1723863 | 0 (0.0%) | 35-301 | 40% (expected: 37.9%) | 1723863 | 0 (0.0%) | 35-301 | 40% (expected: 37.9%) | Enterococcus faecium |
| 042 | 1212517 | 0 (0.0%) | 35-301 | 56% (expected: 57.2%) | 1212517 | 0 (0.0%) | 35-301 | 56% (expected: 57.2%) | Klebsiella pneumoniae |
| 043 | 696629 | 0 (0.0%) | 35-301 | 66% (expected: 66.6%) | 696629 | 0 (0.0%) | 35-301 | 67% (expected: 66.6%) | Pseudomonas aeruginosa |
| 044 | 256200 | 0 (0.0%) | 35-301 | 50% (expected: 50.8%) | 256200 | 0 (0.0%) | 35-301 | 50% (expected: 50.8%) | Escherichia coli |
| 045 | 4615360 | 0 (0.0%) | 148 | 39% (expected: 39.0%) | 4615360 | 0 (0.0%) | 148 | 39% (expected: 39.0%) | Acinetobacter baumannii |
| 046 | 6072009 | 0 (0.0%) | 35-36 | 49% (expected: 50.8%) | 6072009 | 0 (0.0%) | 35-36 | 49% (expected: 50.8%) | Escherichia coli |
| 047 | 5427267 | 0 (0.0%) | 35 | 49% (expected: 50.8%) | 5427267 | 0 (0.0%) | 35 | 49% (expected: 50.8%) | Escherichia coli |
| 048 | 4339802 | 0 (0.0%) | 35-36 | 65% (expected: 66.6%) | 4339802 | 0 (0.0%) | 35-36 | 65% (expected: 66.6%) | Pseudomonas aeruginosa |
| 049 | 4414175 | 0 (0.0%) | 148 | 49% (expected: 50.8%) | 4414175 | 0 (0.0%) | 148 | 49% (expected: 50.8%) | Escherichia coli |
| 050 | 5879270 | 0 (0.0%) | 35-36 | 53% (expected: N/A) | 5879270 | 0 (0.0%) | 35-36 | 54% (expected: N/A) | Cronobacter sakazakii |
| 051 | 775885 | 0 (0.0%) | 35-301 | 32% (expected: 32.7%) | 775885 | 0 (0.0%) | 35-301 | 32% (expected: 32.7%) | Staphylococcus aureus |
| 052 | 1162518 | 0 (0.0%) | 35-301 | 50% (expected: 50.8%) | 1162518 | 0 (0.0%) | 35-301 | 50% (expected: 50.8%) | Escherichia coli |
| 053 | 3614913 | 0 (0.0%) | 148 | 51% (expected: N/A) | 3614913 | 0 (0.0%) | 148 | 51% (expected: N/A) | Citrobacter freundii |
| 054 | 1290830 | 0 (0.0%) | 35-301 | 66% (expected: 66.6%) | 1290830 | 0 (0.0%) | 35-301 | 66% (expected: 66.6%) | Pseudomonas aeruginosa |
| 055 | 1085672 | 0 (0.0%) | 35-301 | 37% (expected: 37.9%) | 1085672 | 0 (0.0%) | 35-301 | 37% (expected: 37.9%) | Enterococcus faecium |
| 056 | 2067045 | 0 (0.0%) | 35-148 | 38% (expected: 39.0%) | 2067045 | 0 (0.0%) | 35-148 | 38% (expected: 39.0%) | Acinetobacter baumannii |
| 057 | 1036532 | 0 (0.0%) | 35-301 | 37% (expected: 37.9%) | 1036532 | 0 (0.0%) | 35-301 | 37% (expected: 37.9%) | Enterococcus faecium |
| 058 | 715564 | 0 (0.0%) | 35-301 | 38% (expected: 39.0%) | 715564 | 0 (0.0%) | 35-301 | 38% (expected: 39.0%) | Acinetobacter baumannii |
| 059 | 1017092 | 0 (0.0%) | 35-301 | 39% (expected: 39.0%) | 1017092 | 0 (0.0%) | 35-301 | 39% (expected: 39.0%) | Acinetobacter baumannii |
| 060 | 485332 | 0 (0.0%) | 35-301 | 50% (expected: 50.8%) | 485332 | 0 (0.0%) | 35-301 | 50% (expected: 50.8%) | Escherichia coli |
| 061 | 7604677 | 0 (0.0%) | 35 | 36% (expected: 37.9%) | 7604677 | 0 (0.0%) | 35 | 36% (expected: 37.9%) | Enterococcus faecium |
| 062 | 3576870 | 0 (0.0%) | 148 | 49% (expected: 50.8%) | 3576870 | 0 (0.0%) | 148 | 49% (expected: 50.8%) | Escherichia coli |
| 063 | 474804 | 0 (0.0%) | 35-301 | 37% (expected: 37.9%) | 474804 | 0 (0.0%) | 35-301 | 37% (expected: 37.9%) | Enterococcus faecium |
| 064 | 1319040 | 0 (0.0%) | 35-301 | 46% (expected: 55.1%) | 1319040 | 0 (0.0%) | 35-301 | 46% (expected: 55.1%) | Enterobacter cloacae |
| 065 | 7761334 | 0 (0.0%) | 35-36 | 49% (expected: 50.8%) | 7761334 | 0 (0.0%) | 35-36 | 49% (expected: 50.8%) | Escherichia coli |
| 066 | 5619607 | 0 (0.0%) | 148 | 38% (expected: 39.0%) | 5619607 | 0 (0.0%) | 148 | 39% (expected: 39.0%) | Acinetobacter baumannii |
| 067 | 791625 | 0 (0.0%) | 35-301 | 39% (expected: 39.0%) | 791625 | 0 (0.0%) | 35-301 | 39% (expected: 39.0%) | Acinetobacter baumannii |
| 068 | 1226647 | 0 (0.0%) | 35-301 | 53% (expected: 57.2%) | 1226647 | 0 (0.0%) | 35-301 | 53% (expected: 57.2%) | Klebsiella pneumoniae |
| 069 | 898577 | 0 (0.0%) | 35-301 | 37% (expected: 37.9%) | 898577 | 0 (0.0%) | 35-301 | 37% (expected: 37.9%) | Enterococcus faecium |
| 070 | 691518 | 0 (0.0%) | 35-301 | 65% (expected: 66.6%) | 691518 | 0 (0.0%) | 35-301 | 66% (expected: 66.6%) | Pseudomonas aeruginosa |
| 071 | 1101941 | 0 (0.0%) | 35-301 | 63% (expected: 66.6%) | 1101941 | 0 (0.0%) | 35-301 | 63% (expected: 66.6%) | Pseudomonas aeruginosa |
| 072 | 974151 | 0 (0.0%) | 35-301 | 50% (expected: 50.8%) | 974151 | 0 (0.0%) | 35-301 | 50% (expected: 50.8%) | Escherichia coli |
| 073 | 6099056 | 0 (0.0%) | 148 | 39% (expected: 39.0%) | 6099056 | 0 (0.0%) | 148 | 39% (expected: 39.0%) | Acinetobacter baumannii |
| 074 | 3978822 | 0 (0.0%) | 35-148 | 38% (expected: 39.0%) | 3978822 | 0 (0.0%) | 35-148 | 38% (expected: 39.0%) | Acinetobacter baumannii |
| 075 | 699958 | 0 (0.0%) | 35-301 | 65% (expected: 66.6%) | 699958 | 0 (0.0%) | 35-301 | 66% (expected: 66.6%) | Pseudomonas aeruginosa |
| 076 | 256117 | 0 (0.0%) | 35-301 | 57% (expected: 57.2%) | 256117 | 0 (0.0%) | 35-301 | 57% (expected: 57.2%) | Klebsiella pneumoniae |
| 077 | 806854 | 0 (0.0%) | 35-301 | 65% (expected: 66.6%) | 806854 | 0 (0.0%) | 35-301 | 66% (expected: 66.6%) | Pseudomonas aeruginosa |
| 078 | 985283 | 0 (0.0%) | 35-301 | 66% (expected: 66.6%) | 985283 | 0 (0.0%) | 35-301 | 67% (expected: 66.6%) | Pseudomonas aeruginosa |
| 079 | 351726 | 0 (0.0%) | 35-301 | 55% (expected: 55.1%) | 351726 | 0 (0.0%) | 35-301 | 55% (expected: 55.1%) | Enterobacter cloacae |
| 080 | 748119 | 0 (0.0%) | 35-301 | 37% (expected: 37.9%) | 748119 | 0 (0.0%) | 35-301 | 37% (expected: 37.9%) | Enterococcus faecium |
| 081 | 1464930 | 0 (0.0%) | 35-301 | 52% (expected: 57.2%) | 1464930 | 0 (0.0%) | 35-301 | 52% (expected: 57.2%) | Klebsiella pneumoniae |
| 082 | 15239057 | 0 (0.0%) | 35 | 36% (expected: 37.9%) | 15239057 | 0 (0.0%) | 35 | 36% (expected: 37.9%) | Enterococcus faecium |
| 083 | 2900121 | 0 (0.0%) | 148 | 55% (expected: 57.2%) | 2900121 | 0 (0.0%) | 148 | 55% (expected: 57.2%) | Klebsiella pneumoniae |
| 084 | 4846266 | 0 (0.0%) | 35-36 | 53% (expected: N/A) | 4846266 | 0 (0.0%) | 35-36 | 53% (expected: N/A) | Klebsiella oxytoca |
| 085 | 845241 | 0 (0.0%) | 35-301 | 37% (expected: 37.9%) | 845241 | 0 (0.0%) | 35-301 | 37% (expected: 37.9%) | Enterococcus faecium |
| 086 | 582645 | 0 (0.0%) | 35-301 | 50% (expected: 50.8%) | 582645 | 0 (0.0%) | 35-301 | 50% (expected: 50.8%) | Escherichia coli |
| 087 | 1217715 | 0 (0.0%) | 35-301 | 56% (expected: 57.2%) | 1217715 | 0 (0.0%) | 35-301 | 56% (expected: 57.2%) | Klebsiella pneumoniae |
| 088 | 895288 | 0 (0.0%) | 35-301 | 37% (expected: 37.9%) | 895288 | 0 (0.0%) | 35-301 | 37% (expected: 37.9%) | Enterococcus faecium |
| 089 | 715834 | 0 (0.0%) | 35-301 | 65% (expected: 66.6%) | 715834 | 0 (0.0%) | 35-301 | 66% (expected: 66.6%) | Pseudomonas aeruginosa |
| 090 | 990543 | 0 (0.0%) | 35-301 | 37% (expected: 37.9%) | 990543 | 0 (0.0%) | 35-301 | 37% (expected: 37.9%) | Enterococcus faecium |
| 091 | 853150 | 0 (0.0%) | 35-301 | 38% (expected: 39.0%) | 853150 | 0 (0.0%) | 35-301 | 38% (expected: 39.0%) | Acinetobacter baumannii |
| 092 | 5463421 | 0 (0.0%) | 35 | 53% (expected: 55.1%) | 5463421 | 0 (0.0%) | 35 | 53% (expected: 55.1%) | Enterobacter cloacae |
| 093 | 760367 | 0 (0.0%) | 35-301 | 37% (expected: 37.9%) | 760367 | 0 (0.0%) | 35-301 | 37% (expected: 37.9%) | Enterococcus faecium |
| 094 | 1265719 | 0 (0.0%) | 35-301 | 54% (expected: N/A) | 1265719 | 0 (0.0%) | 35-301 | 54% (expected: N/A) | Klebsiella oxytoca |
| 095 | 6754500 | 0 (0.0%) | 36 | 54% (expected: 55.1%) | 6754500 | 0 (0.0%) | 36 | 54% (expected: 55.1%) | Enterobacter cloacae |
| 096 | 588803 | 0 (0.0%) | 35-301 | 38% (expected: 39.0%) | 588803 | 0 (0.0%) | 35-301 | 38% (expected: 39.0%) | Acinetobacter baumannii |
| 097 | 938160 | 0 (0.0%) | 35-301 | 39% (expected: 39.0%) | 938160 | 0 (0.0%) | 35-301 | 39% (expected: 39.0%) | Acinetobacter baumannii |
| 098 | 760496 | 0 (0.0%) | 35-301 | 66% (expected: 66.6%) | 760496 | 0 (0.0%) | 35-301 | 66% (expected: 66.6%) | Pseudomonas aeruginosa |
| 099 | 7665674 | 0 (0.0%) | 35-36 | 44% (expected: 39.0%) | 7665674 | 0 (0.0%) | 35-36 | 45% (expected: 39.0%) | Acinetobacter baumannii |
| 100 | 3461499 | 0 (0.0%) | 35-36 | 65% (expected: 66.6%) | 3461499 | 0 (0.0%) | 35-36 | 65% (expected: 66.6%) | Pseudomonas aeruginosa |

---

The **FastQC summary table** provides an overview of key quality metrics for each of your sequencing samples.
This table is designed to help you quickly assess the quality of your raw sequencing data before proceeding to downstream analyses.

#### Table Columns:

- **Total Sequences (R1 and R2):** The total number of sequences (reads) for each read direction—forward (R1) and reverse (R2).
- **Sequences Flagged as Poor Quality (R1 and R2):** The number and percentage of sequences flagged as poor quality by FastQC, displayed as `Number (Percentage%)`.
- **Sequence Length (R1 and R2):** The length of the sequences for each read direction. Consistent lengths indicate proper sequencing.
- **%GC Content (R1 and R2):** The percentage of guanine (G) and cytosine (C) nucleotides in the sequences, displayed as `Actual% (expected: Expected%)`.
- **Species:** The species name associated with each sample.

#### Color Coding in %GC Columns:

To assist in data interpretation, the **%GC** content cells are color-coded:

- Light Green: The actual %GC is within **6%** of the expected value, suggesting consistency with the species' genomic characteristics.
- Light Red: The actual %GC differs from the expected value by more than **6%**. This does not necessarily indicate a problem but suggests further investigation may be needed.

#### Interpreting the Table:

Use the table to evaluate sequencing quality:

- **Low Poor Quality Sequences:** Indicates good sequencing quality.
- **High Poor Quality Sequences:** May require investigation into sequencing conditions or sample integrity.
- **%GC Deviations (Red):** Could suggest contamination, sequencing bias, or natural genomic variation.

#### Action Steps for Unusual Results:

- Review sample labeling and preparation procedures.
- Examine FastQC detailed reports for additional metrics.
- Verify sample purity and check for contamination sources.
- Consult with the sequencing facility if necessary.

**Note:** A red %GC cell is a prompt to investigate further and does not necessarily indicate an issue. Natural variation or technical factors might account for the deviation.

The FastQC summary table is a valuable tool for quickly assessing the quality and integrity of your sequencing data. While the color-coded indicators provide visual cues for potential issues, they are meant to guide further investigation rather than serve as definitive judgments.
Remember: A red-colored cell in the %GC column is a prompt to look closer—it does not necessarily mean there is a problem. It is possible that the observed %GC variation is due to acceptable natural variation or technical factors that can be accounted for in your analysis.

#### N50 Contig Length Distribution

  

#### Average Contig Coverage Distribution

  

#### Contig Lengths for Acinetobacter baumannii

  

#### Contig Lengths for Escherichia coli

  

#### Contig Lengths for Enterococcus faecium

  

#### Contig Lengths for Pseudomonas aeruginosa

  

#### Contig Lengths for Klebsiella pneumoniae

  

#### Contig Lengths for Staphylococcus aureus

  

#### Contig Lengths for Klebsiella oxytoca

  

#### Contig Lengths for Enterobacter cloacae

  

#### Contig Lengths for Unknown

  

#### Contig Lengths for Cronobacter sakazakii

  

#### Contig Lengths for Citrobacter freundii

  


---

The contig assembly quality plots provide insights into the quality and characteristics of the assembled genomes for each species in your dataset. These plots help you assess the assembly process, identify potential issues, and compare assembly metrics across samples and species.

#### 1. Cumulative Contig Length Plots

For each species, a cumulative contig length plot is generated, displaying the cumulative sum of contig lengths sorted in descending order for each sample.

- **X-Axis (Contig Index):** Represents the index of contigs sorted from largest to smallest.
- **Y-Axis (Cumulative Contig Length):** Shows the cumulative length of contigs up to that index.

The plot highlights the **N50** value for each sample:

- **Red Segment:** Indicates the portion of the cumulative length up to the N50 contig. This is the contig length at which half of the total assembly length is reached.
- **Remaining Line:** Represents the rest of the contigs beyond the N50.

**Interpreting the Plot:**

- A steeper curve suggests fewer contigs are needed to reach the total assembly length, indicating a more contiguous assembly.
- A longer red segment (up to N50) means larger contigs contribute significantly to the assembly.
- Comparing samples can reveal variations in assembly quality across different samples of the same species.

#### 2. N50 Contig Length Distribution Box Plot

This plot displays the distribution of N50 contig lengths across all samples, grouped by species.

- **X-Axis (Species):** Different species in your dataset.
- **Y-Axis (N50 Contig Length):** The N50 values for the samples.

**Interpreting the Plot:**

- Higher N50 values indicate better assembly continuity.
- The spread of data points shows variability between samples.
- Outliers can identify samples with unusually high or low N50 values, which may need further investigation.

#### 3. N50 Contig Coverage Box Plot

This plot illustrates the distribution of coverage at the N50 contig across all samples, grouped by species.

- **X-Axis (Species):** Different species in your dataset.
- **Y-Axis (N50 Contig Coverage):** Coverage values at the N50 contig for the samples.

**Interpreting the Plot:**

- Consistent coverage values suggest uniform sequencing depth.
- Variations in coverage may indicate sequencing biases or issues with library preparation.
- Outliers may highlight samples requiring further quality assessment.

#### Important Notes

**N50 Value:** The N50 is a commonly used metric in genomics to assess the quality of genome assemblies. It is the contig length such that 50% of the total assembly length is contained in contigs equal to or larger than this length.

**Interpreting High N50 and Coverage:** While higher N50 and consistent coverage are generally positive indicators, they should be interpreted in the context of the organism's genome and the sequencing technology used.

#### Action Steps if Issues are Observed

- Review sequencing data quality and consider re-sequencing if necessary.
- Check for contamination or mixed samples if unexpected results are observed.
- Adjust assembly parameters or use alternative assembly tools for improvement.

If you have questions or need assistance in interpreting these plots, please consult with a bioinformatics specialist.

| GENE | ACCESSION | PRODUCT | %IDENTITY | %COVERAGE | RESISTANCE | START | END | STRAND | PREDICTION\_SOURCE | SAMPLE | SPECIES | SUBTYPE | CONTIG | SPECIES\_NAME |
| --- | --- | --- | --- | --- | --- | --- | --- | --- | --- | --- | --- | --- | --- | --- |
| aph(2'')-Ih | NG\_047408.1 | aminoglycoside O-phosphotransferase APH(2'')-Ih | 82.99 | 82.21 | AMIKACIN;GENTAMICIN;KANAMYCIN;TOBRAMYCIN | 545 | 1279 | + | NCBI | 001 | efaecium | 203 | 109 | Enterococcus faecium |
| vanY-A | NG\_048516.1 | D-Ala-D-Ala carboxypeptidase VanY-A | 100.00 | 100.00 | VANCOMYCIN | 10446 | 11357 | - | NCBI | 001 | efaecium | 203 | 68 | Enterococcus faecium |
| vanZ-A | NG\_048534.1 | glycopeptide resistance protein VanZ-A | 100.00 | 100.00 | VANCOMYCIN | 9808 | 10293 | - | NCBI | 001 | efaecium | 203 | 68 | Enterococcus faecium |
| catA7 | NG\_047568.1 | type A-7 chloramphenicol O-acetyltransferase | 100.00 | 100.00 | CHLORAMPHENICOL | 3380 | 4027 | + | NCBI | 001 | efaecium | 203 | 68 | Enterococcus faecium |
| tet(M) | NG\_048213.1 | tetracycline resistance ribosomal protection protein Tet(M) | 99.79 | 100.00 | TETRACYCLINE | 19176 | 21095 | + | NCBI | 001 | efaecium | 203 | 19 | Enterococcus faecium |
| eat(A) | NG\_047762.1 | ABC-F type ribosomal protection protein Eat(A) | 100.00 | 100.00 | PLEUROMUTILIN | 27622 | 29124 | - | NCBI | 001 | efaecium | 203 | 16 | Enterococcus faecium |
| aacA-ENT1 | NG\_052371.1 | aminoglycoside 6'-N-acetyltransferase | 100.00 | 100.00 | AMINOGLYCOSIDE | 34196 | 34744 | - | NCBI | 001 | efaecium | 203 | 11 | Enterococcus faecium |
| msr(C) | NG\_048003.1 | ABC-F type ribosomal protection protein Msr(C) | 98.92 | 100.00 | MACROLIDE | 80919 | 82397 | - | NCBI | 001 | efaecium | 203 | 1 | Enterococcus faecium |
| vanS-A | NG\_048425.1 | VanA-type vancomycin resistance histidine kinase VanS | 100.00 | 100.00 | VANCOMYCIN | 852 | 2006 | + | NCBI | 001 | efaecium | 203 | 95 | Enterococcus faecium |
| vanH-A | NG\_048372.1 | D-lactate dehydrogenase VanH-A | 100.00 | 100.00 | VANCOMYCIN | 2221 | 3189 | + | NCBI | 001 | efaecium | 203 | 95 | Enterococcus faecium |
| vanA | NG\_048323.1 | D-alanine--(R)-lactate ligase VanA | 100.00 | 100.00 | VANCOMYCIN | 3182 | 4213 | + | NCBI | 001 | efaecium | 203 | 95 | Enterococcus faecium |
| vanX-A | NG\_048477.1 | D-Ala-D-Ala dipeptidase VanX-A | 100.00 | 100.00 | VANCOMYCIN | 4219 | 4827 | + | NCBI | 001 | efaecium | 203 | 95 | Enterococcus faecium |
| ant(6)-Ia | NG\_047393.1 | aminoglycoside nucleotidyltransferase ANT(6)-Ia | 100.00 | 100.00 | STREPTOMYCIN | 638 | 1546 | - | NCBI | 001 | efaecium | 203 | 107 | Enterococcus faecium |
| vanR-A | NG\_048399.1 | VanA-type vancomycin resistance DNA-binding response regulator VanR | 100.00 | 100.00 | VANCOMYCIN | 179 | 874 | + | NCBI | 001 | efaecium | 203 | 95 | Enterococcus faecium |
| aph(2'')-Ih | NG\_047408.1 | aminoglycoside O-phosphotransferase APH(2'')-Ih | 82.43 | 89.15 | AMIKACIN;GENTAMICIN;KANAMYCIN;TOBRAMYCIN | 2286 | 3082 | + | NCBI | 001 | efaecium | 203 | 109 | Enterococcus faecium |
| erm(B) | NG\_047801.1 | 23S rRNA (adenine(2058)-N(6))-methyltransferase Erm(B) | 99.60 | 99.87 | MACROLIDE | 290 | 1035 | - | NCBI | 001 | efaecium | 203 | 141 | Enterococcus faecium |
| dfrF | NG\_047755.1 | trimethoprim-resistant dihydrofolate reductase DfrF | 99.60 | 100.00 | TRIMETHOPRIM | 2753 | 3248 | - | NCBI | 001 | efaecium | 203 | 77 | Enterococcus faecium |
| blaEC-15 | NG\_049081.1 | class C extended-spectrum beta-lactamase EC-15 | 98.50 | 100.00 | CEPHALOSPORIN | 176235 | 177368 | - | NCBI | 002 | ecoli\_achtman\_4 | 167 | 3 | Escherichia coli |
| tet(A) | NG\_048159.1 | tetracycline efflux MFS transporter Tet(A) | 100.00 | 100.00 | TETRACYCLINE | 12459 | 13658 | - | NCBI | 002 | ecoli\_achtman\_4 | 167 | 41 | Escherichia coli |
| ble-MBL | NG\_047559.1 | bleomycin binding protein Ble-MBL | 100.00 | 100.00 | BLEOMYCIN | 4047 | 4412 | - | NCBI | 002 | ecoli\_achtman\_4 | 167 | 65 | Escherichia coli |
| blaNDM-5 | NG\_049337.1 | subclass B1 metallo-beta-lactamase NDM-5 | 100.00 | 100.00 | CARBAPENEM | 4416 | 5228 | - | NCBI | 002 | ecoli\_achtman\_4 | 167 | 65 | Escherichia coli |
| erm(B) | NG\_047804.1 | 23S rRNA (adenine(2058)-N(6))-methyltransferase Erm(B) | 99.86 | 100.00 | MACROLIDE | 417 | 1154 | + | NCBI | 002 | ecoli\_achtman\_4 | 167 | 71 | Escherichia coli |
| blaTEM-1 | NG\_050145.1 | class A broad-spectrum beta-lactamase TEM-1 | 100.00 | 100.00 | BETA-LACTAM | 1108 | 1968 | + | NCBI | 002 | ecoli\_achtman\_4 | 167 | 72 | Escherichia coli |
| rmtB1 | NG\_048058.1 | 16S rRNA (guanine(1405)-N(7))-methyltransferase RmtB1 | 100.00 | 100.00 | AMINOGLYCOSIDE | 2138 | 2893 | + | NCBI | 002 | ecoli\_achtman\_4 | 167 | 72 | Escherichia coli |
| mph(A) | NG\_047986.1 | Mph(A) family macrolide 2'-phosphotransferase | 98.71 | 100.00 | MACROLIDE | 196 | 1126 | + | NCBI | 002 | ecoli\_achtman\_4 | 167 | 74 | Escherichia coli |
| dfrA12 | NG\_047689.1 | trimethoprim-resistant dihydrofolate reductase DfrA12 | 100.00 | 100.00 | TRIMETHOPRIM | 133 | 630 | + | NCBI | 002 | ecoli\_achtman\_4 | 167 | 82 | Escherichia coli |
| dfrA25 | NG\_047721.1 | trimethoprim-resistant dihydrofolate reductase DfrA25 | 100.00 | 100.00 | TRIMETHOPRIM | 144 | 617 | + | NCBI | 002 | ecoli\_achtman\_4 | 167 | 107 | Escherichia coli |
| sul1 | NG\_048082.1 | sulfonamide-resistant dihydropteroate synthase Sul1 | 100.00 | 100.00 | SULFONAMIDE | 455 | 1294 | + | NCBI | 002 | ecoli\_achtman\_4 | 167 | 88 | Escherichia coli |
| aadA2 | NG\_047343.1 | ANT(3'')-Ia family aminoglycoside nucleotidyltransferase AadA2 | 100.00 | 100.00 | STREPTOMYCIN | 1038 | 1829 | + | NCBI | 002 | ecoli\_achtman\_4 | 167 | 82 | Escherichia coli |
| ant(3'')-IIa | NG\_054648.1 | aminoglycoside nucleotidyltransferase ANT(3'')-IIa | 99.11 | 100.00 | SPECTINOMYCIN;STREPTOMYCIN | 34658 | 35446 | - | NCBI | 003 | abaumannii\_2 | - | 5 | Acinetobacter baumannii |
| blaADC-26 | NG\_048650.1 | class C extended-spectrum beta-lactamase ADC-26 | 98.18 | 100.00 | CEPHALOSPORIN | 1762148 | 1763299 | + | NCBI | 003 | abaumannii\_2 | - | 1 | Acinetobacter baumannii |
| blaOXA-51 | NG\_049788.1 | OXA-51 family carbapenem-hydrolyzing class D beta-lactamase OXA-51 | 100.00 | 100.00 | CARBAPENEM | 780934 | 781758 | - | NCBI | 003 | abaumannii\_2 | - | 1 | Acinetobacter baumannii |
| sul2 | NG\_051852.1 | sulfonamide-resistant dihydropteroate synthase Sul2 | 100.00 | 100.00 | SULFONAMIDE | 146 | 961 | + | NCBI | 004 | abaumannii\_2 | 492 | 53 | Acinetobacter baumannii |
| armA | NG\_047476.1 | ArmA family 16S rRNA (guanine(1405)-N(7))-methyltransferase | 100.00 | 100.00 | GENTAMICIN | 7904 | 8677 | + | NCBI | 004 | abaumannii\_2 | 492 | 38 | Acinetobacter baumannii |
| sul1 | NG\_048082.1 | sulfonamide-resistant dihydropteroate synthase Sul1 | 100.00 | 100.00 | SULFONAMIDE | 3708 | 4547 | + | NCBI | 004 | abaumannii\_2 | 492 | 38 | Acinetobacter baumannii |
| aadA2 | NG\_047343.1 | ANT(3'')-Ia family aminoglycoside nucleotidyltransferase AadA2 | 100.00 | 100.00 | STREPTOMYCIN | 2412 | 3203 | + | NCBI | 004 | abaumannii\_2 | 492 | 38 | Acinetobacter baumannii |
| dfrA12 | NG\_047689.1 | trimethoprim-resistant dihydrofolate reductase DfrA12 | 100.00 | 100.00 | TRIMETHOPRIM | 1507 | 2004 | + | NCBI | 004 | abaumannii\_2 | 492 | 38 | Acinetobacter baumannii |
| ant(3'')-IIa | NG\_054646.1 | aminoglycoside nucleotidyltransferase ANT(3'')-IIa | 99.24 | 100.00 | SPECTINOMYCIN;STREPTOMYCIN | 31793 | 32581 | - | NCBI | 004 | abaumannii\_2 | 492 | 28 | Acinetobacter baumannii |
| tet(B) | NG\_048161.1 | tetracycline efflux MFS transporter Tet(B) | 100.00 | 99.50 | TETRACYCLINE | 64335 | 65534 | + | NCBI | 004 | abaumannii\_2 | 492 | 18 | Acinetobacter baumannii |
| aph(6)-Id | NG\_047464.1 | aminoglycoside O-phosphotransferase APH(6)-Id | 100.00 | 100.00 | STREPTOMYCIN | 60126 | 60962 | + | NCBI | 004 | abaumannii\_2 | 492 | 18 | Acinetobacter baumannii |
| aph(3'')-Ib | NG\_056002.2 | aminoglycoside O-phosphotransferase APH(3'')-Ib | 99.88 | 98.31 | STREPTOMYCIN | 59313 | 60126 | + | NCBI | 004 | abaumannii\_2 | 492 | 18 | Acinetobacter baumannii |
| blaOXA-66 | NG\_049806.1 | OXA-51 family carbapenem-hydrolyzing class D beta-lactamase OXA-66 | 100.00 | 100.00 | CARBAPENEM | 114278 | 115102 | + | NCBI | 004 | abaumannii\_2 | 492 | 1 | Acinetobacter baumannii |
| blaOXA-72 | NG\_049813.1 | OXA-24 family carbapenem-hydrolyzing class D beta-lactamase OXA-72 | 100.00 | 100.00 | CARBAPENEM | 8194 | 9021 | - | NCBI | 004 | abaumannii\_2 | 492 | 44 | Acinetobacter baumannii |
| blaADC-30 | NG\_048652.1 | class C extended-spectrum beta-lactamase ADC-30 | 100.00 | 100.00 | CEPHALOSPORIN | 137 | 1288 | + | NCBI | 004 | abaumannii\_2 | 492 | 2 | Acinetobacter baumannii |
| mph(E) | NG\_064660.1 | Mph(E) family macrolide 2'-phosphotransferase | 100.00 | 100.00 | MACROLIDE | 12507 | 13391 | + | NCBI | 004 | abaumannii\_2 | 492 | 38 | Acinetobacter baumannii |
| msr(E) | NG\_048007.1 | ABC-F type ribosomal protection protein Msr(E) | 100.00 | 100.00 | MACROLIDE | 10976 | 12451 | + | NCBI | 004 | abaumannii\_2 | 492 | 38 | Acinetobacter baumannii |
| CMY2-MIR-ACT-EC | NG\_051160.1 | CMY2/MIR/ACT/EC family class C beta-lactamase | 100.00 | 100.00 | BETA-LACTAM | 64158 | 65303 | + | NCBI | 005 | cronobacter | - | 1 | Cronobacter sakazakii |
| fosA\_gen | NG\_047881.1 | FosA family fosfomycin resistance glutathione transferase | 89.52 | 100.00 | FOSFOMYCIN | 369092 | 369511 | - | NCBI | 005 | cronobacter | - | 3 | Cronobacter sakazakii |
| oqxB20 | NG\_050439.1 | multidrug efflux RND transporter permease subunit OqxB20 | 88.15 | 99.30 | PHENICOL;QUINOLONE | 47524 | 50654 | - | NCBI | 005 | cronobacter | - | 18 | Cronobacter sakazakii |
| oqxA10 | NG\_050418.1 | multidrug efflux RND transporter periplasmic adaptor subunit OqxA10 | 84.18 | 100.00 | PHENICOL;QUINOLONE | 50678 | 51853 | - | NCBI | 005 | cronobacter | - | 18 | Cronobacter sakazakii |
| blaPOM-2 | NG\_065446.1 | subclass B3 metallo-beta-lactamase POM-2 | 97.79 | 100.00 | CARBAPENEM | 19923 | 20783 | - | NCBI | 007 | - | - | 3 | Unknown |
| blaEC-15 | NG\_049081.1 | class C extended-spectrum beta-lactamase EC-15 | 98.50 | 100.00 | CEPHALOSPORIN | 38368 | 39501 | + | NCBI | 008 | ecoli\_achtman\_4 | 4981 | 7 | Escherichia coli |
| qnrS1 | NG\_050543.1 | quinolone resistance pentapeptide repeat protein QnrS1 | 100.00 | 100.00 | QUINOLONE | 3727 | 4383 | - | NCBI | 008 | ecoli\_achtman\_4 | 4981 | 66 | Escherichia coli |
| blaTEM-150 | NG\_050194.1 | class A beta-lactamase TEM-150 | 99.88 | 100.00 | BETA-LACTAM | 121926 | 122786 | - | NCBI | 008 | ecoli\_achtman\_4 | 4981 | 13 | Escherichia coli |
| sul2 | NG\_051852.1 | sulfonamide-resistant dihydropteroate synthase Sul2 | 100.00 | 100.00 | SULFONAMIDE | 120949 | 121764 | - | NCBI | 008 | ecoli\_achtman\_4 | 4981 | 13 | Escherichia coli |
| aph(3'')-Ib | NG\_056002.2 | aminoglycoside O-phosphotransferase APH(3'')-Ib | 100.00 | 100.00 | STREPTOMYCIN | 120085 | 120912 | - | NCBI | 008 | ecoli\_achtman\_4 | 4981 | 13 | Escherichia coli |
| aph(6)-Id | NG\_047464.1 | aminoglycoside O-phosphotransferase APH(6)-Id | 100.00 | 100.00 | STREPTOMYCIN | 119249 | 120085 | - | NCBI | 008 | ecoli\_achtman\_4 | 4981 | 13 | Escherichia coli |
| sul2 | NG\_051852.1 | sulfonamide-resistant dihydropteroate synthase Sul2 | 100.00 | 100.00 | SULFONAMIDE | 2295 | 3110 | + | NCBI | 008 | ecoli\_achtman\_4 | 4981 | 13 | Escherichia coli |
| tet(A) | NG\_048153.1 | tetracycline efflux MFS transporter Tet(A) | 100.00 | 100.00 | TETRACYCLINE | 116714 | 117913 | - | NCBI | 008 | ecoli\_achtman\_4 | 4981 | 13 | Escherichia coli |
| floR | NG\_047878.1 | chloramphenicol/florfenicol efflux MFS transporter FloR | 100.00 | 100.00 | CHLORAMPHENICOL;FLORFENICOL | 114900 | 116114 | + | NCBI | 008 | ecoli\_achtman\_4 | 4981 | 13 | Escherichia coli |
| floR | NG\_047878.1 | chloramphenicol/florfenicol efflux MFS transporter FloR | 100.00 | 100.00 | CHLORAMPHENICOL;FLORFENICOL | 7945 | 9159 | - | NCBI | 008 | ecoli\_achtman\_4 | 4981 | 13 | Escherichia coli |
| tet(A) | NG\_048153.1 | tetracycline efflux MFS transporter Tet(A) | 100.00 | 100.00 | TETRACYCLINE | 6146 | 7345 | + | NCBI | 008 | ecoli\_achtman\_4 | 4981 | 13 | Escherichia coli |
| aph(6)-Id | NG\_047464.1 | aminoglycoside O-phosphotransferase APH(6)-Id | 100.00 | 100.00 | STREPTOMYCIN | 3974 | 4810 | + | NCBI | 008 | ecoli\_achtman\_4 | 4981 | 13 | Escherichia coli |
| blaTEM-150 | NG\_050194.1 | class A beta-lactamase TEM-150 | 99.88 | 100.00 | BETA-LACTAM | 1273 | 2133 | + | NCBI | 008 | ecoli\_achtman\_4 | 4981 | 13 | Escherichia coli |
| aph(3'')-Ib | NG\_056002.2 | aminoglycoside O-phosphotransferase APH(3'')-Ib | 100.00 | 100.00 | STREPTOMYCIN | 3147 | 3974 | + | NCBI | 008 | ecoli\_achtman\_4 | 4981 | 13 | Escherichia coli |
| blaCTX-M-15 | NG\_048935.1 | class A extended-spectrum beta-lactamase CTX-M-15 | 100.00 | 100.00 | CEPHALOSPORIN | 132255 | 133130 | + | NCBI | 008 | ecoli\_achtman\_4 | 4981 | 10 | Escherichia coli |
| vanR-A | NG\_048399.1 | VanA-type vancomycin resistance DNA-binding response regulator VanR | 100.00 | 100.00 | VANCOMYCIN | 179 | 874 | + | NCBI | 009 | efaecium | 80 | 99 | Enterococcus faecium |
| vanS-A | NG\_048425.1 | VanA-type vancomycin resistance histidine kinase VanS | 100.00 | 100.00 | VANCOMYCIN | 852 | 2006 | + | NCBI | 009 | efaecium | 80 | 99 | Enterococcus faecium |
| catA7 | NG\_047568.1 | type A-7 chloramphenicol O-acetyltransferase | 100.00 | 100.00 | CHLORAMPHENICOL | 7760 | 8407 | - | NCBI | 009 | efaecium | 80 | 74 | Enterococcus faecium |
| vanZ-A | NG\_048534.1 | glycopeptide resistance protein VanZ-A | 100.00 | 100.00 | VANCOMYCIN | 1494 | 1979 | + | NCBI | 009 | efaecium | 80 | 74 | Enterococcus faecium |
| vanY-A | NG\_048516.1 | D-Ala-D-Ala carboxypeptidase VanY-A | 100.00 | 100.00 | VANCOMYCIN | 430 | 1341 | + | NCBI | 009 | efaecium | 80 | 74 | Enterococcus faecium |
| msr(C) | NG\_048003.1 | ABC-F type ribosomal protection protein Msr(C) | 98.99 | 100.00 | MACROLIDE | 6152 | 7630 | - | NCBI | 009 | efaecium | 80 | 46 | Enterococcus faecium |
| aacA-ENT1 | NG\_052371.1 | aminoglycoside 6'-N-acetyltransferase | 100.00 | 100.00 | AMINOGLYCOSIDE | 16411 | 16959 | + | NCBI | 009 | efaecium | 80 | 17 | Enterococcus faecium |
| eat(A) | NG\_047762.1 | ABC-F type ribosomal protection protein Eat(A) | 99.80 | 100.00 | PLEUROMUTILIN | 40063 | 41565 | + | NCBI | 009 | efaecium | 80 | 9 | Enterococcus faecium |
| vanH-A | NG\_048372.1 | D-lactate dehydrogenase VanH-A | 100.00 | 100.00 | VANCOMYCIN | 2221 | 3189 | + | NCBI | 009 | efaecium | 80 | 99 | Enterococcus faecium |
| vanA | NG\_048323.1 | D-alanine--(R)-lactate ligase VanA | 100.00 | 100.00 | VANCOMYCIN | 3182 | 4213 | + | NCBI | 009 | efaecium | 80 | 99 | Enterococcus faecium |
| vanX-A | NG\_048477.1 | D-Ala-D-Ala dipeptidase VanX-A | 100.00 | 100.00 | VANCOMYCIN | 4219 | 4827 | + | NCBI | 009 | efaecium | 80 | 99 | Enterococcus faecium |
| aph(3')-IIIa | NG\_047418.1 | aminoglycoside O-phosphotransferase APH(3')-IIIa | 100.00 | 100.00 | AMIKACIN;KANAMYCIN | 1185 | 1979 | - | NCBI | 009 | efaecium | 80 | 120 | Enterococcus faecium |
| ant(6)-Ia | NG\_047393.1 | aminoglycoside nucleotidyltransferase ANT(6)-Ia | 100.00 | 100.00 | STREPTOMYCIN | 140 | 1048 | - | NCBI | 009 | efaecium | 80 | 129 | Enterococcus faecium |
| erm(B) | NG\_047801.1 | 23S rRNA (adenine(2058)-N(6))-methyltransferase Erm(B) | 99.60 | 99.87 | MACROLIDE | 162 | 907 | + | NCBI | 009 | efaecium | 80 | 158 | Enterococcus faecium |
| erm(B) | NG\_047804.1 | 23S rRNA (adenine(2058)-N(6))-methyltransferase Erm(B) | 99.86 | 100.00 | MACROLIDE | 442 | 1179 | - | NCBI | 010 | efaecium | 117 | 120 | Enterococcus faecium |
| aacA-ENT1 | NG\_052371.1 | aminoglycoside 6'-N-acetyltransferase | 100.00 | 100.00 | AMINOGLYCOSIDE | 62240 | 62788 | - | NCBI | 010 | efaecium | 117 | 2 | Enterococcus faecium |
| msr(C) | NG\_048003.1 | ABC-F type ribosomal protection protein Msr(C) | 98.92 | 100.00 | MACROLIDE | 80310 | 81788 | - | NCBI | 010 | efaecium | 117 | 4 | Enterococcus faecium |
| dfrG | NG\_047756.1 | trimethoprim-resistant dihydrofolate reductase DfrG | 100.00 | 100.00 | TRIMETHOPRIM | 4157 | 4654 | - | NCBI | 010 | efaecium | 117 | 6 | Enterococcus faecium |
| eat(A) | NG\_047762.1 | ABC-F type ribosomal protection protein Eat(A) | 100.00 | 100.00 | PLEUROMUTILIN | 60152 | 61654 | - | NCBI | 010 | efaecium | 117 | 5 | Enterococcus faecium |
| vanX-B | NG\_048483.1 | D-Ala-D-Ala dipeptidase VanX-B | 100.00 | 100.00 | VANCOMYCIN | 462 | 1070 | - | NCBI | 010 | efaecium | 117 | 93 | Enterococcus faecium |
| vanB | NG\_048341.1 | D-alanine--(R)-lactate ligase VanB | 99.90 | 100.00 | VANCOMYCIN | 1076 | 2104 | - | NCBI | 010 | efaecium | 117 | 93 | Enterococcus faecium |
| vanY-B | NG\_048519.1 | D-Ala-D-Ala carboxypeptidase VanY-B | 99.88 | 100.00 | VANCOMYCIN | 3910 | 4716 | - | NCBI | 010 | efaecium | 117 | 93 | Enterococcus faecium |
| aph(3')-IIIa | NG\_047418.1 | aminoglycoside O-phosphotransferase APH(3')-IIIa | 100.00 | 100.00 | AMIKACIN;KANAMYCIN | 8247 | 9041 | + | NCBI | 010 | efaecium | 117 | 61 | Enterococcus faecium |
| sat4 | NG\_048072.1 | streptothricin N-acetyltransferase Sat4 | 100.00 | 100.00 | STREPTOTHRICIN | 7612 | 8154 | + | NCBI | 010 | efaecium | 117 | 61 | Enterococcus faecium |
| ant(6)-Ia | NG\_047393.1 | aminoglycoside nucleotidyltransferase ANT(6)-Ia | 100.00 | 100.00 | STREPTOMYCIN | 6707 | 7615 | + | NCBI | 010 | efaecium | 117 | 61 | Enterococcus faecium |
| vanS-B | NG\_048439.1 | VanB-type vancomycin resistance histidine kinase VanS | 99.85 | 100.00 | VANCOMYCIN | 36331 | 37674 | + | NCBI | 010 | efaecium | 117 | 24 | Enterococcus faecium |
| vanH-B | NG\_048379.1 | D-lactate dehydrogenase VanH-B | 100.00 | 100.00 | VANCOMYCIN | 2097 | 3068 | - | NCBI | 010 | efaecium | 117 | 93 | Enterococcus faecium |
| vanR-B | NG\_048410.1 | VanB-type vancomycin resistance response regulator transcription factor VanR | 99.70 | 100.00 | VANCOMYCIN | 35669 | 36331 | + | NCBI | 010 | efaecium | 117 | 24 | Enterococcus faecium |
| aph(2'')-Ih | NG\_047408.1 | aminoglycoside O-phosphotransferase APH(2'')-Ih | 82.43 | 89.15 | AMIKACIN;GENTAMICIN;KANAMYCIN;TOBRAMYCIN | 335 | 1131 | - | NCBI | 010 | efaecium | 117 | 117 | Enterococcus faecium |
| vanW-B | NG\_048472.1 | glycopeptide resistance accessory protein VanW-B | 99.76 | 100.00 | VANCOMYCIN | 3065 | 3892 | - | NCBI | 010 | efaecium | 117 | 93 | Enterococcus faecium |
| sat4 | NG\_048070.1 | streptothricin N-acetyltransferase Sat4 | 100.00 | 81.22 | STREPTOTHRICIN | 1 | 441 | + | NCBI | 011 | efaecium | 117 | 82 | Enterococcus faecium |
| catA7 | NG\_047568.1 | type A-7 chloramphenicol O-acetyltransferase | 100.00 | 100.00 | CHLORAMPHENICOL | 7677 | 8324 | - | NCBI | 011 | efaecium | 117 | 66 | Enterococcus faecium |
| vanZ-A | NG\_048534.1 | glycopeptide resistance protein VanZ-A | 100.00 | 100.00 | VANCOMYCIN | 1411 | 1896 | + | NCBI | 011 | efaecium | 117 | 66 | Enterococcus faecium |
| vanY-A | NG\_048516.1 | D-Ala-D-Ala carboxypeptidase VanY-A | 100.00 | 100.00 | VANCOMYCIN | 347 | 1258 | + | NCBI | 011 | efaecium | 117 | 66 | Enterococcus faecium |
| aacA-ENT1 | NG\_052371.1 | aminoglycoside 6'-N-acetyltransferase | 100.00 | 100.00 | AMINOGLYCOSIDE | 62240 | 62788 | - | NCBI | 011 | efaecium | 117 | 12 | Enterococcus faecium |
| dfrG | NG\_047756.1 | trimethoprim-resistant dihydrofolate reductase DfrG | 100.00 | 100.00 | TRIMETHOPRIM | 4157 | 4654 | - | NCBI | 011 | efaecium | 117 | 9 | Enterococcus faecium |
| vanS-A | NG\_048425.1 | VanA-type vancomycin resistance histidine kinase VanS | 100.00 | 100.00 | VANCOMYCIN | 852 | 2006 | + | NCBI | 011 | efaecium | 117 | 94 | Enterococcus faecium |
| aph(2'')-Ih | NG\_047408.1 | aminoglycoside O-phosphotransferase APH(2'')-Ih | 82.43 | 89.15 | AMIKACIN;GENTAMICIN;KANAMYCIN;TOBRAMYCIN | 545 | 1341 | + | NCBI | 011 | efaecium | 117 | 130 | Enterococcus faecium |
| ant(6)-Ia | NG\_047393.1 | aminoglycoside nucleotidyltransferase ANT(6)-Ia | 100.00 | 100.00 | STREPTOMYCIN | 844 | 1752 | + | NCBI | 011 | efaecium | 117 | 118 | Enterococcus faecium |
| vanX-A | NG\_048477.1 | D-Ala-D-Ala dipeptidase VanX-A | 100.00 | 100.00 | VANCOMYCIN | 4219 | 4827 | + | NCBI | 011 | efaecium | 117 | 94 | Enterococcus faecium |
| eat(A) | NG\_047762.1 | ABC-F type ribosomal protection protein Eat(A) | 100.00 | 100.00 | PLEUROMUTILIN | 60123 | 61625 | - | NCBI | 011 | efaecium | 117 | 6 | Enterococcus faecium |
| aph(3')-IIIa | NG\_047418.1 | aminoglycoside O-phosphotransferase APH(3')-IIIa | 100.00 | 100.00 | AMIKACIN;KANAMYCIN | 534 | 1328 | + | NCBI | 011 | efaecium | 117 | 82 | Enterococcus faecium |
| erm(B) | NG\_047804.1 | 23S rRNA (adenine(2058)-N(6))-methyltransferase Erm(B) | 99.86 | 100.00 | MACROLIDE | 5095 | 5832 | - | NCBI | 011 | efaecium | 117 | 82 | Enterococcus faecium |
| vanR-A | NG\_048399.1 | VanA-type vancomycin resistance DNA-binding response regulator VanR | 100.00 | 100.00 | VANCOMYCIN | 179 | 874 | + | NCBI | 011 | efaecium | 117 | 94 | Enterococcus faecium |
| vanA | NG\_048323.1 | D-alanine--(R)-lactate ligase VanA | 100.00 | 100.00 | VANCOMYCIN | 3182 | 4213 | + | NCBI | 011 | efaecium | 117 | 94 | Enterococcus faecium |
| msr(C) | NG\_048003.1 | ABC-F type ribosomal protection protein Msr(C) | 98.92 | 100.00 | MACROLIDE | 80916 | 82394 | - | NCBI | 011 | efaecium | 117 | 2 | Enterococcus faecium |
| vanH-A | NG\_048372.1 | D-lactate dehydrogenase VanH-A | 100.00 | 100.00 | VANCOMYCIN | 2221 | 3189 | + | NCBI | 011 | efaecium | 117 | 94 | Enterococcus faecium |
| aph(3')-IIIa | NG\_047418.1 | aminoglycoside O-phosphotransferase APH(3')-IIIa | 100.00 | 100.00 | AMIKACIN;KANAMYCIN | 4994 | 5788 | - | NCBI | 012 | efaecium | 117 | 53 | Enterococcus faecium |
| ant(6)-Ia | NG\_047393.1 | aminoglycoside nucleotidyltransferase ANT(6)-Ia | 100.00 | 100.00 | STREPTOMYCIN | 6420 | 7328 | - | NCBI | 012 | efaecium | 117 | 53 | Enterococcus faecium |
| vanY-B | NG\_048519.1 | D-Ala-D-Ala carboxypeptidase VanY-B | 99.88 | 100.00 | VANCOMYCIN | 281 | 1087 | + | NCBI | 012 | efaecium | 117 | 93 | Enterococcus faecium |
| vanW-B | NG\_048472.1 | glycopeptide resistance accessory protein VanW-B | 99.76 | 100.00 | VANCOMYCIN | 1105 | 1932 | + | NCBI | 012 | efaecium | 117 | 93 | Enterococcus faecium |
| vanH-B | NG\_048379.1 | D-lactate dehydrogenase VanH-B | 100.00 | 100.00 | VANCOMYCIN | 1929 | 2900 | + | NCBI | 012 | efaecium | 117 | 93 | Enterococcus faecium |
| vanB | NG\_048341.1 | D-alanine--(R)-lactate ligase VanB | 99.90 | 100.00 | VANCOMYCIN | 2893 | 3921 | + | NCBI | 012 | efaecium | 117 | 93 | Enterococcus faecium |
| vanX-B | NG\_048483.1 | D-Ala-D-Ala dipeptidase VanX-B | 100.00 | 100.00 | VANCOMYCIN | 3927 | 4535 | + | NCBI | 012 | efaecium | 117 | 93 | Enterococcus faecium |
| aph(2'')-Ih | NG\_047408.1 | aminoglycoside O-phosphotransferase APH(2'')-Ih | 82.43 | 89.15 | AMIKACIN;GENTAMICIN;KANAMYCIN;TOBRAMYCIN | 287 | 1083 | - | NCBI | 012 | efaecium | 117 | 114 | Enterococcus faecium |
| erm(B) | NG\_047804.1 | 23S rRNA (adenine(2058)-N(6))-methyltransferase Erm(B) | 99.86 | 100.00 | MACROLIDE | 490 | 1227 | + | NCBI | 012 | efaecium | 117 | 53 | Enterococcus faecium |
| vanR-B | NG\_048410.1 | VanB-type vancomycin resistance response regulator transcription factor VanR | 99.70 | 100.00 | VANCOMYCIN | 1495 | 2157 | - | NCBI | 012 | efaecium | 117 | 27 | Enterococcus faecium |
| vanS-B | NG\_048439.1 | VanB-type vancomycin resistance histidine kinase VanS | 99.85 | 100.00 | VANCOMYCIN | 152 | 1495 | - | NCBI | 012 | efaecium | 117 | 27 | Enterococcus faecium |
| msr(C) | NG\_048003.1 | ABC-F type ribosomal protection protein Msr(C) | 98.92 | 100.00 | MACROLIDE | 27558 | 29036 | - | NCBI | 012 | efaecium | 117 | 11 | Enterococcus faecium |
| dfrG | NG\_047756.1 | trimethoprim-resistant dihydrofolate reductase DfrG | 100.00 | 100.00 | TRIMETHOPRIM | 4157 | 4654 | - | NCBI | 012 | efaecium | 117 | 6 | Enterococcus faecium |
| eat(A) | NG\_047762.1 | ABC-F type ribosomal protection protein Eat(A) | 100.00 | 100.00 | PLEUROMUTILIN | 60123 | 61625 | - | NCBI | 012 | efaecium | 117 | 4 | Enterococcus faecium |
| aacA-ENT1 | NG\_052371.1 | aminoglycoside 6'-N-acetyltransferase | 100.00 | 100.00 | AMINOGLYCOSIDE | 62240 | 62788 | - | NCBI | 012 | efaecium | 117 | 2 | Enterococcus faecium |
| sat4 | NG\_048072.1 | streptothricin N-acetyltransferase Sat4 | 100.00 | 100.00 | STREPTOTHRICIN | 5881 | 6423 | - | NCBI | 012 | efaecium | 117 | 53 | Enterococcus faecium |
| blaADC-30 | NG\_048652.1 | class C extended-spectrum beta-lactamase ADC-30 | 100.00 | 100.00 | CEPHALOSPORIN | 298065 | 299216 | - | NCBI | 013 | abaumannii\_2 | 492 | 2 | Acinetobacter baumannii |
| aph(3'')-Ib | NG\_056002.2 | aminoglycoside O-phosphotransferase APH(3'')-Ib | 99.88 | 98.31 | STREPTOMYCIN | 15877 | 16690 | - | NCBI | 013 | abaumannii\_2 | 492 | 18 | Acinetobacter baumannii |
| sul2 | NG\_051852.1 | sulfonamide-resistant dihydropteroate synthase Sul2 | 100.00 | 100.00 | SULFONAMIDE | 146 | 961 | + | NCBI | 013 | abaumannii\_2 | 492 | 48 | Acinetobacter baumannii |
| blaOXA-72 | NG\_049813.1 | OXA-24 family carbapenem-hydrolyzing class D beta-lactamase OXA-72 | 100.00 | 100.00 | CARBAPENEM | 8193 | 9020 | - | NCBI | 013 | abaumannii\_2 | 492 | 39 | Acinetobacter baumannii |
| ant(3'')-IIa | NG\_054646.1 | aminoglycoside nucleotidyltransferase ANT(3'')-IIa | 99.24 | 100.00 | SPECTINOMYCIN;STREPTOMYCIN | 8290 | 9078 | + | NCBI | 013 | abaumannii\_2 | 492 | 28 | Acinetobacter baumannii |
| aph(6)-Id | NG\_047464.1 | aminoglycoside O-phosphotransferase APH(6)-Id | 100.00 | 100.00 | STREPTOMYCIN | 15041 | 15877 | - | NCBI | 013 | abaumannii\_2 | 492 | 18 | Acinetobacter baumannii |
| tet(B) | NG\_048161.1 | tetracycline efflux MFS transporter Tet(B) | 100.00 | 99.50 | TETRACYCLINE | 10469 | 11668 | - | NCBI | 013 | abaumannii\_2 | 492 | 18 | Acinetobacter baumannii |
| blaOXA-66 | NG\_049806.1 | OXA-51 family carbapenem-hydrolyzing class D beta-lactamase OXA-66 | 100.00 | 100.00 | CARBAPENEM | 122130 | 122954 | + | NCBI | 013 | abaumannii\_2 | 492 | 5 | Acinetobacter baumannii |
| blaCTX-M-15 | NG\_048935.1 | class A extended-spectrum beta-lactamase CTX-M-15 | 100.00 | 100.00 | CEPHALOSPORIN | 3316 | 4191 | - | NCBI | 014 | ecoli\_achtman\_4 | 90 | 5 | Escherichia coli |
| sul2 | NG\_051852.1 | sulfonamide-resistant dihydropteroate synthase Sul2 | 100.00 | 100.00 | SULFONAMIDE | 21212 | 22027 | - | NCBI | 014 | ecoli\_achtman\_4 | 90 | 37 | Escherichia coli |
| tet(A) | NG\_048154.1 | tetracycline efflux MFS transporter Tet(A) | 99.92 | 100.00 | TETRACYCLINE | 3137 | 4336 | + | NCBI | 014 | ecoli\_achtman\_4 | 90 | 18 | Escherichia coli |
| tet(M) | NG\_048237.1 | tetracycline resistance ribosomal protection protein Tet(M) | 97.66 | 100.00 | TETRACYCLINE | 971 | 2890 | + | NCBI | 014 | ecoli\_achtman\_4 | 90 | 37 | Escherichia coli |
| aadA1 | NG\_052266.1 | ANT(3'')-Ia family aminoglycoside nucleotidyltransferase AadA1 | 99.87 | 100.00 | STREPTOMYCIN | 4818 | 5609 | - | NCBI | 014 | ecoli\_achtman\_4 | 90 | 37 | Escherichia coli |
| cmlA1 | NG\_047648.1 | chloramphenicol efflux MFS transporter CmlA1 | 99.92 | 100.00 | CHLORAMPHENICOL | 5702 | 6961 | - | NCBI | 014 | ecoli\_achtman\_4 | 90 | 37 | Escherichia coli |
| aadA2 | NG\_047343.1 | ANT(3'')-Ia family aminoglycoside nucleotidyltransferase AadA2 | 100.00 | 100.00 | STREPTOMYCIN | 7223 | 8014 | - | NCBI | 014 | ecoli\_achtman\_4 | 90 | 37 | Escherichia coli |
| dfrA12 | NG\_047689.1 | trimethoprim-resistant dihydrofolate reductase DfrA12 | 100.00 | 100.00 | TRIMETHOPRIM | 8422 | 8919 | - | NCBI | 014 | ecoli\_achtman\_4 | 90 | 37 | Escherichia coli |
| floR | NG\_047862.1 | chloramphenicol/florfenicol efflux MFS transporter FloR | 99.83 | 100.00 | CHLORAMPHENICOL;FLORFENICOL | 17495 | 18709 | + | NCBI | 014 | ecoli\_achtman\_4 | 90 | 37 | Escherichia coli |
| blaEC-13 | NG\_049079.1 | class C extended-spectrum beta-lactamase EC-13 | 99.91 | 100.00 | CEPHALOSPORIN | 172782 | 173915 | - | NCBI | 014 | ecoli\_achtman\_4 | 90 | 7 | Escherichia coli |
| fosA-354827590 | NG\_047883.1 | FosA family fosfomycin resistance glutathione transferase | 99.02 | 100.00 | FOSFOMYCIN | 234566 | 234973 | + | NCBI | 015 | paeruginosa | - | 11 | Pseudomonas aeruginosa |
| crpP | NG\_062203.1 | ciprofloxacin resistance protein CrpP | 94.95 | 100.00 | FLUOROQUINOLONE | 3683 | 3880 | + | NCBI | 015 | paeruginosa | - | 13 | Pseudomonas aeruginosa |
| aph(3')-IIb | NG\_047424.1 | aminoglycoside O-phosphotransferase APH(3')-IIb | 98.89 | 100.00 | KANAMYCIN | 106847 | 107653 | + | NCBI | 015 | paeruginosa | - | 16 | Pseudomonas aeruginosa |
| blaPDC-374 | NG\_065926.1 | class C beta-lactamase PDC-374 | 99.02 | 100.00 | CEPHALOSPORIN | 119980 | 121202 | - | NCBI | 015 | paeruginosa | - | 16 | Pseudomonas aeruginosa |
| catB7 | NG\_047614.1 | type B-4 chloramphenicol O-acetyltransferase CatB7 | 99.37 | 100.00 | CHLORAMPHENICOL | 265160 | 265798 | + | NCBI | 015 | paeruginosa | - | 7 | Pseudomonas aeruginosa |
| blaOXA-486 | NG\_050612.1 | OXA-50 family oxacillin-hydrolyzing class D beta-lactamase OXA-486 | 99.75 | 100.00 | BETA-LACTAM | 109585 | 110373 | - | NCBI | 015 | paeruginosa | - | 9 | Pseudomonas aeruginosa |
| blaOXA-905 | NG\_068024.1 | OXA-50 family oxacillin-hydrolyzing class D beta-lactamase OXA-905 | 100.00 | 100.00 | BETA-LACTAM | 145930 | 146718 | - | NCBI | 016 | paeruginosa | 395 | 8 | Pseudomonas aeruginosa |
| fosA-354827590 | NG\_047883.1 | FosA family fosfomycin resistance glutathione transferase | 99.51 | 100.00 | FOSFOMYCIN | 119258 | 119665 | - | NCBI | 016 | paeruginosa | 395 | 11 | Pseudomonas aeruginosa |
| blaPDC-374 | NG\_065926.1 | class C beta-lactamase PDC-374 | 98.94 | 100.00 | CEPHALOSPORIN | 119734 | 120956 | - | NCBI | 016 | paeruginosa | 395 | 20 | Pseudomonas aeruginosa |
| catB7 | NG\_047614.1 | type B-4 chloramphenicol O-acetyltransferase CatB7 | 99.53 | 100.00 | CHLORAMPHENICOL | 353731 | 354369 | + | NCBI | 016 | paeruginosa | 395 | 3 | Pseudomonas aeruginosa |
| aph(3')-IIb | NG\_047424.1 | aminoglycoside O-phosphotransferase APH(3')-IIb | 99.38 | 100.00 | KANAMYCIN | 106601 | 107407 | + | NCBI | 016 | paeruginosa | 395 | 20 | Pseudomonas aeruginosa |
| aph(6)-Id | NG\_047464.1 | aminoglycoside O-phosphotransferase APH(6)-Id | 100.00 | 100.00 | STREPTOMYCIN | 3935 | 4771 | - | NCBI | 017 | klebsiella | - | 30 | Klebsiella pneumoniae |
| sul2 | NG\_051852.1 | sulfonamide-resistant dihydropteroate synthase Sul2 | 100.00 | 100.00 | SULFONAMIDE | 5635 | 6450 | - | NCBI | 017 | klebsiella | - | 30 | Klebsiella pneumoniae |
| blaOXA-1 | NG\_049392.1 | oxacillin-hydrolyzing class D beta-lactamase OXA-1 | 99.88 | 100.00 | CEPHALOSPORIN | 904 | 1734 | + | NCBI | 017 | klebsiella | - | 40 | Klebsiella pneumoniae |
| aac(3)-IIe | NG\_047244.1 | aminoglycoside N-acetyltransferase AAC(3)-IIe | 99.77 | 100.00 | GENTAMICIN | 1823 | 2683 | - | NCBI | 017 | klebsiella | - | 38 | Klebsiella pneumoniae |
| dfrA14 | NG\_056035.1 | trimethoprim-resistant dihydrofolate reductase DfrA14 | 100.00 | 100.00 | TRIMETHOPRIM | 4729 | 5202 | - | NCBI | 017 | klebsiella | - | 31 | Klebsiella pneumoniae |
| blaSHV-145 | NG\_050019.1 | class A beta-lactamase SHV-145 | 99.88 | 100.00 | BETA-LACTAM | 490369 | 491229 | + | NCBI | 017 | klebsiella | - | 2 | Klebsiella pneumoniae |
| oqxB20 | NG\_050439.1 | multidrug efflux RND transporter permease subunit OqxB20 | 99.11 | 100.00 | PHENICOL;QUINOLONE | 402012 | 405164 | - | NCBI | 017 | klebsiella | - | 3 | Klebsiella pneumoniae |
| oqxA11 | NG\_050419.1 | multidrug efflux RND transporter periplasmic adaptor subunit OqxA11 | 99.66 | 100.00 | PHENICOL;QUINOLONE | 405188 | 406363 | - | NCBI | 017 | klebsiella | - | 3 | Klebsiella pneumoniae |
| blaCTX-M-15 | NG\_048935.1 | class A extended-spectrum beta-lactamase CTX-M-15 | 100.00 | 100.00 | CEPHALOSPORIN | 731 | 1606 | + | NCBI | 017 | klebsiella | - | 35 | Klebsiella pneumoniae |
| fosA\_gen | NG\_047882.1 | FosA family fosfomycin resistance glutathione transferase | 100.00 | 100.00 | FOSFOMYCIN | 22892 | 23311 | + | NCBI | 017 | klebsiella | - | 6 | Klebsiella pneumoniae |
| blaTEM-1 | NG\_050145.1 | class A broad-spectrum beta-lactamase TEM-1 | 100.00 | 100.00 | BETA-LACTAM | 2354 | 3214 | + | NCBI | 017 | klebsiella | - | 30 | Klebsiella pneumoniae |
| aph(3'')-Ib | NG\_056002.2 | aminoglycoside O-phosphotransferase APH(3'')-Ib | 100.00 | 100.00 | STREPTOMYCIN | 4771 | 5598 | - | NCBI | 017 | klebsiella | - | 30 | Klebsiella pneumoniae |
| aac(6')-Ib-D181Y | NG\_067946.1 | AAC(6')-Ib family aminoglycoside 6'-N-acetyltransferase | 99.82 | 100.00 | AMIKACIN;KANAMYCIN;TOBRAMYCIN | 219 | 773 | + | NCBI | 017 | klebsiella | - | 40 | Klebsiella pneumoniae |
| tet(A) | NG\_048154.1 | tetracycline efflux MFS transporter Tet(A) | 100.00 | 100.00 | TETRACYCLINE | 2716 | 3915 | + | NCBI | 018 | ecoli\_achtman\_4 | 131 | 44 | Escherichia coli |
| sul1 | NG\_048082.1 | sulfonamide-resistant dihydropteroate synthase Sul1 | 100.00 | 100.00 | SULFONAMIDE | 3554 | 4393 | + | NCBI | 018 | ecoli\_achtman\_4 | 131 | 53 | Escherichia coli |
| aadA5 | NG\_047357.1 | ANT(3'')-Ia family aminoglycoside nucleotidyltransferase AadA5 | 100.00 | 100.00 | STREPTOMYCIN | 2219 | 3007 | + | NCBI | 018 | ecoli\_achtman\_4 | 131 | 53 | Escherichia coli |
| blaOXA-1 | NG\_049392.1 | oxacillin-hydrolyzing class D beta-lactamase OXA-1 | 100.00 | 100.00 | CEPHALOSPORIN | 904 | 1734 | + | NCBI | 018 | ecoli\_achtman\_4 | 131 | 73 | Escherichia coli |
| aac(6')-Ib-D181Y | NG\_067946.1 | AAC(6')-Ib family aminoglycoside 6'-N-acetyltransferase | 99.82 | 100.00 | AMIKACIN;KANAMYCIN;TOBRAMYCIN | 219 | 773 | + | NCBI | 018 | ecoli\_achtman\_4 | 131 | 73 | Escherichia coli |
| aac(3)-IIe | NG\_047244.1 | aminoglycoside N-acetyltransferase AAC(3)-IIe | 99.77 | 100.00 | GENTAMICIN | 171 | 1031 | + | NCBI | 018 | ecoli\_achtman\_4 | 131 | 70 | Escherichia coli |
| blaCTX-M-15 | NG\_048935.1 | class A extended-spectrum beta-lactamase CTX-M-15 | 100.00 | 100.00 | CEPHALOSPORIN | 2763 | 3638 | - | NCBI | 018 | ecoli\_achtman\_4 | 131 | 66 | Escherichia coli |
| mph(A) | NG\_047986.1 | Mph(A) family macrolide 2'-phosphotransferase | 99.67 | 100.00 | MACROLIDE | 9688 | 10609 | - | NCBI | 018 | ecoli\_achtman\_4 | 131 | 53 | Escherichia coli |
| blaEC-5 | NG\_049085.1 | cephalosporin-hydrolyzing class C beta-lactamase EC-5 | 99.47 | 100.00 | CEPHALOSPORIN | 131249 | 132382 | + | NCBI | 018 | ecoli\_achtman\_4 | 131 | 14 | Escherichia coli |
| dfrA17 | NG\_047710.1 | trimethoprim-resistant dihydrofolate reductase DfrA17 | 99.79 | 100.00 | TRIMETHOPRIM | 1615 | 2088 | + | NCBI | 018 | ecoli\_achtman\_4 | 131 | 53 | Escherichia coli |
| vanY-A | NG\_048516.1 | D-Ala-D-Ala carboxypeptidase VanY-A | 100.00 | 100.00 | VANCOMYCIN | 6293 | 7204 | - | NCBI | 019 | efaecium | 203 | 138 | Enterococcus faecium |
| vanR-A | NG\_048399.1 | VanA-type vancomycin resistance DNA-binding response regulator VanR | 100.00 | 100.00 | VANCOMYCIN | 79 | 774 | + | NCBI | 019 | efaecium | 203 | 164 | Enterococcus faecium |
| vanS-A | NG\_048425.1 | VanA-type vancomycin resistance histidine kinase VanS | 100.00 | 100.00 | VANCOMYCIN | 752 | 1906 | + | NCBI | 019 | efaecium | 203 | 164 | Enterococcus faecium |
| vanH-A | NG\_048372.1 | D-lactate dehydrogenase VanH-A | 100.00 | 100.00 | VANCOMYCIN | 2121 | 3089 | + | NCBI | 019 | efaecium | 203 | 164 | Enterococcus faecium |
| vanA | NG\_048323.1 | D-alanine--(R)-lactate ligase VanA | 100.00 | 100.00 | VANCOMYCIN | 3082 | 4113 | + | NCBI | 019 | efaecium | 203 | 164 | Enterococcus faecium |
| catA7 | NG\_047568.1 | type A-7 chloramphenicol O-acetyltransferase | 100.00 | 100.00 | CHLORAMPHENICOL | 2544 | 3191 | + | NCBI | 019 | efaecium | 203 | 185 | Enterococcus faecium |
| aph(2'')-Ih | NG\_047408.1 | aminoglycoside O-phosphotransferase APH(2'')-Ih | 82.99 | 82.21 | AMIKACIN;GENTAMICIN;KANAMYCIN;TOBRAMYCIN | 2242 | 2976 | - | NCBI | 019 | efaecium | 203 | 186 | Enterococcus faecium |
| ant(6)-Ia | NG\_047393.1 | aminoglycoside nucleotidyltransferase ANT(6)-Ia | 100.00 | 100.00 | STREPTOMYCIN | 538 | 1446 | - | NCBI | 019 | efaecium | 203 | 188 | Enterococcus faecium |
| erm(B) | NG\_047801.1 | 23S rRNA (adenine(2058)-N(6))-methyltransferase Erm(B) | 99.60 | 99.87 | MACROLIDE | 466 | 1211 | - | NCBI | 019 | efaecium | 203 | 223 | Enterococcus faecium |
| aph(2'')-Ih | NG\_047408.1 | aminoglycoside O-phosphotransferase APH(2'')-Ih | 82.43 | 89.15 | AMIKACIN;GENTAMICIN;KANAMYCIN;TOBRAMYCIN | 439 | 1235 | - | NCBI | 019 | efaecium | 203 | 186 | Enterococcus faecium |
| dfrF | NG\_047755.1 | trimethoprim-resistant dihydrofolate reductase DfrF | 99.60 | 100.00 | TRIMETHOPRIM | 6873 | 7368 | + | NCBI | 019 | efaecium | 203 | 120 | Enterococcus faecium |
| msr(C) | NG\_048003.1 | ABC-F type ribosomal protection protein Msr(C) | 98.92 | 100.00 | MACROLIDE | 2560 | 4038 | - | NCBI | 019 | efaecium | 203 | 69 | Enterococcus faecium |
| eat(A) | NG\_047762.1 | ABC-F type ribosomal protection protein Eat(A) | 100.00 | 100.00 | PLEUROMUTILIN | 5883 | 7385 | + | NCBI | 019 | efaecium | 203 | 37 | Enterococcus faecium |
| aacA-ENT1 | NG\_052371.1 | aminoglycoside 6'-N-acetyltransferase | 100.00 | 100.00 | AMINOGLYCOSIDE | 31465 | 32013 | + | NCBI | 019 | efaecium | 203 | 14 | Enterococcus faecium |
| tet(M) | NG\_048213.1 | tetracycline resistance ribosomal protection protein Tet(M) | 99.79 | 100.00 | TETRACYCLINE | 19076 | 20995 | + | NCBI | 019 | efaecium | 203 | 8 | Enterococcus faecium |
| vanZ-A | NG\_048534.1 | glycopeptide resistance protein VanZ-A | 100.00 | 100.00 | VANCOMYCIN | 5655 | 6140 | - | NCBI | 019 | efaecium | 203 | 138 | Enterococcus faecium |
| catA1 | NG\_047582.1 | type A-1 chloramphenicol O-acetyltransferase | 99.85 | 100.00 | CHLORAMPHENICOL | 4106 | 4765 | + | NCBI | 020 | abaumannii\_2 | 636 | 54 | Acinetobacter baumannii |
| aadA1 | NG\_047324.1 | ANT(3'')-Ia family aminoglycoside nucleotidyltransferase AadA1 | 100.00 | 100.00 | STREPTOMYCIN | 247 | 1038 | - | NCBI | 020 | abaumannii\_2 | 636 | 64 | Acinetobacter baumannii |
| aph(3')-Ia | NG\_047431.1 | aminoglycoside O-phosphotransferase APH(3')-Ia | 100.00 | 100.00 | KANAMYCIN | 674 | 1489 | - | NCBI | 020 | abaumannii\_2 | 636 | 84 | Acinetobacter baumannii |
| aac(3)-Ia | NG\_047234.1 | aminoglycoside N-acetyltransferase AAC(3)-Ia | 100.00 | 100.00 | GENTAMICIN | 2082 | 2546 | - | NCBI | 020 | abaumannii\_2 | 636 | 64 | Acinetobacter baumannii |
| blaOXA-72 | NG\_049813.1 | OXA-24 family carbapenem-hydrolyzing class D beta-lactamase OXA-72 | 100.00 | 100.00 | CARBAPENEM | 10786 | 11613 | + | NCBI | 020 | abaumannii\_2 | 636 | 49 | Acinetobacter baumannii |
| blaOXA-66 | NG\_049806.1 | OXA-51 family carbapenem-hydrolyzing class D beta-lactamase OXA-66 | 100.00 | 100.00 | CARBAPENEM | 19004 | 19828 | - | NCBI | 020 | abaumannii\_2 | 636 | 36 | Acinetobacter baumannii |
| blaADC-74 | NG\_048679.1 | class C extended-spectrum beta-lactamase ADC-74 | 100.00 | 100.00 | CEPHALOSPORIN | 136 | 1287 | + | NCBI | 020 | abaumannii\_2 | 636 | 77 | Acinetobacter baumannii |
| aph(3')-VIa | NG\_047448.1 | aminoglycoside O-phosphotransferase APH(3')-VIa | 99.87 | 100.00 | AMIKACIN;KANAMYCIN | 150 | 929 | - | NCBI | 020 | abaumannii\_2 | 636 | 91 | Acinetobacter baumannii |
| sul1 | NG\_048082.1 | sulfonamide-resistant dihydropteroate synthase Sul1 | 100.00 | 100.00 | SULFONAMIDE | 699 | 1538 | - | NCBI | 020 | abaumannii\_2 | 636 | 79 | Acinetobacter baumannii |
| ant(3'')-IIa | NG\_054646.1 | aminoglycoside nucleotidyltransferase ANT(3'')-IIa | 100.00 | 100.00 | SPECTINOMYCIN;STREPTOMYCIN | 34820 | 35608 | - | NCBI | 020 | abaumannii\_2 | 636 | 6 | Acinetobacter baumannii |
| blaADC-106 | NG\_051445.1 | class C beta-lactamase ADC-106 | 98.18 | 100.00 | CEPHALOSPORIN | 366666 | 367817 | - | NCBI | 021 | abaumannii\_2 | 620 | 2 | Acinetobacter baumannii |
| blaOXA-65 | NG\_049805.1 | OXA-51 family carbapenem-hydrolyzing class D beta-lactamase OXA-65 | 99.76 | 100.00 | CARBAPENEM | 1014953 | 1015777 | - | NCBI | 021 | abaumannii\_2 | 620 | 1 | Acinetobacter baumannii |
| ant(3'')-IIa | NG\_054652.1 | aminoglycoside nucleotidyltransferase ANT(3'')-IIa | 99.49 | 99.87 | SPECTINOMYCIN;STREPTOMYCIN | 131460 | 132247 | + | NCBI | 021 | abaumannii\_2 | 620 | 6 | Acinetobacter baumannii |
| sat4 | NG\_048072.1 | streptothricin N-acetyltransferase Sat4 | 100.00 | 100.00 | STREPTOTHRICIN | 2617 | 3159 | + | NCBI | 022 | saureus | 1 | 13 | Staphylococcus aureus |
| mecA | NG\_047940.1 | PBP2a family beta-lactam-resistant peptidoglycan transpeptidase MecA | 99.95 | 100.00 | METHICILLIN | 321315 | 323321 | + | NCBI | 022 | saureus | 1 | 3 | Staphylococcus aureus |
| tet(38) | NG\_055984.1 | tetracycline efflux MFS transporter Tet(38) | 100.00 | 100.00 | TETRACYCLINE | 232657 | 234009 | - | NCBI | 022 | saureus | 1 | 3 | Staphylococcus aureus |
| aph(3')-IIIa | NG\_047418.1 | aminoglycoside O-phosphotransferase APH(3')-IIIa | 100.00 | 100.00 | AMIKACIN;KANAMYCIN | 3252 | 4046 | + | NCBI | 022 | saureus | 1 | 13 | Staphylococcus aureus |
| blaR1 | NG\_051774.1 | beta-lactam sensor/signal transducer BlaR1 | 97.27 | 100.00 | BETA-LACTAM | 2024 | 3781 | - | NCBI | 022 | saureus | 1 | 12 | Staphylococcus aureus |
| erm(C) | NG\_047806.1 | 23S rRNA (adenine(2058)-N(6))-methyltransferase Erm(C) | 99.86 | 100.00 | MACROLIDE | 452 | 1186 | - | NCBI | 022 | saureus | 1 | 16 | Staphylococcus aureus |
| tet(K) | NG\_048200.1 | tetracycline efflux MFS transporter Tet(K) | 99.86 | 100.00 | TETRACYCLINE | 1237 | 2616 | + | NCBI | 022 | saureus | 1 | 14 | Staphylococcus aureus |
| blaZ | NG\_047534.1 | penicillin-hydrolyzing class A beta-lactamase BlaZ | 100.00 | 100.00 | BETA-LACTAM | 3888 | 4733 | + | NCBI | 022 | saureus | 1 | 12 | Staphylococcus aureus |
| ant(6)-Ia | NG\_047393.1 | aminoglycoside nucleotidyltransferase ANT(6)-Ia | 100.00 | 100.00 | STREPTOMYCIN | 1712 | 2620 | + | NCBI | 022 | saureus | 1 | 13 | Staphylococcus aureus |
| blaI\_of\_Z | NG\_047499.1 | penicillinase repressor BlaI | 98.42 | 100.00 | BETA-LACTAM | 1654 | 2034 | - | NCBI | 022 | saureus | 1 | 12 | Staphylococcus aureus |
| oqxA5 | NG\_050423.1 | multidrug efflux RND transporter periplasmic adaptor subunit OqxA5 | 99.41 | 100.00 | PHENICOL;QUINOLONE | 37141 | 38316 | + | NCBI | 023 | klebsiella | 307 | 2 | Klebsiella pneumoniae |
| oqxB19 | NG\_050437.1 | multidrug efflux RND transporter permease subunit OqxB19 | 99.40 | 100.00 | PHENICOL;QUINOLONE | 38340 | 41492 | + | NCBI | 023 | klebsiella | 307 | 2 | Klebsiella pneumoniae |
| aac(3)-IIe | NG\_047244.1 | aminoglycoside N-acetyltransferase AAC(3)-IIe | 99.77 | 100.00 | GENTAMICIN | 1824 | 2684 | - | NCBI | 023 | klebsiella | 307 | 38 | Klebsiella pneumoniae |
| blaSHV-106 | NG\_049996.1 | class A extended-spectrum beta-lactamase SHV-106 | 99.88 | 100.00 | CEPHALOSPORIN | 225430 | 226290 | - | NCBI | 023 | klebsiella | 307 | 5 | Klebsiella pneumoniae |
| fosA6 | NG\_051497.1 | fosfomycin resistance glutathione transferase FosA6 | 99.76 | 100.00 | FOSFOMYCIN | 22892 | 23311 | + | NCBI | 023 | klebsiella | 307 | 15 | Klebsiella pneumoniae |
| sul2 | NG\_051852.1 | sulfonamide-resistant dihydropteroate synthase Sul2 | 100.00 | 100.00 | SULFONAMIDE | 23689 | 24504 | + | NCBI | 023 | klebsiella | 307 | 29 | Klebsiella pneumoniae |
| aph(3'')-Ib | NG\_056002.2 | aminoglycoside O-phosphotransferase APH(3'')-Ib | 100.00 | 100.00 | STREPTOMYCIN | 24541 | 25368 | + | NCBI | 023 | klebsiella | 307 | 29 | Klebsiella pneumoniae |
| aph(6)-Id | NG\_047464.1 | aminoglycoside O-phosphotransferase APH(6)-Id | 100.00 | 100.00 | STREPTOMYCIN | 25368 | 26204 | + | NCBI | 023 | klebsiella | 307 | 29 | Klebsiella pneumoniae |
| blaTEM-1 | NG\_050145.1 | class A broad-spectrum beta-lactamase TEM-1 | 100.00 | 100.00 | BETA-LACTAM | 26925 | 27785 | - | NCBI | 023 | klebsiella | 307 | 29 | Klebsiella pneumoniae |
| blaCTX-M-15 | NG\_048935.1 | class A extended-spectrum beta-lactamase CTX-M-15 | 100.00 | 100.00 | CEPHALOSPORIN | 30607 | 31482 | + | NCBI | 023 | klebsiella | 307 | 29 | Klebsiella pneumoniae |
| qnrB1 | NG\_050469.1 | quinolone resistance pentapeptide repeat protein QnrB1 | 100.00 | 100.00 | QUINOLONE | 946 | 1590 | - | NCBI | 023 | klebsiella | 307 | 32 | Klebsiella pneumoniae |
| dfrA14 | NG\_056035.1 | trimethoprim-resistant dihydrofolate reductase DfrA14 | 100.00 | 100.00 | TRIMETHOPRIM | 4729 | 5202 | - | NCBI | 023 | klebsiella | 307 | 34 | Klebsiella pneumoniae |
| aac(6')-Ib-D181Y | NG\_067946.1 | AAC(6')-Ib family aminoglycoside 6'-N-acetyltransferase | 99.82 | 100.00 | AMIKACIN;KANAMYCIN;TOBRAMYCIN | 219 | 773 | + | NCBI | 023 | klebsiella | 307 | 39 | Klebsiella pneumoniae |
| blaOXA-1 | NG\_049392.1 | oxacillin-hydrolyzing class D beta-lactamase OXA-1 | 100.00 | 100.00 | CEPHALOSPORIN | 904 | 1734 | + | NCBI | 023 | klebsiella | 307 | 39 | Klebsiella pneumoniae |
| aph(6)-Id | NG\_047464.1 | aminoglycoside O-phosphotransferase APH(6)-Id | 100.00 | 100.00 | STREPTOMYCIN | 28375 | 29211 | + | NCBI | 024 | ecloacae | 136 | 23 | Enterobacter cloacae |
| aph(3'')-Ib | NG\_056002.2 | aminoglycoside O-phosphotransferase APH(3'')-Ib | 100.00 | 100.00 | STREPTOMYCIN | 27548 | 28375 | + | NCBI | 024 | ecloacae | 136 | 23 | Enterobacter cloacae |
| blaCMY-16 | NG\_048810.1 | class C beta-lactamase CMY-16 | 99.91 | 100.00 | CEPHALOSPORIN | 55711 | 56856 | - | NCBI | 024 | ecloacae | 136 | 17 | Enterobacter cloacae |
| blaACT-46 | NG\_050709.1 | cephalosporin-hydrolyzing class C beta-lactamase ACT-46 | 99.65 | 100.00 | CEPHALOSPORIN | 472699 | 473844 | + | NCBI | 024 | ecloacae | 136 | 2 | Enterobacter cloacae |
| qnrB1 | NG\_050469.1 | quinolone resistance pentapeptide repeat protein QnrB1 | 100.00 | 100.00 | QUINOLONE | 4531 | 5175 | + | NCBI | 024 | ecloacae | 136 | 29 | Enterobacter cloacae |
| oqxA9 | NG\_050427.1 | multidrug efflux RND transporter periplasmic adaptor subunit OqxA9 | 86.22 | 100.00 | PHENICOL;QUINOLONE | 773232 | 774407 | - | NCBI | 024 | ecloacae | 136 | 1 | Enterobacter cloacae |
| oqxB9 | NG\_050458.1 | multidrug efflux RND transporter permease subunit OqxB9 | 89.42 | 98.95 | PHENICOL;QUINOLONE | 770089 | 773208 | - | NCBI | 024 | ecloacae | 136 | 1 | Enterobacter cloacae |
| tet(A) | NG\_048153.1 | tetracycline efflux MFS transporter Tet(A) | 100.00 | 100.00 | TETRACYCLINE | 30547 | 31746 | + | NCBI | 024 | ecloacae | 136 | 23 | Enterobacter cloacae |
| fosA | NG\_050405.1 | fosfomycin resistance glutathione transferase FosA | 95.78 | 100.00 | FOSFOMYCIN | 317578 | 318003 | - | NCBI | 024 | ecloacae | 136 | 2 | Enterobacter cloacae |
| floR | NG\_047869.1 | chloramphenicol/florfenicol efflux MFS transporter FloR | 100.00 | 100.00 | CHLORAMPHENICOL;FLORFENICOL | 32346 | 33560 | - | NCBI | 024 | ecloacae | 136 | 23 | Enterobacter cloacae |
| sul2 | NG\_051852.1 | sulfonamide-resistant dihydropteroate synthase Sul2 | 100.00 | 100.00 | SULFONAMIDE | 26696 | 27511 | + | NCBI | 024 | ecloacae | 136 | 23 | Enterobacter cloacae |
| blaNDM-1 | NG\_049326.1 | subclass B1 metallo-beta-lactamase NDM-1 | 100.00 | 100.00 | CARBAPENEM | 304 | 1116 | - | NCBI | 024 | ecloacae | 136 | 30 | Enterobacter cloacae |
| aph(3')-VI | NG\_051730.1 | APH(3')-VI family aminoglycoside O-phosphotransferase | 100.00 | 100.00 | AMIKACIN;KANAMYCIN | 2394 | 3173 | - | NCBI | 024 | ecloacae | 136 | 30 | Enterobacter cloacae |
| aadA2 | NG\_047343.1 | ANT(3'')-Ia family aminoglycoside nucleotidyltransferase AadA2 | 100.00 | 100.00 | STREPTOMYCIN | 659 | 1450 | - | NCBI | 024 | ecloacae | 136 | 33 | Enterobacter cloacae |
| sul1 | NG\_048082.1 | sulfonamide-resistant dihydropteroate synthase Sul1 | 100.00 | 96.79 | SULFONAMIDE | 1 | 813 | + | NCBI | 024 | ecloacae | 136 | 43 | Enterobacter cloacae |
| dfrA12 | NG\_047689.1 | trimethoprim-resistant dihydrofolate reductase DfrA12 | 100.00 | 100.00 | TRIMETHOPRIM | 1858 | 2355 | - | NCBI | 024 | ecloacae | 136 | 33 | Enterobacter cloacae |
| blaCTX-M-15 | NG\_048935.1 | class A extended-spectrum beta-lactamase CTX-M-15 | 100.00 | 100.00 | CEPHALOSPORIN | 2765 | 3640 | - | NCBI | 024 | ecloacae | 136 | 34 | Enterobacter cloacae |
| aac(3)-IIe | NG\_047244.1 | aminoglycoside N-acetyltransferase AAC(3)-IIe | 99.77 | 100.00 | GENTAMICIN | 1824 | 2684 | - | NCBI | 024 | ecloacae | 136 | 36 | Enterobacter cloacae |
| blaOXA-48 | NG\_049762.1 | carbapenem-hydrolyzing class D beta-lactamase OXA-48 | 100.00 | 100.00 | CARBAPENEM | 1409 | 2206 | - | NCBI | 024 | ecloacae | 136 | 39 | Enterobacter cloacae |
| aac(6')-Ib-D181Y | NG\_067946.1 | AAC(6')-Ib family aminoglycoside 6'-N-acetyltransferase | 99.82 | 100.00 | AMIKACIN;KANAMYCIN;TOBRAMYCIN | 1666 | 2220 | - | NCBI | 024 | ecloacae | 136 | 38 | Enterobacter cloacae |
| blaOXA-1 | NG\_049392.1 | oxacillin-hydrolyzing class D beta-lactamase OXA-1 | 100.00 | 100.00 | CEPHALOSPORIN | 705 | 1535 | - | NCBI | 024 | ecloacae | 136 | 38 | Enterobacter cloacae |
| qnrB19 | NG\_050479.1 | quinolone resistance pentapeptide repeat protein QnrB19 | 100.00 | 100.00 | QUINOLONE | 300 | 944 | + | NCBI | 025 | ecoli\_achtman\_4 | 167 | 319 | Escherichia coli |
| blaOXA-48 | NG\_049762.1 | carbapenem-hydrolyzing class D beta-lactamase OXA-48 | 100.00 | 100.00 | CARBAPENEM | 1564 | 2361 | - | NCBI | 025 | ecoli\_achtman\_4 | 167 | 295 | Escherichia coli |
| aph(6)-Id | NG\_047464.1 | aminoglycoside O-phosphotransferase APH(6)-Id | 100.00 | 100.00 | STREPTOMYCIN | 3568 | 4404 | + | NCBI | 025 | ecoli\_achtman\_4 | 167 | 253 | Escherichia coli |
| tet(A) | NG\_048154.1 | tetracycline efflux MFS transporter Tet(A) | 99.92 | 100.00 | TETRACYCLINE | 1096 | 2295 | - | NCBI | 025 | ecoli\_achtman\_4 | 167 | 277 | Escherichia coli |
| sul2 | NG\_048118.1 | sulfonamide-resistant dihydropteroate synthase Sul2 | 99.88 | 100.00 | SULFONAMIDE | 1889 | 2704 | + | NCBI | 025 | ecoli\_achtman\_4 | 167 | 253 | Escherichia coli |
| aph(3'')-Ib | NG\_056002.2 | aminoglycoside O-phosphotransferase APH(3'')-Ib | 100.00 | 100.00 | STREPTOMYCIN | 2741 | 3568 | + | NCBI | 025 | ecoli\_achtman\_4 | 167 | 253 | Escherichia coli |
| aadA5 | NG\_047357.1 | ANT(3'')-Ia family aminoglycoside nucleotidyltransferase AadA5 | 100.00 | 100.00 | STREPTOMYCIN | 13497 | 14285 | + | NCBI | 025 | ecoli\_achtman\_4 | 167 | 129 | Escherichia coli |
| dfrA17 | NG\_047710.1 | trimethoprim-resistant dihydrofolate reductase DfrA17 | 99.79 | 100.00 | TRIMETHOPRIM | 12893 | 13366 | + | NCBI | 025 | ecoli\_achtman\_4 | 167 | 129 | Escherichia coli |
| tet(B) | NG\_048163.1 | tetracycline efflux MFS transporter Tet(B) | 100.00 | 100.00 | TETRACYCLINE | 4307 | 5512 | - | NCBI | 025 | ecoli\_achtman\_4 | 167 | 129 | Escherichia coli |
| blaTEM-1 | NG\_050145.1 | class A broad-spectrum beta-lactamase TEM-1 | 100.00 | 100.00 | BETA-LACTAM | 1401 | 2261 | + | NCBI | 025 | ecoli\_achtman\_4 | 167 | 129 | Escherichia coli |
| blaEC-15 | NG\_049081.1 | class C extended-spectrum beta-lactamase EC-15 | 98.50 | 100.00 | CEPHALOSPORIN | 3241 | 4374 | - | NCBI | 025 | ecoli\_achtman\_4 | 167 | 92 | Escherichia coli |
| blaADC-167 | NG\_056109.1 | class C beta-lactamase ADC-167 | 99.05 | 100.00 | CEPHALOSPORIN | 1227527 | 1228678 | + | NCBI | 026 | abaumannii\_2 | 155 | 1 | Acinetobacter baumannii |
| blaOXA-126 | NG\_049425.1 | OXA-51 family carbapenem-hydrolyzing class D beta-lactamase OXA-126 | 100.00 | 100.00 | CARBAPENEM | 287361 | 288185 | - | NCBI | 026 | abaumannii\_2 | 155 | 1 | Acinetobacter baumannii |
| oqxA9 | NG\_050427.1 | multidrug efflux RND transporter periplasmic adaptor subunit OqxA9 | 86.82 | 100.00 | PHENICOL;QUINOLONE | 285683 | 286858 | + | NCBI | 027 | ecloacae | - | 1 | Enterobacter cloacae |
| oqxB9 | NG\_050458.1 | multidrug efflux RND transporter permease subunit OqxB9 | 89.79 | 100.00 | PHENICOL;QUINOLONE | 286882 | 290034 | + | NCBI | 027 | ecloacae | - | 1 | Enterobacter cloacae |
| fosA2 | NG\_050406.1 | fosfomycin resistance glutathione transferase FosA2 | 98.83 | 100.00 | FOSFOMYCIN | 309303 | 309728 | - | NCBI | 027 | ecloacae | - | 3 | Enterobacter cloacae |
| blaACT-12 | NG\_048599.1 | cephalosporin-hydrolyzing class C beta-lactamase ACT-12 | 98.60 | 100.00 | CEPHALOSPORIN | 486215 | 487360 | + | NCBI | 027 | ecloacae | - | 3 | Enterobacter cloacae |
| oqxA9 | NG\_050427.1 | multidrug efflux RND transporter periplasmic adaptor subunit OqxA9 | 86.91 | 100.00 | PHENICOL;QUINOLONE | 10512 | 11687 | + | NCBI | 028 | ecloacae | - | 57 | Enterobacter cloacae |
| oqxB9 | NG\_050458.1 | multidrug efflux RND transporter permease subunit OqxB9 | 89.10 | 98.95 | PHENICOL;QUINOLONE | 11711 | 14830 | + | NCBI | 028 | ecloacae | - | 57 | Enterobacter cloacae |
| fosA | NG\_050405.1 | fosfomycin resistance glutathione transferase FosA | 96.48 | 100.00 | FOSFOMYCIN | 24937 | 25362 | - | NCBI | 028 | ecloacae | - | 13 | Enterobacter cloacae |
| blaACT-17 | NG\_048603.1 | cephalosporin-hydrolyzing class C beta-lactamase ACT-17 | 99.65 | 100.00 | CEPHALOSPORIN | 13670 | 14815 | + | NCBI | 028 | ecloacae | - | 50 | Enterobacter cloacae |
| vanZ-A | NG\_048534.1 | glycopeptide resistance protein VanZ-A | 100.00 | 100.00 | VANCOMYCIN | 9808 | 10293 | - | NCBI | 029 | efaecium | 203 | 64 | Enterococcus faecium |
| aph(2'')-Ih | NG\_047408.1 | aminoglycoside O-phosphotransferase APH(2'')-Ih | 82.43 | 89.15 | AMIKACIN;GENTAMICIN;KANAMYCIN;TOBRAMYCIN | 2593 | 3389 | + | NCBI | 029 | efaecium | 203 | 103 | Enterococcus faecium |
| catA7 | NG\_047568.1 | type A-7 chloramphenicol O-acetyltransferase | 100.00 | 100.00 | CHLORAMPHENICOL | 3380 | 4027 | + | NCBI | 029 | efaecium | 203 | 64 | Enterococcus faecium |
| tet(M) | NG\_048213.1 | tetracycline resistance ribosomal protection protein Tet(M) | 99.79 | 100.00 | TETRACYCLINE | 22179 | 24098 | - | NCBI | 029 | efaecium | 203 | 20 | Enterococcus faecium |
| eat(A) | NG\_047762.1 | ABC-F type ribosomal protection protein Eat(A) | 100.00 | 100.00 | PLEUROMUTILIN | 27622 | 29124 | - | NCBI | 029 | efaecium | 203 | 16 | Enterococcus faecium |
| aacA-ENT1 | NG\_052371.1 | aminoglycoside 6'-N-acetyltransferase | 100.00 | 100.00 | AMINOGLYCOSIDE | 34196 | 34744 | - | NCBI | 029 | efaecium | 203 | 11 | Enterococcus faecium |
| msr(C) | NG\_048003.1 | ABC-F type ribosomal protection protein Msr(C) | 98.92 | 100.00 | MACROLIDE | 80919 | 82397 | - | NCBI | 029 | efaecium | 203 | 2 | Enterococcus faecium |
| dfrF | NG\_047755.1 | trimethoprim-resistant dihydrofolate reductase DfrF | 99.60 | 100.00 | TRIMETHOPRIM | 2753 | 3248 | - | NCBI | 029 | efaecium | 203 | 81 | Enterococcus faecium |
| vanX-A | NG\_048477.1 | D-Ala-D-Ala dipeptidase VanX-A | 100.00 | 100.00 | VANCOMYCIN | 152 | 760 | - | NCBI | 029 | efaecium | 203 | 92 | Enterococcus faecium |
| vanA | NG\_048323.1 | D-alanine--(R)-lactate ligase VanA | 100.00 | 100.00 | VANCOMYCIN | 766 | 1797 | - | NCBI | 029 | efaecium | 203 | 92 | Enterococcus faecium |
| vanH-A | NG\_048372.1 | D-lactate dehydrogenase VanH-A | 100.00 | 100.00 | VANCOMYCIN | 1790 | 2758 | - | NCBI | 029 | efaecium | 203 | 92 | Enterococcus faecium |
| vanS-A | NG\_048425.1 | VanA-type vancomycin resistance histidine kinase VanS | 100.00 | 100.00 | VANCOMYCIN | 2973 | 4127 | - | NCBI | 029 | efaecium | 203 | 92 | Enterococcus faecium |
| vanY-A | NG\_048516.1 | D-Ala-D-Ala carboxypeptidase VanY-A | 100.00 | 100.00 | VANCOMYCIN | 10446 | 11357 | - | NCBI | 029 | efaecium | 203 | 64 | Enterococcus faecium |
| erm(B) | NG\_047801.1 | 23S rRNA (adenine(2058)-N(6))-methyltransferase Erm(B) | 99.60 | 99.87 | MACROLIDE | 290 | 1035 | - | NCBI | 029 | efaecium | 203 | 137 | Enterococcus faecium |
| ant(6)-Ia | NG\_047393.1 | aminoglycoside nucleotidyltransferase ANT(6)-Ia | 100.00 | 100.00 | STREPTOMYCIN | 2206 | 3114 | + | NCBI | 029 | efaecium | 203 | 104 | Enterococcus faecium |
| vanR-A | NG\_048399.1 | VanA-type vancomycin resistance DNA-binding response regulator VanR | 100.00 | 100.00 | VANCOMYCIN | 4105 | 4800 | - | NCBI | 029 | efaecium | 203 | 92 | Enterococcus faecium |
| aph(2'')-Ih | NG\_047408.1 | aminoglycoside O-phosphotransferase APH(2'')-Ih | 82.99 | 82.21 | AMIKACIN;GENTAMICIN;KANAMYCIN;TOBRAMYCIN | 852 | 1586 | + | NCBI | 029 | efaecium | 203 | 103 | Enterococcus faecium |
| blaEC-18 | NG\_049083.1 | class C extended-spectrum beta-lactamase EC-18 | 99.21 | 100.00 | CEPHALOSPORIN | 159376 | 160509 | - | NCBI | 030 | ecoli\_achtman\_4 | 155 | 5 | Escherichia coli |
| blaCTX-M-32 | NG\_048982.1 | class A extended-spectrum beta-lactamase CTX-M-32 | 100.00 | 100.00 | CEPHALOSPORIN | 68588 | 69463 | - | NCBI | 030 | ecoli\_achtman\_4 | 155 | 18 | Escherichia coli |
| mph(B) | NG\_047988.1 | Mph(B) family macrolide 2'-phosphotransferase | 100.00 | 100.00 | MACROLIDE | 75531 | 76439 | + | NCBI | 030 | ecoli\_achtman\_4 | 155 | 18 | Escherichia coli |
| catA1 | NG\_047582.1 | type A-1 chloramphenicol O-acetyltransferase | 99.85 | 100.00 | CHLORAMPHENICOL | 4106 | 4765 | + | NCBI | 031 | abaumannii\_2 | 2 | 49 | Acinetobacter baumannii |
| blaADC-73 | NG\_048678.1 | class C extended-spectrum beta-lactamase ADC-73 | 100.00 | 100.00 | CEPHALOSPORIN | 18345 | 19496 | - | NCBI | 031 | abaumannii\_2 | 2 | 38 | Acinetobacter baumannii |
| armA | NG\_047476.1 | ArmA family 16S rRNA (guanine(1405)-N(7))-methyltransferase | 100.00 | 100.00 | GENTAMICIN | 8993 | 9766 | - | NCBI | 031 | abaumannii\_2 | 2 | 28 | Acinetobacter baumannii |
| msr(E) | NG\_048007.1 | ABC-F type ribosomal protection protein Msr(E) | 100.00 | 100.00 | MACROLIDE | 5219 | 6694 | - | NCBI | 031 | abaumannii\_2 | 2 | 28 | Acinetobacter baumannii |
| sul1 | NG\_048082.1 | sulfonamide-resistant dihydropteroate synthase Sul1 | 100.00 | 100.00 | SULFONAMIDE | 2444 | 3283 | - | NCBI | 031 | abaumannii\_2 | 2 | 52 | Acinetobacter baumannii |
| mph(E) | NG\_064660.1 | Mph(E) family macrolide 2'-phosphotransferase | 100.00 | 100.00 | MACROLIDE | 4279 | 5163 | - | NCBI | 031 | abaumannii\_2 | 2 | 28 | Acinetobacter baumannii |
| aph(3'')-Ib | NG\_056002.2 | aminoglycoside O-phosphotransferase APH(3'')-Ib | 99.88 | 98.31 | STREPTOMYCIN | 7772 | 8585 | - | NCBI | 031 | abaumannii\_2 | 2 | 19 | Acinetobacter baumannii |
| aph(6)-Id | NG\_047464.1 | aminoglycoside O-phosphotransferase APH(6)-Id | 100.00 | 100.00 | STREPTOMYCIN | 6936 | 7772 | - | NCBI | 031 | abaumannii\_2 | 2 | 19 | Acinetobacter baumannii |
| tet(B) | NG\_048161.1 | tetracycline efflux MFS transporter Tet(B) | 100.00 | 99.50 | TETRACYCLINE | 2364 | 3563 | - | NCBI | 031 | abaumannii\_2 | 2 | 19 | Acinetobacter baumannii |
| ant(3'')-IIa | NG\_054646.1 | aminoglycoside nucleotidyltransferase ANT(3'')-IIa | 98.61 | 100.00 | SPECTINOMYCIN;STREPTOMYCIN | 126470 | 127258 | + | NCBI | 031 | abaumannii\_2 | 2 | 10 | Acinetobacter baumannii |
| blaOXA-66 | NG\_049806.1 | OXA-51 family carbapenem-hydrolyzing class D beta-lactamase OXA-66 | 100.00 | 100.00 | CARBAPENEM | 315387 | 316211 | - | NCBI | 031 | abaumannii\_2 | 2 | 1 | Acinetobacter baumannii |
| blaOXA-23 | NG\_049525.1 | carbapenem-hydrolyzing class D beta-lactamase OXA-23 | 100.00 | 100.00 | CARBAPENEM | 161 | 982 | + | NCBI | 031 | abaumannii\_2 | 2 | 56 | Acinetobacter baumannii |
| catB7 | NG\_047614.1 | type B-4 chloramphenicol O-acetyltransferase CatB7 | 98.91 | 100.00 | CHLORAMPHENICOL | 49512 | 50150 | - | NCBI | 032 | paeruginosa | 685 | 15 | Pseudomonas aeruginosa |
| blaOXA-494 | NG\_049773.1 | OXA-50 family oxacillin-hydrolyzing class D beta-lactamase OXA-494 | 100.00 | 100.00 | BETA-LACTAM | 33786 | 34574 | - | NCBI | 032 | paeruginosa | 685 | 17 | Pseudomonas aeruginosa |
| aph(3')-IIb | NG\_047424.1 | aminoglycoside O-phosphotransferase APH(3')-IIb | 99.75 | 100.00 | KANAMYCIN | 850 | 1656 | + | NCBI | 032 | paeruginosa | 685 | 196 | Pseudomonas aeruginosa |
| blaPDC-374 | NG\_065926.1 | class C beta-lactamase PDC-374 | 98.94 | 100.00 | CEPHALOSPORIN | 13731 | 14953 | + | NCBI | 032 | paeruginosa | 685 | 126 | Pseudomonas aeruginosa |
| fosA-354827590 | NG\_047883.1 | FosA family fosfomycin resistance glutathione transferase | 99.27 | 100.00 | FOSFOMYCIN | 33038 | 33445 | + | NCBI | 032 | paeruginosa | 685 | 34 | Pseudomonas aeruginosa |
| blaOXA-846 | NG\_066519.1 | OXA-50 family oxacillin-hydrolyzing class D beta-lactamase OXA-846 | 100.00 | 100.00 | BETA-LACTAM | 968 | 1756 | - | NCBI | 033 | paeruginosa | - | 485 | Pseudomonas aeruginosa |
| aac(6')-Il | NG\_047303.1 | aminoglycoside N-acetyltransferase AAC(6')-Il | 100.00 | 100.00 | AMIKACIN;KANAMYCIN;TOBRAMYCIN | 72 | 530 | - | NCBI | 033 | paeruginosa | - | 2424 | Pseudomonas aeruginosa |
| ant(2'')-Ia | NG\_047387.1 | aminoglycoside nucleotidyltransferase ANT(2'')-Ia | 100.00 | 100.00 | GENTAMICIN;KANAMYCIN;TOBRAMYCIN | 175 | 708 | - | NCBI | 033 | paeruginosa | - | 1746 | Pseudomonas aeruginosa |
| aac(6')-Ib' | NG\_051695.1 | aminoglycoside N-acetyltransferase AAC(6')-Ib' | 100.00 | 100.00 | GENTAMICIN | 1204 | 1758 | - | NCBI | 033 | paeruginosa | - | 1143 | Pseudomonas aeruginosa |
| blaGES-1 | NG\_049111.1 | class A extended-spectrum beta-lactamase GES-1 | 99.88 | 100.00 | CEPHALOSPORIN | 237 | 1100 | - | NCBI | 033 | paeruginosa | - | 1143 | Pseudomonas aeruginosa |
| crpP | NG\_062203.1 | ciprofloxacin resistance protein CrpP | 98.48 | 100.00 | FLUOROQUINOLONE | 13783 | 13980 | - | NCBI | 033 | paeruginosa | - | 46 | Pseudomonas aeruginosa |
| aph(3')-IIb | NG\_047424.1 | aminoglycoside O-phosphotransferase APH(3')-IIb | 99.63 | 100.00 | KANAMYCIN | 7654 | 8460 | - | NCBI | 033 | paeruginosa | - | 98 | Pseudomonas aeruginosa |
| blaVEB-9 | NG\_050333.1 | class A extended-spectrum beta-lactamase VEB-9 | 100.00 | 100.00 | CEPHALOSPORIN | 534 | 1433 | + | NCBI | 033 | paeruginosa | - | 191 | Pseudomonas aeruginosa |
| tet(A) | NG\_048156.1 | tetracycline efflux MFS transporter Tet(A) | 100.00 | 96.92 | TETRACYCLINE | 2195 | 3357 | - | NCBI | 033 | paeruginosa | - | 191 | Pseudomonas aeruginosa |
| fosA-354827590 | NG\_047883.1 | FosA family fosfomycin resistance glutathione transferase | 99.75 | 100.00 | FOSFOMYCIN | 3075 | 3482 | - | NCBI | 033 | paeruginosa | - | 263 | Pseudomonas aeruginosa |
| tet(G) | NG\_051907.1 | tetracycline efflux MFS transporter Tet(G) | 100.00 | 100.00 | TETRACYCLINE | 2449 | 3624 | - | NCBI | 033 | paeruginosa | - | 305 | Pseudomonas aeruginosa |
| floR2 | NG\_047875.1 | chloramphenicol/florfenicol efflux MFS transporter FloR2 | 100.00 | 100.00 | CHLORAMPHENICOL;FLORFENICOL | 4561 | 5775 | - | NCBI | 033 | paeruginosa | - | 305 | Pseudomonas aeruginosa |
| blaVIM-2 | NG\_050347.1 | subclass B1 metallo-beta-lactamase VIM-2 | 100.00 | 100.00 | CARBAPENEM | 1177 | 1977 | - | NCBI | 033 | paeruginosa | - | 1017 | Pseudomonas aeruginosa |
| aadA2 | NG\_047343.1 | ANT(3'')-Ia family aminoglycoside nucleotidyltransferase AadA2 | 99.87 | 100.00 | STREPTOMYCIN | 1753 | 2544 | - | NCBI | 033 | paeruginosa | - | 616 | Pseudomonas aeruginosa |
| blaOXA-4 | NG\_049688.1 | OXA-1 family oxacillin-hydrolyzing class D beta-lactamase OXA-4 | 100.00 | 100.00 | BETA-LACTAM | 2657 | 3487 | - | NCBI | 033 | paeruginosa | - | 616 | Pseudomonas aeruginosa |
| aadA1 | NG\_047325.1 | ANT(3'')-Ia family aminoglycoside nucleotidyltransferase AadA1 | 99.87 | 100.00 | STREPTOMYCIN | 1562 | 2353 | - | NCBI | 033 | paeruginosa | - | 617 | Pseudomonas aeruginosa |
| catB7 | NG\_047614.1 | type B-4 chloramphenicol O-acetyltransferase CatB7 | 98.59 | 100.00 | CHLORAMPHENICOL | 4868 | 5506 | + | NCBI | 033 | paeruginosa | - | 317 | Pseudomonas aeruginosa |
| dfrB2 | NG\_047746.1 | trimethoprim-resistant dihydrofolate reductase DfrB2 | 100.00 | 100.00 | TRIMETHOPRIM | 3397 | 3633 | - | NCBI | 033 | paeruginosa | - | 617 | Pseudomonas aeruginosa |
| blaOXA-10 | NG\_049393.1 | oxacillin-hydrolyzing class D beta-lactamase OXA-10 | 99.88 | 100.00 | CEPHALOSPORIN | 2370 | 3170 | - | NCBI | 033 | paeruginosa | - | 617 | Pseudomonas aeruginosa |
| qnrVC1 | NG\_050551.1 | quinolone resistance pentapeptide repeat protein QnrVC1 | 100.00 | 100.00 | QUINOLONE | 1892 | 2548 | - | NCBI | 033 | paeruginosa | - | 763 | Pseudomonas aeruginosa |
| blaPDC-11 | NG\_049877.1 | class C beta-lactamase PDC-11 | 100.00 | 80.82 | CEPHALOSPORIN | 1 | 965 | - | NCBI | 033 | paeruginosa | - | 957 | Pseudomonas aeruginosa |
| aac(3)-Id | NG\_052489.1 | aminoglycoside N-acetyltransferase AAC(3)-Id | 100.00 | 100.00 | GENTAMICIN | 190 | 666 | - | NCBI | 033 | paeruginosa | - | 1017 | Pseudomonas aeruginosa |
| dfrB5 | NG\_047758.1 | trimethoprim-resistant dihydrofolate reductase DfrB5 | 100.00 | 100.00 | TRIMETHOPRIM | 792 | 1028 | - | NCBI | 033 | paeruginosa | - | 1017 | Pseudomonas aeruginosa |
| dfrA6 | NG\_047735.1 | trimethoprim-resistant dihydrofolate reductase DfrA6 | 99.58 | 100.00 | TRIMETHOPRIM | 588 | 1061 | - | NCBI | 033 | paeruginosa | - | 763 | Pseudomonas aeruginosa |
| cmlA6 | NG\_047655.1 | chloramphenicol efflux MFS transporter CmlA6 | 100.00 | 100.00 | CHLORAMPHENICOL | 232 | 1491 | - | NCBI | 033 | paeruginosa | - | 616 | Pseudomonas aeruginosa |
| aacA-ENT1 | NG\_052371.1 | aminoglycoside 6'-N-acetyltransferase | 100.00 | 100.00 | AMINOGLYCOSIDE | 34196 | 34744 | - | NCBI | 034 | efaecium | 192 | 12 | Enterococcus faecium |
| eat(A) | NG\_047762.1 | ABC-F type ribosomal protection protein Eat(A) | 100.00 | 100.00 | PLEUROMUTILIN | 51996 | 53498 | + | NCBI | 034 | efaecium | 192 | 3 | Enterococcus faecium |
| vanR-A | NG\_048399.1 | VanA-type vancomycin resistance DNA-binding response regulator VanR | 100.00 | 100.00 | VANCOMYCIN | 179 | 874 | + | NCBI | 034 | efaecium | 192 | 99 | Enterococcus faecium |
| aph(2'')-Ih | NG\_047408.1 | aminoglycoside O-phosphotransferase APH(2'')-Ih | 82.99 | 82.21 | AMIKACIN;GENTAMICIN;KANAMYCIN;TOBRAMYCIN | 4588 | 5322 | + | NCBI | 034 | efaecium | 192 | 77 | Enterococcus faecium |
| aph(2'')-Ih | NG\_047408.1 | aminoglycoside O-phosphotransferase APH(2'')-Ih | 82.43 | 89.15 | AMIKACIN;GENTAMICIN;KANAMYCIN;TOBRAMYCIN | 6329 | 7125 | + | NCBI | 034 | efaecium | 192 | 77 | Enterococcus faecium |
| vanZ-A | NG\_048534.1 | glycopeptide resistance protein VanZ-A | 100.00 | 100.00 | VANCOMYCIN | 4375 | 4860 | - | NCBI | 034 | efaecium | 192 | 88 | Enterococcus faecium |
| vanY-A | NG\_048516.1 | D-Ala-D-Ala carboxypeptidase VanY-A | 100.00 | 100.00 | VANCOMYCIN | 5013 | 5924 | - | NCBI | 034 | efaecium | 192 | 88 | Enterococcus faecium |
| catA7 | NG\_047568.1 | type A-7 chloramphenicol O-acetyltransferase | 100.00 | 100.00 | CHLORAMPHENICOL | 3380 | 4027 | + | NCBI | 034 | efaecium | 192 | 97 | Enterococcus faecium |
| msr(C) | NG\_048003.1 | ABC-F type ribosomal protection protein Msr(C) | 98.99 | 100.00 | MACROLIDE | 6152 | 7630 | - | NCBI | 034 | efaecium | 192 | 50 | Enterococcus faecium |
| vanS-A | NG\_048425.1 | VanA-type vancomycin resistance histidine kinase VanS | 100.00 | 100.00 | VANCOMYCIN | 852 | 2006 | + | NCBI | 034 | efaecium | 192 | 99 | Enterococcus faecium |
| vanA | NG\_048323.1 | D-alanine--(R)-lactate ligase VanA | 100.00 | 100.00 | VANCOMYCIN | 3182 | 4213 | + | NCBI | 034 | efaecium | 192 | 99 | Enterococcus faecium |
| vanX-A | NG\_048477.1 | D-Ala-D-Ala dipeptidase VanX-A | 100.00 | 100.00 | VANCOMYCIN | 4219 | 4827 | + | NCBI | 034 | efaecium | 192 | 99 | Enterococcus faecium |
| ant(6)-Ia | NG\_047393.1 | aminoglycoside nucleotidyltransferase ANT(6)-Ia | 100.00 | 100.00 | STREPTOMYCIN | 2955 | 3863 | + | NCBI | 034 | efaecium | 192 | 106 | Enterococcus faecium |
| dfrG | NG\_047756.1 | trimethoprim-resistant dihydrofolate reductase DfrG | 100.00 | 100.00 | TRIMETHOPRIM | 402 | 899 | + | NCBI | 034 | efaecium | 192 | 109 | Enterococcus faecium |
| erm(B) | NG\_047801.1 | 23S rRNA (adenine(2058)-N(6))-methyltransferase Erm(B) | 99.60 | 99.87 | MACROLIDE | 290 | 1035 | - | NCBI | 034 | efaecium | 192 | 140 | Enterococcus faecium |
| vanH-A | NG\_048372.1 | D-lactate dehydrogenase VanH-A | 100.00 | 100.00 | VANCOMYCIN | 2221 | 3189 | + | NCBI | 034 | efaecium | 192 | 99 | Enterococcus faecium |
| crpP | NG\_062203.1 | ciprofloxacin resistance protein CrpP | 98.99 | 100.00 | FLUOROQUINOLONE | 14056 | 14253 | - | NCBI | 035 | paeruginosa | - | 122 | Pseudomonas aeruginosa |
| fosA-354827590 | NG\_047883.1 | FosA family fosfomycin resistance glutathione transferase | 98.78 | 100.00 | FOSFOMYCIN | 2014 | 2421 | + | NCBI | 035 | paeruginosa | - | 121 | Pseudomonas aeruginosa |
| aph(3')-IIb | NG\_047424.1 | aminoglycoside O-phosphotransferase APH(3')-IIb | 98.64 | 100.00 | KANAMYCIN | 3014 | 3820 | - | NCBI | 035 | paeruginosa | - | 99 | Pseudomonas aeruginosa |
| catB7 | NG\_047614.1 | type B-4 chloramphenicol O-acetyltransferase CatB7 | 99.69 | 100.00 | CHLORAMPHENICOL | 16769 | 17407 | + | NCBI | 035 | paeruginosa | - | 60 | Pseudomonas aeruginosa |
| blaOXA-396 | NG\_049685.1 | OXA-50 family oxacillin-hydrolyzing class D beta-lactamase OXA-396 | 99.87 | 100.00 | BETA-LACTAM | 11496 | 12284 | + | NCBI | 035 | paeruginosa | - | 56 | Pseudomonas aeruginosa |
| blaOXA-66 | NG\_049806.1 | OXA-51 family carbapenem-hydrolyzing class D beta-lactamase OXA-66 | 100.00 | 100.00 | CARBAPENEM | 18968 | 19792 | - | NCBI | 036 | abaumannii\_2 | 636 | 34 | Acinetobacter baumannii |
| blaOXA-72 | NG\_049813.1 | OXA-24 family carbapenem-hydrolyzing class D beta-lactamase OXA-72 | 100.00 | 100.00 | CARBAPENEM | 2031 | 2858 | - | NCBI | 036 | abaumannii\_2 | 636 | 48 | Acinetobacter baumannii |
| aadA1 | NG\_047324.1 | ANT(3'')-Ia family aminoglycoside nucleotidyltransferase AadA1 | 100.00 | 100.00 | STREPTOMYCIN | 3134 | 3925 | + | NCBI | 036 | abaumannii\_2 | 636 | 56 | Acinetobacter baumannii |
| aac(3)-Ia | NG\_047234.1 | aminoglycoside N-acetyltransferase AAC(3)-Ia | 100.00 | 100.00 | GENTAMICIN | 1626 | 2090 | + | NCBI | 036 | abaumannii\_2 | 636 | 56 | Acinetobacter baumannii |
| ant(3'')-IIa | NG\_054646.1 | aminoglycoside nucleotidyltransferase ANT(3'')-IIa | 100.00 | 100.00 | SPECTINOMYCIN;STREPTOMYCIN | 34639 | 35427 | - | NCBI | 036 | abaumannii\_2 | 636 | 21 | Acinetobacter baumannii |
| blaOXA-23 | NG\_049525.1 | carbapenem-hydrolyzing class D beta-lactamase OXA-23 | 100.00 | 100.00 | CARBAPENEM | 124 | 945 | + | NCBI | 036 | abaumannii\_2 | 636 | 60 | Acinetobacter baumannii |
| blaADC-74 | NG\_048679.1 | class C extended-spectrum beta-lactamase ADC-74 | 100.00 | 100.00 | CEPHALOSPORIN | 695 | 1846 | - | NCBI | 036 | abaumannii\_2 | 636 | 62 | Acinetobacter baumannii |
| sul1 | NG\_048082.1 | sulfonamide-resistant dihydropteroate synthase Sul1 | 100.00 | 100.00 | SULFONAMIDE | 386 | 1225 | + | NCBI | 036 | abaumannii\_2 | 636 | 63 | Acinetobacter baumannii |
| aph(3')-VIa | NG\_047448.1 | aminoglycoside O-phosphotransferase APH(3')-VIa | 99.87 | 100.00 | AMIKACIN;KANAMYCIN | 184 | 963 | + | NCBI | 036 | abaumannii\_2 | 636 | 77 | Acinetobacter baumannii |
| catA1 | NG\_047582.1 | type A-1 chloramphenicol O-acetyltransferase | 99.85 | 100.00 | CHLORAMPHENICOL | 4070 | 4729 | + | NCBI | 036 | abaumannii\_2 | 636 | 52 | Acinetobacter baumannii |
| dfrB2 | NG\_047746.1 | trimethoprim-resistant dihydrofolate reductase DfrB2 | 100.00 | 100.00 | TRIMETHOPRIM | 313 | 549 | + | NCBI | 037 | paeruginosa | 357 | 74 | Pseudomonas aeruginosa |
| aph(3')-IIb | NG\_047424.1 | aminoglycoside O-phosphotransferase APH(3')-IIb | 99.63 | 100.00 | KANAMYCIN | 101500 | 102306 | + | NCBI | 037 | paeruginosa | 357 | 1 | Pseudomonas aeruginosa |
| blaPDC-374 | NG\_065926.1 | class C beta-lactamase PDC-374 | 98.77 | 100.00 | CEPHALOSPORIN | 114620 | 115842 | - | NCBI | 037 | paeruginosa | 357 | 1 | Pseudomonas aeruginosa |
| tet(A) | NG\_048156.1 | tetracycline efflux MFS transporter Tet(A) | 100.00 | 96.92 | TETRACYCLINE | 1950 | 3112 | - | NCBI | 037 | paeruginosa | 357 | 49 | Pseudomonas aeruginosa |
| aadA1 | NG\_047325.1 | ANT(3'')-Ia family aminoglycoside nucleotidyltransferase AadA1 | 99.87 | 100.00 | STREPTOMYCIN | 1593 | 2384 | + | NCBI | 037 | paeruginosa | 357 | 74 | Pseudomonas aeruginosa |
| sul1 | NG\_048082.1 | sulfonamide-resistant dihydropteroate synthase Sul1 | 100.00 | 100.00 | SULFONAMIDE | 429 | 1268 | + | NCBI | 037 | paeruginosa | 357 | 81 | Pseudomonas aeruginosa |
| blaVEB-9 | NG\_050333.1 | class A extended-spectrum beta-lactamase VEB-9 | 100.00 | 100.00 | CEPHALOSPORIN | 289 | 1188 | + | NCBI | 037 | paeruginosa | 357 | 49 | Pseudomonas aeruginosa |
| crpP | NG\_062203.1 | ciprofloxacin resistance protein CrpP | 98.48 | 100.00 | FLUOROQUINOLONE | 586 | 783 | + | NCBI | 037 | paeruginosa | 357 | 56 | Pseudomonas aeruginosa |
| blaOXA-846 | NG\_066519.1 | OXA-50 family oxacillin-hydrolyzing class D beta-lactamase OXA-846 | 100.00 | 100.00 | BETA-LACTAM | 83067 | 83855 | - | NCBI | 037 | paeruginosa | 357 | 11 | Pseudomonas aeruginosa |
| aac(6')-Il | NG\_047303.1 | aminoglycoside N-acetyltransferase AAC(6')-Il | 100.00 | 100.00 | AMIKACIN;KANAMYCIN;TOBRAMYCIN | 11488 | 11946 | + | NCBI | 037 | paeruginosa | 357 | 44 | Pseudomonas aeruginosa |
| catB7 | NG\_047614.1 | type B-4 chloramphenicol O-acetyltransferase CatB7 | 98.59 | 100.00 | CHLORAMPHENICOL | 300678 | 301316 | + | NCBI | 037 | paeruginosa | 357 | 5 | Pseudomonas aeruginosa |
| fosA-354827590 | NG\_047883.1 | FosA family fosfomycin resistance glutathione transferase | 99.75 | 100.00 | FOSFOMYCIN | 162060 | 162467 | + | NCBI | 037 | paeruginosa | 357 | 8 | Pseudomonas aeruginosa |
| blaOXA-10 | NG\_049393.1 | oxacillin-hydrolyzing class D beta-lactamase OXA-10 | 99.88 | 100.00 | CEPHALOSPORIN | 776 | 1576 | + | NCBI | 037 | paeruginosa | 357 | 74 | Pseudomonas aeruginosa |
| aph(6)-Id | NG\_047464.1 | aminoglycoside O-phosphotransferase APH(6)-Id | 100.00 | 100.00 | STREPTOMYCIN | 131 | 967 | - | NCBI | 038 | klebsiella | 17 | 25 | Klebsiella pneumoniae |
| aph(3'')-Ib | NG\_056002.2 | aminoglycoside O-phosphotransferase APH(3'')-Ib | 100.00 | 100.00 | STREPTOMYCIN | 967 | 1794 | - | NCBI | 038 | klebsiella | 17 | 25 | Klebsiella pneumoniae |
| sul2 | NG\_051852.1 | sulfonamide-resistant dihydropteroate synthase Sul2 | 99.75 | 100.00 | SULFONAMIDE | 1831 | 2646 | - | NCBI | 038 | klebsiella | 17 | 25 | Klebsiella pneumoniae |
| tet(D) | NG\_048184.1 | tetracycline efflux MFS transporter Tet(D) | 99.92 | 100.00 | TETRACYCLINE | 828 | 2012 | + | NCBI | 038 | klebsiella | 17 | 28 | Klebsiella pneumoniae |
| aph(3')-Ia | NG\_047430.1 | aminoglycoside O-phosphotransferase APH(3')-Ia | 100.00 | 100.00 | KANAMYCIN | 265 | 1080 | - | NCBI | 038 | klebsiella | 17 | 37 | Klebsiella pneumoniae |
| sul1 | NG\_048082.1 | sulfonamide-resistant dihydropteroate synthase Sul1 | 100.00 | 100.00 | SULFONAMIDE | 7987 | 8826 | + | NCBI | 038 | klebsiella | 17 | 23 | Klebsiella pneumoniae |
| dfrA7 | NG\_047737.1 | trimethoprim-resistant dihydrofolate reductase DfrA7 | 100.00 | 100.00 | TRIMETHOPRIM | 6943 | 7416 | + | NCBI | 038 | klebsiella | 17 | 23 | Klebsiella pneumoniae |
| fosA\_gen | NG\_047884.1 | FosA family fosfomycin resistance glutathione transferase | 100.00 | 98.10 | FOSFOMYCIN | 258936 | 259347 | - | NCBI | 038 | klebsiella | 17 | 8 | Klebsiella pneumoniae |
| oqxA6 | NG\_050424.1 | multidrug efflux RND transporter periplasmic adaptor subunit OqxA6 | 99.75 | 100.00 | PHENICOL;QUINOLONE | 42253 | 43428 | + | NCBI | 038 | klebsiella | 17 | 4 | Klebsiella pneumoniae |
| oqxB19 | NG\_050437.1 | multidrug efflux RND transporter permease subunit OqxB19 | 99.30 | 100.00 | PHENICOL;QUINOLONE | 43452 | 46604 | + | NCBI | 038 | klebsiella | 17 | 4 | Klebsiella pneumoniae |
| catB7 | NG\_047614.1 | type B-4 chloramphenicol O-acetyltransferase CatB7 | 98.59 | 100.00 | CHLORAMPHENICOL | 51855 | 52493 | - | NCBI | 039 | paeruginosa | 357 | 23 | Pseudomonas aeruginosa |
| crpP | NG\_062203.1 | ciprofloxacin resistance protein CrpP | 98.48 | 100.00 | FLUOROQUINOLONE | 63098 | 63295 | - | NCBI | 039 | paeruginosa | 357 | 25 | Pseudomonas aeruginosa |
| aph(3')-IIb | NG\_047424.1 | aminoglycoside O-phosphotransferase APH(3')-IIb | 99.63 | 100.00 | KANAMYCIN | 101500 | 102306 | + | NCBI | 039 | paeruginosa | 357 | 1 | Pseudomonas aeruginosa |
| blaVEB-9 | NG\_050333.1 | class A extended-spectrum beta-lactamase VEB-9 | 100.00 | 100.00 | CEPHALOSPORIN | 222079 | 222978 | + | NCBI | 039 | paeruginosa | 357 | 6 | Pseudomonas aeruginosa |
| tet(A) | NG\_048156.1 | tetracycline efflux MFS transporter Tet(A) | 100.00 | 96.92 | TETRACYCLINE | 223740 | 224902 | - | NCBI | 039 | paeruginosa | 357 | 6 | Pseudomonas aeruginosa |
| sul1 | NG\_048082.1 | sulfonamide-resistant dihydropteroate synthase Sul1 | 100.00 | 100.00 | SULFONAMIDE | 231284 | 232123 | - | NCBI | 039 | paeruginosa | 357 | 6 | Pseudomonas aeruginosa |
| aadA1 | NG\_047325.1 | ANT(3'')-Ia family aminoglycoside nucleotidyltransferase AadA1 | 99.87 | 100.00 | STREPTOMYCIN | 234010 | 234801 | - | NCBI | 039 | paeruginosa | 357 | 6 | Pseudomonas aeruginosa |
| blaPDC-374 | NG\_065926.1 | class C beta-lactamase PDC-374 | 98.77 | 100.00 | CEPHALOSPORIN | 114620 | 115842 | - | NCBI | 039 | paeruginosa | 357 | 1 | Pseudomonas aeruginosa |
| dfrB2 | NG\_047746.1 | trimethoprim-resistant dihydrofolate reductase DfrB2 | 100.00 | 100.00 | TRIMETHOPRIM | 235845 | 236081 | - | NCBI | 039 | paeruginosa | 357 | 6 | Pseudomonas aeruginosa |
| aac(6')-Il | NG\_047303.1 | aminoglycoside N-acetyltransferase AAC(6')-Il | 100.00 | 100.00 | AMIKACIN;KANAMYCIN;TOBRAMYCIN | 236306 | 236764 | - | NCBI | 039 | paeruginosa | 357 | 6 | Pseudomonas aeruginosa |
| ant(2'')-Ia | NG\_047387.1 | aminoglycoside nucleotidyltransferase ANT(2'')-Ia | 100.00 | 100.00 | GENTAMICIN;KANAMYCIN;TOBRAMYCIN | 236823 | 237356 | - | NCBI | 039 | paeruginosa | 357 | 6 | Pseudomonas aeruginosa |
| aac(6')-Il | NG\_047303.1 | aminoglycoside N-acetyltransferase AAC(6')-Il | 100.00 | 100.00 | AMIKACIN;KANAMYCIN;TOBRAMYCIN | 237488 | 237946 | - | NCBI | 039 | paeruginosa | 357 | 6 | Pseudomonas aeruginosa |
| fosA-354827590 | NG\_047883.1 | FosA family fosfomycin resistance glutathione transferase | 99.75 | 100.00 | FOSFOMYCIN | 163396 | 163803 | + | NCBI | 039 | paeruginosa | 357 | 10 | Pseudomonas aeruginosa |
| blaOXA-846 | NG\_066519.1 | OXA-50 family oxacillin-hydrolyzing class D beta-lactamase OXA-846 | 100.00 | 100.00 | BETA-LACTAM | 112491 | 113279 | + | NCBI | 039 | paeruginosa | 357 | 16 | Pseudomonas aeruginosa |
| blaOXA-10 | NG\_049393.1 | oxacillin-hydrolyzing class D beta-lactamase OXA-10 | 99.88 | 100.00 | CEPHALOSPORIN | 234818 | 235618 | - | NCBI | 039 | paeruginosa | 357 | 6 | Pseudomonas aeruginosa |
| tet(A) | NG\_048156.1 | tetracycline efflux MFS transporter Tet(A) | 100.00 | 96.92 | TETRACYCLINE | 223740 | 224902 | - | NCBI | 040 | paeruginosa | 357 | 14 | Pseudomonas aeruginosa |
| blaVEB-9 | NG\_050333.1 | class A extended-spectrum beta-lactamase VEB-9 | 100.00 | 100.00 | CEPHALOSPORIN | 222079 | 222978 | + | NCBI | 040 | paeruginosa | 357 | 14 | Pseudomonas aeruginosa |
| blaOXA-846 | NG\_066519.1 | OXA-50 family oxacillin-hydrolyzing class D beta-lactamase OXA-846 | 100.00 | 100.00 | BETA-LACTAM | 162366 | 163154 | + | NCBI | 040 | paeruginosa | 357 | 13 | Pseudomonas aeruginosa |
| fosA-354827590 | NG\_047883.1 | FosA family fosfomycin resistance glutathione transferase | 99.75 | 100.00 | FOSFOMYCIN | 121070 | 121477 | - | NCBI | 040 | paeruginosa | 357 | 11 | Pseudomonas aeruginosa |
| aph(3')-IIb | NG\_047424.1 | aminoglycoside O-phosphotransferase APH(3')-IIb | 99.63 | 100.00 | KANAMYCIN | 680490 | 681296 | - | NCBI | 040 | paeruginosa | 357 | 1 | Pseudomonas aeruginosa |
| blaOXA-10 | NG\_049393.1 | oxacillin-hydrolyzing class D beta-lactamase OXA-10 | 99.88 | 100.00 | CEPHALOSPORIN | 687 | 1487 | + | NCBI | 040 | paeruginosa | 357 | 53 | Pseudomonas aeruginosa |
| crpP | NG\_062203.1 | ciprofloxacin resistance protein CrpP | 98.48 | 100.00 | FLUOROQUINOLONE | 3765 | 3962 | + | NCBI | 040 | paeruginosa | 357 | 25 | Pseudomonas aeruginosa |
| dfrB2 | NG\_047746.1 | trimethoprim-resistant dihydrofolate reductase DfrB2 | 100.00 | 100.00 | TRIMETHOPRIM | 224 | 460 | + | NCBI | 040 | paeruginosa | 357 | 53 | Pseudomonas aeruginosa |
| aadA1 | NG\_047325.1 | ANT(3'')-Ia family aminoglycoside nucleotidyltransferase AadA1 | 99.87 | 100.00 | STREPTOMYCIN | 1504 | 2295 | + | NCBI | 040 | paeruginosa | 357 | 53 | Pseudomonas aeruginosa |
| ant(2'')-Ia | NG\_047387.1 | aminoglycoside nucleotidyltransferase ANT(2'')-Ia | 100.00 | 100.00 | GENTAMICIN;KANAMYCIN;TOBRAMYCIN | 131 | 664 | + | NCBI | 040 | paeruginosa | 357 | 77 | Pseudomonas aeruginosa |
| aac(6')-Il | NG\_047303.1 | aminoglycoside N-acetyltransferase AAC(6')-Il | 100.00 | 100.00 | AMIKACIN;KANAMYCIN;TOBRAMYCIN | 129 | 587 | - | NCBI | 040 | paeruginosa | 357 | 83 | Pseudomonas aeruginosa |
| blaPDC-374 | NG\_065926.1 | class C beta-lactamase PDC-374 | 98.77 | 100.00 | CEPHALOSPORIN | 666954 | 668176 | + | NCBI | 040 | paeruginosa | 357 | 1 | Pseudomonas aeruginosa |
| sul1 | NG\_048082.1 | sulfonamide-resistant dihydropteroate synthase Sul1 | 100.00 | 100.00 | SULFONAMIDE | 231284 | 232123 | - | NCBI | 040 | paeruginosa | 357 | 14 | Pseudomonas aeruginosa |
| catB7 | NG\_047614.1 | type B-4 chloramphenicol O-acetyltransferase CatB7 | 98.59 | 100.00 | CHLORAMPHENICOL | 21205 | 21843 | + | NCBI | 040 | paeruginosa | 357 | 21 | Pseudomonas aeruginosa |
| eat(A) | NG\_047762.1 | ABC-F type ribosomal protection protein Eat(A) | 100.00 | 100.00 | PLEUROMUTILIN | 27622 | 29124 | - | NCBI | 041 | efaecium | 203 | 17 | Enterococcus faecium |
| blaPDC-374 | NG\_065926.1 | class C beta-lactamase PDC-374 | 98.76 | 91.93 | CEPHALOSPORIN | 1 | 1125 | + | NCBI | 041 | efaecium | 203 | 388 | Enterococcus faecium |
| tet(A) | NG\_048156.1 | tetracycline efflux MFS transporter Tet(A) | 99.91 | 96.92 | TETRACYCLINE | 800 | 1962 | + | NCBI | 041 | efaecium | 203 | 567 | Enterococcus faecium |
| aph(3')-IIb | NG\_047424.1 | aminoglycoside O-phosphotransferase APH(3')-IIb | 99.70 | 83.52 | KANAMYCIN | 1003 | 1676 | - | NCBI | 041 | efaecium | 203 | 627 | Enterococcus faecium |
| catB7 | NG\_047614.1 | type B-4 chloramphenicol O-acetyltransferase CatB7 | 98.64 | 92.18 | CHLORAMPHENICOL | 1 | 589 | - | NCBI | 041 | efaecium | 203 | 647 | Enterococcus faecium |
| erm(B) | NG\_047801.1 | 23S rRNA (adenine(2058)-N(6))-methyltransferase Erm(B) | 99.60 | 99.87 | MACROLIDE | 290 | 1035 | - | NCBI | 041 | efaecium | 203 | 710 | Enterococcus faecium |
| blaVEB-9 | NG\_050333.1 | class A extended-spectrum beta-lactamase VEB-9 | 100.00 | 100.00 | CEPHALOSPORIN | 7938 | 8837 | + | NCBI | 041 | efaecium | 203 | 106 | Enterococcus faecium |
| aadA1 | NG\_047325.1 | ANT(3'')-Ia family aminoglycoside nucleotidyltransferase AadA1 | 99.87 | 100.00 | STREPTOMYCIN | 6166 | 6957 | - | NCBI | 041 | efaecium | 203 | 118 | Enterococcus faecium |
| blaOXA-10 | NG\_049393.1 | oxacillin-hydrolyzing class D beta-lactamase OXA-10 | 99.88 | 100.00 | CEPHALOSPORIN | 6974 | 7774 | - | NCBI | 041 | efaecium | 203 | 118 | Enterococcus faecium |
| dfrB2 | NG\_047746.1 | trimethoprim-resistant dihydrofolate reductase DfrB2 | 99.58 | 100.00 | TRIMETHOPRIM | 8001 | 8237 | - | NCBI | 041 | efaecium | 203 | 118 | Enterococcus faecium |
| vanR-A | NG\_048399.1 | VanA-type vancomycin resistance DNA-binding response regulator VanR | 100.00 | 100.00 | VANCOMYCIN | 179 | 874 | + | NCBI | 041 | efaecium | 203 | 237 | Enterococcus faecium |
| vanS-A | NG\_048425.1 | VanA-type vancomycin resistance histidine kinase VanS | 100.00 | 100.00 | VANCOMYCIN | 852 | 2006 | + | NCBI | 041 | efaecium | 203 | 237 | Enterococcus faecium |
| aph(2'')-Ih | NG\_047408.1 | aminoglycoside O-phosphotransferase APH(2'')-Ih | 82.99 | 82.21 | AMIKACIN;GENTAMICIN;KANAMYCIN;TOBRAMYCIN | 2318 | 3052 | - | NCBI | 041 | efaecium | 203 | 357 | Enterococcus faecium |
| vanH-A | NG\_048372.1 | D-lactate dehydrogenase VanH-A | 100.00 | 100.00 | VANCOMYCIN | 2221 | 3189 | + | NCBI | 041 | efaecium | 203 | 237 | Enterococcus faecium |
| vanA | NG\_048323.1 | D-alanine--(R)-lactate ligase VanA | 100.00 | 100.00 | VANCOMYCIN | 3182 | 4213 | + | NCBI | 041 | efaecium | 203 | 237 | Enterococcus faecium |
| vanX-A | NG\_048477.1 | D-Ala-D-Ala dipeptidase VanX-A | 100.00 | 100.00 | VANCOMYCIN | 4219 | 4827 | + | NCBI | 041 | efaecium | 203 | 237 | Enterococcus faecium |
| sul1 | NG\_048082.1 | sulfonamide-resistant dihydropteroate synthase Sul1 | 100.00 | 100.00 | SULFONAMIDE | 6509 | 7348 | - | NCBI | 041 | efaecium | 203 | 106 | Enterococcus faecium |
| aph(2'')-Ih | NG\_047408.1 | aminoglycoside O-phosphotransferase APH(2'')-Ih | 82.43 | 89.15 | AMIKACIN;GENTAMICIN;KANAMYCIN;TOBRAMYCIN | 515 | 1311 | - | NCBI | 041 | efaecium | 203 | 357 | Enterococcus faecium |
| aacA-ENT1 | NG\_052371.1 | aminoglycoside 6'-N-acetyltransferase | 100.00 | 100.00 | AMINOGLYCOSIDE | 34196 | 34744 | - | NCBI | 041 | efaecium | 203 | 12 | Enterococcus faecium |
| msr(C) | NG\_048003.1 | ABC-F type ribosomal protection protein Msr(C) | 98.92 | 100.00 | MACROLIDE | 80312 | 81790 | - | NCBI | 041 | efaecium | 203 | 1 | Enterococcus faecium |
| tet(M) | NG\_048213.1 | tetracycline resistance ribosomal protection protein Tet(M) | 99.79 | 100.00 | TETRACYCLINE | 22179 | 24098 | - | NCBI | 041 | efaecium | 203 | 20 | Enterococcus faecium |
| blaOXA-846 | NG\_066519.1 | OXA-50 family oxacillin-hydrolyzing class D beta-lactamase OXA-846 | 100.00 | 100.00 | BETA-LACTAM | 2479 | 3267 | - | NCBI | 041 | efaecium | 203 | 34 | Enterococcus faecium |
| vanY-A | NG\_048516.1 | D-Ala-D-Ala carboxypeptidase VanY-A | 100.00 | 100.00 | VANCOMYCIN | 452 | 1363 | + | NCBI | 041 | efaecium | 203 | 82 | Enterococcus faecium |
| vanZ-A | NG\_048534.1 | glycopeptide resistance protein VanZ-A | 100.00 | 100.00 | VANCOMYCIN | 1516 | 2001 | + | NCBI | 041 | efaecium | 203 | 82 | Enterococcus faecium |
| catA7 | NG\_047568.1 | type A-7 chloramphenicol O-acetyltransferase | 100.00 | 100.00 | CHLORAMPHENICOL | 7782 | 8429 | - | NCBI | 041 | efaecium | 203 | 82 | Enterococcus faecium |
| dfrF | NG\_047755.1 | trimethoprim-resistant dihydrofolate reductase DfrF | 99.60 | 100.00 | TRIMETHOPRIM | 6425 | 6920 | + | NCBI | 041 | efaecium | 203 | 102 | Enterococcus faecium |
| ant(6)-Ia | NG\_047393.1 | aminoglycoside nucleotidyltransferase ANT(6)-Ia | 100.00 | 100.00 | STREPTOMYCIN | 638 | 1546 | - | NCBI | 041 | efaecium | 203 | 323 | Enterococcus faecium |
| aac(6')-Ib-AKT | NG\_056043.1 | AAC(6')-Ib family aminoglycoside 6'-N-acetyltransferase | 100.00 | 100.00 | AMIKACIN;KANAMYCIN;TOBRAMYCIN | 415 | 969 | - | NCBI | 042 | klebsiella | 258 | 55 | Klebsiella pneumoniae |
| sul1 | NG\_048082.1 | sulfonamide-resistant dihydropteroate synthase Sul1 | 99.88 | 100.00 | SULFONAMIDE | 2996 | 3835 | - | NCBI | 042 | klebsiella | 258 | 107 | Klebsiella pneumoniae |
| aph(3')-Ia | NG\_047430.1 | aminoglycoside O-phosphotransferase APH(3')-Ia | 100.00 | 100.00 | KANAMYCIN | 264 | 1079 | - | NCBI | 042 | klebsiella | 258 | 943 | Klebsiella pneumoniae |
| blaCTX-M-15 | NG\_048935.1 | class A extended-spectrum beta-lactamase CTX-M-15 | 100.00 | 100.00 | CEPHALOSPORIN | 671 | 1546 | + | NCBI | 042 | klebsiella | 258 | 714 | Klebsiella pneumoniae |
| ble-MBL | NG\_047559.1 | bleomycin binding protein Ble-MBL | 100.00 | 100.00 | BLEOMYCIN | 1435 | 1800 | + | NCBI | 042 | klebsiella | 258 | 544 | Klebsiella pneumoniae |
| aadA2 | NG\_047343.1 | ANT(3'')-Ia family aminoglycoside nucleotidyltransferase AadA2 | 100.00 | 100.00 | STREPTOMYCIN | 4340 | 5131 | - | NCBI | 042 | klebsiella | 258 | 107 | Klebsiella pneumoniae |
| blaNDM-5 | NG\_049337.1 | subclass B1 metallo-beta-lactamase NDM-5 | 100.00 | 100.00 | CARBAPENEM | 619 | 1431 | + | NCBI | 042 | klebsiella | 258 | 544 | Klebsiella pneumoniae |
| aph(3'')-Ib | NG\_056002.2 | aminoglycoside O-phosphotransferase APH(3'')-Ib | 100.00 | 94.81 | STREPTOMYCIN | 5543 | 6327 | + | NCBI | 042 | klebsiella | 258 | 134 | Klebsiella pneumoniae |
| sul2 | NG\_051852.1 | sulfonamide-resistant dihydropteroate synthase Sul2 | 100.00 | 100.00 | SULFONAMIDE | 4691 | 5506 | + | NCBI | 042 | klebsiella | 258 | 134 | Klebsiella pneumoniae |
| aph(6)-Id | NG\_047464.1 | aminoglycoside O-phosphotransferase APH(6)-Id | 100.00 | 100.00 | STREPTOMYCIN | 170 | 1006 | + | NCBI | 042 | klebsiella | 258 | 134 | Klebsiella pneumoniae |
| dfrA12 | NG\_047689.1 | trimethoprim-resistant dihydrofolate reductase DfrA12 | 100.00 | 100.00 | TRIMETHOPRIM | 5539 | 6036 | - | NCBI | 042 | klebsiella | 258 | 107 | Klebsiella pneumoniae |
| oqxB | NG\_048025.1 | multidrug efflux RND transporter permease subunit OqxB | 100.00 | 100.00 | PHENICOL;QUINOLONE | 554012 | 557164 | - | NCBI | 042 | klebsiella | 258 | 1 | Klebsiella pneumoniae |
| blaSHV-158 | NG\_050033.1 | class A beta-lactamase SHV-158 | 99.88 | 100.00 | BETA-LACTAM | 149641 | 150501 | + | NCBI | 042 | klebsiella | 258 | 13 | Klebsiella pneumoniae |
| fosA6 | NG\_051497.1 | fosfomycin resistance glutathione transferase FosA6 | 98.81 | 100.00 | FOSFOMYCIN | 101469 | 101888 | - | NCBI | 042 | klebsiella | 258 | 15 | Klebsiella pneumoniae |
| blaTEM-1 | NG\_050145.1 | class A broad-spectrum beta-lactamase TEM-1 | 99.77 | 100.00 | BETA-LACTAM | 49879 | 50739 | - | NCBI | 042 | klebsiella | 258 | 29 | Klebsiella pneumoniae |
| blaOXA-9 | NG\_049830.1 | oxacillin-hydrolyzing class D beta-lactamase OXA-9 | 99.88 | 100.00 | BETA-LACTAM | 51439 | 52263 | - | NCBI | 042 | klebsiella | 258 | 29 | Klebsiella pneumoniae |
| blaKPC-2 | NG\_049253.1 | carbapenem-hydrolyzing class A beta-lactamase KPC-2 | 100.00 | 100.00 | CARBAPENEM | 14212 | 15093 | + | NCBI | 042 | klebsiella | 258 | 42 | Klebsiella pneumoniae |
| oqxA | NG\_048024.1 | multidrug efflux RND transporter periplasmic adaptor subunit OqxA | 100.00 | 100.00 | PHENICOL;QUINOLONE | 557188 | 558363 | - | NCBI | 042 | klebsiella | 258 | 1 | Klebsiella pneumoniae |
| aph(6)-Id | NG\_047464.1 | aminoglycoside O-phosphotransferase APH(6)-Id | 100.00 | 100.00 | STREPTOMYCIN | 169740 | 170576 | + | NCBI | 043 | paeruginosa | - | 7 | Pseudomonas aeruginosa |
| tet(E) | NG\_048188.1 | tetracycline efflux MFS transporter Tet(E) | 100.00 | 100.00 | TETRACYCLINE | 165512 | 166729 | + | NCBI | 043 | paeruginosa | - | 7 | Pseudomonas aeruginosa |
| aph(3'')-Ib | NG\_056002.2 | aminoglycoside O-phosphotransferase APH(3'')-Ib | 99.88 | 100.00 | STREPTOMYCIN | 168913 | 169740 | + | NCBI | 043 | paeruginosa | - | 7 | Pseudomonas aeruginosa |
| blaOXA-48 | NG\_049762.1 | carbapenem-hydrolyzing class D beta-lactamase OXA-48 | 100.00 | 100.00 | CARBAPENEM | 1409 | 2206 | - | NCBI | 044 | ecoli\_achtman\_4 | 10 | 79 | Escherichia coli |
| blaEC | NG\_047494.1 | BlaEC family class C beta-lactamase | 100.00 | 100.00 | BETA-LACTAM | 129162 | 130295 | + | NCBI | 044 | ecoli\_achtman\_4 | 10 | 2 | Escherichia coli |
| ant(3'')-IIa | NG\_054646.1 | aminoglycoside nucleotidyltransferase ANT(3'')-IIa | 98.86 | 100.00 | SPECTINOMYCIN;STREPTOMYCIN | 34677 | 35465 | - | NCBI | 045 | abaumannii\_2 | 647 | 9 | Acinetobacter baumannii |
| blaADC-179 | NG\_061395.1 | class C beta-lactamase ADC-179 | 98.61 | 100.00 | CEPHALOSPORIN | 392074 | 393225 | + | NCBI | 045 | abaumannii\_2 | 647 | 1 | Acinetobacter baumannii |
| blaOXA-703 | NG\_062325.1 | OXA-51 family carbapenem-hydrolyzing class D beta-lactamase OXA-703 | 100.00 | 100.00 | BETA-LACTAM | 67124 | 67948 | - | NCBI | 045 | abaumannii\_2 | 647 | 2 | Acinetobacter baumannii |
| dfrA12 | NG\_047689.1 | trimethoprim-resistant dihydrofolate reductase DfrA12 | 100.00 | 100.00 | TRIMETHOPRIM | 472 | 969 | + | NCBI | 046 | ecoli\_achtman\_4 | 617 | 266 | Escherichia coli |
| lnu(G) | NG\_051760.1 | lincosamide nucleotidyltransferase Lnu(G) | 100.00 | 100.00 | LINCOSAMIDE | 304 | 1107 | - | NCBI | 046 | ecoli\_achtman\_4 | 617 | 262 | Escherichia coli |
| aac(3)-IId | NG\_047251.1 | aminoglycoside N-acetyltransferase AAC(3)-IId | 99.88 | 100.00 | GENTAMICIN | 214 | 1074 | + | NCBI | 046 | ecoli\_achtman\_4 | 617 | 252 | Escherichia coli |
| sul3 | NG\_048120.1 | sulfonamide-resistant dihydropteroate synthase Sul3 | 100.00 | 100.00 | SULFONAMIDE | 2038 | 2829 | + | NCBI | 046 | ecoli\_achtman\_4 | 617 | 236 | Escherichia coli |
| sul2 | NG\_051852.1 | sulfonamide-resistant dihydropteroate synthase Sul2 | 100.00 | 100.00 | SULFONAMIDE | 357 | 1172 | - | NCBI | 046 | ecoli\_achtman\_4 | 617 | 232 | Escherichia coli |
| floR | NG\_047862.1 | chloramphenicol/florfenicol efflux MFS transporter FloR | 99.83 | 100.00 | CHLORAMPHENICOL;FLORFENICOL | 1134 | 2348 | - | NCBI | 046 | ecoli\_achtman\_4 | 617 | 231 | Escherichia coli |
| tet(B) | NG\_048163.1 | tetracycline efflux MFS transporter Tet(B) | 100.00 | 100.00 | TETRACYCLINE | 2026 | 3231 | + | NCBI | 046 | ecoli\_achtman\_4 | 617 | 203 | Escherichia coli |
| tet(M) | NG\_048237.1 | tetracycline resistance ribosomal protection protein Tet(M) | 97.66 | 100.00 | TETRACYCLINE | 4302 | 6221 | - | NCBI | 046 | ecoli\_achtman\_4 | 617 | 186 | Escherichia coli |
| aadA1 | NG\_052266.1 | ANT(3'')-Ia family aminoglycoside nucleotidyltransferase AadA1 | 99.12 | 100.00 | STREPTOMYCIN | 1583 | 2374 | + | NCBI | 046 | ecoli\_achtman\_4 | 617 | 186 | Escherichia coli |
| cmlA1 | NG\_047648.1 | chloramphenicol efflux MFS transporter CmlA1 | 99.92 | 100.00 | CHLORAMPHENICOL | 231 | 1490 | + | NCBI | 046 | ecoli\_achtman\_4 | 617 | 186 | Escherichia coli |
| blaEC-15 | NG\_049081.1 | class C extended-spectrum beta-lactamase EC-15 | 98.50 | 100.00 | CEPHALOSPORIN | 13443 | 14576 | + | NCBI | 046 | ecoli\_achtman\_4 | 617 | 97 | Escherichia coli |
| mph(A) | NG\_047986.1 | Mph(A) family macrolide 2'-phosphotransferase | 99.67 | 100.00 | MACROLIDE | 96 | 1017 | + | NCBI | 046 | ecoli\_achtman\_4 | 617 | 214 | Escherichia coli |
| blaTEM-35 | NG\_050264.1 | inhibitor-resistant class A broad-spectrum beta-lactamase TEM-35 | 99.88 | 100.00 | BETA-LACTAM | 356 | 1216 | + | NCBI | 046 | ecoli\_achtman\_4 | 617 | 275 | Escherichia coli |
| lnu(F) | NG\_047927.1 | lincosamide nucleotidyltransferase Lnu(F) | 99.88 | 100.00 | LINCOSAMIDE | 113 | 934 | - | NCBI | 046 | ecoli\_achtman\_4 | 617 | 291 | Escherichia coli |
| blaOXA-48 | NG\_049762.1 | carbapenem-hydrolyzing class D beta-lactamase OXA-48 | 100.00 | 100.00 | CARBAPENEM | 53 | 850 | + | NCBI | 046 | ecoli\_achtman\_4 | 617 | 288 | Escherichia coli |
| qnrB19 | NG\_050479.1 | quinolone resistance pentapeptide repeat protein QnrB19 | 100.00 | 100.00 | QUINOLONE | 364 | 1008 | + | NCBI | 047 | ecoli\_achtman\_4 | 13096 | 385 | Escherichia coli |
| blaKPC-2 | NG\_049253.1 | carbapenem-hydrolyzing class A beta-lactamase KPC-2 | 100.00 | 100.00 | CARBAPENEM | 55 | 936 | + | NCBI | 047 | ecoli\_achtman\_4 | 13096 | 296 | Escherichia coli |
| blaEC-15 | NG\_049081.1 | class C extended-spectrum beta-lactamase EC-15 | 98.59 | 100.00 | CEPHALOSPORIN | 6415 | 7548 | + | NCBI | 047 | ecoli\_achtman\_4 | 13096 | 167 | Escherichia coli |
| catB7 | NG\_047614.1 | type B-4 chloramphenicol O-acetyltransferase CatB7 | 99.06 | 100.00 | CHLORAMPHENICOL | 6607 | 7245 | + | NCBI | 048 | paeruginosa | - | 169 | Pseudomonas aeruginosa |
| crpP | NG\_062203.1 | ciprofloxacin resistance protein CrpP | 98.99 | 100.00 | FLUOROQUINOLONE | 3446 | 3643 | + | NCBI | 048 | paeruginosa | - | 85 | Pseudomonas aeruginosa |
| blaOXA-395 | NG\_049684.1 | OXA-50 family oxacillin-hydrolyzing class D beta-lactamase OXA-395 | 100.00 | 100.00 | BETA-LACTAM | 19138 | 19926 | + | NCBI | 048 | paeruginosa | - | 55 | Pseudomonas aeruginosa |
| aph(3')-IIb | NG\_047424.1 | aminoglycoside O-phosphotransferase APH(3')-IIb | 98.76 | 100.00 | KANAMYCIN | 18874 | 19680 | - | NCBI | 048 | paeruginosa | - | 42 | Pseudomonas aeruginosa |
| blaPDC-374 | NG\_065926.1 | class C beta-lactamase PDC-374 | 98.77 | 100.00 | CEPHALOSPORIN | 5338 | 6560 | + | NCBI | 048 | paeruginosa | - | 42 | Pseudomonas aeruginosa |
| fosA-354827590 | NG\_047883.1 | FosA family fosfomycin resistance glutathione transferase | 99.02 | 100.00 | FOSFOMYCIN | 72101 | 72508 | + | NCBI | 048 | paeruginosa | - | 1 | Pseudomonas aeruginosa |
| blaEC-18 | NG\_049083.1 | class C extended-spectrum beta-lactamase EC-18 | 99.12 | 100.00 | CEPHALOSPORIN | 473649 | 474782 | + | NCBI | 049 | ecoli\_achtman\_4 | 101 | 2 | Escherichia coli |
| fosA | NG\_050405.1 | fosfomycin resistance glutathione transferase FosA | 95.78 | 100.00 | FOSFOMYCIN | 47470 | 47895 | - | NCBI | 050 | cronobacter | 420 | 3 | Cronobacter sakazakii |
| fosA | NG\_050405.1 | fosfomycin resistance glutathione transferase FosA | 95.78 | 100.00 | FOSFOMYCIN | 33860 | 34285 | + | NCBI | 050 | cronobacter | 420 | 3 | Cronobacter sakazakii |
| oqxB9 | NG\_050458.1 | multidrug efflux RND transporter permease subunit OqxB9 | 89.10 | 98.95 | PHENICOL;QUINOLONE | 45324 | 48443 | + | NCBI | 050 | cronobacter | 420 | 5 | Cronobacter sakazakii |
| oqxA9 | NG\_050427.1 | multidrug efflux RND transporter periplasmic adaptor subunit OqxA9 | 87.25 | 100.00 | PHENICOL;QUINOLONE | 44125 | 45300 | + | NCBI | 050 | cronobacter | 420 | 5 | Cronobacter sakazakii |
| blaACT-15 | NG\_048602.1 | cephalosporin-hydrolyzing class C beta-lactamase ACT-15 | 100.00 | 100.00 | CEPHALOSPORIN | 31 | 1176 | - | NCBI | 050 | cronobacter | 420 | 118 | Cronobacter sakazakii |
| tet(M) | NG\_048252.1 | tetracycline resistance ribosomal protection protein Tet(M) | 100.00 | 100.00 | TETRACYCLINE | 39220 | 41139 | + | NCBI | 051 | saureus | 239 | 12 | Staphylococcus aureus |
| mecA | NG\_047940.1 | PBP2a family beta-lactam-resistant peptidoglycan transpeptidase MecA | 99.90 | 100.00 | METHICILLIN | 2842 | 4848 | - | NCBI | 051 | saureus | 239 | 31 | Staphylococcus aureus |
| mecR1 | NG\_051163.1 | beta-lactam sensor/signal transducer MecR1 | 100.00 | 100.00 | METHICILLIN | 4948 | 6705 | + | NCBI | 051 | saureus | 239 | 31 | Staphylococcus aureus |
| mecI\_of\_mecA | NG\_055650.1 | mecA-type methicillin resistance repressor MecI | 99.73 | 100.00 | METHICILLIN | 6705 | 7076 | + | NCBI | 051 | saureus | 239 | 31 | Staphylococcus aureus |
| ant(9)-Ia | NG\_047397.1 | aminoglycoside nucleotidyltransferase ANT(9)-Ia | 99.87 | 100.00 | SPECTINOMYCIN | 566 | 1348 | - | NCBI | 051 | saureus | 239 | 52 | Staphylococcus aureus |
| aph(2'')-Ih | NG\_047408.1 | aminoglycoside O-phosphotransferase APH(2'')-Ih | 82.43 | 89.15 | AMIKACIN;GENTAMICIN;KANAMYCIN;TOBRAMYCIN | 1155 | 1951 | + | NCBI | 051 | saureus | 239 | 47 | Staphylococcus aureus |
| blaR1 | NG\_051774.1 | beta-lactam sensor/signal transducer BlaR1 | 98.92 | 100.00 | BETA-LACTAM | 101622 | 103379 | - | NCBI | 051 | saureus | 239 | 7 | Staphylococcus aureus |
| blaI\_of\_Z | NG\_047499.1 | penicillinase repressor BlaI | 100.00 | 100.00 | BETA-LACTAM | 101252 | 101632 | - | NCBI | 051 | saureus | 239 | 7 | Staphylococcus aureus |
| fosB-Saur | NG\_065844.1 | FosB1/FosB3 family fosfomycin resistance bacillithiol transferase | 100.00 | 100.00 | FOSFOMYCIN | 24118 | 24537 | + | NCBI | 051 | saureus | 239 | 6 | Staphylococcus aureus |
| tet(38) | NG\_048135.1 | tetracycline efflux MFS transporter Tet(38) | 100.00 | 100.00 | TETRACYCLINE | 225521 | 226873 | - | NCBI | 051 | saureus | 239 | 2 | Staphylococcus aureus |
| blaZ | NG\_047532.1 | penicillin-hydrolyzing class A beta-lactamase BlaZ | 99.17 | 100.00 | BETA-LACTAM | 103486 | 104331 | + | NCBI | 051 | saureus | 239 | 7 | Staphylococcus aureus |
| blaEC | NG\_047496.1 | BlaEC family class C beta-lactamase | 98.33 | 100.00 | BETA-LACTAM | 31635 | 32768 | + | NCBI | 052 | ecoli\_achtman\_4 | - | 5 | Escherichia coli |
| blaKPC-2 | NG\_049253.1 | carbapenem-hydrolyzing class A beta-lactamase KPC-2 | 100.00 | 100.00 | CARBAPENEM | 4279 | 5160 | - | NCBI | 052 | ecoli\_achtman\_4 | - | 86 | Escherichia coli |
| aph(3')-VIa | NG\_047448.1 | aminoglycoside O-phosphotransferase APH(3')-VIa | 98.46 | 100.00 | AMIKACIN;KANAMYCIN | 192 | 971 | - | NCBI | 052 | ecoli\_achtman\_4 | - | 118 | Escherichia coli |
| qnrB19 | NG\_050479.1 | quinolone resistance pentapeptide repeat protein QnrB19 | 100.00 | 100.00 | QUINOLONE | 258 | 902 | - | NCBI | 052 | ecoli\_achtman\_4 | - | 173 | Escherichia coli |
| sul2 | NG\_051852.1 | sulfonamide-resistant dihydropteroate synthase Sul2 | 100.00 | 85.05 | SULFONAMIDE | 7475 | 8168 | + | NCBI | 052 | ecoli\_achtman\_4 | - | 118 | Escherichia coli |
| blaCMY-152 | NG\_054963.1 | class C beta-lactamase CMY-152 | 100.00 | 100.00 | CEPHALOSPORIN | 479902 | 481047 | + | NCBI | 053 | cfreundii | 139 | 3 | Citrobacter freundii |
| floR2 | NG\_047875.1 | chloramphenicol/florfenicol efflux MFS transporter FloR2 | 100.00 | 100.00 | CHLORAMPHENICOL;FLORFENICOL | 552 | 1766 | - | NCBI | 054 | paeruginosa | 233 | 46 | Pseudomonas aeruginosa |
| blaOXA-486 | NG\_050612.1 | OXA-50 family oxacillin-hydrolyzing class D beta-lactamase OXA-486 | 99.87 | 100.00 | BETA-LACTAM | 297728 | 298516 | - | NCBI | 054 | paeruginosa | 233 | 4 | Pseudomonas aeruginosa |
| aac(6')-Ib' | NG\_051695.1 | aminoglycoside N-acetyltransferase AAC(6')-Ib' | 100.00 | 100.00 | GENTAMICIN | 1204 | 1758 | - | NCBI | 054 | paeruginosa | 233 | 48 | Pseudomonas aeruginosa |
| tet(G) | NG\_051907.1 | tetracycline efflux MFS transporter Tet(G) | 100.00 | 100.00 | TETRACYCLINE | 661 | 1836 | - | NCBI | 054 | paeruginosa | 233 | 49 | Pseudomonas aeruginosa |
| aac(6')-Il | NG\_047303.1 | aminoglycoside N-acetyltransferase AAC(6')-Il | 100.00 | 100.00 | AMIKACIN;KANAMYCIN;TOBRAMYCIN | 2132 | 2590 | - | NCBI | 054 | paeruginosa | 233 | 43 | Pseudomonas aeruginosa |
| blaGES-1 | NG\_049111.1 | class A extended-spectrum beta-lactamase GES-1 | 99.88 | 100.00 | CEPHALOSPORIN | 237 | 1100 | - | NCBI | 054 | paeruginosa | 233 | 48 | Pseudomonas aeruginosa |
| catB7 | NG\_047614.1 | type B-4 chloramphenicol O-acetyltransferase CatB7 | 98.75 | 100.00 | CHLORAMPHENICOL | 52369 | 53007 | - | NCBI | 054 | paeruginosa | 233 | 8 | Pseudomonas aeruginosa |
| dfrB5 | NG\_047758.1 | trimethoprim-resistant dihydrofolate reductase DfrB5 | 100.00 | 100.00 | TRIMETHOPRIM | 794 | 1030 | - | NCBI | 054 | paeruginosa | 233 | 43 | Pseudomonas aeruginosa |
| fosA-354827590 | NG\_047883.1 | FosA family fosfomycin resistance glutathione transferase | 99.27 | 100.00 | FOSFOMYCIN | 218851 | 219258 | + | NCBI | 054 | paeruginosa | 233 | 11 | Pseudomonas aeruginosa |
| aph(3')-IIb | NG\_047424.1 | aminoglycoside O-phosphotransferase APH(3')-IIb | 98.76 | 100.00 | KANAMYCIN | 111868 | 112674 | + | NCBI | 054 | paeruginosa | 233 | 14 | Pseudomonas aeruginosa |
| blaPDC-374 | NG\_065926.1 | class C beta-lactamase PDC-374 | 98.86 | 100.00 | CEPHALOSPORIN | 124988 | 126210 | - | NCBI | 054 | paeruginosa | 233 | 14 | Pseudomonas aeruginosa |
| qnrVC1 | NG\_050551.1 | quinolone resistance pentapeptide repeat protein QnrVC1 | 100.00 | 100.00 | QUINOLONE | 562 | 1218 | + | NCBI | 054 | paeruginosa | 233 | 18 | Pseudomonas aeruginosa |
| blaVIM-2 | NG\_050347.1 | subclass B1 metallo-beta-lactamase VIM-2 | 100.00 | 100.00 | CARBAPENEM | 1179 | 1979 | - | NCBI | 054 | paeruginosa | 233 | 43 | Pseudomonas aeruginosa |
| dfrA6 | NG\_047735.1 | trimethoprim-resistant dihydrofolate reductase DfrA6 | 99.58 | 100.00 | TRIMETHOPRIM | 2049 | 2522 | + | NCBI | 054 | paeruginosa | 233 | 18 | Pseudomonas aeruginosa |
| crpP | NG\_062203.1 | ciprofloxacin resistance protein CrpP | 98.48 | 100.00 | FLUOROQUINOLONE | 22544 | 22741 | - | NCBI | 054 | paeruginosa | 233 | 34 | Pseudomonas aeruginosa |
| aac(3)-Id | NG\_052489.1 | aminoglycoside N-acetyltransferase AAC(3)-Id | 100.00 | 100.00 | GENTAMICIN | 192 | 668 | - | NCBI | 054 | paeruginosa | 233 | 43 | Pseudomonas aeruginosa |
| cmlA6 | NG\_047655.1 | chloramphenicol efflux MFS transporter CmlA6 | 100.00 | 100.00 | CHLORAMPHENICOL | 2425 | 3684 | + | NCBI | 054 | paeruginosa | 233 | 40 | Pseudomonas aeruginosa |
| aadA2 | NG\_047343.1 | ANT(3'')-Ia family aminoglycoside nucleotidyltransferase AadA2 | 99.87 | 100.00 | STREPTOMYCIN | 1372 | 2163 | + | NCBI | 054 | paeruginosa | 233 | 40 | Pseudomonas aeruginosa |
| blaOXA-4 | NG\_049688.1 | OXA-1 family oxacillin-hydrolyzing class D beta-lactamase OXA-4 | 100.00 | 100.00 | BETA-LACTAM | 429 | 1259 | + | NCBI | 054 | paeruginosa | 233 | 40 | Pseudomonas aeruginosa |
| ant(6)-Ia | NG\_047393.1 | aminoglycoside nucleotidyltransferase ANT(6)-Ia | 100.00 | 100.00 | STREPTOMYCIN | 140 | 1048 | - | NCBI | 055 | efaecium | 80 | 124 | Enterococcus faecium |
| aph(3')-IIIa | NG\_047418.1 | aminoglycoside O-phosphotransferase APH(3')-IIIa | 100.00 | 100.00 | AMIKACIN;KANAMYCIN | 628 | 1422 | + | NCBI | 055 | efaecium | 80 | 114 | Enterococcus faecium |
| aph(2'')-Ih | NG\_047408.1 | aminoglycoside O-phosphotransferase APH(2'')-Ih | 82.43 | 89.15 | AMIKACIN;GENTAMICIN;KANAMYCIN;TOBRAMYCIN | 192 | 988 | - | NCBI | 055 | efaecium | 80 | 111 | Enterococcus faecium |
| eat(A) | NG\_047762.1 | ABC-F type ribosomal protection protein Eat(A) | 99.80 | 100.00 | PLEUROMUTILIN | 60142 | 61644 | - | NCBI | 055 | efaecium | 80 | 8 | Enterococcus faecium |
| vanR-A | NG\_048399.1 | VanA-type vancomycin resistance DNA-binding response regulator VanR | 100.00 | 100.00 | VANCOMYCIN | 15161 | 15856 | - | NCBI | 055 | efaecium | 80 | 55 | Enterococcus faecium |
| msr(C) | NG\_048003.1 | ABC-F type ribosomal protection protein Msr(C) | 98.99 | 100.00 | MACROLIDE | 13037 | 14515 | + | NCBI | 055 | efaecium | 80 | 45 | Enterococcus faecium |
| catA7 | NG\_047568.1 | type A-7 chloramphenicol O-acetyltransferase | 100.00 | 100.00 | CHLORAMPHENICOL | 2644 | 3291 | + | NCBI | 055 | efaecium | 80 | 55 | Enterococcus faecium |
| erm(B) | NG\_047801.1 | 23S rRNA (adenine(2058)-N(6))-methyltransferase Erm(B) | 99.60 | 99.87 | MACROLIDE | 162 | 907 | + | NCBI | 055 | efaecium | 80 | 149 | Enterococcus faecium |
| vanY-A | NG\_048516.1 | D-Ala-D-Ala carboxypeptidase VanY-A | 100.00 | 100.00 | VANCOMYCIN | 9710 | 10621 | - | NCBI | 055 | efaecium | 80 | 55 | Enterococcus faecium |
| vanX-A | NG\_048477.1 | D-Ala-D-Ala dipeptidase VanX-A | 100.00 | 100.00 | VANCOMYCIN | 11208 | 11816 | - | NCBI | 055 | efaecium | 80 | 55 | Enterococcus faecium |
| vanA | NG\_048323.1 | D-alanine--(R)-lactate ligase VanA | 100.00 | 100.00 | VANCOMYCIN | 11822 | 12853 | - | NCBI | 055 | efaecium | 80 | 55 | Enterococcus faecium |
| vanH-A | NG\_048372.1 | D-lactate dehydrogenase VanH-A | 100.00 | 100.00 | VANCOMYCIN | 12846 | 13814 | - | NCBI | 055 | efaecium | 80 | 55 | Enterococcus faecium |
| vanS-A | NG\_048425.1 | VanA-type vancomycin resistance histidine kinase VanS | 100.00 | 100.00 | VANCOMYCIN | 14029 | 15183 | - | NCBI | 055 | efaecium | 80 | 55 | Enterococcus faecium |
| aacA-ENT1 | NG\_052371.1 | aminoglycoside 6'-N-acetyltransferase | 100.00 | 100.00 | AMINOGLYCOSIDE | 34194 | 34742 | - | NCBI | 055 | efaecium | 80 | 10 | Enterococcus faecium |
| vanZ-A | NG\_048534.1 | glycopeptide resistance protein VanZ-A | 100.00 | 100.00 | VANCOMYCIN | 9072 | 9557 | - | NCBI | 055 | efaecium | 80 | 55 | Enterococcus faecium |
| ant(3'')-IIa | NG\_054646.1 | aminoglycoside nucleotidyltransferase ANT(3'')-IIa | 98.61 | 100.00 | SPECTINOMYCIN;STREPTOMYCIN | 127875 | 128663 | + | NCBI | 056 | abaumannii\_2 | 2 | 9 | Acinetobacter baumannii |
| aac(3)-Ia | NG\_047234.1 | aminoglycoside N-acetyltransferase AAC(3)-Ia | 100.00 | 100.00 | GENTAMICIN | 2024 | 2488 | - | NCBI | 056 | abaumannii\_2 | 2 | 51 | Acinetobacter baumannii |
| tet(B) | NG\_048161.1 | tetracycline efflux MFS transporter Tet(B) | 100.00 | 99.50 | TETRACYCLINE | 64302 | 65501 | + | NCBI | 056 | abaumannii\_2 | 2 | 19 | Acinetobacter baumannii |
| aph(6)-Id | NG\_047464.1 | aminoglycoside O-phosphotransferase APH(6)-Id | 100.00 | 100.00 | STREPTOMYCIN | 60093 | 60929 | + | NCBI | 056 | abaumannii\_2 | 2 | 19 | Acinetobacter baumannii |
| blaOXA-66 | NG\_049806.1 | OXA-51 family carbapenem-hydrolyzing class D beta-lactamase OXA-66 | 100.00 | 100.00 | CARBAPENEM | 82411 | 83235 | + | NCBI | 056 | abaumannii\_2 | 2 | 5 | Acinetobacter baumannii |
| blaADC-30 | NG\_048652.1 | class C extended-spectrum beta-lactamase ADC-30 | 99.91 | 100.00 | CEPHALOSPORIN | 99 | 1250 | + | NCBI | 056 | abaumannii\_2 | 2 | 2 | Acinetobacter baumannii |
| aph(3'')-Ib | NG\_056002.2 | aminoglycoside O-phosphotransferase APH(3'')-Ib | 99.88 | 98.31 | STREPTOMYCIN | 59280 | 60093 | + | NCBI | 056 | abaumannii\_2 | 2 | 19 | Acinetobacter baumannii |
| vanA | NG\_048323.1 | D-alanine--(R)-lactate ligase VanA | 100.00 | 100.00 | VANCOMYCIN | 3182 | 4213 | + | NCBI | 057 | efaecium | 117 | 94 | Enterococcus faecium |
| erm(B) | NG\_047801.1 | 23S rRNA (adenine(2058)-N(6))-methyltransferase Erm(B) | 99.60 | 99.87 | MACROLIDE | 289 | 1034 | + | NCBI | 057 | efaecium | 117 | 143 | Enterococcus faecium |
| vanH-A | NG\_048372.1 | D-lactate dehydrogenase VanH-A | 100.00 | 100.00 | VANCOMYCIN | 2221 | 3189 | + | NCBI | 057 | efaecium | 117 | 94 | Enterococcus faecium |
| vanS-A | NG\_048425.1 | VanA-type vancomycin resistance histidine kinase VanS | 100.00 | 100.00 | VANCOMYCIN | 852 | 2006 | + | NCBI | 057 | efaecium | 117 | 94 | Enterococcus faecium |
| vanR-A | NG\_048399.1 | VanA-type vancomycin resistance DNA-binding response regulator VanR | 99.86 | 100.00 | VANCOMYCIN | 179 | 874 | + | NCBI | 057 | efaecium | 117 | 94 | Enterococcus faecium |
| catA7 | NG\_047568.1 | type A-7 chloramphenicol O-acetyltransferase | 100.00 | 100.00 | CHLORAMPHENICOL | 7677 | 8324 | - | NCBI | 057 | efaecium | 117 | 63 | Enterococcus faecium |
| vanZ-A | NG\_048534.1 | glycopeptide resistance protein VanZ-A | 100.00 | 100.00 | VANCOMYCIN | 1411 | 1896 | + | NCBI | 057 | efaecium | 117 | 63 | Enterococcus faecium |
| vanX-A | NG\_048477.1 | D-Ala-D-Ala dipeptidase VanX-A | 100.00 | 100.00 | VANCOMYCIN | 4219 | 4827 | + | NCBI | 057 | efaecium | 117 | 94 | Enterococcus faecium |
| ant(6)-Ia | NG\_047393.1 | aminoglycoside nucleotidyltransferase ANT(6)-Ia | 100.00 | 100.00 | STREPTOMYCIN | 2316 | 3224 | + | NCBI | 057 | efaecium | 117 | 101 | Enterococcus faecium |
| msr(C) | NG\_048003.1 | ABC-F type ribosomal protection protein Msr(C) | 98.92 | 100.00 | MACROLIDE | 47300 | 48778 | + | NCBI | 057 | efaecium | 117 | 3 | Enterococcus faecium |
| vanY-A | NG\_048516.1 | D-Ala-D-Ala carboxypeptidase VanY-A | 100.00 | 100.00 | VANCOMYCIN | 347 | 1258 | + | NCBI | 057 | efaecium | 117 | 63 | Enterococcus faecium |
| eat(A) | NG\_047762.1 | ABC-F type ribosomal protection protein Eat(A) | 100.00 | 100.00 | PLEUROMUTILIN | 60123 | 61625 | - | NCBI | 057 | efaecium | 117 | 6 | Enterococcus faecium |
| aacA-ENT1 | NG\_052371.1 | aminoglycoside 6'-N-acetyltransferase | 100.00 | 100.00 | AMINOGLYCOSIDE | 62240 | 62788 | - | NCBI | 057 | efaecium | 117 | 1 | Enterococcus faecium |
| dfrG | NG\_047756.1 | trimethoprim-resistant dihydrofolate reductase DfrG | 100.00 | 100.00 | TRIMETHOPRIM | 4157 | 4654 | - | NCBI | 057 | efaecium | 117 | 10 | Enterococcus faecium |
| blaADC-5 | NG\_048660.1 | class C beta-lactamase ADC-5 | 100.00 | 100.00 | CEPHALOSPORIN | 196736 | 197887 | - | NCBI | 058 | abaumannii\_2 | 79 | 6 | Acinetobacter baumannii |
| sul2 | NG\_051852.1 | sulfonamide-resistant dihydropteroate synthase Sul2 | 100.00 | 100.00 | SULFONAMIDE | 15862 | 16677 | - | NCBI | 058 | abaumannii\_2 | 79 | 34 | Acinetobacter baumannii |
| cmlB1 | NG\_047658.1 | chloramphenicol efflux MFS transporter CmlB1 | 86.49 | 100.00 | CHLORAMPHENICOL | 7669 | 8934 | + | NCBI | 058 | abaumannii\_2 | 79 | 34 | Acinetobacter baumannii |
| aph(6)-Id | NG\_047464.1 | aminoglycoside O-phosphotransferase APH(6)-Id | 100.00 | 100.00 | STREPTOMYCIN | 960 | 1796 | + | NCBI | 058 | abaumannii\_2 | 79 | 34 | Acinetobacter baumannii |
| aph(3'')-Ib | NG\_056002.2 | aminoglycoside O-phosphotransferase APH(3'')-Ib | 99.88 | 100.00 | STREPTOMYCIN | 133 | 960 | + | NCBI | 058 | abaumannii\_2 | 79 | 34 | Acinetobacter baumannii |
| ant(3'')-IIa | NG\_054648.1 | aminoglycoside nucleotidyltransferase ANT(3'')-IIa | 99.49 | 100.00 | SPECTINOMYCIN;STREPTOMYCIN | 50834 | 51622 | + | NCBI | 058 | abaumannii\_2 | 79 | 15 | Acinetobacter baumannii |
| aph(3')-VIa | NG\_047448.1 | aminoglycoside O-phosphotransferase APH(3')-VIa | 99.87 | 100.00 | AMIKACIN;KANAMYCIN | 1839 | 2618 | - | NCBI | 058 | abaumannii\_2 | 79 | 49 | Acinetobacter baumannii |
| blaOXA-65 | NG\_049805.1 | OXA-51 family carbapenem-hydrolyzing class D beta-lactamase OXA-65 | 100.00 | 100.00 | CARBAPENEM | 162102 | 162926 | + | NCBI | 058 | abaumannii\_2 | 79 | 4 | Acinetobacter baumannii |
| blaOXA-23 | NG\_049525.1 | carbapenem-hydrolyzing class D beta-lactamase OXA-23 | 100.00 | 100.00 | CARBAPENEM | 154 | 975 | + | NCBI | 058 | abaumannii\_2 | 79 | 49 | Acinetobacter baumannii |
| ant(3'')-IIa | NG\_054646.1 | aminoglycoside nucleotidyltransferase ANT(3'')-IIa | 98.61 | 100.00 | SPECTINOMYCIN;STREPTOMYCIN | 22774 | 23562 | - | NCBI | 059 | abaumannii\_2 | 2 | 9 | Acinetobacter baumannii |
| aph(3'')-Ib | NG\_056002.2 | aminoglycoside O-phosphotransferase APH(3'')-Ib | 99.88 | 98.31 | STREPTOMYCIN | 59316 | 60129 | + | NCBI | 059 | abaumannii\_2 | 2 | 18 | Acinetobacter baumannii |
| aph(6)-Id | NG\_047464.1 | aminoglycoside O-phosphotransferase APH(6)-Id | 100.00 | 100.00 | STREPTOMYCIN | 60129 | 60965 | + | NCBI | 059 | abaumannii\_2 | 2 | 18 | Acinetobacter baumannii |
| tet(B) | NG\_048161.1 | tetracycline efflux MFS transporter Tet(B) | 100.00 | 99.50 | TETRACYCLINE | 64338 | 65537 | + | NCBI | 059 | abaumannii\_2 | 2 | 18 | Acinetobacter baumannii |
| blaOXA-72 | NG\_049813.1 | OXA-24 family carbapenem-hydrolyzing class D beta-lactamase OXA-72 | 100.00 | 100.00 | CARBAPENEM | 3262 | 4089 | - | NCBI | 059 | abaumannii\_2 | 2 | 47 | Acinetobacter baumannii |
| blaOXA-66 | NG\_049806.1 | OXA-51 family carbapenem-hydrolyzing class D beta-lactamase OXA-66 | 100.00 | 100.00 | CARBAPENEM | 212633 | 213457 | - | NCBI | 059 | abaumannii\_2 | 2 | 2 | Acinetobacter baumannii |
| blaADC-30 | NG\_048652.1 | class C extended-spectrum beta-lactamase ADC-30 | 100.00 | 100.00 | CEPHALOSPORIN | 137 | 1288 | + | NCBI | 059 | abaumannii\_2 | 2 | 7 | Acinetobacter baumannii |
| blaTEM-12 | NG\_050163.1 | class A extended-spectrum beta-lactamase TEM-12 | 99.88 | 100.00 | CEPHALOSPORIN | 144 | 1004 | - | NCBI | 059 | abaumannii\_2 | 2 | 64 | Acinetobacter baumannii |
| sul1 | NG\_048082.1 | sulfonamide-resistant dihydropteroate synthase Sul1 | 100.00 | 100.00 | SULFONAMIDE | 737 | 1576 | - | NCBI | 059 | abaumannii\_2 | 2 | 65 | Acinetobacter baumannii |
| aph(3')-Ia | NG\_047431.1 | aminoglycoside O-phosphotransferase APH(3')-Ia | 100.00 | 100.00 | KANAMYCIN | 193 | 1008 | + | NCBI | 059 | abaumannii\_2 | 2 | 75 | Acinetobacter baumannii |
| sul2 | NG\_051852.1 | sulfonamide-resistant dihydropteroate synthase Sul2 | 100.00 | 100.00 | SULFONAMIDE | 146 | 961 | + | NCBI | 059 | abaumannii\_2 | 2 | 78 | Acinetobacter baumannii |
| aac(6')-Ip | NG\_047307.2 | aminoglycoside 6'-N-acetyltransferase AAC(6')-Ip | 99.66 | 100.00 | AMINOGLYCOSIDE | 175 | 757 | + | NCBI | 059 | abaumannii\_2 | 2 | 87 | Acinetobacter baumannii |
| aac(3)-Ia | NG\_047234.1 | aminoglycoside N-acetyltransferase AAC(3)-Ia | 100.00 | 100.00 | GENTAMICIN | 2060 | 2524 | - | NCBI | 059 | abaumannii\_2 | 2 | 63 | Acinetobacter baumannii |
| blaNDM-5 | NG\_049337.1 | subclass B1 metallo-beta-lactamase NDM-5 | 100.00 | 100.00 | CARBAPENEM | 8593 | 9405 | - | NCBI | 060 | ecoli\_achtman\_4 | 167 | 61 | Escherichia coli |
| erm(B) | NG\_047804.1 | 23S rRNA (adenine(2058)-N(6))-methyltransferase Erm(B) | 99.86 | 100.00 | MACROLIDE | 417 | 1154 | + | NCBI | 060 | ecoli\_achtman\_4 | 167 | 74 | Escherichia coli |
| mph(A) | NG\_047986.1 | Mph(A) family macrolide 2'-phosphotransferase | 98.71 | 100.00 | MACROLIDE | 196 | 1126 | + | NCBI | 060 | ecoli\_achtman\_4 | 167 | 64 | Escherichia coli |
| rmtB1 | NG\_048058.1 | 16S rRNA (guanine(1405)-N(7))-methyltransferase RmtB1 | 100.00 | 100.00 | AMINOGLYCOSIDE | 6258 | 7013 | + | NCBI | 060 | ecoli\_achtman\_4 | 167 | 64 | Escherichia coli |
| blaTEM-1 | NG\_050145.1 | class A broad-spectrum beta-lactamase TEM-1 | 100.00 | 100.00 | BETA-LACTAM | 5228 | 6088 | + | NCBI | 060 | ecoli\_achtman\_4 | 167 | 64 | Escherichia coli |
| blaEC-15 | NG\_049081.1 | class C extended-spectrum beta-lactamase EC-15 | 98.50 | 100.00 | CEPHALOSPORIN | 81269 | 82402 | + | NCBI | 060 | ecoli\_achtman\_4 | 167 | 4 | Escherichia coli |
| dfrA12 | NG\_047689.1 | trimethoprim-resistant dihydrofolate reductase DfrA12 | 100.00 | 100.00 | TRIMETHOPRIM | 1240 | 1737 | + | NCBI | 060 | ecoli\_achtman\_4 | 167 | 61 | Escherichia coli |
| aadA2 | NG\_047343.1 | ANT(3'')-Ia family aminoglycoside nucleotidyltransferase AadA2 | 100.00 | 100.00 | STREPTOMYCIN | 2145 | 2936 | + | NCBI | 060 | ecoli\_achtman\_4 | 167 | 61 | Escherichia coli |
| sul1 | NG\_048082.1 | sulfonamide-resistant dihydropteroate synthase Sul1 | 100.00 | 100.00 | SULFONAMIDE | 3441 | 4280 | + | NCBI | 060 | ecoli\_achtman\_4 | 167 | 61 | Escherichia coli |
| ble-MBL | NG\_047559.1 | bleomycin binding protein Ble-MBL | 100.00 | 100.00 | BLEOMYCIN | 8224 | 8589 | - | NCBI | 060 | ecoli\_achtman\_4 | 167 | 61 | Escherichia coli |
| erm(T) | NG\_047841.1 | 23S rRNA (adenine(2058)-N(6))-methyltransferase Erm(T) | 100.00 | 100.00 | MACROLIDE | 696 | 1430 | - | NCBI | 061 | efaecium | 80 | 246 | Enterococcus faecium |
| vanA | NG\_048323.1 | D-alanine--(R)-lactate ligase VanA | 100.00 | 100.00 | VANCOMYCIN | 490 | 1521 | - | NCBI | 061 | efaecium | 80 | 232 | Enterococcus faecium |
| ant(6)-Ia | NG\_047393.1 | aminoglycoside nucleotidyltransferase ANT(6)-Ia | 100.00 | 100.00 | STREPTOMYCIN | 1455 | 2363 | - | NCBI | 061 | efaecium | 80 | 169 | Enterococcus faecium |
| erm(B) | NG\_047802.1 | 23S rRNA (adenine(2058)-N(6))-methyltransferase Erm(B) | 99.86 | 100.00 | MACROLIDE | 487 | 1224 | - | NCBI | 061 | efaecium | 80 | 251 | Enterococcus faecium |
| aph(2'')-Ih | NG\_047408.1 | aminoglycoside O-phosphotransferase APH(2'')-Ih | 82.64 | 88.26 | AMIKACIN;GENTAMICIN;KANAMYCIN;TOBRAMYCIN | 121 | 909 | - | NCBI | 061 | efaecium | 80 | 231 | Enterococcus faecium |
| aph(3')-IIIa | NG\_047418.1 | aminoglycoside O-phosphotransferase APH(3')-IIIa | 100.00 | 100.00 | AMIKACIN;KANAMYCIN | 29 | 823 | - | NCBI | 061 | efaecium | 80 | 169 | Enterococcus faecium |
| vanY-A | NG\_048516.1 | D-Ala-D-Ala carboxypeptidase VanY-A | 100.00 | 100.00 | VANCOMYCIN | 5394 | 6305 | - | NCBI | 061 | efaecium | 80 | 155 | Enterococcus faecium |
| vanZ-A | NG\_048534.1 | glycopeptide resistance protein VanZ-A | 100.00 | 100.00 | VANCOMYCIN | 4756 | 5241 | - | NCBI | 061 | efaecium | 80 | 155 | Enterococcus faecium |
| aacA-ENT1 | NG\_052371.1 | aminoglycoside 6'-N-acetyltransferase | 100.00 | 100.00 | AMINOGLYCOSIDE | 23 | 571 | - | NCBI | 061 | efaecium | 80 | 137 | Enterococcus faecium |
| tet(L) | NG\_048203.1 | tetracycline efflux MFS transporter Tet(L) | 99.85 | 97.53 | TETRACYCLINE | 8496 | 9838 | + | NCBI | 061 | efaecium | 80 | 91 | Enterococcus faecium |
| tet(M) | NG\_048240.1 | tetracycline resistance ribosomal protection protein Tet(M) | 99.43 | 100.00 | TETRACYCLINE | 6448 | 8367 | + | NCBI | 061 | efaecium | 80 | 91 | Enterococcus faecium |
| tet(M) | NG\_048240.1 | tetracycline resistance ribosomal protection protein Tet(M) | 99.43 | 100.00 | TETRACYCLINE | 4418 | 6337 | - | NCBI | 061 | efaecium | 80 | 91 | Enterococcus faecium |
| tet(L) | NG\_048203.1 | tetracycline efflux MFS transporter Tet(L) | 99.85 | 97.53 | TETRACYCLINE | 2947 | 4289 | - | NCBI | 061 | efaecium | 80 | 91 | Enterococcus faecium |
| vanR-A | NG\_048399.1 | VanA-type vancomycin resistance DNA-binding response regulator VanR | 100.00 | 100.00 | VANCOMYCIN | 1148 | 1843 | - | NCBI | 061 | efaecium | 80 | 58 | Enterococcus faecium |
| msr(C) | NG\_048003.1 | ABC-F type ribosomal protection protein Msr(C) | 98.92 | 100.00 | MACROLIDE | 1775 | 3253 | + | NCBI | 061 | efaecium | 80 | 164 | Enterococcus faecium |
| eat(A) | NG\_047762.1 | ABC-F type ribosomal protection protein Eat(A) | 100.00 | 100.00 | PLEUROMUTILIN | 5825 | 7327 | + | NCBI | 061 | efaecium | 80 | 22 | Enterococcus faecium |
| vanS-A | NG\_048425.1 | VanA-type vancomycin resistance histidine kinase VanS | 100.00 | 99.48 | VANCOMYCIN | 22 | 1170 | - | NCBI | 061 | efaecium | 80 | 58 | Enterococcus faecium |
| sat4 | NG\_048072.1 | streptothricin N-acetyltransferase Sat4 | 100.00 | 100.00 | STREPTOTHRICIN | 916 | 1458 | - | NCBI | 061 | efaecium | 80 | 169 | Enterococcus faecium |
| tet(A) | NG\_048154.1 | tetracycline efflux MFS transporter Tet(A) | 100.00 | 100.00 | TETRACYCLINE | 2015 | 3214 | + | NCBI | 062 | ecoli\_achtman\_4 | 58 | 61 | Escherichia coli |
| aph(3'')-Ib | NG\_056002.2 | aminoglycoside O-phosphotransferase APH(3'')-Ib | 99.88 | 100.00 | STREPTOMYCIN | 27046 | 27873 | - | NCBI | 062 | ecoli\_achtman\_4 | 58 | 42 | Escherichia coli |
| aph(6)-Id | NG\_047466.1 | aminoglycoside O-phosphotransferase APH(6)-Id | 100.00 | 100.00 | STREPTOMYCIN | 26210 | 27046 | - | NCBI | 062 | ecoli\_achtman\_4 | 58 | 42 | Escherichia coli |
| blaTEM-1 | NG\_050145.1 | class A broad-spectrum beta-lactamase TEM-1 | 100.00 | 100.00 | BETA-LACTAM | 25119 | 25979 | + | NCBI | 062 | ecoli\_achtman\_4 | 58 | 42 | Escherichia coli |
| blaEC-18 | NG\_049083.1 | class C extended-spectrum beta-lactamase EC-18 | 99.12 | 100.00 | CEPHALOSPORIN | 34064 | 35197 | - | NCBI | 062 | ecoli\_achtman\_4 | 58 | 8 | Escherichia coli |
| sul2 | NG\_051852.1 | sulfonamide-resistant dihydropteroate synthase Sul2 | 100.00 | 100.00 | SULFONAMIDE | 27910 | 28725 | - | NCBI | 062 | ecoli\_achtman\_4 | 58 | 42 | Escherichia coli |
| aph(2'')-If | NG\_047406.1 | aminoglycoside O-phosphotransferase APH(2'')-If | 82.65 | 84.40 | AMIKACIN;GENTAMICIN;KANAMYCIN;TOBRAMYCIN | 132 | 886 | + | NCBI | 063 | efaecium | 80 | 160 | Enterococcus faecium |
| erm(B) | NG\_047801.1 | 23S rRNA (adenine(2058)-N(6))-methyltransferase Erm(B) | 99.60 | 99.87 | MACROLIDE | 565 | 1310 | - | NCBI | 063 | efaecium | 80 | 138 | Enterococcus faecium |
| vanZ-A | NG\_048534.1 | glycopeptide resistance protein VanZ-A | 100.00 | 100.00 | VANCOMYCIN | 1411 | 1896 | + | NCBI | 063 | efaecium | 80 | 125 | Enterococcus faecium |
| vanY-A | NG\_048516.1 | D-Ala-D-Ala carboxypeptidase VanY-A | 100.00 | 100.00 | VANCOMYCIN | 347 | 1258 | + | NCBI | 063 | efaecium | 80 | 125 | Enterococcus faecium |
| dfrG | NG\_047756.1 | trimethoprim-resistant dihydrofolate reductase DfrG | 100.00 | 100.00 | TRIMETHOPRIM | 2506 | 3003 | - | NCBI | 063 | efaecium | 80 | 114 | Enterococcus faecium |
| vanX-A | NG\_048477.1 | D-Ala-D-Ala dipeptidase VanX-A | 100.00 | 100.00 | VANCOMYCIN | 4219 | 4827 | + | NCBI | 063 | efaecium | 80 | 103 | Enterococcus faecium |
| vanH-A | NG\_048372.1 | D-lactate dehydrogenase VanH-A | 100.00 | 100.00 | VANCOMYCIN | 2221 | 3189 | + | NCBI | 063 | efaecium | 80 | 103 | Enterococcus faecium |
| vanS-A | NG\_048425.1 | VanA-type vancomycin resistance histidine kinase VanS | 100.00 | 100.00 | VANCOMYCIN | 852 | 2006 | + | NCBI | 063 | efaecium | 80 | 103 | Enterococcus faecium |
| vanR-A | NG\_048399.1 | VanA-type vancomycin resistance DNA-binding response regulator VanR | 100.00 | 100.00 | VANCOMYCIN | 179 | 874 | + | NCBI | 063 | efaecium | 80 | 103 | Enterococcus faecium |
| tet(L) | NG\_048203.1 | tetracycline efflux MFS transporter Tet(L) | 99.85 | 97.53 | TETRACYCLINE | 3002 | 4344 | - | NCBI | 063 | efaecium | 80 | 97 | Enterococcus faecium |
| catA7 | NG\_047568.1 | type A-7 chloramphenicol O-acetyltransferase | 100.00 | 100.00 | CHLORAMPHENICOL | 5644 | 6291 | - | NCBI | 063 | efaecium | 80 | 82 | Enterococcus faecium |
| aacA-ENT1 | NG\_052371.1 | aminoglycoside 6'-N-acetyltransferase | 100.00 | 100.00 | AMINOGLYCOSIDE | 14047 | 14595 | + | NCBI | 063 | efaecium | 80 | 20 | Enterococcus faecium |
| msr(C) | NG\_048003.1 | ABC-F type ribosomal protection protein Msr(C) | 98.99 | 100.00 | MACROLIDE | 43262 | 44740 | - | NCBI | 063 | efaecium | 80 | 8 | Enterococcus faecium |
| vanA | NG\_048323.1 | D-alanine--(R)-lactate ligase VanA | 100.00 | 100.00 | VANCOMYCIN | 3182 | 4213 | + | NCBI | 063 | efaecium | 80 | 103 | Enterococcus faecium |
| eat(A) | NG\_047762.1 | ABC-F type ribosomal protection protein Eat(A) | 100.00 | 100.00 | PLEUROMUTILIN | 60273 | 61775 | - | NCBI | 063 | efaecium | 80 | 5 | Enterococcus faecium |
| rmtC | NG\_048060.1 | RmtC family 16S rRNA (guanine(1405)-N(7))-methyltransferase | 100.00 | 100.00 | AMINOGLYCOSIDE | 10460 | 11305 | + | NCBI | 064 | ecloacae | 277 | 74 | Enterobacter cloacae |
| fosA | NG\_050405.1 | fosfomycin resistance glutathione transferase FosA | 96.95 | 100.00 | FOSFOMYCIN | 17044 | 17469 | - | NCBI | 064 | ecloacae | 277 | 46 | Enterobacter cloacae |
| sul1 | NG\_048102.1 | sulfonamide-resistant dihydropteroate synthase Sul1 | 99.88 | 100.00 | SULFONAMIDE | 3296 | 4135 | - | NCBI | 064 | ecloacae | 277 | 74 | Enterobacter cloacae |
| blaNDM-1 | NG\_049326.1 | subclass B1 metallo-beta-lactamase NDM-1 | 100.00 | 100.00 | CARBAPENEM | 11863 | 12675 | + | NCBI | 064 | ecloacae | 277 | 74 | Enterobacter cloacae |
| ble-MBL | NG\_047559.1 | bleomycin binding protein Ble-MBL | 100.00 | 100.00 | BLEOMYCIN | 12679 | 13044 | + | NCBI | 064 | ecloacae | 277 | 74 | Enterobacter cloacae |
| aph(6)-Id | NG\_047464.1 | aminoglycoside O-phosphotransferase APH(6)-Id | 99.88 | 100.00 | STREPTOMYCIN | 67 | 903 | + | NCBI | 064 | ecloacae | 277 | 138 | Enterobacter cloacae |
| blaOXA-2 | NG\_049496.1 | oxacillin-hydrolyzing class D beta-lactamase OXA-2 | 100.00 | 100.00 | BETA-LACTAM | 294 | 1121 | - | NCBI | 064 | ecloacae | 277 | 176 | Enterobacter cloacae |
| aac(6')-Ib4 | NG\_051844.1 | aminoglycoside N-acetyltransferase AAC(6')-Ib4 | 100.00 | 100.00 | GENTAMICIN | 1150 | 1704 | - | NCBI | 064 | ecloacae | 277 | 176 | Enterobacter cloacae |
| aph(3'')-Ib | NG\_056002.2 | aminoglycoside O-phosphotransferase APH(3'')-Ib | 97.57 | 89.49 | STREPTOMYCIN | 1 | 741 | - | NCBI | 064 | ecloacae | 277 | 246 | Enterobacter cloacae |
| blaACT-57 | NG\_061418.1 | cephalosporin-hydrolyzing class C beta-lactamase ACT-57 | 98.60 | 100.00 | CEPHALOSPORIN | 124454 | 125599 | + | NCBI | 064 | ecloacae | 277 | 5 | Enterobacter cloacae |
| rmtC | NG\_048060.1 | RmtC family 16S rRNA (guanine(1405)-N(7))-methyltransferase | 100.00 | 100.00 | AMINOGLYCOSIDE | 10590 | 11435 | + | NCBI | 065 | ecoli\_achtman\_4 | 10 | 101 | Escherichia coli |
| sul1 | NG\_048102.1 | sulfonamide-resistant dihydropteroate synthase Sul1 | 99.88 | 100.00 | SULFONAMIDE | 3196 | 4035 | - | NCBI | 065 | ecoli\_achtman\_4 | 10 | 101 | Escherichia coli |
| blaEC | NG\_047494.1 | BlaEC family class C beta-lactamase | 100.00 | 100.00 | BETA-LACTAM | 4539 | 5672 | - | NCBI | 065 | ecoli\_achtman\_4 | 10 | 92 | Escherichia coli |
| ble-MBL | NG\_047559.1 | bleomycin binding protein Ble-MBL | 100.00 | 100.00 | BLEOMYCIN | 12809 | 13174 | + | NCBI | 065 | ecoli\_achtman\_4 | 10 | 101 | Escherichia coli |
| blaNDM-1 | NG\_049326.1 | subclass B1 metallo-beta-lactamase NDM-1 | 100.00 | 100.00 | CARBAPENEM | 11993 | 12805 | + | NCBI | 065 | ecoli\_achtman\_4 | 10 | 101 | Escherichia coli |
| armA | NG\_047476.1 | ArmA family 16S rRNA (guanine(1405)-N(7))-methyltransferase | 100.00 | 100.00 | GENTAMICIN | 61601 | 62374 | + | NCBI | 066 | abaumannii\_2 | 1 | 17 | Acinetobacter baumannii |
| blaOXA-69 | NG\_049809.1 | OXA-51 family carbapenem-hydrolyzing class D beta-lactamase OXA-69 | 100.00 | 100.00 | CARBAPENEM | 8429 | 9253 | - | NCBI | 066 | abaumannii\_2 | 1 | 5 | Acinetobacter baumannii |
| ant(3'')-IIa | NG\_054648.1 | aminoglycoside nucleotidyltransferase ANT(3'')-IIa | 99.62 | 100.00 | SPECTINOMYCIN;STREPTOMYCIN | 34808 | 35596 | - | NCBI | 066 | abaumannii\_2 | 1 | 10 | Acinetobacter baumannii |
| msr(E) | NG\_048007.1 | ABC-F type ribosomal protection protein Msr(E) | 100.00 | 100.00 | MACROLIDE | 64673 | 66148 | + | NCBI | 066 | abaumannii\_2 | 1 | 17 | Acinetobacter baumannii |
| mph(E) | NG\_064660.1 | Mph(E) family macrolide 2'-phosphotransferase | 100.00 | 100.00 | MACROLIDE | 66204 | 67088 | + | NCBI | 066 | abaumannii\_2 | 1 | 17 | Acinetobacter baumannii |
| blaADC-187 | NG\_064710.1 | class C beta-lactamase ADC-187 | 100.00 | 100.00 | CEPHALOSPORIN | 39548 | 40699 | - | NCBI | 066 | abaumannii\_2 | 1 | 24 | Acinetobacter baumannii |
| tet(A) | NG\_048154.1 | tetracycline efflux MFS transporter Tet(A) | 100.00 | 100.00 | TETRACYCLINE | 5577 | 6776 | + | NCBI | 066 | abaumannii\_2 | 1 | 29 | Acinetobacter baumannii |
| catA1 | NG\_047582.1 | type A-1 chloramphenicol O-acetyltransferase | 99.85 | 100.00 | CHLORAMPHENICOL | 10622 | 11281 | - | NCBI | 066 | abaumannii\_2 | 1 | 29 | Acinetobacter baumannii |
| aac(3)-Ia | NG\_047234.1 | aminoglycoside N-acetyltransferase AAC(3)-Ia | 100.00 | 100.00 | GENTAMICIN | 2392 | 2856 | - | NCBI | 066 | abaumannii\_2 | 1 | 38 | Acinetobacter baumannii |
| blaOXA-23 | NG\_049525.1 | carbapenem-hydrolyzing class D beta-lactamase OXA-23 | 100.00 | 100.00 | CARBAPENEM | 139 | 960 | + | NCBI | 066 | abaumannii\_2 | 1 | 49 | Acinetobacter baumannii |
| aadA1 | NG\_047324.1 | ANT(3'')-Ia family aminoglycoside nucleotidyltransferase AadA1 | 100.00 | 100.00 | STREPTOMYCIN | 557 | 1348 | - | NCBI | 066 | abaumannii\_2 | 1 | 38 | Acinetobacter baumannii |
| sul1 | NG\_048082.1 | sulfonamide-resistant dihydropteroate synthase Sul1 | 100.00 | 100.00 | SULFONAMIDE | 60 | 899 | + | NCBI | 066 | abaumannii\_2 | 1 | 64 | Acinetobacter baumannii |
| aph(3')-VIa | NG\_047448.1 | aminoglycoside O-phosphotransferase APH(3')-VIa | 99.87 | 100.00 | AMIKACIN;KANAMYCIN | 1824 | 2603 | - | NCBI | 066 | abaumannii\_2 | 1 | 49 | Acinetobacter baumannii |
| ant(3'')-IIa | NG\_054648.1 | aminoglycoside nucleotidyltransferase ANT(3'')-IIa | 99.49 | 99.75 | SPECTINOMYCIN;STREPTOMYCIN | 34713 | 35499 | - | NCBI | 067 | abaumannii\_2 | 1526 | 10 | Acinetobacter baumannii |
| blaOXA-208 | NG\_049506.1 | OXA-51 family carbapenem-hydrolyzing class D beta-lactamase OXA-208 | 100.00 | 100.00 | CARBAPENEM | 373200 | 374024 | + | NCBI | 067 | abaumannii\_2 | 1526 | 3 | Acinetobacter baumannii |
| blaADC-166 | NG\_056108.1 | class C beta-lactamase ADC-166 | 99.83 | 100.00 | CEPHALOSPORIN | 343135 | 344286 | - | NCBI | 067 | abaumannii\_2 | 1526 | 1 | Acinetobacter baumannii |
| sul2 | NG\_051852.1 | sulfonamide-resistant dihydropteroate synthase Sul2 | 100.00 | 100.00 | SULFONAMIDE | 1570 | 2385 | + | NCBI | 068 | klebsiella | 1878 | 20 | Klebsiella pneumoniae |
| sul1 | NG\_048082.1 | sulfonamide-resistant dihydropteroate synthase Sul1 | 100.00 | 100.00 | SULFONAMIDE | 7738 | 8577 | - | NCBI | 068 | klebsiella | 1878 | 30 | Klebsiella pneumoniae |
| oqxA5 | NG\_050423.1 | multidrug efflux RND transporter periplasmic adaptor subunit OqxA5 | 99.58 | 100.00 | PHENICOL;QUINOLONE | 69337 | 70512 | - | NCBI | 068 | klebsiella | 1878 | 14 | Klebsiella pneumoniae |
| oqxB20 | NG\_050439.1 | multidrug efflux RND transporter permease subunit OqxB20 | 99.30 | 100.00 | PHENICOL;QUINOLONE | 66161 | 69313 | - | NCBI | 068 | klebsiella | 1878 | 14 | Klebsiella pneumoniae |
| fosA6 | NG\_051497.1 | fosfomycin resistance glutathione transferase FosA6 | 99.52 | 100.00 | FOSFOMYCIN | 346863 | 347282 | - | NCBI | 068 | klebsiella | 1878 | 5 | Klebsiella pneumoniae |
| blaSHV-187 | NG\_050053.1 | class A beta-lactamase SHV-187 | 99.77 | 100.00 | BETA-LACTAM | 487437 | 488303 | + | NCBI | 068 | klebsiella | 1878 | 2 | Klebsiella pneumoniae |
| aph(3'')-Ib | NG\_056002.2 | aminoglycoside O-phosphotransferase APH(3'')-Ib | 99.88 | 100.00 | STREPTOMYCIN | 2422 | 3249 | + | NCBI | 068 | klebsiella | 1878 | 20 | Klebsiella pneumoniae |
| aph(6)-Id | NG\_047466.1 | aminoglycoside O-phosphotransferase APH(6)-Id | 100.00 | 100.00 | STREPTOMYCIN | 3249 | 4085 | + | NCBI | 068 | klebsiella | 1878 | 20 | Klebsiella pneumoniae |
| blaCTX-M-15 | NG\_048935.1 | class A extended-spectrum beta-lactamase CTX-M-15 | 100.00 | 100.00 | CEPHALOSPORIN | 75325 | 76200 | - | NCBI | 068 | klebsiella | 1878 | 20 | Klebsiella pneumoniae |
| mph(A) | NG\_047986.1 | Mph(A) family macrolide 2'-phosphotransferase | 99.67 | 100.00 | MACROLIDE | 195 | 1116 | + | NCBI | 068 | klebsiella | 1878 | 30 | Klebsiella pneumoniae |
| aadA2 | NG\_047343.1 | ANT(3'')-Ia family aminoglycoside nucleotidyltransferase AadA2 | 100.00 | 100.00 | STREPTOMYCIN | 9082 | 9873 | - | NCBI | 068 | klebsiella | 1878 | 30 | Klebsiella pneumoniae |
| dfrA12 | NG\_047689.1 | trimethoprim-resistant dihydrofolate reductase DfrA12 | 100.00 | 100.00 | TRIMETHOPRIM | 10281 | 10778 | - | NCBI | 068 | klebsiella | 1878 | 30 | Klebsiella pneumoniae |
| tet(L) | NG\_048203.1 | tetracycline efflux MFS transporter Tet(L) | 99.93 | 97.53 | TETRACYCLINE | 10387 | 11729 | + | NCBI | 069 | efaecium | 117 | 62 | Enterococcus faecium |
| erm(B) | NG\_047801.1 | 23S rRNA (adenine(2058)-N(6))-methyltransferase Erm(B) | 99.60 | 99.87 | MACROLIDE | 289 | 1034 | + | NCBI | 069 | efaecium | 117 | 140 | Enterococcus faecium |
| erm(T) | NG\_047841.1 | 23S rRNA (adenine(2058)-N(6))-methyltransferase Erm(T) | 100.00 | 100.00 | MACROLIDE | 293 | 1027 | + | NCBI | 069 | efaecium | 117 | 129 | Enterococcus faecium |
| ant(6)-Ia | NG\_047393.1 | aminoglycoside nucleotidyltransferase ANT(6)-Ia | 100.00 | 100.00 | STREPTOMYCIN | 2206 | 3114 | + | NCBI | 069 | efaecium | 117 | 105 | Enterococcus faecium |
| eat(A) | NG\_047762.1 | ABC-F type ribosomal protection protein Eat(A) | 100.00 | 100.00 | PLEUROMUTILIN | 52237 | 53739 | + | NCBI | 069 | efaecium | 117 | 1 | Enterococcus faecium |
| vanS-A | NG\_048425.1 | VanA-type vancomycin resistance histidine kinase VanS | 100.00 | 100.00 | VANCOMYCIN | 852 | 2006 | + | NCBI | 069 | efaecium | 117 | 94 | Enterococcus faecium |
| vanR-A | NG\_048399.1 | VanA-type vancomycin resistance DNA-binding response regulator VanR | 100.00 | 100.00 | VANCOMYCIN | 179 | 874 | + | NCBI | 069 | efaecium | 117 | 94 | Enterococcus faecium |
| vanX-B | NG\_048483.1 | D-Ala-D-Ala dipeptidase VanX-B | 100.00 | 100.00 | VANCOMYCIN | 3927 | 4535 | + | NCBI | 069 | efaecium | 117 | 93 | Enterococcus faecium |
| msr(C) | NG\_048003.1 | ABC-F type ribosomal protection protein Msr(C) | 98.92 | 100.00 | MACROLIDE | 58754 | 60232 | - | NCBI | 069 | efaecium | 117 | 3 | Enterococcus faecium |
| dfrG | NG\_047756.1 | trimethoprim-resistant dihydrofolate reductase DfrG | 100.00 | 100.00 | TRIMETHOPRIM | 95474 | 95971 | + | NCBI | 069 | efaecium | 117 | 4 | Enterococcus faecium |
| aacA-ENT1 | NG\_052371.1 | aminoglycoside 6'-N-acetyltransferase | 100.00 | 100.00 | AMINOGLYCOSIDE | 53925 | 54473 | + | NCBI | 069 | efaecium | 117 | 9 | Enterococcus faecium |
| aph(2'')-Ih | NG\_047408.1 | aminoglycoside O-phosphotransferase APH(2'')-Ih | 82.99 | 82.21 | AMIKACIN;GENTAMICIN;KANAMYCIN;TOBRAMYCIN | 633 | 1367 | - | NCBI | 069 | efaecium | 117 | 97 | Enterococcus faecium |
| vanR-B | NG\_048410.1 | VanB-type vancomycin resistance response regulator transcription factor VanR | 99.85 | 100.00 | VANCOMYCIN | 49668 | 50330 | + | NCBI | 069 | efaecium | 117 | 16 | Enterococcus faecium |
| vanS-B | NG\_048439.1 | VanB-type vancomycin resistance histidine kinase VanS | 99.85 | 100.00 | VANCOMYCIN | 50330 | 51673 | + | NCBI | 069 | efaecium | 117 | 16 | Enterococcus faecium |
| tet(M) | NG\_048240.1 | tetracycline resistance ribosomal protection protein Tet(M) | 99.43 | 100.00 | TETRACYCLINE | 8339 | 10258 | + | NCBI | 069 | efaecium | 117 | 62 | Enterococcus faecium |
| vanB | NG\_048341.1 | D-alanine--(R)-lactate ligase VanB | 99.90 | 100.00 | VANCOMYCIN | 2893 | 3921 | + | NCBI | 069 | efaecium | 117 | 93 | Enterococcus faecium |
| vanH-B | NG\_048379.1 | D-lactate dehydrogenase VanH-B | 100.00 | 100.00 | VANCOMYCIN | 1929 | 2900 | + | NCBI | 069 | efaecium | 117 | 93 | Enterococcus faecium |
| vanW-B | NG\_048472.1 | glycopeptide resistance accessory protein VanW-B | 99.76 | 100.00 | VANCOMYCIN | 1105 | 1932 | + | NCBI | 069 | efaecium | 117 | 93 | Enterococcus faecium |
| vanY-B | NG\_048519.1 | D-Ala-D-Ala carboxypeptidase VanY-B | 100.00 | 100.00 | VANCOMYCIN | 281 | 1087 | + | NCBI | 069 | efaecium | 117 | 93 | Enterococcus faecium |
| dfrF | NG\_047755.1 | trimethoprim-resistant dihydrofolate reductase DfrF | 99.60 | 100.00 | TRIMETHOPRIM | 4158 | 4653 | + | NCBI | 069 | efaecium | 117 | 85 | Enterococcus faecium |
| vanX-A | NG\_048477.1 | D-Ala-D-Ala dipeptidase VanX-A | 100.00 | 100.00 | VANCOMYCIN | 4219 | 4827 | + | NCBI | 069 | efaecium | 117 | 94 | Enterococcus faecium |
| catA7 | NG\_047568.1 | type A-7 chloramphenicol O-acetyltransferase | 100.00 | 100.00 | CHLORAMPHENICOL | 7868 | 8515 | - | NCBI | 069 | efaecium | 117 | 70 | Enterococcus faecium |
| vanZ-A | NG\_048534.1 | glycopeptide resistance protein VanZ-A | 100.00 | 100.00 | VANCOMYCIN | 1602 | 2087 | + | NCBI | 069 | efaecium | 117 | 70 | Enterococcus faecium |
| vanY-A | NG\_048516.1 | D-Ala-D-Ala carboxypeptidase VanY-A | 100.00 | 100.00 | VANCOMYCIN | 538 | 1449 | + | NCBI | 069 | efaecium | 117 | 70 | Enterococcus faecium |
| vanA | NG\_048323.1 | D-alanine--(R)-lactate ligase VanA | 100.00 | 100.00 | VANCOMYCIN | 3182 | 4213 | + | NCBI | 069 | efaecium | 117 | 94 | Enterococcus faecium |
| vanH-A | NG\_048372.1 | D-lactate dehydrogenase VanH-A | 100.00 | 100.00 | VANCOMYCIN | 2221 | 3189 | + | NCBI | 069 | efaecium | 117 | 94 | Enterococcus faecium |
| aac(6')-Il | NG\_047303.1 | aminoglycoside N-acetyltransferase AAC(6')-Il | 100.00 | 100.00 | AMIKACIN;KANAMYCIN;TOBRAMYCIN | 2132 | 2590 | - | NCBI | 070 | paeruginosa | 233 | 57 | Pseudomonas aeruginosa |
| blaGES-1 | NG\_049111.1 | class A extended-spectrum beta-lactamase GES-1 | 99.88 | 100.00 | CEPHALOSPORIN | 803 | 1666 | + | NCBI | 070 | paeruginosa | 233 | 62 | Pseudomonas aeruginosa |
| catB7 | NG\_047614.1 | type B-4 chloramphenicol O-acetyltransferase CatB7 | 98.75 | 100.00 | CHLORAMPHENICOL | 52541 | 53179 | - | NCBI | 070 | paeruginosa | 233 | 6 | Pseudomonas aeruginosa |
| blaOXA-486 | NG\_050612.1 | OXA-50 family oxacillin-hydrolyzing class D beta-lactamase OXA-486 | 99.87 | 100.00 | BETA-LACTAM | 126598 | 127386 | + | NCBI | 070 | paeruginosa | 233 | 7 | Pseudomonas aeruginosa |
| fosA-354827590 | NG\_047883.1 | FosA family fosfomycin resistance glutathione transferase | 99.27 | 100.00 | FOSFOMYCIN | 218851 | 219258 | + | NCBI | 070 | paeruginosa | 233 | 9 | Pseudomonas aeruginosa |
| blaPDC-374 | NG\_065926.1 | class C beta-lactamase PDC-374 | 98.86 | 100.00 | CEPHALOSPORIN | 13806 | 15028 | + | NCBI | 070 | paeruginosa | 233 | 17 | Pseudomonas aeruginosa |
| aph(3')-IIb | NG\_047424.1 | aminoglycoside O-phosphotransferase APH(3')-IIb | 98.76 | 100.00 | KANAMYCIN | 27342 | 28148 | - | NCBI | 070 | paeruginosa | 233 | 17 | Pseudomonas aeruginosa |
| dfrA6 | NG\_047735.1 | trimethoprim-resistant dihydrofolate reductase DfrA6 | 99.58 | 100.00 | TRIMETHOPRIM | 116939 | 117412 | - | NCBI | 070 | paeruginosa | 233 | 19 | Pseudomonas aeruginosa |
| qnrVC1 | NG\_050551.1 | quinolone resistance pentapeptide repeat protein QnrVC1 | 100.00 | 100.00 | QUINOLONE | 118243 | 118899 | - | NCBI | 070 | paeruginosa | 233 | 19 | Pseudomonas aeruginosa |
| crpP | NG\_062203.1 | ciprofloxacin resistance protein CrpP | 98.48 | 100.00 | FLUOROQUINOLONE | 4506 | 4703 | + | NCBI | 070 | paeruginosa | 233 | 42 | Pseudomonas aeruginosa |
| blaOXA-4 | NG\_049688.1 | OXA-1 family oxacillin-hydrolyzing class D beta-lactamase OXA-4 | 100.00 | 100.00 | BETA-LACTAM | 429 | 1259 | + | NCBI | 070 | paeruginosa | 233 | 53 | Pseudomonas aeruginosa |
| aadA2 | NG\_047343.1 | ANT(3'')-Ia family aminoglycoside nucleotidyltransferase AadA2 | 99.87 | 100.00 | STREPTOMYCIN | 1372 | 2163 | + | NCBI | 070 | paeruginosa | 233 | 53 | Pseudomonas aeruginosa |
| cmlA6 | NG\_047655.1 | chloramphenicol efflux MFS transporter CmlA6 | 100.00 | 100.00 | CHLORAMPHENICOL | 2425 | 3684 | + | NCBI | 070 | paeruginosa | 233 | 53 | Pseudomonas aeruginosa |
| tet(G) | NG\_051907.1 | tetracycline efflux MFS transporter Tet(G) | 100.00 | 100.00 | TETRACYCLINE | 55 | 1230 | + | NCBI | 070 | paeruginosa | 233 | 63 | Pseudomonas aeruginosa |
| dfrB5 | NG\_047758.1 | trimethoprim-resistant dihydrofolate reductase DfrB5 | 100.00 | 100.00 | TRIMETHOPRIM | 794 | 1030 | - | NCBI | 070 | paeruginosa | 233 | 57 | Pseudomonas aeruginosa |
| aac(3)-Id | NG\_052489.1 | aminoglycoside N-acetyltransferase AAC(3)-Id | 100.00 | 100.00 | GENTAMICIN | 192 | 668 | - | NCBI | 070 | paeruginosa | 233 | 57 | Pseudomonas aeruginosa |
| aac(6')-Ib' | NG\_051695.1 | aminoglycoside N-acetyltransferase AAC(6')-Ib' | 100.00 | 100.00 | GENTAMICIN | 145 | 699 | + | NCBI | 070 | paeruginosa | 233 | 62 | Pseudomonas aeruginosa |
| floR2 | NG\_047875.1 | chloramphenicol/florfenicol efflux MFS transporter FloR2 | 100.00 | 100.00 | CHLORAMPHENICOL;FLORFENICOL | 171 | 1385 | + | NCBI | 070 | paeruginosa | 233 | 60 | Pseudomonas aeruginosa |
| blaVIM-2 | NG\_050347.1 | subclass B1 metallo-beta-lactamase VIM-2 | 100.00 | 100.00 | CARBAPENEM | 1179 | 1979 | - | NCBI | 070 | paeruginosa | 233 | 57 | Pseudomonas aeruginosa |
| blaOXA-10 | NG\_049393.1 | oxacillin-hydrolyzing class D beta-lactamase OXA-10 | 99.88 | 100.00 | CEPHALOSPORIN | 2394 | 3194 | - | NCBI | 071 | paeruginosa | 357 | 78 | Pseudomonas aeruginosa |
| aadA1 | NG\_047325.1 | ANT(3'')-Ia family aminoglycoside nucleotidyltransferase AadA1 | 99.87 | 100.00 | STREPTOMYCIN | 1586 | 2377 | - | NCBI | 071 | paeruginosa | 357 | 78 | Pseudomonas aeruginosa |
| blaOXA-846 | NG\_066519.1 | OXA-50 family oxacillin-hydrolyzing class D beta-lactamase OXA-846 | 100.00 | 100.00 | BETA-LACTAM | 24706 | 25494 | - | NCBI | 071 | paeruginosa | 357 | 54 | Pseudomonas aeruginosa |
| catB7 | NG\_047614.1 | type B-4 chloramphenicol O-acetyltransferase CatB7 | 98.59 | 100.00 | CHLORAMPHENICOL | 51855 | 52493 | - | NCBI | 071 | paeruginosa | 357 | 28 | Pseudomonas aeruginosa |
| aph(3')-IIb | NG\_047424.1 | aminoglycoside O-phosphotransferase APH(3')-IIb | 99.63 | 100.00 | KANAMYCIN | 99180 | 99986 | - | NCBI | 071 | paeruginosa | 357 | 1 | Pseudomonas aeruginosa |
| blaPDC-374 | NG\_065926.1 | class C beta-lactamase PDC-374 | 98.77 | 100.00 | CEPHALOSPORIN | 85644 | 86866 | + | NCBI | 071 | paeruginosa | 357 | 1 | Pseudomonas aeruginosa |
| aac(6')-Il | NG\_047303.1 | aminoglycoside N-acetyltransferase AAC(6')-Il | 100.00 | 100.00 | AMIKACIN;KANAMYCIN;TOBRAMYCIN | 3882 | 4340 | - | NCBI | 071 | paeruginosa | 357 | 78 | Pseudomonas aeruginosa |
| crpP | NG\_062203.1 | ciprofloxacin resistance protein CrpP | 98.48 | 100.00 | FLUOROQUINOLONE | 3258 | 3455 | + | NCBI | 071 | paeruginosa | 357 | 231 | Pseudomonas aeruginosa |
| blaVEB-9 | NG\_050333.1 | class A extended-spectrum beta-lactamase VEB-9 | 100.00 | 100.00 | CEPHALOSPORIN | 3340 | 4239 | - | NCBI | 071 | paeruginosa | 357 | 229 | Pseudomonas aeruginosa |
| tet(A) | NG\_048156.1 | tetracycline efflux MFS transporter Tet(A) | 100.00 | 96.92 | TETRACYCLINE | 1416 | 2578 | + | NCBI | 071 | paeruginosa | 357 | 229 | Pseudomonas aeruginosa |
| sul1 | NG\_048082.1 | sulfonamide-resistant dihydropteroate synthase Sul1 | 100.00 | 100.00 | SULFONAMIDE | 311 | 1150 | + | NCBI | 071 | paeruginosa | 357 | 195 | Pseudomonas aeruginosa |
| fosA-354827590 | NG\_047883.1 | FosA family fosfomycin resistance glutathione transferase | 99.75 | 100.00 | FOSFOMYCIN | 7231 | 7638 | + | NCBI | 071 | paeruginosa | 357 | 140 | Pseudomonas aeruginosa |
| aac(6')-Il | NG\_047303.1 | aminoglycoside N-acetyltransferase AAC(6')-Il | 100.00 | 100.00 | AMIKACIN;KANAMYCIN;TOBRAMYCIN | 5064 | 5522 | - | NCBI | 071 | paeruginosa | 357 | 78 | Pseudomonas aeruginosa |
| ant(2'')-Ia | NG\_047387.1 | aminoglycoside nucleotidyltransferase ANT(2'')-Ia | 100.00 | 100.00 | GENTAMICIN;KANAMYCIN;TOBRAMYCIN | 4399 | 4932 | - | NCBI | 071 | paeruginosa | 357 | 78 | Pseudomonas aeruginosa |
| dfrB2 | NG\_047746.1 | trimethoprim-resistant dihydrofolate reductase DfrB2 | 100.00 | 100.00 | TRIMETHOPRIM | 3421 | 3657 | - | NCBI | 071 | paeruginosa | 357 | 78 | Pseudomonas aeruginosa |
| aac(6')-Ib-D181Y | NG\_067946.1 | AAC(6')-Ib family aminoglycoside 6'-N-acetyltransferase | 99.82 | 100.00 | AMIKACIN;KANAMYCIN;TOBRAMYCIN | 1162 | 1716 | - | NCBI | 072 | ecoli\_achtman\_4 | 167 | 67 | Escherichia coli |
| mph(A) | NG\_047986.1 | Mph(A) family macrolide 2'-phosphotransferase | 99.67 | 100.00 | MACROLIDE | 2887 | 3808 | - | NCBI | 072 | ecoli\_achtman\_4 | 167 | 62 | Escherichia coli |
| blaCTX-M-15 | NG\_048935.1 | class A extended-spectrum beta-lactamase CTX-M-15 | 100.00 | 100.00 | CEPHALOSPORIN | 2636 | 3511 | - | NCBI | 072 | ecoli\_achtman\_4 | 167 | 61 | Escherichia coli |
| aph(6)-Id | NG\_047465.1 | aminoglycoside O-phosphotransferase APH(6)-Id | 100.00 | 82.44 | STREPTOMYCIN | 1 | 690 | - | NCBI | 072 | ecoli\_achtman\_4 | 167 | 57 | Escherichia coli |
| aph(3'')-Ib | NG\_056002.2 | aminoglycoside O-phosphotransferase APH(3'')-Ib | 100.00 | 100.00 | STREPTOMYCIN | 690 | 1517 | - | NCBI | 072 | ecoli\_achtman\_4 | 167 | 57 | Escherichia coli |
| sul2 | NG\_051852.1 | sulfonamide-resistant dihydropteroate synthase Sul2 | 100.00 | 100.00 | SULFONAMIDE | 1554 | 2369 | - | NCBI | 072 | ecoli\_achtman\_4 | 167 | 57 | Escherichia coli |
| blaEC-15 | NG\_049081.1 | class C extended-spectrum beta-lactamase EC-15 | 98.50 | 100.00 | CEPHALOSPORIN | 111639 | 112772 | - | NCBI | 072 | ecoli\_achtman\_4 | 167 | 3 | Escherichia coli |
| dfrA12 | NG\_047689.1 | trimethoprim-resistant dihydrofolate reductase DfrA12 | 100.00 | 100.00 | TRIMETHOPRIM | 1140 | 1637 | + | NCBI | 072 | ecoli\_achtman\_4 | 167 | 50 | Escherichia coli |
| tet(A) | NG\_048154.1 | tetracycline efflux MFS transporter Tet(A) | 100.00 | 100.00 | TETRACYCLINE | 10680 | 11879 | - | NCBI | 072 | ecoli\_achtman\_4 | 167 | 46 | Escherichia coli |
| sul1 | NG\_048082.1 | sulfonamide-resistant dihydropteroate synthase Sul1 | 100.00 | 100.00 | SULFONAMIDE | 3341 | 4180 | + | NCBI | 072 | ecoli\_achtman\_4 | 167 | 50 | Escherichia coli |
| ble-MBL | NG\_047559.1 | bleomycin binding protein Ble-MBL | 100.00 | 100.00 | BLEOMYCIN | 8124 | 8489 | - | NCBI | 072 | ecoli\_achtman\_4 | 167 | 50 | Escherichia coli |
| blaNDM-5 | NG\_049337.1 | subclass B1 metallo-beta-lactamase NDM-5 | 100.00 | 100.00 | CARBAPENEM | 8493 | 9305 | - | NCBI | 072 | ecoli\_achtman\_4 | 167 | 50 | Escherichia coli |
| aadA2 | NG\_047343.1 | ANT(3'')-Ia family aminoglycoside nucleotidyltransferase AadA2 | 100.00 | 100.00 | STREPTOMYCIN | 2045 | 2836 | + | NCBI | 072 | ecoli\_achtman\_4 | 167 | 50 | Escherichia coli |
| sul1 | NG\_048082.1 | sulfonamide-resistant dihydropteroate synthase Sul1 | 100.00 | 100.00 | SULFONAMIDE | 494 | 1333 | + | NCBI | 073 | abaumannii\_2 | 2 | 51 | Acinetobacter baumannii |
| blaADC-73 | NG\_048678.1 | class C extended-spectrum beta-lactamase ADC-73 | 100.00 | 100.00 | CEPHALOSPORIN | 95963 | 97114 | - | NCBI | 073 | abaumannii\_2 | 2 | 17 | Acinetobacter baumannii |
| msr(E) | NG\_048007.1 | ABC-F type ribosomal protection protein Msr(E) | 100.00 | 100.00 | MACROLIDE | 5203 | 6678 | - | NCBI | 073 | abaumannii\_2 | 2 | 9 | Acinetobacter baumannii |
| mph(E) | NG\_064660.1 | Mph(E) family macrolide 2'-phosphotransferase | 100.00 | 100.00 | MACROLIDE | 4263 | 5147 | - | NCBI | 073 | abaumannii\_2 | 2 | 9 | Acinetobacter baumannii |
| ant(3'')-IIa | NG\_054646.1 | aminoglycoside nucleotidyltransferase ANT(3'')-IIa | 98.61 | 100.00 | SPECTINOMYCIN;STREPTOMYCIN | 35813 | 36601 | - | NCBI | 073 | abaumannii\_2 | 2 | 7 | Acinetobacter baumannii |
| blaOXA-66 | NG\_049806.1 | OXA-51 family carbapenem-hydrolyzing class D beta-lactamase OXA-66 | 100.00 | 100.00 | CARBAPENEM | 292426 | 293250 | - | NCBI | 073 | abaumannii\_2 | 2 | 1 | Acinetobacter baumannii |
| aph(3'')-Ib | NG\_056002.2 | aminoglycoside O-phosphotransferase APH(3'')-Ib | 99.88 | 98.31 | STREPTOMYCIN | 59300 | 60113 | + | NCBI | 073 | abaumannii\_2 | 2 | 23 | Acinetobacter baumannii |
| aph(6)-Id | NG\_047464.1 | aminoglycoside O-phosphotransferase APH(6)-Id | 100.00 | 100.00 | STREPTOMYCIN | 60113 | 60949 | + | NCBI | 073 | abaumannii\_2 | 2 | 23 | Acinetobacter baumannii |
| tet(B) | NG\_048161.1 | tetracycline efflux MFS transporter Tet(B) | 100.00 | 99.50 | TETRACYCLINE | 64322 | 65521 | + | NCBI | 073 | abaumannii\_2 | 2 | 23 | Acinetobacter baumannii |
| catA1 | NG\_047582.1 | type A-1 chloramphenicol O-acetyltransferase | 99.85 | 100.00 | CHLORAMPHENICOL | 2751 | 3410 | - | NCBI | 073 | abaumannii\_2 | 2 | 48 | Acinetobacter baumannii |
| armA | NG\_047476.1 | ArmA family 16S rRNA (guanine(1405)-N(7))-methyltransferase | 100.00 | 100.00 | GENTAMICIN | 8977 | 9750 | - | NCBI | 073 | abaumannii\_2 | 2 | 9 | Acinetobacter baumannii |
| blaOXA-23 | NG\_049525.1 | carbapenem-hydrolyzing class D beta-lactamase OXA-23 | 100.00 | 100.00 | CARBAPENEM | 145 | 966 | + | NCBI | 073 | abaumannii\_2 | 2 | 53 | Acinetobacter baumannii |
| blaOXA-66 | NG\_049806.1 | OXA-51 family carbapenem-hydrolyzing class D beta-lactamase OXA-66 | 100.00 | 100.00 | CARBAPENEM | 315022 | 315846 | - | NCBI | 074 | abaumannii\_2 | 2 | 1 | Acinetobacter baumannii |
| catA1 | NG\_047582.1 | type A-1 chloramphenicol O-acetyltransferase | 99.85 | 100.00 | CHLORAMPHENICOL | 4070 | 4729 | + | NCBI | 074 | abaumannii\_2 | 2 | 47 | Acinetobacter baumannii |
| blaADC-73 | NG\_048678.1 | class C extended-spectrum beta-lactamase ADC-73 | 100.00 | 100.00 | CEPHALOSPORIN | 18309 | 19460 | - | NCBI | 074 | abaumannii\_2 | 2 | 39 | Acinetobacter baumannii |
| mph(E) | NG\_064660.1 | Mph(E) family macrolide 2'-phosphotransferase | 100.00 | 100.00 | MACROLIDE | 38438 | 39322 | + | NCBI | 074 | abaumannii\_2 | 2 | 28 | Acinetobacter baumannii |
| msr(E) | NG\_048007.1 | ABC-F type ribosomal protection protein Msr(E) | 100.00 | 100.00 | MACROLIDE | 36907 | 38382 | + | NCBI | 074 | abaumannii\_2 | 2 | 28 | Acinetobacter baumannii |
| armA | NG\_047476.1 | ArmA family 16S rRNA (guanine(1405)-N(7))-methyltransferase | 100.00 | 100.00 | GENTAMICIN | 33835 | 34608 | + | NCBI | 074 | abaumannii\_2 | 2 | 28 | Acinetobacter baumannii |
| aph(3'')-Ib | NG\_056002.2 | aminoglycoside O-phosphotransferase APH(3'')-Ib | 99.88 | 98.31 | STREPTOMYCIN | 7736 | 8549 | - | NCBI | 074 | abaumannii\_2 | 2 | 21 | Acinetobacter baumannii |
| aph(6)-Id | NG\_047464.1 | aminoglycoside O-phosphotransferase APH(6)-Id | 100.00 | 100.00 | STREPTOMYCIN | 6900 | 7736 | - | NCBI | 074 | abaumannii\_2 | 2 | 21 | Acinetobacter baumannii |
| tet(B) | NG\_048161.1 | tetracycline efflux MFS transporter Tet(B) | 100.00 | 99.50 | TETRACYCLINE | 2328 | 3527 | - | NCBI | 074 | abaumannii\_2 | 2 | 21 | Acinetobacter baumannii |
| aph(3')-VIa | NG\_047448.1 | aminoglycoside O-phosphotransferase APH(3')-VIa | 99.87 | 100.00 | AMIKACIN;KANAMYCIN | 9687 | 10466 | + | NCBI | 074 | abaumannii\_2 | 2 | 20 | Acinetobacter baumannii |
| ant(3'')-IIa | NG\_054646.1 | aminoglycoside nucleotidyltransferase ANT(3'')-IIa | 98.61 | 100.00 | SPECTINOMYCIN;STREPTOMYCIN | 1029 | 1817 | - | NCBI | 074 | abaumannii\_2 | 2 | 11 | Acinetobacter baumannii |
| sul1 | NG\_048082.1 | sulfonamide-resistant dihydropteroate synthase Sul1 | 100.00 | 100.00 | SULFONAMIDE | 2045 | 2884 | - | NCBI | 074 | abaumannii\_2 | 2 | 51 | Acinetobacter baumannii |
| blaOXA-23 | NG\_049525.1 | carbapenem-hydrolyzing class D beta-lactamase OXA-23 | 100.00 | 100.00 | CARBAPENEM | 1680 | 2501 | - | NCBI | 074 | abaumannii\_2 | 2 | 53 | Acinetobacter baumannii |
| fosA-354827590 | NG\_047883.1 | FosA family fosfomycin resistance glutathione transferase | 99.02 | 100.00 | FOSFOMYCIN | 112401 | 112808 | - | NCBI | 075 | paeruginosa | - | 6 | Pseudomonas aeruginosa |
| blaOXA-847 | NG\_066520.1 | OXA-50 family oxacillin-hydrolyzing class D beta-lactamase OXA-847 | 100.00 | 100.00 | BETA-LACTAM | 126476 | 127264 | + | NCBI | 075 | paeruginosa | - | 8 | Pseudomonas aeruginosa |
| aph(3')-IIb | NG\_047424.1 | aminoglycoside O-phosphotransferase APH(3')-IIb | 98.89 | 100.00 | KANAMYCIN | 25389 | 26195 | + | NCBI | 075 | paeruginosa | - | 39 | Pseudomonas aeruginosa |
| cmlA6 | NG\_047655.1 | chloramphenicol efflux MFS transporter CmlA6 | 100.00 | 100.00 | CHLORAMPHENICOL | 4361 | 5620 | + | NCBI | 075 | paeruginosa | - | 64 | Pseudomonas aeruginosa |
| blaPDC-374 | NG\_065926.1 | class C beta-lactamase PDC-374 | 98.94 | 100.00 | CEPHALOSPORIN | 38522 | 39744 | - | NCBI | 075 | paeruginosa | - | 39 | Pseudomonas aeruginosa |
| aadA2 | NG\_051846.1 | ANT(3'')-Ia family aminoglycoside nucleotidyltransferase AadA2 | 100.00 | 100.00 | STREPTOMYCIN | 3308 | 4099 | + | NCBI | 075 | paeruginosa | - | 64 | Pseudomonas aeruginosa |
| mph(E) | NG\_064660.1 | Mph(E) family macrolide 2'-phosphotransferase | 100.00 | 100.00 | MACROLIDE | 2056 | 2940 | + | NCBI | 075 | paeruginosa | - | 64 | Pseudomonas aeruginosa |
| msr(E) | NG\_048007.1 | ABC-F type ribosomal protection protein Msr(E) | 100.00 | 100.00 | MACROLIDE | 525 | 2000 | + | NCBI | 075 | paeruginosa | - | 64 | Pseudomonas aeruginosa |
| mph(F) | NG\_047997.1 | Mph(F) family macrolide 2'-phosphotransferase | 100.00 | 100.00 | MACROLIDE | 946 | 1845 | + | NCBI | 075 | paeruginosa | - | 58 | Pseudomonas aeruginosa |
| catB7 | NG\_047614.1 | type B-4 chloramphenicol O-acetyltransferase CatB7 | 99.84 | 100.00 | CHLORAMPHENICOL | 52578 | 53216 | - | NCBI | 075 | paeruginosa | - | 3 | Pseudomonas aeruginosa |
| floR2 | NG\_047875.1 | chloramphenicol/florfenicol efflux MFS transporter FloR2 | 100.00 | 100.00 | CHLORAMPHENICOL;FLORFENICOL | 787 | 2001 | + | NCBI | 075 | paeruginosa | - | 59 | Pseudomonas aeruginosa |
| tet(G) | NG\_051907.1 | tetracycline efflux MFS transporter Tet(G) | 100.00 | 100.00 | TETRACYCLINE | 2938 | 4113 | + | NCBI | 075 | paeruginosa | - | 59 | Pseudomonas aeruginosa |
| aadA2 | NG\_047343.1 | ANT(3'')-Ia family aminoglycoside nucleotidyltransferase AadA2 | 100.00 | 100.00 | STREPTOMYCIN | 3154 | 3945 | + | NCBI | 076 | klebsiella | 1412 | 30 | Klebsiella pneumoniae |
| mph(A) | NG\_047986.1 | Mph(A) family macrolide 2'-phosphotransferase | 99.67 | 100.00 | MACROLIDE | 10584 | 11505 | - | NCBI | 076 | klebsiella | 1412 | 30 | Klebsiella pneumoniae |
| aph(3'')-Ib | NG\_056002.2 | aminoglycoside O-phosphotransferase APH(3'')-Ib | 99.88 | 100.00 | STREPTOMYCIN | 2422 | 3249 | + | NCBI | 076 | klebsiella | 1412 | 31 | Klebsiella pneumoniae |
| aph(6)-Id | NG\_047466.1 | aminoglycoside O-phosphotransferase APH(6)-Id | 100.00 | 100.00 | STREPTOMYCIN | 3249 | 4085 | + | NCBI | 076 | klebsiella | 1412 | 31 | Klebsiella pneumoniae |
| qnrS1 | NG\_050543.1 | quinolone resistance pentapeptide repeat protein QnrS1 | 100.00 | 100.00 | QUINOLONE | 8326 | 8982 | - | NCBI | 076 | klebsiella | 1412 | 31 | Klebsiella pneumoniae |
| blaCTX-M-15 | NG\_048935.1 | class A extended-spectrum beta-lactamase CTX-M-15 | 100.00 | 100.00 | CEPHALOSPORIN | 2763 | 3638 | - | NCBI | 076 | klebsiella | 1412 | 36 | Klebsiella pneumoniae |
| aph(3')-Ia | NG\_047430.1 | aminoglycoside O-phosphotransferase APH(3')-Ia | 100.00 | 100.00 | KANAMYCIN | 265 | 1080 | - | NCBI | 076 | klebsiella | 1412 | 49 | Klebsiella pneumoniae |
| sul1 | NG\_048082.1 | sulfonamide-resistant dihydropteroate synthase Sul1 | 100.00 | 100.00 | SULFONAMIDE | 4450 | 5289 | + | NCBI | 076 | klebsiella | 1412 | 30 | Klebsiella pneumoniae |
| dfrA12 | NG\_047689.1 | trimethoprim-resistant dihydrofolate reductase DfrA12 | 100.00 | 100.00 | TRIMETHOPRIM | 2249 | 2746 | + | NCBI | 076 | klebsiella | 1412 | 30 | Klebsiella pneumoniae |
| sul2 | NG\_051852.1 | sulfonamide-resistant dihydropteroate synthase Sul2 | 99.88 | 100.00 | SULFONAMIDE | 1570 | 2385 | + | NCBI | 076 | klebsiella | 1412 | 31 | Klebsiella pneumoniae |
| oqxA6 | NG\_050424.1 | multidrug efflux RND transporter periplasmic adaptor subunit OqxA6 | 99.58 | 100.00 | PHENICOL;QUINOLONE | 30790 | 31965 | + | NCBI | 076 | klebsiella | 1412 | 4 | Klebsiella pneumoniae |
| blaSHV-145 | NG\_050019.1 | class A beta-lactamase SHV-145 | 99.88 | 100.00 | BETA-LACTAM | 555019 | 555879 | + | NCBI | 076 | klebsiella | 1412 | 2 | Klebsiella pneumoniae |
| fosA5 | NG\_050409.1 | fosfomycin resistance glutathione transferase FosA5 | 95.43 | 99.05 | FOSFOMYCIN | 14230 | 14645 | + | NCBI | 076 | klebsiella | 1412 | 7 | Klebsiella pneumoniae |
| oqxB12 | NG\_050430.1 | multidrug efflux RND transporter permease subunit OqxB12 | 99.37 | 100.00 | PHENICOL;QUINOLONE | 31989 | 35141 | + | NCBI | 076 | klebsiella | 1412 | 4 | Klebsiella pneumoniae |
| blaPDC-374 | NG\_065926.1 | class C beta-lactamase PDC-374 | 98.69 | 100.00 | CEPHALOSPORIN | 148151 | 149373 | + | NCBI | 077 | paeruginosa | 620 | 10 | Pseudomonas aeruginosa |
| catB7 | NG\_047614.1 | type B-4 chloramphenicol O-acetyltransferase CatB7 | 98.44 | 99.84 | CHLORAMPHENICOL | 334091 | 334728 | + | NCBI | 077 | paeruginosa | 620 | 7 | Pseudomonas aeruginosa |
| blaGES-1 | NG\_049111.1 | class A extended-spectrum beta-lactamase GES-1 | 99.88 | 100.00 | CEPHALOSPORIN | 43801 | 44664 | - | NCBI | 077 | paeruginosa | 620 | 29 | Pseudomonas aeruginosa |
| fosA-354827590 | NG\_047883.1 | FosA family fosfomycin resistance glutathione transferase | 99.27 | 100.00 | FOSFOMYCIN | 425086 | 425493 | - | NCBI | 077 | paeruginosa | 620 | 2 | Pseudomonas aeruginosa |
| aac(6')-Ib' | NG\_051695.1 | aminoglycoside N-acetyltransferase AAC(6')-Ib' | 100.00 | 100.00 | GENTAMICIN | 44768 | 45322 | - | NCBI | 077 | paeruginosa | 620 | 29 | Pseudomonas aeruginosa |
| aph(3')-IIb | NG\_047423.1 | aminoglycoside O-phosphotransferase APH(3')-IIb | 99.26 | 100.00 | KANAMYCIN | 161603 | 162409 | - | NCBI | 077 | paeruginosa | 620 | 10 | Pseudomonas aeruginosa |
| blaOXA-395 | NG\_049684.1 | OXA-50 family oxacillin-hydrolyzing class D beta-lactamase OXA-395 | 99.37 | 100.00 | BETA-LACTAM | 162309 | 163097 | + | NCBI | 077 | paeruginosa | 620 | 11 | Pseudomonas aeruginosa |
| aph(3')-IIb | NG\_047424.1 | aminoglycoside O-phosphotransferase APH(3')-IIb | 99.50 | 100.00 | KANAMYCIN | 26028 | 26834 | + | NCBI | 078 | paeruginosa | 399 | 19 | Pseudomonas aeruginosa |
| catB7 | NG\_047614.1 | type B-4 chloramphenicol O-acetyltransferase CatB7 | 99.84 | 100.00 | CHLORAMPHENICOL | 104840 | 105478 | + | NCBI | 078 | paeruginosa | 399 | 18 | Pseudomonas aeruginosa |
| crpP | NG\_062203.1 | ciprofloxacin resistance protein CrpP | 96.47 | 100.00 | FLUOROQUINOLONE | 95569 | 95766 | + | NCBI | 078 | paeruginosa | 399 | 4 | Pseudomonas aeruginosa |
| blaOXA-905 | NG\_068024.1 | OXA-50 family oxacillin-hydrolyzing class D beta-lactamase OXA-905 | 99.75 | 100.00 | BETA-LACTAM | 146064 | 146852 | - | NCBI | 078 | paeruginosa | 399 | 11 | Pseudomonas aeruginosa |
| blaPDC-55 | NG\_049929.1 | class C beta-lactamase PDC-55 | 99.02 | 100.00 | CEPHALOSPORIN | 39148 | 40370 | - | NCBI | 078 | paeruginosa | 399 | 19 | Pseudomonas aeruginosa |
| fosA-354827590 | NG\_047883.1 | FosA family fosfomycin resistance glutathione transferase | 99.27 | 100.00 | FOSFOMYCIN | 315374 | 315781 | + | NCBI | 078 | paeruginosa | 399 | 5 | Pseudomonas aeruginosa |
| oqxA10 | NG\_050418.1 | multidrug efflux RND transporter periplasmic adaptor subunit OqxA10 | 86.58 | 99.91 | PHENICOL;QUINOLONE | 91523 | 92698 | + | NCBI | 079 | ecloacae | 102 | 14 | Enterobacter cloacae |
| fosA | NG\_050405.1 | fosfomycin resistance glutathione transferase FosA | 97.18 | 100.00 | FOSFOMYCIN | 149495 | 149920 | - | NCBI | 079 | ecloacae | 102 | 9 | Enterobacter cloacae |
| oqxB5 | NG\_050454.1 | multidrug efflux RND transporter permease subunit OqxB5 | 89.06 | 100.00 | PHENICOL;QUINOLONE | 92722 | 95874 | + | NCBI | 079 | ecloacae | 102 | 14 | Enterobacter cloacae |
| blaACT-23 | NG\_048610.1 | cephalosporin-hydrolyzing class C beta-lactamase ACT-23 | 99.30 | 100.00 | CEPHALOSPORIN | 126653 | 127798 | + | NCBI | 079 | ecloacae | 102 | 11 | Enterobacter cloacae |
| erm(T) | NG\_047841.1 | 23S rRNA (adenine(2058)-N(6))-methyltransferase Erm(T) | 100.00 | 100.00 | MACROLIDE | 293 | 1027 | + | NCBI | 080 | efaecium | 1283 | 138 | Enterococcus faecium |
| vanR-A | NG\_048399.1 | VanA-type vancomycin resistance DNA-binding response regulator VanR | 100.00 | 100.00 | VANCOMYCIN | 4106 | 4801 | - | NCBI | 080 | efaecium | 1283 | 97 | Enterococcus faecium |
| erm(B) | NG\_047801.1 | 23S rRNA (adenine(2058)-N(6))-methyltransferase Erm(B) | 99.60 | 99.87 | MACROLIDE | 290 | 1035 | - | NCBI | 080 | efaecium | 1283 | 166 | Enterococcus faecium |
| sat4 | NG\_048070.1 | streptothricin N-acetyltransferase Sat4 | 100.00 | 81.22 | STREPTOTHRICIN | 1 | 441 | + | NCBI | 080 | efaecium | 1283 | 107 | Enterococcus faecium |
| vanA | NG\_048323.1 | D-alanine--(R)-lactate ligase VanA | 100.00 | 100.00 | VANCOMYCIN | 767 | 1798 | - | NCBI | 080 | efaecium | 1283 | 97 | Enterococcus faecium |
| vanS-A | NG\_048425.1 | VanA-type vancomycin resistance histidine kinase VanS | 100.00 | 100.00 | VANCOMYCIN | 2974 | 4128 | - | NCBI | 080 | efaecium | 1283 | 97 | Enterococcus faecium |
| aph(2'')-Ih | NG\_047408.1 | aminoglycoside O-phosphotransferase APH(2'')-Ih | 82.43 | 89.15 | AMIKACIN;GENTAMICIN;KANAMYCIN;TOBRAMYCIN | 527 | 1323 | + | NCBI | 080 | efaecium | 1283 | 155 | Enterococcus faecium |
| vanH-A | NG\_048372.1 | D-lactate dehydrogenase VanH-A | 100.00 | 100.00 | VANCOMYCIN | 1791 | 2759 | - | NCBI | 080 | efaecium | 1283 | 97 | Enterococcus faecium |
| aph(3')-IIIa | NG\_047418.1 | aminoglycoside O-phosphotransferase APH(3')-IIIa | 100.00 | 100.00 | AMIKACIN;KANAMYCIN | 534 | 1328 | + | NCBI | 080 | efaecium | 1283 | 107 | Enterococcus faecium |
| vanY-A | NG\_048516.1 | D-Ala-D-Ala carboxypeptidase VanY-A | 100.00 | 100.00 | VANCOMYCIN | 9710 | 10621 | - | NCBI | 080 | efaecium | 1283 | 69 | Enterococcus faecium |
| vanX-A | NG\_048477.1 | D-Ala-D-Ala dipeptidase VanX-A | 100.00 | 100.00 | VANCOMYCIN | 153 | 761 | - | NCBI | 080 | efaecium | 1283 | 97 | Enterococcus faecium |
| msr(C) | NG\_048003.1 | ABC-F type ribosomal protection protein Msr(C) | 98.99 | 100.00 | MACROLIDE | 81762 | 83240 | - | NCBI | 080 | efaecium | 1283 | 1 | Enterococcus faecium |
| dfrG | NG\_047756.1 | trimethoprim-resistant dihydrofolate reductase DfrG | 100.00 | 100.00 | TRIMETHOPRIM | 147484 | 147981 | + | NCBI | 080 | efaecium | 1283 | 2 | Enterococcus faecium |
| eat(A) | NG\_047762.1 | ABC-F type ribosomal protection protein Eat(A) | 93.88 | 100.00 | PLEUROMUTILIN | 31579 | 33081 | - | NCBI | 080 | efaecium | 1283 | 5 | Enterococcus faecium |
| tet(L) | NG\_048203.1 | tetracycline efflux MFS transporter Tet(L) | 99.93 | 97.53 | TETRACYCLINE | 1960 | 3302 | - | NCBI | 080 | efaecium | 1283 | 27 | Enterococcus faecium |
| aacA-ENT1 | NG\_052371.1 | aminoglycoside 6'-N-acetyltransferase | 100.00 | 100.00 | AMINOGLYCOSIDE | 11648 | 12196 | - | NCBI | 080 | efaecium | 1283 | 35 | Enterococcus faecium |
| tet(M) | NG\_048240.1 | tetracycline resistance ribosomal protection protein Tet(M) | 99.43 | 100.00 | TETRACYCLINE | 3431 | 5350 | - | NCBI | 080 | efaecium | 1283 | 27 | Enterococcus faecium |
| ant(6)-Ia | NG\_047393.1 | aminoglycoside nucleotidyltransferase ANT(6)-Ia | 100.00 | 100.00 | STREPTOMYCIN | 823 | 1731 | + | NCBI | 080 | efaecium | 1283 | 134 | Enterococcus faecium |
| catA7 | NG\_047568.1 | type A-7 chloramphenicol O-acetyltransferase | 100.00 | 100.00 | CHLORAMPHENICOL | 2644 | 3291 | + | NCBI | 080 | efaecium | 1283 | 69 | Enterococcus faecium |
| vanZ-A | NG\_048534.1 | glycopeptide resistance protein VanZ-A | 100.00 | 100.00 | VANCOMYCIN | 9072 | 9557 | - | NCBI | 080 | efaecium | 1283 | 69 | Enterococcus faecium |
| aac(6')-Ib-D181Y | NG\_067946.1 | AAC(6')-Ib family aminoglycoside 6'-N-acetyltransferase | 99.82 | 100.00 | AMIKACIN;KANAMYCIN;TOBRAMYCIN | 219 | 773 | + | NCBI | 081 | klebsiella | 101 | 58 | Klebsiella pneumoniae |
| blaSHV-212 | NG\_062284.1 | class A beta-lactamase SHV-212 | 99.88 | 100.00 | BETA-LACTAM | 27985 | 28845 | - | NCBI | 081 | klebsiella | 101 | 14 | Klebsiella pneumoniae |
| blaOXA-1 | NG\_049392.1 | oxacillin-hydrolyzing class D beta-lactamase OXA-1 | 100.00 | 100.00 | CEPHALOSPORIN | 904 | 1734 | + | NCBI | 081 | klebsiella | 101 | 58 | Klebsiella pneumoniae |
| blaOXA-48 | NG\_049762.1 | carbapenem-hydrolyzing class D beta-lactamase OXA-48 | 100.00 | 100.00 | CARBAPENEM | 1409 | 2206 | - | NCBI | 081 | klebsiella | 101 | 60 | Klebsiella pneumoniae |
| aph(3'')-Ib | NG\_056002.2 | aminoglycoside O-phosphotransferase APH(3'')-Ib | 100.00 | 100.00 | STREPTOMYCIN | 962 | 1789 | - | NCBI | 081 | klebsiella | 101 | 56 | Klebsiella pneumoniae |
| tet(D) | NG\_048184.1 | tetracycline efflux MFS transporter Tet(D) | 99.92 | 100.00 | TETRACYCLINE | 1801 | 2985 | - | NCBI | 081 | klebsiella | 101 | 52 | Klebsiella pneumoniae |
| dfrA14 | NG\_056035.1 | trimethoprim-resistant dihydrofolate reductase DfrA14 | 100.00 | 100.00 | TRIMETHOPRIM | 1968 | 2441 | + | NCBI | 081 | klebsiella | 101 | 48 | Klebsiella pneumoniae |
| sul2 | NG\_051852.1 | sulfonamide-resistant dihydropteroate synthase Sul2 | 100.00 | 100.00 | SULFONAMIDE | 1826 | 2641 | - | NCBI | 081 | klebsiella | 101 | 56 | Klebsiella pneumoniae |
| fosA\_gen | NG\_047881.1 | FosA family fosfomycin resistance glutathione transferase | 96.43 | 100.00 | FOSFOMYCIN | 335995 | 336414 | - | NCBI | 081 | klebsiella | 101 | 5 | Klebsiella pneumoniae |
| aph(6)-Id | NG\_047464.1 | aminoglycoside O-phosphotransferase APH(6)-Id | 100.00 | 100.00 | STREPTOMYCIN | 126 | 962 | - | NCBI | 081 | klebsiella | 101 | 56 | Klebsiella pneumoniae |
| oqxA10 | NG\_050418.1 | multidrug efflux RND transporter periplasmic adaptor subunit OqxA10 | 99.75 | 100.00 | PHENICOL;QUINOLONE | 87603 | 88778 | + | NCBI | 081 | klebsiella | 101 | 1 | Klebsiella pneumoniae |
| oqxB17 | NG\_050435.1 | multidrug efflux RND transporter permease subunit OqxB17 | 99.62 | 100.00 | PHENICOL;QUINOLONE | 88802 | 91954 | + | NCBI | 081 | klebsiella | 101 | 1 | Klebsiella pneumoniae |
| aph(2'')-Ih | NG\_047408.1 | aminoglycoside O-phosphotransferase APH(2'')-Ih | 82.99 | 82.21 | AMIKACIN;GENTAMICIN;KANAMYCIN;TOBRAMYCIN | 294 | 1028 | + | NCBI | 082 | efaecium | 203 | 276 | Enterococcus faecium |
| erm(B) | NG\_047801.1 | 23S rRNA (adenine(2058)-N(6))-methyltransferase Erm(B) | 99.60 | 99.87 | MACROLIDE | 183 | 928 | + | NCBI | 082 | efaecium | 203 | 262 | Enterococcus faecium |
| vanA | NG\_048323.1 | D-alanine--(R)-lactate ligase VanA | 100.00 | 100.00 | VANCOMYCIN | 490 | 1521 | - | NCBI | 082 | efaecium | 203 | 240 | Enterococcus faecium |
| catA7 | NG\_047568.1 | type A-7 chloramphenicol O-acetyltransferase | 100.00 | 100.00 | CHLORAMPHENICOL | 1210 | 1857 | + | NCBI | 082 | efaecium | 203 | 239 | Enterococcus faecium |
| vanR-A | NG\_048399.1 | VanA-type vancomycin resistance DNA-binding response regulator VanR | 100.00 | 100.00 | VANCOMYCIN | 1895 | 2590 | - | NCBI | 082 | efaecium | 203 | 219 | Enterococcus faecium |
| vanS-A | NG\_048425.1 | VanA-type vancomycin resistance histidine kinase VanS | 100.00 | 100.00 | VANCOMYCIN | 763 | 1917 | - | NCBI | 082 | efaecium | 203 | 219 | Enterococcus faecium |
| ant(6)-Ia | NG\_047393.1 | aminoglycoside nucleotidyltransferase ANT(6)-Ia | 100.00 | 100.00 | STREPTOMYCIN | 532 | 1440 | - | NCBI | 082 | efaecium | 203 | 207 | Enterococcus faecium |
| vanY-A | NG\_048516.1 | D-Ala-D-Ala carboxypeptidase VanY-A | 100.00 | 100.00 | VANCOMYCIN | 6287 | 7198 | - | NCBI | 082 | efaecium | 203 | 142 | Enterococcus faecium |
| vanZ-A | NG\_048534.1 | glycopeptide resistance protein VanZ-A | 100.00 | 100.00 | VANCOMYCIN | 5649 | 6134 | - | NCBI | 082 | efaecium | 203 | 142 | Enterococcus faecium |
| aacA-ENT1 | NG\_052371.1 | aminoglycoside 6'-N-acetyltransferase | 100.00 | 100.00 | AMINOGLYCOSIDE | 23 | 571 | - | NCBI | 082 | efaecium | 203 | 134 | Enterococcus faecium |
| dfrF | NG\_047755.1 | trimethoprim-resistant dihydrofolate reductase DfrF | 99.60 | 100.00 | TRIMETHOPRIM | 6360 | 6855 | + | NCBI | 082 | efaecium | 203 | 115 | Enterococcus faecium |
| msr(C) | NG\_048003.1 | ABC-F type ribosomal protection protein Msr(C) | 98.92 | 100.00 | MACROLIDE | 10853 | 12331 | + | NCBI | 082 | efaecium | 203 | 66 | Enterococcus faecium |
| eat(A) | NG\_047762.1 | ABC-F type ribosomal protection protein Eat(A) | 100.00 | 100.00 | PLEUROMUTILIN | 5826 | 7328 | + | NCBI | 082 | efaecium | 203 | 34 | Enterococcus faecium |
| tet(M) | NG\_048213.1 | tetracycline resistance ribosomal protection protein Tet(M) | 99.79 | 100.00 | TETRACYCLINE | 19070 | 20989 | + | NCBI | 082 | efaecium | 203 | 7 | Enterococcus faecium |
| blaSHV-110 | NG\_050001.1 | class A beta-lactamase SHV-110 | 99.77 | 100.00 | BETA-LACTAM | 368038 | 368898 | - | NCBI | 083 | klebsiella | 37 | 2 | Klebsiella pneumoniae |
| oqxB | NG\_048025.1 | multidrug efflux RND transporter permease subunit OqxB | 100.00 | 100.00 | PHENICOL;QUINOLONE | 430127 | 433279 | - | NCBI | 083 | klebsiella | 37 | 3 | Klebsiella pneumoniae |
| fosA6 | NG\_051497.1 | fosfomycin resistance glutathione transferase FosA6 | 99.52 | 100.00 | FOSFOMYCIN | 334488 | 334907 | - | NCBI | 083 | klebsiella | 37 | 4 | Klebsiella pneumoniae |
| blaOXA-48 | NG\_049762.1 | carbapenem-hydrolyzing class D beta-lactamase OXA-48 | 100.00 | 100.00 | CARBAPENEM | 1409 | 2206 | - | NCBI | 083 | klebsiella | 37 | 30 | Klebsiella pneumoniae |
| oqxA | NG\_048024.1 | multidrug efflux RND transporter periplasmic adaptor subunit OqxA | 100.00 | 100.00 | PHENICOL;QUINOLONE | 433303 | 434478 | - | NCBI | 083 | klebsiella | 37 | 3 | Klebsiella pneumoniae |
| blaOXY-4-1 | NG\_050613.1 | class A extended-spectrum beta-lactamase OXY-4-1 | 100.00 | 100.00 | BETA-LACTAM | 35817 | 36689 | + | NCBI | 084 | koxytoca | 386 | 18 | Klebsiella oxytoca |
| oqxB20 | NG\_050439.1 | multidrug efflux RND transporter permease subunit OqxB20 | 90.45 | 100.00 | PHENICOL;QUINOLONE | 53809 | 56961 | + | NCBI | 084 | koxytoca | 386 | 5 | Klebsiella oxytoca |
| oqxA10 | NG\_050418.1 | multidrug efflux RND transporter periplasmic adaptor subunit OqxA10 | 86.82 | 100.00 | PHENICOL;QUINOLONE | 52610 | 53785 | + | NCBI | 084 | koxytoca | 386 | 5 | Klebsiella oxytoca |
| tet(A) | NG\_048154.1 | tetracycline efflux MFS transporter Tet(A) | 100.00 | 100.00 | TETRACYCLINE | 1304 | 2503 | + | NCBI | 084 | koxytoca | 386 | 231 | Klebsiella oxytoca |
| aac(6')-Ib-D181Y | NG\_067946.1 | AAC(6')-Ib family aminoglycoside 6'-N-acetyltransferase | 99.82 | 100.00 | AMIKACIN;KANAMYCIN;TOBRAMYCIN | 119 | 673 | + | NCBI | 084 | koxytoca | 386 | 266 | Klebsiella oxytoca |
| aac(3)-IIe | NG\_047244.1 | aminoglycoside N-acetyltransferase AAC(3)-IIe | 99.77 | 100.00 | GENTAMICIN | 71 | 931 | + | NCBI | 084 | koxytoca | 386 | 262 | Klebsiella oxytoca |
| dfrA14 | NG\_056035.1 | trimethoprim-resistant dihydrofolate reductase DfrA14 | 100.00 | 100.00 | TRIMETHOPRIM | 1516 | 1989 | - | NCBI | 084 | koxytoca | 386 | 246 | Klebsiella oxytoca |
| qnrB1 | NG\_050469.1 | quinolone resistance pentapeptide repeat protein QnrB1 | 100.00 | 100.00 | QUINOLONE | 2414 | 3058 | + | NCBI | 084 | koxytoca | 386 | 242 | Klebsiella oxytoca |
| blaOXA-1 | NG\_049392.1 | oxacillin-hydrolyzing class D beta-lactamase OXA-1 | 100.00 | 100.00 | CEPHALOSPORIN | 804 | 1634 | + | NCBI | 084 | koxytoca | 386 | 266 | Klebsiella oxytoca |
| fosA\_gen | NG\_047885.1 | FosA family fosfomycin resistance glutathione transferase | 82.49 | 99.29 | FOSFOMYCIN | 44 | 460 | - | NCBI | 084 | koxytoca | 386 | 225 | Klebsiella oxytoca |
| blaKPC-2 | NG\_049253.1 | carbapenem-hydrolyzing class A beta-lactamase KPC-2 | 100.00 | 100.00 | CARBAPENEM | 2697 | 3578 | - | NCBI | 084 | koxytoca | 386 | 188 | Klebsiella oxytoca |
| erm(T) | NG\_047841.1 | 23S rRNA (adenine(2058)-N(6))-methyltransferase Erm(T) | 100.00 | 100.00 | MACROLIDE | 293 | 1027 | + | NCBI | 085 | efaecium | 612 | 224 | Enterococcus faecium |
| aph(2'')-Ih | NG\_047408.1 | aminoglycoside O-phosphotransferase APH(2'')-Ih | 82.43 | 89.15 | AMIKACIN;GENTAMICIN;KANAMYCIN;TOBRAMYCIN | 1157 | 1953 | + | NCBI | 085 | efaecium | 612 | 204 | Enterococcus faecium |
| aph(3')-IIIa | NG\_047418.1 | aminoglycoside O-phosphotransferase APH(3')-IIIa | 100.00 | 100.00 | AMIKACIN;KANAMYCIN | 521 | 1315 | + | NCBI | 085 | efaecium | 612 | 203 | Enterococcus faecium |
| aacA-ENT1 | NG\_052371.1 | aminoglycoside 6'-N-acetyltransferase | 100.00 | 100.00 | AMINOGLYCOSIDE | 79680 | 80228 | + | NCBI | 085 | efaecium | 612 | 2 | Enterococcus faecium |
| eat(A) | NG\_047762.1 | ABC-F type ribosomal protection protein Eat(A) | 99.80 | 100.00 | PLEUROMUTILIN | 22747 | 24249 | - | NCBI | 085 | efaecium | 612 | 8 | Enterococcus faecium |
| erm(B) | NG\_047801.1 | 23S rRNA (adenine(2058)-N(6))-methyltransferase Erm(B) | 99.60 | 99.87 | MACROLIDE | 289 | 1034 | + | NCBI | 085 | efaecium | 612 | 150 | Enterococcus faecium |
| vanR-A | NG\_048399.1 | VanA-type vancomycin resistance DNA-binding response regulator VanR | 100.00 | 100.00 | VANCOMYCIN | 4106 | 4801 | - | NCBI | 085 | efaecium | 612 | 133 | Enterococcus faecium |
| vanS-A | NG\_048425.1 | VanA-type vancomycin resistance histidine kinase VanS | 100.00 | 100.00 | VANCOMYCIN | 2974 | 4128 | - | NCBI | 085 | efaecium | 612 | 133 | Enterococcus faecium |
| dfrG | NG\_047756.1 | trimethoprim-resistant dihydrofolate reductase DfrG | 100.00 | 100.00 | TRIMETHOPRIM | 2801 | 3298 | - | NCBI | 085 | efaecium | 612 | 11 | Enterococcus faecium |
| vanA | NG\_048323.1 | D-alanine--(R)-lactate ligase VanA | 100.00 | 100.00 | VANCOMYCIN | 767 | 1798 | - | NCBI | 085 | efaecium | 612 | 133 | Enterococcus faecium |
| vanX-A | NG\_048477.1 | D-Ala-D-Ala dipeptidase VanX-A | 100.00 | 100.00 | VANCOMYCIN | 153 | 761 | - | NCBI | 085 | efaecium | 612 | 133 | Enterococcus faecium |
| msr(C) | NG\_048003.1 | ABC-F type ribosomal protection protein Msr(C) | 98.99 | 100.00 | MACROLIDE | 16012 | 17490 | - | NCBI | 085 | efaecium | 612 | 15 | Enterococcus faecium |
| vanY-A | NG\_048516.1 | D-Ala-D-Ala carboxypeptidase VanY-A | 100.00 | 100.00 | VANCOMYCIN | 348 | 1259 | + | NCBI | 085 | efaecium | 612 | 84 | Enterococcus faecium |
| vanZ-A | NG\_048534.1 | glycopeptide resistance protein VanZ-A | 100.00 | 100.00 | VANCOMYCIN | 1412 | 1897 | + | NCBI | 085 | efaecium | 612 | 84 | Enterococcus faecium |
| catA7 | NG\_047568.1 | type A-7 chloramphenicol O-acetyltransferase | 100.00 | 100.00 | CHLORAMPHENICOL | 7678 | 8325 | - | NCBI | 085 | efaecium | 612 | 84 | Enterococcus faecium |
| tet(L) | NG\_048203.1 | tetracycline efflux MFS transporter Tet(L) | 99.93 | 97.53 | TETRACYCLINE | 1544 | 2886 | + | NCBI | 085 | efaecium | 612 | 120 | Enterococcus faecium |
| vanH-A | NG\_048372.1 | D-lactate dehydrogenase VanH-A | 100.00 | 100.00 | VANCOMYCIN | 1791 | 2759 | - | NCBI | 085 | efaecium | 612 | 133 | Enterococcus faecium |
| aph(3')-Ia | NG\_047430.1 | aminoglycoside O-phosphotransferase APH(3')-Ia | 100.00 | 100.00 | KANAMYCIN | 213 | 1028 | + | NCBI | 086 | ecoli\_achtman\_4 | 744 | 247 | Escherichia coli |
| qnrS13 | NG\_059275.1 | quinolone resistance pentapeptide repeat protein QnrS13 | 100.00 | 100.00 | QUINOLONE | 1421 | 2077 | + | NCBI | 086 | ecoli\_achtman\_4 | 744 | 201 | Escherichia coli |
| tet(B) | NG\_048163.1 | tetracycline efflux MFS transporter Tet(B) | 100.00 | 100.00 | TETRACYCLINE | 2125 | 3330 | + | NCBI | 086 | ecoli\_achtman\_4 | 744 | 138 | Escherichia coli |
| blaOXA-48 | NG\_049762.1 | carbapenem-hydrolyzing class D beta-lactamase OXA-48 | 100.00 | 100.00 | CARBAPENEM | 1409 | 2206 | - | NCBI | 086 | ecoli\_achtman\_4 | 744 | 202 | Escherichia coli |
| catA1 | NG\_047582.1 | type A-1 chloramphenicol O-acetyltransferase | 99.85 | 100.00 | CHLORAMPHENICOL | 11946 | 12605 | - | NCBI | 086 | ecoli\_achtman\_4 | 744 | 96 | Escherichia coli |
| dfrA17 | NG\_047710.1 | trimethoprim-resistant dihydrofolate reductase DfrA17 | 99.79 | 100.00 | TRIMETHOPRIM | 5449 | 5922 | - | NCBI | 086 | ecoli\_achtman\_4 | 744 | 96 | Escherichia coli |
| sul1 | NG\_048082.1 | sulfonamide-resistant dihydropteroate synthase Sul1 | 100.00 | 100.00 | SULFONAMIDE | 3144 | 3983 | - | NCBI | 086 | ecoli\_achtman\_4 | 744 | 96 | Escherichia coli |
| blaKPC-2 | NG\_049253.1 | carbapenem-hydrolyzing class A beta-lactamase KPC-2 | 100.00 | 100.00 | CARBAPENEM | 202 | 1083 | + | NCBI | 086 | ecoli\_achtman\_4 | 744 | 70 | Escherichia coli |
| blaEC | NG\_047494.1 | BlaEC family class C beta-lactamase | 100.00 | 100.00 | BETA-LACTAM | 25140 | 26273 | - | NCBI | 086 | ecoli\_achtman\_4 | 744 | 34 | Escherichia coli |
| tet(A) | NG\_048154.1 | tetracycline efflux MFS transporter Tet(A) | 100.00 | 100.00 | TETRACYCLINE | 104181 | 105380 | - | NCBI | 086 | ecoli\_achtman\_4 | 744 | 8 | Escherichia coli |
| blaCTX-M-1 | NG\_048897.1 | class A extended-spectrum beta-lactamase CTX-M-1 | 100.00 | 100.00 | CEPHALOSPORIN | 76808 | 77683 | + | NCBI | 086 | ecoli\_achtman\_4 | 744 | 8 | Escherichia coli |
| aph(6)-Id | NG\_047464.1 | aminoglycoside O-phosphotransferase APH(6)-Id | 100.00 | 100.00 | STREPTOMYCIN | 9494 | 10330 | - | NCBI | 086 | ecoli\_achtman\_4 | 744 | 107 | Escherichia coli |
| aadA5 | NG\_047357.1 | ANT(3'')-Ia family aminoglycoside nucleotidyltransferase AadA5 | 100.00 | 100.00 | STREPTOMYCIN | 4530 | 5318 | - | NCBI | 086 | ecoli\_achtman\_4 | 744 | 96 | Escherichia coli |
| blaSHV-187 | NG\_050053.1 | class A beta-lactamase SHV-187 | 99.57 | 81.31 | BETA-LACTAM | 1 | 705 | - | NCBI | 087 | klebsiella | 258 | 358 | Klebsiella pneumoniae |
| vanZ-A | NG\_048534.1 | glycopeptide resistance protein VanZ-A | 100.00 | 100.00 | VANCOMYCIN | 200 | 685 | + | NCBI | 087 | klebsiella | 258 | 293 | Klebsiella pneumoniae |
| aac(6')-Ib-AKT | NG\_056043.1 | AAC(6')-Ib family aminoglycoside 6'-N-acetyltransferase | 100.00 | 100.00 | AMIKACIN;KANAMYCIN;TOBRAMYCIN | 499 | 1053 | - | NCBI | 087 | klebsiella | 258 | 243 | Klebsiella pneumoniae |
| blaOXA-9 | NG\_049830.1 | oxacillin-hydrolyzing class D beta-lactamase OXA-9 | 99.88 | 100.00 | BETA-LACTAM | 326 | 1150 | - | NCBI | 087 | klebsiella | 258 | 207 | Klebsiella pneumoniae |
| aph(2'')-IIa | NG\_047401.1 | aminoglycoside O-phosphotransferase APH(2'')-IIa | 100.00 | 100.00 | AMIKACIN;GENTAMICIN;KANAMYCIN;TOBRAMYCIN | 1514 | 2413 | - | NCBI | 087 | klebsiella | 258 | 82 | Klebsiella pneumoniae |
| vanR-A | NG\_048399.1 | VanA-type vancomycin resistance DNA-binding response regulator VanR | 100.00 | 100.00 | VANCOMYCIN | 4105 | 4800 | - | NCBI | 087 | klebsiella | 258 | 58 | Klebsiella pneumoniae |
| vanS-A | NG\_048425.1 | VanA-type vancomycin resistance histidine kinase VanS | 100.00 | 100.00 | VANCOMYCIN | 2973 | 4127 | - | NCBI | 087 | klebsiella | 258 | 58 | Klebsiella pneumoniae |
| vanX-A | NG\_048477.1 | D-Ala-D-Ala dipeptidase VanX-A | 100.00 | 100.00 | VANCOMYCIN | 152 | 760 | - | NCBI | 087 | klebsiella | 258 | 58 | Klebsiella pneumoniae |
| aac(6')-Im | NG\_047306.1 | aminoglycoside N-acetyltransferase AAC(6')-Im | 100.00 | 100.00 | AMIKACIN;KANAMYCIN;TOBRAMYCIN | 934 | 1470 | - | NCBI | 087 | klebsiella | 258 | 82 | Klebsiella pneumoniae |
| mph(A) | NG\_047986.1 | Mph(A) family macrolide 2'-phosphotransferase | 99.67 | 100.00 | MACROLIDE | 44733 | 45654 | - | NCBI | 087 | klebsiella | 258 | 29 | Klebsiella pneumoniae |
| aph(3')-IIIa | NG\_047418.1 | aminoglycoside O-phosphotransferase APH(3')-IIIa | 100.00 | 100.00 | AMIKACIN;KANAMYCIN | 921 | 1715 | - | NCBI | 087 | klebsiella | 258 | 44 | Klebsiella pneumoniae |
| ant(6)-Ia | NG\_047393.1 | aminoglycoside nucleotidyltransferase ANT(6)-Ia | 100.00 | 100.00 | STREPTOMYCIN | 2285 | 3193 | - | NCBI | 087 | klebsiella | 258 | 44 | Klebsiella pneumoniae |
| erm(B) | NG\_047802.1 | 23S rRNA (adenine(2058)-N(6))-methyltransferase Erm(B) | 100.00 | 100.00 | MACROLIDE | 7974 | 8711 | - | NCBI | 087 | klebsiella | 258 | 44 | Klebsiella pneumoniae |
| dfrA12 | NG\_047689.1 | trimethoprim-resistant dihydrofolate reductase DfrA12 | 100.00 | 100.00 | TRIMETHOPRIM | 36398 | 36895 | + | NCBI | 087 | klebsiella | 258 | 29 | Klebsiella pneumoniae |
| sul1 | NG\_048082.1 | sulfonamide-resistant dihydropteroate synthase Sul1 | 100.00 | 100.00 | SULFONAMIDE | 38599 | 39438 | + | NCBI | 087 | klebsiella | 258 | 29 | Klebsiella pneumoniae |
| vanA | NG\_048323.1 | D-alanine--(R)-lactate ligase VanA | 99.71 | 100.00 | VANCOMYCIN | 766 | 1797 | - | NCBI | 087 | klebsiella | 258 | 58 | Klebsiella pneumoniae |
| vanH-A | NG\_048372.1 | D-lactate dehydrogenase VanH-A | 100.00 | 100.00 | VANCOMYCIN | 1790 | 2758 | - | NCBI | 087 | klebsiella | 258 | 58 | Klebsiella pneumoniae |
| blaKPC-2 | NG\_049253.1 | carbapenem-hydrolyzing class A beta-lactamase KPC-2 | 100.00 | 100.00 | CARBAPENEM | 8802 | 9683 | - | NCBI | 087 | klebsiella | 258 | 29 | Klebsiella pneumoniae |
| blaTEM-1 | NG\_050145.1 | class A broad-spectrum beta-lactamase TEM-1 | 99.77 | 100.00 | BETA-LACTAM | 74650 | 75510 | - | NCBI | 087 | klebsiella | 258 | 23 | Klebsiella pneumoniae |
| fosA6 | NG\_051497.1 | fosfomycin resistance glutathione transferase FosA6 | 98.81 | 100.00 | FOSFOMYCIN | 14078 | 14497 | + | NCBI | 087 | klebsiella | 258 | 5 | Klebsiella pneumoniae |
| oqxA | NG\_048024.1 | multidrug efflux RND transporter periplasmic adaptor subunit OqxA | 100.00 | 100.00 | PHENICOL;QUINOLONE | 317190 | 318365 | - | NCBI | 087 | klebsiella | 258 | 3 | Klebsiella pneumoniae |
| oqxB | NG\_048025.1 | multidrug efflux RND transporter permease subunit OqxB | 100.00 | 100.00 | PHENICOL;QUINOLONE | 314014 | 317166 | - | NCBI | 087 | klebsiella | 258 | 3 | Klebsiella pneumoniae |
| catA1 | NG\_047582.1 | type A-1 chloramphenicol O-acetyltransferase | 99.85 | 100.00 | CHLORAMPHENICOL | 29728 | 30387 | + | NCBI | 087 | klebsiella | 258 | 29 | Klebsiella pneumoniae |
| aadA2 | NG\_047343.1 | ANT(3'')-Ia family aminoglycoside nucleotidyltransferase AadA2 | 100.00 | 100.00 | STREPTOMYCIN | 37303 | 38094 | + | NCBI | 087 | klebsiella | 258 | 29 | Klebsiella pneumoniae |
| aph(2'')-Ih | NG\_047408.1 | aminoglycoside O-phosphotransferase APH(2'')-Ih | 82.99 | 82.21 | AMIKACIN;GENTAMICIN;KANAMYCIN;TOBRAMYCIN | 2318 | 3052 | - | NCBI | 088 | efaecium | 203 | 103 | Enterococcus faecium |
| msr(C) | NG\_048003.1 | ABC-F type ribosomal protection protein Msr(C) | 98.92 | 100.00 | MACROLIDE | 80919 | 82397 | - | NCBI | 088 | efaecium | 203 | 2 | Enterococcus faecium |
| aacA-ENT1 | NG\_052371.1 | aminoglycoside 6'-N-acetyltransferase | 100.00 | 100.00 | AMINOGLYCOSIDE | 34197 | 34745 | - | NCBI | 088 | efaecium | 203 | 10 | Enterococcus faecium |
| vanZ-A | NG\_048534.1 | glycopeptide resistance protein VanZ-A | 100.00 | 100.00 | VANCOMYCIN | 9808 | 10293 | - | NCBI | 088 | efaecium | 203 | 66 | Enterococcus faecium |
| tet(M) | NG\_048213.1 | tetracycline resistance ribosomal protection protein Tet(M) | 99.79 | 100.00 | TETRACYCLINE | 19540 | 21459 | + | NCBI | 088 | efaecium | 203 | 21 | Enterococcus faecium |
| catA7 | NG\_047568.1 | type A-7 chloramphenicol O-acetyltransferase | 100.00 | 100.00 | CHLORAMPHENICOL | 3380 | 4027 | + | NCBI | 088 | efaecium | 203 | 66 | Enterococcus faecium |
| vanY-A | NG\_048516.1 | D-Ala-D-Ala carboxypeptidase VanY-A | 100.00 | 100.00 | VANCOMYCIN | 10446 | 11357 | - | NCBI | 088 | efaecium | 203 | 66 | Enterococcus faecium |
| dfrF | NG\_047755.1 | trimethoprim-resistant dihydrofolate reductase DfrF | 99.60 | 100.00 | TRIMETHOPRIM | 6425 | 6920 | + | NCBI | 088 | efaecium | 203 | 74 | Enterococcus faecium |
| vanR-A | NG\_048399.1 | VanA-type vancomycin resistance DNA-binding response regulator VanR | 100.00 | 100.00 | VANCOMYCIN | 179 | 874 | + | NCBI | 088 | efaecium | 203 | 90 | Enterococcus faecium |
| vanS-A | NG\_048425.1 | VanA-type vancomycin resistance histidine kinase VanS | 100.00 | 100.00 | VANCOMYCIN | 852 | 2006 | + | NCBI | 088 | efaecium | 203 | 90 | Enterococcus faecium |
| vanH-A | NG\_048372.1 | D-lactate dehydrogenase VanH-A | 100.00 | 100.00 | VANCOMYCIN | 2221 | 3189 | + | NCBI | 088 | efaecium | 203 | 90 | Enterococcus faecium |
| vanA | NG\_048323.1 | D-alanine--(R)-lactate ligase VanA | 100.00 | 100.00 | VANCOMYCIN | 3182 | 4213 | + | NCBI | 088 | efaecium | 203 | 90 | Enterococcus faecium |
| vanX-A | NG\_048477.1 | D-Ala-D-Ala dipeptidase VanX-A | 100.00 | 100.00 | VANCOMYCIN | 4219 | 4827 | + | NCBI | 088 | efaecium | 203 | 90 | Enterococcus faecium |
| ant(6)-Ia | NG\_047393.1 | aminoglycoside nucleotidyltransferase ANT(6)-Ia | 100.00 | 100.00 | STREPTOMYCIN | 2317 | 3225 | + | NCBI | 088 | efaecium | 203 | 100 | Enterococcus faecium |
| aph(2'')-Ih | NG\_047408.1 | aminoglycoside O-phosphotransferase APH(2'')-Ih | 82.43 | 89.15 | AMIKACIN;GENTAMICIN;KANAMYCIN;TOBRAMYCIN | 515 | 1311 | - | NCBI | 088 | efaecium | 203 | 103 | Enterococcus faecium |
| erm(B) | NG\_047801.1 | 23S rRNA (adenine(2058)-N(6))-methyltransferase Erm(B) | 99.60 | 99.87 | MACROLIDE | 290 | 1035 | - | NCBI | 088 | efaecium | 203 | 132 | Enterococcus faecium |
| eat(A) | NG\_047762.1 | ABC-F type ribosomal protection protein Eat(A) | 100.00 | 100.00 | PLEUROMUTILIN | 25590 | 27092 | + | NCBI | 088 | efaecium | 203 | 16 | Enterococcus faecium |
| aac(3)-Id | NG\_052489.1 | aminoglycoside N-acetyltransferase AAC(3)-Id | 100.00 | 100.00 | GENTAMICIN | 192 | 668 | - | NCBI | 089 | paeruginosa | 233 | 63 | Pseudomonas aeruginosa |
| tet(G) | NG\_051907.1 | tetracycline efflux MFS transporter Tet(G) | 100.00 | 100.00 | TETRACYCLINE | 55 | 1230 | + | NCBI | 089 | paeruginosa | 233 | 55 | Pseudomonas aeruginosa |
| aac(6')-Il | NG\_047303.1 | aminoglycoside N-acetyltransferase AAC(6')-Il | 100.00 | 100.00 | AMIKACIN;KANAMYCIN;TOBRAMYCIN | 1568 | 2026 | - | NCBI | 089 | paeruginosa | 233 | 53 | Pseudomonas aeruginosa |
| blaOXA-4 | NG\_049688.1 | OXA-1 family oxacillin-hydrolyzing class D beta-lactamase OXA-4 | 99.88 | 99.88 | BETA-LACTAM | 3087 | 3916 | - | NCBI | 089 | paeruginosa | 233 | 49 | Pseudomonas aeruginosa |
| fosA-354827590 | NG\_047883.1 | FosA family fosfomycin resistance glutathione transferase | 99.27 | 100.00 | FOSFOMYCIN | 25051 | 25458 | - | NCBI | 089 | paeruginosa | 233 | 8 | Pseudomonas aeruginosa |
| aph(3')-IIb | NG\_047424.1 | aminoglycoside O-phosphotransferase APH(3')-IIb | 98.76 | 100.00 | KANAMYCIN | 111868 | 112674 | + | NCBI | 089 | paeruginosa | 233 | 12 | Pseudomonas aeruginosa |
| blaPDC-374 | NG\_065926.1 | class C beta-lactamase PDC-374 | 98.86 | 100.00 | CEPHALOSPORIN | 124988 | 126210 | - | NCBI | 089 | paeruginosa | 233 | 12 | Pseudomonas aeruginosa |
| blaOXA-486 | NG\_050612.1 | OXA-50 family oxacillin-hydrolyzing class D beta-lactamase OXA-486 | 99.87 | 100.00 | BETA-LACTAM | 162364 | 163152 | + | NCBI | 089 | paeruginosa | 233 | 15 | Pseudomonas aeruginosa |
| catB7 | NG\_047614.1 | type B-4 chloramphenicol O-acetyltransferase CatB7 | 98.75 | 100.00 | CHLORAMPHENICOL | 52230 | 52868 | - | NCBI | 089 | paeruginosa | 233 | 35 | Pseudomonas aeruginosa |
| crpP | NG\_062203.1 | ciprofloxacin resistance protein CrpP | 98.48 | 100.00 | FLUOROQUINOLONE | 4203 | 4400 | + | NCBI | 089 | paeruginosa | 233 | 41 | Pseudomonas aeruginosa |
| blaVIM-2 | NG\_050347.1 | subclass B1 metallo-beta-lactamase VIM-2 | 100.00 | 100.00 | CARBAPENEM | 615 | 1415 | - | NCBI | 089 | paeruginosa | 233 | 53 | Pseudomonas aeruginosa |
| dfrB5 | NG\_047758.1 | trimethoprim-resistant dihydrofolate reductase DfrB5 | 100.00 | 100.00 | TRIMETHOPRIM | 230 | 466 | - | NCBI | 089 | paeruginosa | 233 | 53 | Pseudomonas aeruginosa |
| floR2 | NG\_047875.1 | chloramphenicol/florfenicol efflux MFS transporter FloR2 | 100.00 | 100.00 | CHLORAMPHENICOL;FLORFENICOL | 171 | 1385 | + | NCBI | 089 | paeruginosa | 233 | 54 | Pseudomonas aeruginosa |
| aadA2 | NG\_047343.1 | ANT(3'')-Ia family aminoglycoside nucleotidyltransferase AadA2 | 99.87 | 100.00 | STREPTOMYCIN | 2183 | 2974 | - | NCBI | 089 | paeruginosa | 233 | 49 | Pseudomonas aeruginosa |
| cmlA6 | NG\_047655.1 | chloramphenicol efflux MFS transporter CmlA6 | 100.00 | 100.00 | CHLORAMPHENICOL | 662 | 1921 | - | NCBI | 089 | paeruginosa | 233 | 49 | Pseudomonas aeruginosa |
| vanR-A | NG\_048399.1 | VanA-type vancomycin resistance DNA-binding response regulator VanR | 100.00 | 100.00 | VANCOMYCIN | 179 | 874 | + | NCBI | 090 | efaecium | 80 | 132 | Enterococcus faecium |
| msr(C) | NG\_048003.1 | ABC-F type ribosomal protection protein Msr(C) | 98.99 | 100.00 | MACROLIDE | 28342 | 29820 | + | NCBI | 090 | efaecium | 80 | 8 | Enterococcus faecium |
| aacA-ENT1 | NG\_052371.1 | aminoglycoside 6'-N-acetyltransferase | 100.00 | 100.00 | AMINOGLYCOSIDE | 34196 | 34744 | - | NCBI | 090 | efaecium | 80 | 16 | Enterococcus faecium |
| catA7 | NG\_047568.1 | type A-7 chloramphenicol O-acetyltransferase | 100.00 | 100.00 | CHLORAMPHENICOL | 2316 | 2963 | + | NCBI | 090 | efaecium | 80 | 105 | Enterococcus faecium |
| aph(3')-IIIa | NG\_047418.1 | aminoglycoside O-phosphotransferase APH(3')-IIIa | 100.00 | 100.00 | AMIKACIN;KANAMYCIN | 1097 | 1891 | + | NCBI | 090 | efaecium | 80 | 122 | Enterococcus faecium |
| tet(L) | NG\_048203.1 | tetracycline efflux MFS transporter Tet(L) | 99.85 | 97.53 | TETRACYCLINE | 3002 | 4344 | - | NCBI | 090 | efaecium | 80 | 125 | Enterococcus faecium |
| vanS-A | NG\_048425.1 | VanA-type vancomycin resistance histidine kinase VanS | 100.00 | 100.00 | VANCOMYCIN | 852 | 2006 | + | NCBI | 090 | efaecium | 80 | 132 | Enterococcus faecium |
| vanH-A | NG\_048372.1 | D-lactate dehydrogenase VanH-A | 100.00 | 100.00 | VANCOMYCIN | 2221 | 3189 | + | NCBI | 090 | efaecium | 80 | 132 | Enterococcus faecium |
| dfrG | NG\_047756.1 | trimethoprim-resistant dihydrofolate reductase DfrG | 100.00 | 100.00 | TRIMETHOPRIM | 2506 | 3003 | - | NCBI | 090 | efaecium | 80 | 159 | Enterococcus faecium |
| vanX-A | NG\_048477.1 | D-Ala-D-Ala dipeptidase VanX-A | 100.00 | 100.00 | VANCOMYCIN | 4219 | 4827 | + | NCBI | 090 | efaecium | 80 | 132 | Enterococcus faecium |
| eat(A) | NG\_047762.1 | ABC-F type ribosomal protection protein Eat(A) | 100.00 | 100.00 | PLEUROMUTILIN | 60273 | 61775 | - | NCBI | 090 | efaecium | 80 | 4 | Enterococcus faecium |
| erm(B) | NG\_047801.1 | 23S rRNA (adenine(2058)-N(6))-methyltransferase Erm(B) | 99.54 | 87.28 | MACROLIDE | 1 | 652 | + | NCBI | 090 | efaecium | 80 | 160 | Enterococcus faecium |
| vanY-A | NG\_048516.1 | D-Ala-D-Ala carboxypeptidase VanY-A | 100.00 | 100.00 | VANCOMYCIN | 347 | 1258 | + | NCBI | 090 | efaecium | 80 | 180 | Enterococcus faecium |
| vanZ-A | NG\_048534.1 | glycopeptide resistance protein VanZ-A | 100.00 | 100.00 | VANCOMYCIN | 1411 | 1896 | + | NCBI | 090 | efaecium | 80 | 180 | Enterococcus faecium |
| aph(2'')-If | NG\_047406.1 | aminoglycoside O-phosphotransferase APH(2'')-If | 82.65 | 84.40 | AMIKACIN;GENTAMICIN;KANAMYCIN;TOBRAMYCIN | 132 | 886 | + | NCBI | 090 | efaecium | 80 | 254 | Enterococcus faecium |
| vanA | NG\_048323.1 | D-alanine--(R)-lactate ligase VanA | 100.00 | 100.00 | VANCOMYCIN | 3182 | 4213 | + | NCBI | 090 | efaecium | 80 | 132 | Enterococcus faecium |
| blaOXA-66 | NG\_049806.1 | OXA-51 family carbapenem-hydrolyzing class D beta-lactamase OXA-66 | 100.00 | 100.00 | CARBAPENEM | 142613 | 143437 | - | NCBI | 091 | abaumannii\_2 | 2 | 11 | Acinetobacter baumannii |
| blaADC-73 | NG\_048678.1 | class C extended-spectrum beta-lactamase ADC-73 | 100.00 | 100.00 | CEPHALOSPORIN | 95979 | 97130 | - | NCBI | 091 | abaumannii\_2 | 2 | 18 | Acinetobacter baumannii |
| ant(3'')-IIa | NG\_054646.1 | aminoglycoside nucleotidyltransferase ANT(3'')-IIa | 98.61 | 100.00 | SPECTINOMYCIN;STREPTOMYCIN | 35573 | 36361 | - | NCBI | 091 | abaumannii\_2 | 2 | 10 | Acinetobacter baumannii |
| aph(3'')-Ib | NG\_056002.2 | aminoglycoside O-phosphotransferase APH(3'')-Ib | 99.88 | 98.31 | STREPTOMYCIN | 59316 | 60129 | + | NCBI | 091 | abaumannii\_2 | 2 | 22 | Acinetobacter baumannii |
| tet(B) | NG\_048161.1 | tetracycline efflux MFS transporter Tet(B) | 100.00 | 99.50 | TETRACYCLINE | 64338 | 65537 | + | NCBI | 091 | abaumannii\_2 | 2 | 22 | Acinetobacter baumannii |
| sul1 | NG\_048082.1 | sulfonamide-resistant dihydropteroate synthase Sul1 | 100.00 | 100.00 | SULFONAMIDE | 2081 | 2920 | - | NCBI | 091 | abaumannii\_2 | 2 | 59 | Acinetobacter baumannii |
| blaOXA-23 | NG\_049525.1 | carbapenem-hydrolyzing class D beta-lactamase OXA-23 | 100.00 | 100.00 | CARBAPENEM | 162 | 983 | + | NCBI | 091 | abaumannii\_2 | 2 | 65 | Acinetobacter baumannii |
| mph(E) | NG\_064660.1 | Mph(E) family macrolide 2'-phosphotransferase | 100.00 | 100.00 | MACROLIDE | 4279 | 5163 | - | NCBI | 091 | abaumannii\_2 | 2 | 21 | Acinetobacter baumannii |
| msr(E) | NG\_048007.1 | ABC-F type ribosomal protection protein Msr(E) | 100.00 | 100.00 | MACROLIDE | 5219 | 6694 | - | NCBI | 091 | abaumannii\_2 | 2 | 21 | Acinetobacter baumannii |
| armA | NG\_047476.1 | ArmA family 16S rRNA (guanine(1405)-N(7))-methyltransferase | 100.00 | 100.00 | GENTAMICIN | 8993 | 9766 | - | NCBI | 091 | abaumannii\_2 | 2 | 21 | Acinetobacter baumannii |
| aph(6)-Id | NG\_047464.1 | aminoglycoside O-phosphotransferase APH(6)-Id | 100.00 | 100.00 | STREPTOMYCIN | 60129 | 60965 | + | NCBI | 091 | abaumannii\_2 | 2 | 22 | Acinetobacter baumannii |
| blaKPC-2 | NG\_049253.1 | carbapenem-hydrolyzing class A beta-lactamase KPC-2 | 100.00 | 100.00 | CARBAPENEM | 5033 | 5914 | + | NCBI | 092 | ecloacae | - | 128 | Enterobacter cloacae |
| blaACT-50 | NG\_050713.1 | cephalosporin-hydrolyzing class C beta-lactamase ACT-50 | 98.87 | 100.00 | CEPHALOSPORIN | 14393 | 15538 | - | NCBI | 092 | ecloacae | - | 83 | Enterobacter cloacae |
| blaKPC-2 | NG\_049253.1 | carbapenem-hydrolyzing class A beta-lactamase KPC-2 | 100.00 | 100.00 | CARBAPENEM | 7621 | 8502 | - | NCBI | 092 | ecloacae | - | 128 | Enterobacter cloacae |
| oqxA6 | NG\_050424.1 | multidrug efflux RND transporter periplasmic adaptor subunit OqxA6 | 86.73 | 100.00 | PHENICOL;QUINOLONE | 746 | 1921 | + | NCBI | 092 | ecloacae | - | 146 | Enterobacter cloacae |
| oqxB20 | NG\_050439.1 | multidrug efflux RND transporter permease subunit OqxB20 | 89.63 | 99.11 | PHENICOL;QUINOLONE | 1946 | 5070 | + | NCBI | 092 | ecloacae | - | 146 | Enterobacter cloacae |
| fosA | NG\_050405.1 | fosfomycin resistance glutathione transferase FosA | 96.01 | 100.00 | FOSFOMYCIN | 5015 | 5440 | + | NCBI | 092 | ecloacae | - | 168 | Enterobacter cloacae |
| qnrB19 | NG\_050479.1 | quinolone resistance pentapeptide repeat protein QnrB19 | 100.00 | 100.00 | QUINOLONE | 1249 | 1893 | - | NCBI | 092 | ecloacae | - | 302 | Enterobacter cloacae |
| vanH-A | NG\_048372.1 | D-lactate dehydrogenase VanH-A | 100.00 | 100.00 | VANCOMYCIN | 6655 | 7623 | - | NCBI | 093 | efaecium | 80 | 79 | Enterococcus faecium |
| dfrG | NG\_047756.1 | trimethoprim-resistant dihydrofolate reductase DfrG | 100.00 | 100.00 | TRIMETHOPRIM | 2506 | 3003 | - | NCBI | 093 | efaecium | 80 | 105 | Enterococcus faecium |
| aph(3')-IIIa | NG\_047418.1 | aminoglycoside O-phosphotransferase APH(3')-IIIa | 100.00 | 100.00 | AMIKACIN;KANAMYCIN | 870 | 1664 | - | NCBI | 093 | efaecium | 80 | 113 | Enterococcus faecium |
| vanR-A | NG\_048399.1 | VanA-type vancomycin resistance DNA-binding response regulator VanR | 100.00 | 100.00 | VANCOMYCIN | 240 | 935 | + | NCBI | 093 | efaecium | 80 | 121 | Enterococcus faecium |
| vanX-A | NG\_048476.1 | D-Ala-D-Ala dipeptidase VanX-A | 100.00 | 100.00 | VANCOMYCIN | 5017 | 5625 | - | NCBI | 093 | efaecium | 80 | 79 | Enterococcus faecium |
| msr(C) | NG\_048003.1 | ABC-F type ribosomal protection protein Msr(C) | 98.99 | 100.00 | MACROLIDE | 28342 | 29820 | + | NCBI | 093 | efaecium | 80 | 9 | Enterococcus faecium |
| tet(L) | NG\_048203.1 | tetracycline efflux MFS transporter Tet(L) | 99.85 | 97.53 | TETRACYCLINE | 3002 | 4344 | - | NCBI | 093 | efaecium | 80 | 89 | Enterococcus faecium |
| aacA-ENT1 | NG\_052371.1 | aminoglycoside 6'-N-acetyltransferase | 100.00 | 100.00 | AMINOGLYCOSIDE | 34196 | 34744 | - | NCBI | 093 | efaecium | 80 | 19 | Enterococcus faecium |
| aph(2'')-Ih | NG\_047408.1 | aminoglycoside O-phosphotransferase APH(2'')-Ih | 82.43 | 89.15 | AMIKACIN;GENTAMICIN;KANAMYCIN;TOBRAMYCIN | 1030 | 1826 | + | NCBI | 093 | efaecium | 80 | 124 | Enterococcus faecium |
| vanA | NG\_048323.1 | D-alanine--(R)-lactate ligase VanA | 99.90 | 100.00 | VANCOMYCIN | 5631 | 6662 | - | NCBI | 093 | efaecium | 80 | 79 | Enterococcus faecium |
| eat(A) | NG\_047762.1 | ABC-F type ribosomal protection protein Eat(A) | 100.00 | 100.00 | PLEUROMUTILIN | 60273 | 61775 | - | NCBI | 093 | efaecium | 80 | 4 | Enterococcus faecium |
| vanY-A | NG\_048516.1 | D-Ala-D-Ala carboxypeptidase VanY-A | 99.89 | 99.89 | VANCOMYCIN | 3679 | 4589 | - | NCBI | 093 | efaecium | 80 | 79 | Enterococcus faecium |
| vanZ-A | NG\_048534.1 | glycopeptide resistance protein VanZ-A | 100.00 | 100.00 | VANCOMYCIN | 3041 | 3526 | - | NCBI | 093 | efaecium | 80 | 79 | Enterococcus faecium |
| vanS-A | NG\_048425.1 | VanA-type vancomycin resistance histidine kinase VanS | 100.00 | 100.00 | VANCOMYCIN | 913 | 2067 | + | NCBI | 093 | efaecium | 80 | 121 | Enterococcus faecium |
| oqxA10 | NG\_050418.1 | multidrug efflux RND transporter periplasmic adaptor subunit OqxA10 | 83.97 | 99.74 | PHENICOL;QUINOLONE | 92448 | 93623 | - | NCBI | 094 | koxytoca | - | 12 | Klebsiella oxytoca |
| oqxB9 | NG\_050458.1 | multidrug efflux RND transporter permease subunit OqxB9 | 87.99 | 99.27 | PHENICOL;QUINOLONE | 89295 | 92424 | - | NCBI | 094 | koxytoca | - | 12 | Klebsiella oxytoca |
| fosA\_gen | NG\_047885.1 | FosA family fosfomycin resistance glutathione transferase | 99.29 | 100.00 | FOSFOMYCIN | 28010 | 28429 | + | NCBI | 094 | koxytoca | - | 9 | Klebsiella oxytoca |
| blaORN-1 | NG\_049386.1 | class A broad-spectrum beta-lactamase ORN-1 | 99.89 | 100.00 | BETA-LACTAM | 714715 | 715590 | + | NCBI | 094 | koxytoca | - | 1 | Klebsiella oxytoca |
| blaOXA-1 | NG\_049392.1 | oxacillin-hydrolyzing class D beta-lactamase OXA-1 | 100.00 | 100.00 | CEPHALOSPORIN | 802 | 1632 | + | NCBI | 095 | ecloacae | - | 126 | Enterobacter cloacae |
| aac(6')-Ib-D181Y | NG\_067946.1 | AAC(6')-Ib family aminoglycoside 6'-N-acetyltransferase | 99.82 | 100.00 | AMIKACIN;KANAMYCIN;TOBRAMYCIN | 117 | 671 | + | NCBI | 095 | ecloacae | - | 126 | Enterobacter cloacae |
| aph(3'')-Ib | NG\_056002.2 | aminoglycoside O-phosphotransferase APH(3'')-Ib | 100.00 | 100.00 | STREPTOMYCIN | 4415 | 5242 | + | NCBI | 095 | ecloacae | - | 90 | Enterobacter cloacae |
| tet(A) | NG\_048157.1 | tetracycline efflux MFS transporter Tet(A) | 100.00 | 100.00 | TETRACYCLINE | 1019 | 2218 | - | NCBI | 095 | ecloacae | - | 117 | Enterobacter cloacae |
| dfrA14 | NG\_056035.1 | trimethoprim-resistant dihydrofolate reductase DfrA14 | 100.00 | 100.00 | TRIMETHOPRIM | 5430 | 5903 | - | NCBI | 095 | ecloacae | - | 108 | Enterobacter cloacae |
| blaCTX-M-15 | NG\_048935.1 | class A extended-spectrum beta-lactamase CTX-M-15 | 100.00 | 100.00 | CEPHALOSPORIN | 10481 | 11356 | + | NCBI | 095 | ecloacae | - | 90 | Enterobacter cloacae |
| blaTEM-1 | NG\_050145.1 | class A broad-spectrum beta-lactamase TEM-1 | 100.00 | 100.00 | BETA-LACTAM | 6799 | 7659 | - | NCBI | 095 | ecloacae | - | 90 | Enterobacter cloacae |
| aph(6)-Id | NG\_047464.1 | aminoglycoside O-phosphotransferase APH(6)-Id | 100.00 | 100.00 | STREPTOMYCIN | 5242 | 6078 | + | NCBI | 095 | ecloacae | - | 90 | Enterobacter cloacae |
| sul2 | NG\_051852.1 | sulfonamide-resistant dihydropteroate synthase Sul2 | 100.00 | 100.00 | SULFONAMIDE | 3563 | 4378 | + | NCBI | 095 | ecloacae | - | 90 | Enterobacter cloacae |
| blaACT-25 | NG\_048612.1 | cephalosporin-hydrolyzing class C beta-lactamase ACT-25 | 99.39 | 100.00 | CEPHALOSPORIN | 31 | 1176 | - | NCBI | 095 | ecloacae | - | 88 | Enterobacter cloacae |
| fosA | NG\_050405.1 | fosfomycin resistance glutathione transferase FosA | 96.48 | 100.00 | FOSFOMYCIN | 25993 | 26418 | - | NCBI | 095 | ecloacae | - | 61 | Enterobacter cloacae |
| oqxB9 | NG\_050458.1 | multidrug efflux RND transporter permease subunit OqxB9 | 89.36 | 98.95 | PHENICOL;QUINOLONE | 70197 | 73316 | + | NCBI | 095 | ecloacae | - | 2 | Enterobacter cloacae |
| oqxA9 | NG\_050427.1 | multidrug efflux RND transporter periplasmic adaptor subunit OqxA9 | 86.91 | 100.00 | PHENICOL;QUINOLONE | 68997 | 70172 | + | NCBI | 095 | ecloacae | - | 2 | Enterobacter cloacae |
| aac(3)-IIe | NG\_047244.1 | aminoglycoside N-acetyltransferase AAC(3)-IIe | 99.77 | 100.00 | GENTAMICIN | 71 | 931 | + | NCBI | 095 | ecloacae | - | 124 | Enterobacter cloacae |
| aadA1 | NG\_052536.1 | ANT(3'')-Ia family aminoglycoside nucleotidyltransferase AadA1 | 100.00 | 100.00 | STREPTOMYCIN | 33 | 824 | + | NCBI | 095 | ecloacae | - | 140 | Enterobacter cloacae |
| qnrB1 | NG\_050469.1 | quinolone resistance pentapeptide repeat protein QnrB1 | 100.00 | 100.00 | QUINOLONE | 522 | 1166 | + | NCBI | 095 | ecloacae | - | 127 | Enterobacter cloacae |
| catA1 | NG\_047582.1 | type A-1 chloramphenicol O-acetyltransferase | 99.85 | 98.79 | CHLORAMPHENICOL | 1 | 652 | - | NCBI | 095 | ecloacae | - | 143 | Enterobacter cloacae |
| blaTEM-1 | NG\_050145.1 | class A broad-spectrum beta-lactamase TEM-1 | 100.00 | 100.00 | BETA-LACTAM | 3963 | 4823 | - | NCBI | 096 | abaumannii\_2 | 79 | 26 | Acinetobacter baumannii |
| blaOXA-65 | NG\_049805.1 | OXA-51 family carbapenem-hydrolyzing class D beta-lactamase OXA-65 | 100.00 | 100.00 | CARBAPENEM | 69020 | 69844 | - | NCBI | 096 | abaumannii\_2 | 79 | 8 | Acinetobacter baumannii |
| blaADC-5 | NG\_048660.1 | class C beta-lactamase ADC-5 | 100.00 | 100.00 | CEPHALOSPORIN | 137 | 1288 | + | NCBI | 096 | abaumannii\_2 | 79 | 3 | Acinetobacter baumannii |
| cmlB1 | NG\_047658.1 | chloramphenicol efflux MFS transporter CmlB1 | 86.49 | 100.00 | CHLORAMPHENICOL | 1500 | 2765 | - | NCBI | 096 | abaumannii\_2 | 79 | 21 | Acinetobacter baumannii |
| aph(6)-Id | NG\_047464.1 | aminoglycoside O-phosphotransferase APH(6)-Id | 100.00 | 100.00 | STREPTOMYCIN | 8638 | 9474 | - | NCBI | 096 | abaumannii\_2 | 79 | 21 | Acinetobacter baumannii |
| aph(3'')-Ib | NG\_056002.2 | aminoglycoside O-phosphotransferase APH(3'')-Ib | 99.88 | 100.00 | STREPTOMYCIN | 9474 | 10301 | - | NCBI | 096 | abaumannii\_2 | 79 | 21 | Acinetobacter baumannii |
| blaOXA-72 | NG\_049813.1 | OXA-24 family carbapenem-hydrolyzing class D beta-lactamase OXA-72 | 100.00 | 100.00 | CARBAPENEM | 7147 | 7974 | + | NCBI | 096 | abaumannii\_2 | 79 | 22 | Acinetobacter baumannii |
| aac(3)-IIe | NG\_047244.1 | aminoglycoside N-acetyltransferase AAC(3)-IIe | 100.00 | 100.00 | GENTAMICIN | 2961 | 3821 | - | NCBI | 096 | abaumannii\_2 | 79 | 26 | Acinetobacter baumannii |
| ant(3'')-IIa | NG\_054648.1 | aminoglycoside nucleotidyltransferase ANT(3'')-IIa | 99.49 | 100.00 | SPECTINOMYCIN;STREPTOMYCIN | 123519 | 124307 | + | NCBI | 096 | abaumannii\_2 | 79 | 9 | Acinetobacter baumannii |
| sul2 | NG\_051852.1 | sulfonamide-resistant dihydropteroate synthase Sul2 | 100.00 | 100.00 | SULFONAMIDE | 145 | 960 | + | NCBI | 096 | abaumannii\_2 | 79 | 32 | Acinetobacter baumannii |
| aph(3')-VIa | NG\_047448.1 | aminoglycoside O-phosphotransferase APH(3')-VIa | 99.87 | 100.00 | AMIKACIN;KANAMYCIN | 60210 | 60989 | + | NCBI | 096 | abaumannii\_2 | 79 | 14 | Acinetobacter baumannii |
| dfrA12 | NG\_047689.1 | trimethoprim-resistant dihydrofolate reductase DfrA12 | 100.00 | 100.00 | TRIMETHOPRIM | 13237 | 13734 | - | NCBI | 097 | abaumannii\_2 | 492 | 32 | Acinetobacter baumannii |
| blaOXA-72 | NG\_049813.1 | OXA-24 family carbapenem-hydrolyzing class D beta-lactamase OXA-72 | 100.00 | 100.00 | CARBAPENEM | 198 | 1025 | + | NCBI | 097 | abaumannii\_2 | 492 | 36 | Acinetobacter baumannii |
| sul2 | NG\_051852.1 | sulfonamide-resistant dihydropteroate synthase Sul2 | 100.00 | 100.00 | SULFONAMIDE | 455 | 1270 | - | NCBI | 097 | abaumannii\_2 | 492 | 45 | Acinetobacter baumannii |
| sul1 | NG\_048082.1 | sulfonamide-resistant dihydropteroate synthase Sul1 | 100.00 | 100.00 | SULFONAMIDE | 10694 | 11533 | - | NCBI | 097 | abaumannii\_2 | 492 | 32 | Acinetobacter baumannii |
| armA | NG\_047476.1 | ArmA family 16S rRNA (guanine(1405)-N(7))-methyltransferase | 100.00 | 100.00 | GENTAMICIN | 6564 | 7337 | - | NCBI | 097 | abaumannii\_2 | 492 | 32 | Acinetobacter baumannii |
| blaOXA-66 | NG\_049806.1 | OXA-51 family carbapenem-hydrolyzing class D beta-lactamase OXA-66 | 100.00 | 100.00 | CARBAPENEM | 82447 | 83271 | + | NCBI | 097 | abaumannii\_2 | 492 | 2 | Acinetobacter baumannii |
| msr(E) | NG\_048007.1 | ABC-F type ribosomal protection protein Msr(E) | 100.00 | 100.00 | MACROLIDE | 2790 | 4265 | - | NCBI | 097 | abaumannii\_2 | 492 | 32 | Acinetobacter baumannii |
| mph(E) | NG\_064660.1 | Mph(E) family macrolide 2'-phosphotransferase | 100.00 | 100.00 | MACROLIDE | 1850 | 2734 | - | NCBI | 097 | abaumannii\_2 | 492 | 32 | Acinetobacter baumannii |
| ant(3'')-IIa | NG\_054646.1 | aminoglycoside nucleotidyltransferase ANT(3'')-IIa | 99.24 | 100.00 | SPECTINOMYCIN;STREPTOMYCIN | 31793 | 32581 | - | NCBI | 097 | abaumannii\_2 | 492 | 26 | Acinetobacter baumannii |
| blaADC-30 | NG\_048652.1 | class C extended-spectrum beta-lactamase ADC-30 | 100.00 | 100.00 | CEPHALOSPORIN | 137 | 1288 | + | NCBI | 097 | abaumannii\_2 | 492 | 3 | Acinetobacter baumannii |
| aadA2 | NG\_047343.1 | ANT(3'')-Ia family aminoglycoside nucleotidyltransferase AadA2 | 100.00 | 100.00 | STREPTOMYCIN | 12038 | 12829 | - | NCBI | 097 | abaumannii\_2 | 492 | 32 | Acinetobacter baumannii |
| aph(3'')-Ib | NG\_056002.2 | aminoglycoside O-phosphotransferase APH(3'')-Ib | 99.88 | 98.31 | STREPTOMYCIN | 74240 | 75053 | + | NCBI | 097 | abaumannii\_2 | 492 | 16 | Acinetobacter baumannii |
| aph(6)-Id | NG\_047464.1 | aminoglycoside O-phosphotransferase APH(6)-Id | 100.00 | 100.00 | STREPTOMYCIN | 75053 | 75889 | + | NCBI | 097 | abaumannii\_2 | 492 | 16 | Acinetobacter baumannii |
| tet(B) | NG\_048161.1 | tetracycline efflux MFS transporter Tet(B) | 100.00 | 99.50 | TETRACYCLINE | 79262 | 80461 | + | NCBI | 097 | abaumannii\_2 | 492 | 16 | Acinetobacter baumannii |
| blaPDC-55 | NG\_049929.1 | class C beta-lactamase PDC-55 | 98.86 | 100.00 | CEPHALOSPORIN | 90036 | 91258 | + | NCBI | 098 | paeruginosa | 254 | 17 | Pseudomonas aeruginosa |
| catB7 | NG\_047614.1 | type B-4 chloramphenicol O-acetyltransferase CatB7 | 99.06 | 100.00 | CHLORAMPHENICOL | 53234 | 53872 | - | NCBI | 098 | paeruginosa | 254 | 13 | Pseudomonas aeruginosa |
| fosA-354827590 | NG\_047883.1 | FosA family fosfomycin resistance glutathione transferase | 99.51 | 100.00 | FOSFOMYCIN | 28332 | 28739 | + | NCBI | 098 | paeruginosa | 254 | 14 | Pseudomonas aeruginosa |
| aph(3')-IIb | NG\_047424.1 | aminoglycoside O-phosphotransferase APH(3')-IIb | 99.01 | 100.00 | KANAMYCIN | 103585 | 104391 | - | NCBI | 098 | paeruginosa | 254 | 17 | Pseudomonas aeruginosa |
| blaOXA-905 | NG\_068024.1 | OXA-50 family oxacillin-hydrolyzing class D beta-lactamase OXA-905 | 99.75 | 100.00 | BETA-LACTAM | 79487 | 80275 | + | NCBI | 098 | paeruginosa | 254 | 8 | Pseudomonas aeruginosa |
| aph(6)-Id | NG\_047464.1 | aminoglycoside O-phosphotransferase APH(6)-Id | 100.00 | 100.00 | STREPTOMYCIN | 8538 | 9374 | - | NCBI | 099 | abaumannii\_2 | 79 | 258 | Acinetobacter baumannii |
| blaPDC-374 | NG\_065926.1 | class C beta-lactamase PDC-374 | 98.77 | 100.00 | CEPHALOSPORIN | 5356 | 6578 | + | NCBI | 099 | abaumannii\_2 | 79 | 369 | Acinetobacter baumannii |
| aph(3'')-Ib | NG\_056002.2 | aminoglycoside O-phosphotransferase APH(3'')-Ib | 99.88 | 100.00 | STREPTOMYCIN | 9374 | 10201 | - | NCBI | 099 | abaumannii\_2 | 79 | 258 | Acinetobacter baumannii |
| cmlB1 | NG\_047658.1 | chloramphenicol efflux MFS transporter CmlB1 | 86.49 | 100.00 | CHLORAMPHENICOL | 1400 | 2665 | - | NCBI | 099 | abaumannii\_2 | 79 | 258 | Acinetobacter baumannii |
| blaOXA-903 | NG\_068022.1 | OXA-50 family oxacillin-hydrolyzing class D beta-lactamase OXA-903 | 99.75 | 100.00 | BETA-LACTAM | 5919 | 6707 | + | NCBI | 099 | abaumannii\_2 | 79 | 183 | Acinetobacter baumannii |
| ant(3'')-IIa | NG\_054648.1 | aminoglycoside nucleotidyltransferase ANT(3'')-IIa | 99.49 | 100.00 | SPECTINOMYCIN;STREPTOMYCIN | 114214 | 115002 | + | NCBI | 099 | abaumannii\_2 | 79 | 5 | Acinetobacter baumannii |
| fosA-354827590 | NG\_047883.1 | FosA family fosfomycin resistance glutathione transferase | 99.75 | 100.00 | FOSFOMYCIN | 6243 | 6650 | + | NCBI | 099 | abaumannii\_2 | 79 | 170 | Acinetobacter baumannii |
| aph(3')-IIb | NG\_047424.1 | aminoglycoside O-phosphotransferase APH(3')-IIb | 99.26 | 100.00 | KANAMYCIN | 9904 | 10710 | + | NCBI | 099 | abaumannii\_2 | 79 | 112 | Acinetobacter baumannii |
| blaADC-5 | NG\_048660.1 | class C beta-lactamase ADC-5 | 100.00 | 100.00 | CEPHALOSPORIN | 36 | 1187 | + | NCBI | 099 | abaumannii\_2 | 79 | 44 | Acinetobacter baumannii |
| catB7 | NG\_047614.1 | type B-4 chloramphenicol O-acetyltransferase CatB7 | 99.37 | 100.00 | CHLORAMPHENICOL | 57659 | 58297 | + | NCBI | 099 | abaumannii\_2 | 79 | 25 | Acinetobacter baumannii |
| blaOXA-65 | NG\_049805.1 | OXA-51 family carbapenem-hydrolyzing class D beta-lactamase OXA-65 | 100.00 | 100.00 | CARBAPENEM | 65850 | 66674 | - | NCBI | 099 | abaumannii\_2 | 79 | 3 | Acinetobacter baumannii |
| blaOXA-72 | NG\_049813.1 | OXA-24 family carbapenem-hydrolyzing class D beta-lactamase OXA-72 | 100.00 | 100.00 | CARBAPENEM | 7042 | 7869 | + | NCBI | 099 | abaumannii\_2 | 79 | 271 | Acinetobacter baumannii |
| sul2 | NG\_051852.1 | sulfonamide-resistant dihydropteroate synthase Sul2 | 100.00 | 100.00 | SULFONAMIDE | 46 | 861 | + | NCBI | 099 | abaumannii\_2 | 79 | 764 | Acinetobacter baumannii |
| blaTEM-1 | NG\_050145.1 | class A broad-spectrum beta-lactamase TEM-1 | 100.00 | 100.00 | BETA-LACTAM | 1761 | 2621 | - | NCBI | 099 | abaumannii\_2 | 79 | 662 | Acinetobacter baumannii |
| aac(3)-IIe | NG\_047244.1 | aminoglycoside N-acetyltransferase AAC(3)-IIe | 100.00 | 100.00 | GENTAMICIN | 759 | 1619 | - | NCBI | 099 | abaumannii\_2 | 79 | 662 | Acinetobacter baumannii |
| aph(3')-VIa | NG\_047448.1 | aminoglycoside O-phosphotransferase APH(3')-VIa | 99.87 | 100.00 | AMIKACIN;KANAMYCIN | 7534 | 8313 | - | NCBI | 099 | abaumannii\_2 | 79 | 178 | Acinetobacter baumannii |
| blaPDC-374 | NG\_065926.1 | class C beta-lactamase PDC-374 | 98.94 | 100.00 | CEPHALOSPORIN | 13739 | 14961 | + | NCBI | 100 | paeruginosa | - | 150 | Pseudomonas aeruginosa |
| crpP | NG\_062203.1 | ciprofloxacin resistance protein CrpP | 98.99 | 100.00 | FLUOROQUINOLONE | 74787 | 74984 | - | NCBI | 100 | paeruginosa | - | 6 | Pseudomonas aeruginosa |
| blaOXA-494 | NG\_049773.1 | OXA-50 family oxacillin-hydrolyzing class D beta-lactamase OXA-494 | 99.75 | 100.00 | BETA-LACTAM | 19132 | 19920 | + | NCBI | 100 | paeruginosa | - | 35 | Pseudomonas aeruginosa |
| aph(3')-IIb | NG\_047424.1 | aminoglycoside O-phosphotransferase APH(3')-IIb | 98.76 | 100.00 | KANAMYCIN | 11562 | 12368 | + | NCBI | 100 | paeruginosa | - | 91 | Pseudomonas aeruginosa |
| fosA-354827590 | NG\_047883.1 | FosA family fosfomycin resistance glutathione transferase | 99.75 | 100.00 | FOSFOMYCIN | 8402 | 8809 | + | NCBI | 100 | paeruginosa | - | 145 | Pseudomonas aeruginosa |
| catB7 | NG\_047614.1 | type B-4 chloramphenicol O-acetyltransferase CatB7 | 98.12 | 100.00 | CHLORAMPHENICOL | 6604 | 7242 | - | NCBI | 100 | paeruginosa | - | 186 | Pseudomonas aeruginosa |

| GENE | ACCESSION | PRODUCT | %IDENTITY | %COVERAGE | RESISTANCE | START | END | STRAND | PREDICTION\_SOURCE | SAMPLE | SPECIES | SUBTYPE | CONTIG | SPECIES\_NAME |
| --- | --- | --- | --- | --- | --- | --- | --- | --- | --- | --- | --- | --- | --- | --- |
| fss3 | NP\_815578 | (fss3) Enterococcus faecalis surface protein Fss3 fibrinogen binding protein [Fibrinogen binding protein (AI273)] [Enterococcus faecalis V583] | 90.67 | 98.27 | NaN | 119802 | 122973 | + | VFDB | 001 | efaecium | 203 | 1 | Enterococcus faecium |
| sgrA | ZP\_00602747 | (sgrA) cell wall anchored protein SgrA [SgrA (VF0540)] [Enterococcus faecium DO] | 100.00 | 100.00 | NaN | 17673 | 18647 | - | VFDB | 001 | efaecium | 203 | 43 | Enterococcus faecium |
| ecbA | ZP\_00603098 | (ecbA) Collagen binding MSCRAMM EcbA [EcbA (VF0539)] [Enterococcus faecium DO] | 99.97 | 100.00 | NaN | 5800 | 9027 | - | VFDB | 001 | efaecium | 203 | 9 | Enterococcus faecium |
| acm | AAN12397 | (acm) collagen adhesin precursor Acm [Acm (VF0419)] [Enterococcus faecium str. TX2555] | 100.00 | 100.00 | NaN | 45646 | 47811 | - | VFDB | 001 | efaecium | 203 | 11 | Enterococcus faecium |
| gspI | YP\_404605 | (gspI) general secretion pathway protein I [T2SS (VF0333)] [Shigella dysenteriae Sd197] | 95.16 | 100.00 | NaN | 5504 | 5875 | - | VFDB | 002 | ecoli\_achtman\_4 | 167 | 18 | Escherichia coli |
| entE | NP\_752612 | (entE) 23-dihydroxybenzoate-AMP ligase component of enterobactin synthase multienzyme complex [Enterobactin (VF0228)] [Escherichia coli CFT073] | 95.47 | 100.00 | NaN | 16847 | 18457 | + | VFDB | 002 | ecoli\_achtman\_4 | 167 | 15 | Escherichia coli |
| entC | NP\_752611 | (entC) isochorismate synthase 1 [Enterobactin (VF0228)] [Escherichia coli CFT073] | 97.90 | 100.00 | NaN | 15650 | 16837 | + | VFDB | 002 | ecoli\_achtman\_4 | 167 | 15 | Escherichia coli |
| fepB | NP\_752610 | (fepB) ferrienterobactin ABC transporter periplasmic binding protein [Enterobactin (VF0228)] [Escherichia coli CFT073] | 97.49 | 100.00 | NaN | 14331 | 15287 | - | VFDB | 002 | ecoli\_achtman\_4 | 167 | 15 | Escherichia coli |
| entS | NP\_752609 | (entS) enterobactin exporter iron-regulated [enterobactin (IA019)] [Escherichia coli CFT073] | 95.44 | 100.00 | NaN | 13077 | 14327 | + | VFDB | 002 | ecoli\_achtman\_4 | 167 | 15 | Escherichia coli |
| fepD | NP\_752608 | (fepD) ferrienterobactin ABC transporter permease [Enterobactin (VF0228)] [Escherichia coli CFT073] | 96.07 | 100.00 | NaN | 11962 | 12978 | - | VFDB | 002 | ecoli\_achtman\_4 | 167 | 15 | Escherichia coli |
| fepG | NP\_752607 | (fepG) iron-enterobactin ABC transporter permease [Enterobactin (VF0228)] [Escherichia coli CFT073] | 94.06 | 100.00 | NaN | 10973 | 11965 | - | VFDB | 002 | ecoli\_achtman\_4 | 167 | 15 | Escherichia coli |
| fepC | NP\_752606 | (fepC) ferrienterobactin ABC transporter ATPase [Enterobactin (VF0228)] [Escherichia coli CFT073] | 97.30 | 100.00 | NaN | 10161 | 10976 | - | VFDB | 002 | ecoli\_achtman\_4 | 167 | 15 | Escherichia coli |
| fes | NP\_752602 | (fes) enterobactin/ferric enterobactin esterase [enterobactin (IA019)] [Escherichia coli CFT073] | 96.67 | 100.00 | NaN | 3514 | 4716 | + | VFDB | 002 | ecoli\_achtman\_4 | 167 | 15 | Escherichia coli |
| fepA | NP\_752600 | (fepA) ferrienterobactin outer membrane transporter [Enterobactin (VF0228)] [Escherichia coli CFT073] | 96.74 | 100.00 | NaN | 1031 | 3271 | - | VFDB | 002 | ecoli\_achtman\_4 | 167 | 15 | Escherichia coli |
| entB | NP\_752613 | (entB) isochorismatase [Enterobactin (VF0228)] [Escherichia coli CFT073] | 98.02 | 100.00 | NaN | 18471 | 19328 | + | VFDB | 002 | ecoli\_achtman\_4 | 167 | 15 | Escherichia coli |
| espX1 | NP\_285716 | (espX1) Type III secretion system effector EspX1 [LEE encoded T3SS (SS020)] [Escherichia coli O157:H7 str. EDL933] | 95.15 | 100.00 | NaN | 203441 | 204862 | + | VFDB | 002 | ecoli\_achtman\_4 | 167 | 6 | Escherichia coli |
| espY1 | NP\_285753 | (espY1) Type III secretion system effector EspY1 [LEE encoded T3SS (SS020)] [Escherichia coli O157:H7 str. EDL933] | 86.96 | 99.87 | NaN | 164486 | 165297 | - | VFDB | 002 | ecoli\_achtman\_4 | 167 | 6 | Escherichia coli |
| espL1 | NP\_288154 | (espL1) Type III secretion system effector espL1 [LEE encoded T3SS (SS020)] [Escherichia coli O157:H7 str. EDL933] | 97.58 | 100.00 | NaN | 211021 | 212920 | + | VFDB | 002 | ecoli\_achtman\_4 | 167 | 4 | Escherichia coli |
| espX5 | NP\_290699 | (espX5) Type III secretion system effector EspX5 [LEE encoded T3SS (SS020)] [Escherichia coli O157:H7 str. EDL933] | 97.14 | 100.00 | NaN | 80858 | 82150 | - | VFDB | 002 | ecoli\_achtman\_4 | 167 | 3 | Escherichia coli |
| espX4 | NP\_290672 | (espX4) Type III secretion system effector EspX4 [LEE encoded T3SS (SS020)] [Escherichia coli O157:H7 str. EDL933] | 93.30 | 99.94 | NaN | 37878 | 39458 | + | VFDB | 002 | ecoli\_achtman\_4 | 167 | 3 | Escherichia coli |
| espL4 | NP\_290644 | (espL4) Type III secretion system effector EspL4 [LEE encoded T3SS (SS020)] [Escherichia coli O157:H7 str. EDL933] | 97.26 | 100.00 | NaN | 7476 | 9662 | - | VFDB | 002 | ecoli\_achtman\_4 | 167 | 3 | Escherichia coli |
| ompA | AAF37887 | (ompA) outer membrane protein A [OmpA (VF0236)] [Escherichia coli O18:K1:H7 str. RS218] | 98.08 | 100.00 | NaN | 284115 | 285155 | - | VFDB | 002 | ecoli\_achtman\_4 | 167 | 1 | Escherichia coli |
| entD | NP\_752599 | (entD) phosphopantetheinyl transferase component of enterobactin synthase multienzyme complex [Enterobactin (VF0228)] [Escherichia coli CFT073] | 95.20 | 100.00 | NaN | 236 | 1006 | - | VFDB | 002 | ecoli\_achtman\_4 | 167 | 15 | Escherichia coli |
| espR4 | NP\_288396 | (espR4) Type III secretion system effector espR4 [LEE encoded T3SS (SS020)] [Escherichia coli O157:H7 str. EDL933] | 84.40 | 99.56 | NaN | 136332 | 137467 | - | VFDB | 002 | ecoli\_achtman\_4 | 167 | 8 | Escherichia coli |
| entF | NP\_752604 | (entF) enterobactin synthase multienzyme complex component ATP-dependent [Enterobactin (VF0228)] [Escherichia coli CFT073] | 95.69 | 99.28 | NaN | 4934 | 8787 | + | VFDB | 002 | ecoli\_achtman\_4 | 167 | 15 | Escherichia coli |
| gspL | YP\_404608 | (gspL) general secretion pathway protein L [T2SS (VF0333)] [Shigella dysenteriae Sd197] | 97.10 | 100.00 | NaN | 2753 | 3613 | - | VFDB | 002 | ecoli\_achtman\_4 | 167 | 18 | Escherichia coli |
| gspH | YP\_404604 | (gspH) general secretion pathway protein H [T2SS (VF0333)] [Shigella dysenteriae Sd197] | 95.48 | 100.00 | NaN | 5872 | 6402 | - | VFDB | 002 | ecoli\_achtman\_4 | 167 | 18 | Escherichia coli |
| gspG | YP\_404603 | (gspG) general secretion pathway protein G [T2SS (VF0333)] [Shigella dysenteriae Sd197] | 94.96 | 100.00 | NaN | 6439 | 6894 | - | VFDB | 002 | ecoli\_achtman\_4 | 167 | 18 | Escherichia coli |
| gspF | YP\_404602 | (gspF) general secretion pathway protein F [T2SS (VF0333)] [Shigella dysenteriae Sd197] | 96.00 | 100.00 | NaN | 6935 | 8134 | - | VFDB | 002 | ecoli\_achtman\_4 | 167 | 18 | Escherichia coli |
| gspE | YP\_404601 | (gspE) general secretion pathway protein E [T2SS (VF0333)] [Shigella dysenteriae Sd197] | 96.92 | 100.00 | NaN | 8134 | 9627 | - | VFDB | 002 | ecoli\_achtman\_4 | 167 | 18 | Escherichia coli |
| gspD | YP\_404600 | (gspD) general secretion pathway protein D [T2SS (VF0333)] [Shigella dysenteriae Sd197] | 96.06 | 100.00 | NaN | 9627 | 11477 | - | VFDB | 002 | ecoli\_achtman\_4 | 167 | 18 | Escherichia coli |
| gspC | YP\_404599 | (gspC) general secretion pathway protein C [T2SS (VF0333)] [Shigella dysenteriae Sd197] | 94.70 | 100.00 | NaN | 11717 | 12547 | - | VFDB | 002 | ecoli\_achtman\_4 | 167 | 18 | Escherichia coli |
| aslA | AAG10151 | (aslA) putative arylsulfatase [AslA (VF0238)] [Escherichia coli O18:K1:H7 str. RS218] | 93.49 | 100.00 | NaN | 49187 | 50615 | + | VFDB | 002 | ecoli\_achtman\_4 | 167 | 19 | Escherichia coli |
| fdeC | YP\_002390132 | (fdeC) adhesin FdeC [FdeC (VF0506)] [Escherichia coli O45:K1:H7 str. S88] | 94.21 | 99.88 | NaN | 47213 | 51461 | - | VFDB | 002 | ecoli\_achtman\_4 | 167 | 21 | Escherichia coli |
| ykgK/ecpR | NP\_286011 | (ykgK/ecpR) regulator protein EcpR [ECP (VF0404)] [Escherichia coli O157:H7 str. EDL933] | 96.95 | 100.00 | NaN | 54483 | 55073 | + | VFDB | 002 | ecoli\_achtman\_4 | 167 | 21 | Escherichia coli |
| gspM | YP\_404609 | (gspM) general secretion pathway protein M [T2SS (VF0333)] [Shigella dysenteriae Sd197] | 98.34 | 100.00 | NaN | 2215 | 2637 | - | VFDB | 002 | ecoli\_achtman\_4 | 167 | 18 | Escherichia coli |
| yagZ/ecpA | NP\_286010 | (yagZ/ecpA) E. coli common pilus structural subunit EcpA [ECP (VF0404)] [Escherichia coli O157:H7 str. EDL933] | 99.15 | 100.00 | NaN | 55148 | 55735 | + | VFDB | 002 | ecoli\_achtman\_4 | 167 | 21 | Escherichia coli |
| yagX/ecpC | NP\_286008 | (yagX/ecpC) E. coli common pilus usher EcpC [ECP (VF0404)] [Escherichia coli O157:H7 str. EDL933] | 98.77 | 100.00 | NaN | 56487 | 59012 | + | VFDB | 002 | ecoli\_achtman\_4 | 167 | 21 | Escherichia coli |
| yagW/ecpD | NP\_286007 | (yagW/ecpD) polymerized tip adhesin of ECP fibers [ECP (VF0404)] [Escherichia coli O157:H7 str. EDL933] | 99.09 | 100.00 | NaN | 59002 | 60645 | + | VFDB | 002 | ecoli\_achtman\_4 | 167 | 21 | Escherichia coli |
| yagV/ecpE | NP\_286006 | (yagV/ecpE) E. coli common pilus chaperone EcpE [ECP (VF0404)] [Escherichia coli O157:H7 str. EDL933] | 97.08 | 99.74 | NaN | 60569 | 61322 | + | VFDB | 002 | ecoli\_achtman\_4 | 167 | 21 | Escherichia coli |
| csgB | NP\_460114 | (csgB) minor curlin subunit precursor curli nucleator protein CsgB [Agf (VF0103)] [Salmonella enterica subsp. enterica serovar Typhimurium str. LT2] | 83.81 | 99.78 | NaN | 40611 | 41066 | - | VFDB | 002 | ecoli\_achtman\_4 | 167 | 30 | Escherichia coli |
| csgD | NP\_460113 | (csgD) DNA-binding transcriptional regulator CsgD [curli fibers/thin aggregative fimbriae (AGF) (AI094)] [Salmonella enterica subsp. enterica serovar Typhimurium str. LT2] | 81.11 | 100.00 | NaN | 41821 | 42471 | + | VFDB | 002 | ecoli\_achtman\_4 | 167 | 30 | Escherichia coli |
| csgF | NP\_460111 | (csgF) curli production assembly/transport protein CsgF [Agf (VF0103)] [Salmonella enterica subsp. enterica serovar Typhimurium str. LT2] | 81.23 | 99.04 | NaN | 42890 | 43306 | + | VFDB | 002 | ecoli\_achtman\_4 | 167 | 30 | Escherichia coli |
| csgG | NP\_460110 | (csgG) curli production assembly/transport protein CsgG [Agf (VF0103)] [Salmonella enterica subsp. enterica serovar Typhimurium str. LT2] | 83.45 | 100.00 | NaN | 43333 | 44166 | + | VFDB | 002 | ecoli\_achtman\_4 | 167 | 30 | Escherichia coli |
| espR1 | NP\_287686 | (espR1) Type III secretion system effector espR1 [LEE encoded T3SS (SS020)] [Escherichia coli O157:H7 str. EDL933] | 93.84 | 83.65 | NaN | 11597 | 12650 | - | VFDB | 002 | ecoli\_achtman\_4 | 167 | 37 | Escherichia coli |
| gspK | YP\_404607 | (gspK) general secretion pathway protein K [T2SS (VF0333)] [Shigella dysenteriae Sd197] | 97.03 | 100.00 | NaN | 3928 | 4905 | - | VFDB | 002 | ecoli\_achtman\_4 | 167 | 18 | Escherichia coli |
| yagY/ecpB | NP\_286009 | (yagY/ecpB) E. coli common pilus chaperone EcpB [ECP (VF0404)] [Escherichia coli O157:H7 str. EDL933] | 98.06 | 100.00 | NaN | 55793 | 56461 | + | VFDB | 002 | ecoli\_achtman\_4 | 167 | 21 | Escherichia coli |
| entA | NP\_752614 | (entA) 23-dihydro-23-dihydroxybenzoate dehydrogenase [Enterobactin (VF0228)] [Escherichia coli CFT073] | 95.72 | 100.00 | NaN | 19328 | 20074 | + | VFDB | 002 | ecoli\_achtman\_4 | 167 | 15 | Escherichia coli |
| gspJ | YP\_404606 | (gspJ) general secretion pathway protein J [T2SS (VF0333)] [Shigella dysenteriae Sd197] | 96.67 | 100.00 | NaN | 4902 | 5471 | - | VFDB | 002 | ecoli\_achtman\_4 | 167 | 18 | Escherichia coli |
| fliM | YP\_001006748 | (fliM) flagellar motor switch protein FliM [Flagella (VF0394)] [Yersinia enterocolitica subsp. enterocolitica 8081] | 81.05 | 100.00 | NaN | 78437 | 79444 | + | VFDB | 005 | cronobacter | - | 10 | Cronobacter sakazakii |
| ybtT | NP\_405469 | (ybtT) yersiniabactin biosynthetic protein YbtT [Yersiniabactin (VF0136)] [Yersinia pestis CO92] | 99.75 | 100.00 | NaN | 119108 | 119911 | + | VFDB | 005 | cronobacter | - | 12 | Cronobacter sakazakii |
| ybtU | NP\_405470 | (ybtU) yersiniabactin biosynthetic protein YbtU [Yersiniabactin (VF0136)] [Yersinia pestis CO92] | 99.73 | 100.00 | NaN | 118011 | 119111 | + | VFDB | 005 | cronobacter | - | 12 | Cronobacter sakazakii |
| irp1 | NP\_405471 | (irp1) yersiniabactin biosynthetic protein Irp1 [Yersiniabactin (VF0136)] [Yersinia pestis CO92] | 99.36 | 100.00 | NaN | 108523 | 118014 | + | VFDB | 005 | cronobacter | - | 12 | Cronobacter sakazakii |
| irp2 | NP\_405472 | (irp2) yersiniabactin biosynthetic protein Irp2 [Yersiniabactin (VF0136)] [Yersinia pestis CO92] | 99.74 | 100.00 | NaN | 102327 | 108435 | + | VFDB | 005 | cronobacter | - | 12 | Cronobacter sakazakii |
| ybtA | NP\_405473 | (ybtA) transcriptional regulator YbtA [Yersiniabactin (VF0136)] [Yersinia pestis CO92] | 99.79 | 100.00 | NaN | 101177 | 102136 | + | VFDB | 005 | cronobacter | - | 12 | Cronobacter sakazakii |
| yagZ/ecpA | NP\_286010 | (yagZ/ecpA) E. coli common pilus structural subunit EcpA [ECP (VF0404)] [Escherichia coli O157:H7 str. EDL933] | 82.56 | 99.15 | NaN | 79698 | 80281 | + | VFDB | 005 | cronobacter | - | 1 | Cronobacter sakazakii |
| yagW/ecpD | NP\_286007 | (yagW/ecpD) polymerized tip adhesin of ECP fibers [ECP (VF0404)] [Escherichia coli O157:H7 str. EDL933] | 80.52 | 95.26 | NaN | 83630 | 85195 | + | VFDB | 005 | cronobacter | - | 1 | Cronobacter sakazakii |
| entB | NP\_752613 | (entB) isochorismatase [Enterobactin (VF0228)] [Escherichia coli CFT073] | 82.42 | 99.18 | NaN | 383229 | 384080 | + | VFDB | 005 | cronobacter | - | 1 | Cronobacter sakazakii |
| entA | NP\_752614 | (entA) 23-dihydro-23-dihydroxybenzoate dehydrogenase [Enterobactin (VF0228)] [Escherichia coli CFT073] | 80.99 | 99.46 | NaN | 384090 | 384834 | + | VFDB | 005 | cronobacter | - | 1 | Cronobacter sakazakii |
| iroB | NP\_753168 | (iroB) glucosyltransferase IroB [Salmochelin (IA013)] [Escherichia coli CFT073] | 83.91 | 99.83 | NaN | 56004 | 57165 | + | VFDB | 005 | cronobacter | - | 8 | Cronobacter sakazakii |
| iroN | NP\_753164 | (iroN) salmochelin receptor IroN [IroN (VF0230)] [Escherichia coli CFT073] | 83.24 | 99.86 | NaN | 63294 | 65468 | - | VFDB | 005 | cronobacter | - | 8 | Cronobacter sakazakii |
| fliG | YP\_001006742 | (fliG) flagellar motor switch protein G [Flagella (VF0394)] [Yersinia enterocolitica subsp. enterocolitica 8081] | 80.06 | 100.00 | NaN | 73616 | 74608 | + | VFDB | 005 | cronobacter | - | 10 | Cronobacter sakazakii |
| ybtP | NP\_405474 | (ybtP) lipoprotein inner membrane ABC-transporter [Yersiniabactin (VF0136)] [Yersinia pestis CO92] | 99.67 | 100.00 | NaN | 99208 | 101010 | - | VFDB | 005 | cronobacter | - | 12 | Cronobacter sakazakii |
| fliN | YP\_001006749 | (fliN) flagellar motor switch protein FliN [Flagella (VF0394)] [Yersinia enterocolitica subsp. enterocolitica 8081] | 81.82 | 99.76 | NaN | 79437 | 79853 | + | VFDB | 005 | cronobacter | - | 10 | Cronobacter sakazakii |
| flgH | YP\_001006758 | (flgH) flagellar L-ring protein precursor FlgH [Flagella (VF0394)] [Yersinia enterocolitica subsp. enterocolitica 8081] | 80.59 | 93.42 | NaN | 101099 | 101737 | - | VFDB | 005 | cronobacter | - | 10 | Cronobacter sakazakii |
| ompA | AAF37887 | (ompA) outer membrane protein A [OmpA (VF0236)] [Escherichia coli O18:K1:H7 str. RS218] | 83.86 | 100.00 | NaN | 163382 | 164434 | + | VFDB | 005 | cronobacter | - | 10 | Cronobacter sakazakii |
| ybtS | NP\_405477 | (ybtS) salicylate synthase Irp9 [Yersiniabactin (VF0136)] [Yersinia pestis CO92] | 97.62 | 100.00 | NaN | 94814 | 96118 | - | VFDB | 005 | cronobacter | - | 12 | Cronobacter sakazakii |
| ybtE | NP\_405468 | (ybtE) yersiniabactin siderophore biosynthetic protein [Yersiniabactin (VF0136)] [Yersinia pestis CO92] | 99.81 | 100.00 | NaN | 119915 | 121492 | + | VFDB | 005 | cronobacter | - | 12 | Cronobacter sakazakii |
| fyuA | NP\_405467 | (fyuA) pesticin/yersiniabactin receptor protein [Yersiniabactin (VF0136)] [Yersinia pestis CO92] | 99.95 | 100.00 | NaN | 121623 | 123644 | + | VFDB | 005 | cronobacter | - | 12 | Cronobacter sakazakii |
| ybtQ | NP\_405475 | (ybtQ) inner membrane ABC-transporter YbtQ [Yersiniabactin (VF0136)] [Yersinia pestis CO92] | 99.89 | 100.00 | NaN | 97419 | 99221 | - | VFDB | 005 | cronobacter | - | 12 | Cronobacter sakazakii |
| ybtX | NP\_405476 | (ybtX) putative signal transducer [Yersiniabactin (VF0136)] [Yersinia pestis CO92] | 97.74 | 100.00 | NaN | 96146 | 97426 | - | VFDB | 005 | cronobacter | - | 12 | Cronobacter sakazakii |
| flgF | NP\_249772 | (flgF) flagellar basal-body rod protein FlgF [Flagella (VF0273)] [Pseudomonas aeruginosa PAO1] | 81.04 | 99.07 | NaN | 32820 | 33562 | + | VFDB | 006 | paeruginosa | - | 15 | Pseudomonas aeruginosa |
| fliP | NP\_250137 | (fliP) flagellar biosynthetic protein FliP [Flagella (VF0273)] [Pseudomonas aeruginosa PAO1] | 88.77 | 93.10 | NaN | 256403 | 257123 | + | VFDB | 006 | paeruginosa | - | 8 | Pseudomonas aeruginosa |
| fliQ | NP\_250138 | (fliQ) flagellar biosynthetic protein FliQ [Flagella (VF0273)] [Pseudomonas aeruginosa PAO1] | 83.70 | 100.00 | NaN | 257147 | 257416 | + | VFDB | 006 | paeruginosa | - | 8 | Pseudomonas aeruginosa |
| fliR | NP\_250139 | (fliR) flagellar biosynthetic protein FliR [Flagella (VF0273)] [Pseudomonas aeruginosa PAO1] | 84.72 | 99.10 | NaN | 257416 | 258186 | + | VFDB | 006 | paeruginosa | - | 8 | Pseudomonas aeruginosa |
| flhB | NP\_250140 | (flhB) flagellar biosynthetic protein FlhB [Flagella (VF0273)] [Pseudomonas aeruginosa PAO1] | 84.52 | 100.00 | NaN | 258192 | 259328 | + | VFDB | 006 | paeruginosa | - | 8 | Pseudomonas aeruginosa |
| algW | NP\_253136 | (algW) AlgW protein [Alginate regulation (CVF523)] [Pseudomonas aeruginosa PAO1] | 85.44 | 96.32 | NaN | 50924 | 52053 | + | VFDB | 006 | paeruginosa | - | 12 | Pseudomonas aeruginosa |
| pilB | NP\_253216 | (pilB) type 4 fimbrial biogenesis protein PilB [Type IV pili (VF0082)] [Pseudomonas aeruginosa PAO1] | 82.88 | 96.65 | NaN | 203379 | 205028 | + | VFDB | 006 | paeruginosa | - | 12 | Pseudomonas aeruginosa |
| xcpA/pilD | NP\_253218 | (xcpA/pilD) type 4 prepilin peptidase PilD [Type IV pili (VF0082)] [Pseudomonas aeruginosa PAO1] | 81.49 | 98.97 | NaN | 206252 | 207118 | + | VFDB | 006 | paeruginosa | - | 12 | Pseudomonas aeruginosa |
| pilR | NP\_253237 | (pilR) two-component response regulator PilR [Type IV pili (VF0082)] [Pseudomonas aeruginosa PAO1] | 86.58 | 99.78 | NaN | 230726 | 232063 | + | VFDB | 006 | paeruginosa | - | 12 | Pseudomonas aeruginosa |
| mucP | NP\_252339 | (mucP) metalloprotease protease [Alginate regulation (CVF523)] [Pseudomonas aeruginosa PAO1] | 80.13 | 99.93 | NaN | 112099 | 113451 | - | VFDB | 006 | paeruginosa | - | 13 | Pseudomonas aeruginosa |
| pilU | NP\_249087 | (pilU) twitching motility protein PilU [Type IV pili (VF0082)] [Pseudomonas aeruginosa PAO1] | 91.73 | 99.74 | NaN | 26307 | 27452 | + | VFDB | 006 | paeruginosa | - | 14 | Pseudomonas aeruginosa |
| pilG | NP\_249099 | (pilG) twitching motility protein PilG [Type IV pili (VF0082)] [Pseudomonas aeruginosa PAO1] | 89.71 | 100.00 | NaN | 37539 | 37946 | + | VFDB | 006 | paeruginosa | - | 14 | Pseudomonas aeruginosa |
| pilH | NP\_249100 | (pilH) twitching motility protein PilH [Type IV pili (VF0082)] [Pseudomonas aeruginosa PAO1] | 90.71 | 100.00 | NaN | 37991 | 38356 | + | VFDB | 006 | paeruginosa | - | 14 | Pseudomonas aeruginosa |
| pilI | NP\_249101 | (pilI) twitching motility protein PilI [Type IV pili (VF0082)] [Pseudomonas aeruginosa PAO1] | 85.88 | 98.88 | NaN | 38402 | 38932 | + | VFDB | 006 | paeruginosa | - | 14 | Pseudomonas aeruginosa |
| pilJ | NP\_249102 | (pilJ) twitching motility protein PilJ [Type IV pili (VF0082)] [Pseudomonas aeruginosa PAO1] | 91.23 | 99.07 | NaN | 39022 | 41051 | + | VFDB | 006 | paeruginosa | - | 14 | Pseudomonas aeruginosa |
| chpE | NP\_249108 | (chpE) probable chemotaxis protein [Type IV pili (VF0082)] [Pseudomonas aeruginosa PAO1] | 87.09 | 98.69 | NaN | 52325 | 52932 | + | VFDB | 006 | paeruginosa | - | 14 | Pseudomonas aeruginosa |
| flgB | NP\_249768 | (flgB) flagellar basal body rod protein FlgB [Deoxyhexose linking sugar 209 Da capping structure (AI138)] [Pseudomonas aeruginosa PAO1] | 80.54 | 99.51 | NaN | 29719 | 30124 | + | VFDB | 006 | paeruginosa | - | 15 | Pseudomonas aeruginosa |
| flgC | NP\_249769 | (flgC) flagellar basal-body rod protein FlgC [Flagella (VF0273)] [Pseudomonas aeruginosa PAO1] | 87.47 | 99.55 | NaN | 30132 | 30570 | + | VFDB | 006 | paeruginosa | - | 15 | Pseudomonas aeruginosa |
| pilT | NP\_249086 | (pilT) twitching motility protein PilT [Type IV pili (VF0082)] [Pseudomonas aeruginosa PAO1] | 91.79 | 100.00 | NaN | 25168 | 26202 | + | VFDB | 006 | paeruginosa | - | 14 | Pseudomonas aeruginosa |
| flgG | NP\_249773 | (flgG) flagellar basal-body rod protein FlgG [Flagella (VF0273)] [Pseudomonas aeruginosa PAO1] | 89.99 | 99.11 | NaN | 33616 | 34394 | + | VFDB | 006 | paeruginosa | - | 15 | Pseudomonas aeruginosa |
| fliG | NP\_249793 | (fliG) flagellar motor switch protein G [Flagella (VF0273)] [Pseudomonas aeruginosa PAO1] | 92.16 | 97.84 | NaN | 65977 | 66971 | + | VFDB | 006 | paeruginosa | - | 15 | Pseudomonas aeruginosa |
| flgH | NP\_249774 | (flgH) flagellar L-ring protein precursor FlgH [Flagella (VF0273)] [Pseudomonas aeruginosa PAO1] | 89.36 | 93.97 | NaN | 34490 | 35145 | + | VFDB | 006 | paeruginosa | - | 15 | Pseudomonas aeruginosa |
| flhA | NP\_250143 | (flhA) flagellar biosynthesis protein FlhA [Flagella (VF0273)] [Pseudomonas aeruginosa PAO1] | 89.22 | 100.00 | NaN | 27447 | 29570 | - | VFDB | 006 | paeruginosa | - | 32 | Pseudomonas aeruginosa |
| flhF | NP\_250144 | (flhF) flagellar biosynthesis protein FlhF [Flagella (VF0273)] [Pseudomonas aeruginosa PAO1] | 81.65 | 99.77 | NaN | 26131 | 27435 | - | VFDB | 006 | paeruginosa | - | 32 | Pseudomonas aeruginosa |
| fleN | NP\_250145 | (fleN) flagellar synthesis regulator FleN [Flagella (VF0273)] [Pseudomonas aeruginosa PAO1] | 86.09 | 98.58 | NaN | 25152 | 25982 | - | VFDB | 006 | paeruginosa | - | 32 | Pseudomonas aeruginosa |
| fliA | NP\_250146 | (fliA) flagellar biosynthesis sigma factor FliA [Deoxyhexose linking sugar 209 Da capping structure (AI138)] [Pseudomonas aeruginosa PAO1] | 85.39 | 99.19 | NaN | 24412 | 25149 | - | VFDB | 006 | paeruginosa | - | 32 | Pseudomonas aeruginosa |
| motC | NP\_250151 | (motC) flagellar motor protein [Deoxyhexose linking sugar 209 Da capping structure (AI138)] [Pseudomonas aeruginosa PAO1] | 86.10 | 100.00 | NaN | 18819 | 19559 | - | VFDB | 006 | paeruginosa | - | 32 | Pseudomonas aeruginosa |
| motD | NP\_250152 | (motD) flagellar motor protein [Deoxyhexose linking sugar 209 Da capping structure (AI138)] [Pseudomonas aeruginosa PAO1] | 80.81 | 94.95 | NaN | 17937 | 18784 | - | VFDB | 006 | paeruginosa | - | 32 | Pseudomonas aeruginosa |
| motB | NP\_253640 | (motB) flagellar motor protein [Deoxyhexose linking sugar 209 Da capping structure (AI138)] [Pseudomonas aeruginosa PAO1] | 83.84 | 97.32 | NaN | 45146 | 46188 | + | VFDB | 006 | paeruginosa | - | 27 | Pseudomonas aeruginosa |
| motA | NP\_253641 | (motA) flagellar motor protein [Deoxyhexose linking sugar 209 Da capping structure (AI138)] [Pseudomonas aeruginosa PAO1] | 87.41 | 99.77 | NaN | 44291 | 45140 | + | VFDB | 006 | paeruginosa | - | 27 | Pseudomonas aeruginosa |
| waaA | NP\_253675 | (waaA) lipopolysaccharide core biosynthesis protein WaaP [LPS (VF0085)] [Pseudomonas aeruginosa PAO1] | 84.04 | 98.12 | NaN | 7693 | 8952 | + | VFDB | 006 | paeruginosa | - | 27 | Pseudomonas aeruginosa |
| pilM | NP\_253731 | (pilM) type IV pilus inner membrane platform protein PilM [Type IV pili (VF0082)] [Pseudomonas aeruginosa PAO1] | 85.63 | 100.00 | NaN | 65998 | 67062 | - | VFDB | 006 | paeruginosa | - | 24 | Pseudomonas aeruginosa |
| pilN | NP\_253730 | (pilN) type IV pilus inner membrane platform protein PilN [Type IV pili (VF0082)] [Pseudomonas aeruginosa PAO1] | 84.19 | 99.83 | NaN | 65399 | 65998 | - | VFDB | 006 | paeruginosa | - | 24 | Pseudomonas aeruginosa |
| pilO | NP\_253729 | (pilO) type IV pilus inner membrane platform protein PilO [Type IV pili (VF0082)] [Pseudomonas aeruginosa PAO1] | 86.64 | 97.44 | NaN | 64792 | 65402 | - | VFDB | 006 | paeruginosa | - | 24 | Pseudomonas aeruginosa |
| fliN | NP\_250135 | (fliN) flagellar motor switch protein FliN [Flagella (VF0273)] [Pseudomonas aeruginosa PAO1] | 87.55 | 98.73 | NaN | 255399 | 255866 | + | VFDB | 006 | paeruginosa | - | 8 | Pseudomonas aeruginosa |
| pilP | NP\_253728 | (pilP) type IV pilus biogenesis protein PilP [Type IV pili (VF0082)] [Pseudomonas aeruginosa PAO1] | 82.17 | 97.52 | NaN | 64227 | 64740 | - | VFDB | 006 | paeruginosa | - | 24 | Pseudomonas aeruginosa |
| waaC | NP\_253698 | (waaC) 3-deoxy-D-manno-octulosonic-acid (KDO) transferase [LPS (VF0085)] [Pseudomonas aeruginosa PAO1] | 84.13 | 99.72 | NaN | 17909 | 18976 | - | VFDB | 006 | paeruginosa | - | 24 | Pseudomonas aeruginosa |
| waaG | NP\_253697 | (waaG) B-band O-antigen polymerase [LPS (VF0085)] [Pseudomonas aeruginosa PAO1] | 83.10 | 99.82 | NaN | 16791 | 17912 | - | VFDB | 006 | paeruginosa | - | 24 | Pseudomonas aeruginosa |
| waaP | NP\_253696 | (waaP) UDP-glucose:(heptosyl) LPS alpha 13-glucosyltransferase WaaG [LPS (VF0085)] [Pseudomonas aeruginosa PAO1] | 83.05 | 99.88 | NaN | 15936 | 16742 | - | VFDB | 006 | paeruginosa | - | 24 | Pseudomonas aeruginosa |
| mucD | NP\_249457 | (mucD) serine protease MucD precursor [Alginate regulation (CVF523)] [Pseudomonas aeruginosa PAO1] | 85.01 | 98.88 | NaN | 83609 | 85022 | + | VFDB | 006 | paeruginosa | - | 20 | Pseudomonas aeruginosa |
| algU | NP\_249453 | (algU) alginate biosynthesis protein AlgZ/FimS [Alginate (VF0091)] [Pseudomonas aeruginosa PAO1] | 88.44 | 98.11 | NaN | 80941 | 81511 | + | VFDB | 006 | paeruginosa | - | 20 | Pseudomonas aeruginosa |
| fliI | NP\_249795 | (fliI) flagellum-specific ATP synthase FliI [Flagella (VF0273)] [Pseudomonas aeruginosa PAO1] | 87.90 | 96.90 | NaN | 67749 | 69062 | + | VFDB | 006 | paeruginosa | - | 15 | Pseudomonas aeruginosa |
| fliF | NP\_249792 | (fliF) flagellar M-ring protein FliF [Flagella (VF0273)] [Pseudomonas aeruginosa PAO1] | 86.40 | 99.22 | NaN | 64160 | 65948 | + | VFDB | 006 | paeruginosa | - | 15 | Pseudomonas aeruginosa |
| fliE | NP\_249791 | (fliE) flagellar hook-basal body complex protein FliE [Flagella (VF0273)] [Pseudomonas aeruginosa PAO1] | 86.35 | 99.70 | NaN | 63801 | 64136 | + | VFDB | 006 | paeruginosa | - | 15 | Pseudomonas aeruginosa |
| fleR | NP\_249790 | (fleR) two-component response regulator [Flagella (VF0273)] [Pseudomonas aeruginosa PAO1] | 83.01 | 98.73 | NaN | 61596 | 63006 | + | VFDB | 006 | paeruginosa | - | 15 | Pseudomonas aeruginosa |
| fleS | NP\_249789 | (fleS) two-component sensor [Deoxyhexose linking sugar 209 Da capping structure (AI138)] [Pseudomonas aeruginosa PAO1] | 84.93 | 94.87 | NaN | 60421 | 61567 | + | VFDB | 006 | paeruginosa | - | 15 | Pseudomonas aeruginosa |
| fleQ | NP\_249788 | (fleQ) transcriptional regulator FleQ [Flagella (VF0273)] [Pseudomonas aeruginosa PAO1] | 88.37 | 99.80 | NaN | 58789 | 60264 | + | VFDB | 006 | paeruginosa | - | 15 | Pseudomonas aeruginosa |
| flgI | NP\_249775 | (flgI) flagellar P-ring protein precursor FlgI [Flagella (VF0273)] [Pseudomonas aeruginosa PAO1] | 90.63 | 95.23 | NaN | 35213 | 36269 | + | VFDB | 006 | paeruginosa | - | 15 | Pseudomonas aeruginosa |
| waaF | NP\_253699 | (waaF) heptosyltransferase I [LPS (VF0085)] [Pseudomonas aeruginosa PAO1] | 83.12 | 99.13 | NaN | 18978 | 20007 | - | VFDB | 006 | paeruginosa | - | 24 | Pseudomonas aeruginosa |
| fliM | NP\_250134 | (fliM) flagellar motor switch protein FliM [Flagella (VF0273)] [Pseudomonas aeruginosa PAO1] | 91.06 | 99.90 | NaN | 254402 | 255373 | + | VFDB | 006 | paeruginosa | - | 8 | Pseudomonas aeruginosa |
| algB | NP\_254170 | (algB) two-component response regulator AlgB [Alginate (VF0091)] [Pseudomonas aeruginosa PAO1] | 85.23 | 99.70 | NaN | 186115 | 187464 | - | VFDB | 006 | paeruginosa | - | 10 | Pseudomonas aeruginosa |
| algR | NP\_253948 | (algR) alginate biosynthesis regulatory protein AlgR [Alginate (VF0091)] [Pseudomonas aeruginosa PAO1] | 88.19 | 98.66 | NaN | 101267 | 102003 | - | VFDB | 006 | paeruginosa | - | 6 | Pseudomonas aeruginosa |
| pvdM | NP\_251083 | (pvdM) dipeptidase precursor [pyoverdine (IA001)] [Pseudomonas aeruginosa PAO1] | 85.38 | 99.48 | NaN | 171441 | 172787 | + | VFDB | 006 | paeruginosa | - | 1 | Pseudomonas aeruginosa |
| pvdN | NP\_251084 | (pvdN) pyoverdine biosynthesis protein PvdN [pyoverdine (IA001)] [Pseudomonas aeruginosa PAO1] | 85.43 | 99.30 | NaN | 172807 | 174087 | + | VFDB | 006 | paeruginosa | - | 1 | Pseudomonas aeruginosa |
| pvdO | NP\_251085 | (pvdO) pyoverdine biosynthesis protein PvdO [pyoverdine (IA001)] [Pseudomonas aeruginosa PAO1] | 87.95 | 100.00 | NaN | 174115 | 174969 | + | VFDB | 006 | paeruginosa | - | 1 | Pseudomonas aeruginosa |
| mbtH-like | NP\_251102 | (mbtH-like) MbtH-like protein from the pyoverdine cluster [pyoverdine (IA001)] [Pseudomonas aeruginosa PAO1] | 88.13 | 100.00 | NaN | 243123 | 243341 | - | VFDB | 006 | paeruginosa | - | 1 | Pseudomonas aeruginosa |
| pvdH | NP\_251103 | (pvdH) diaminobutyrate-2-oxoglutarate aminotransferase PvdH [pyoverdine (IA001)] [Pseudomonas aeruginosa PAO1] | 86.84 | 100.00 | NaN | 243736 | 245148 | - | VFDB | 006 | paeruginosa | - | 1 | Pseudomonas aeruginosa |
| pvdL | NP\_251114 | (pvdL) peptide synthase PvdL [pyoverdine (IA001)] [Pseudomonas aeruginosa PAO1] | 83.41 | 99.10 | NaN | 245382 | 258351 | - | VFDB | 006 | paeruginosa | - | 1 | Pseudomonas aeruginosa |
| algA | NP\_252241 | (algA) phosphomannose isomerase / guanosine 5'-diphospho-D-mannose pyrophosphorylase [Alginate (VF0091)] [Pseudomonas aeruginosa PAO1] | 89.02 | 97.65 | NaN | 306296 | 307707 | - | VFDB | 006 | paeruginosa | - | 2 | Pseudomonas aeruginosa |
| algF | NP\_252240 | (algF) alginate o-acetyltransferase AlgF [Alginate (VF0091)] [Pseudomonas aeruginosa PAO1] | 86.58 | 93.70 | NaN | 308189 | 308798 | - | VFDB | 006 | paeruginosa | - | 2 | Pseudomonas aeruginosa |
| algJ | NP\_252239 | (algJ) alginate o-acetyltransferase AlgJ [Alginate (VF0091)] [Pseudomonas aeruginosa PAO1] | 80.17 | 95.49 | NaN | 308921 | 310049 | - | VFDB | 006 | paeruginosa | - | 2 | Pseudomonas aeruginosa |
| algI | NP\_252238 | (algI) alginate o-acetyltransferase AlgI [Alginate (VF0091)] [Pseudomonas aeruginosa PAO1] | 91.36 | 100.00 | NaN | 310099 | 311661 | - | VFDB | 006 | paeruginosa | - | 2 | Pseudomonas aeruginosa |
| algZ | NP\_253949 | (algZ) sigma factor AlgU [Alginate (VF0091)] [Pseudomonas aeruginosa PAO1] | 83.09 | 96.84 | NaN | 102008 | 103052 | - | VFDB | 006 | paeruginosa | - | 6 | Pseudomonas aeruginosa |
| algX | NP\_252236 | (algX) alginate biosynthesis protein AlgX [Alginate (VF0091)] [Pseudomonas aeruginosa PAO1] | 86.55 | 94.53 | NaN | 313170 | 314519 | - | VFDB | 006 | paeruginosa | - | 2 | Pseudomonas aeruginosa |
| algG | NP\_252235 | (algG) outer membrane protein AlgE [Alginate (VF0091)] [Pseudomonas aeruginosa PAO1] | 87.56 | 90.62 | NaN | 314624 | 316109 | - | VFDB | 006 | paeruginosa | - | 2 | Pseudomonas aeruginosa |
| algE | NP\_252234 | (algE) alginate biosynthetic protein AlgK precursor [Alginate (VF0091)] [Pseudomonas aeruginosa PAO1] | 84.73 | 92.26 | NaN | 316363 | 317729 | - | VFDB | 006 | paeruginosa | - | 2 | Pseudomonas aeruginosa |
| algK | NP\_252233 | (algK) alginate biosynthesis protein Alg44 [Alginate (VF0091)] [Pseudomonas aeruginosa PAO1] | 86.80 | 93.35 | NaN | 317906 | 319238 | - | VFDB | 006 | paeruginosa | - | 2 | Pseudomonas aeruginosa |
| alg44 | NP\_252232 | (alg44) alginate biosynthesis protein Alg8 [Alginate (VF0091)] [Pseudomonas aeruginosa PAO1] | 87.86 | 100.00 | NaN | 319288 | 320457 | - | VFDB | 006 | paeruginosa | - | 2 | Pseudomonas aeruginosa |
| alg8 | NP\_252231 | (alg8) alginate-c5-mannuronan-epimerase AlgG [Alginate (VF0091)] [Pseudomonas aeruginosa PAO1] | 87.20 | 100.00 | NaN | 320516 | 322000 | - | VFDB | 006 | paeruginosa | - | 2 | Pseudomonas aeruginosa |
| algD | NP\_252230 | (algD) GDP-mannose 6-dehydrogenase AlgD [Alginate (VF0091)] [Pseudomonas aeruginosa PAO1] | 89.39 | 99.92 | NaN | 322091 | 323400 | - | VFDB | 006 | paeruginosa | - | 2 | Pseudomonas aeruginosa |
| algC | NP\_254009 | (algC) phosphomannomutase AlgC [Alginate biosynthesis (CVF522)] [Pseudomonas aeruginosa PAO1] | 88.53 | 99.78 | NaN | 50594 | 51985 | - | VFDB | 006 | paeruginosa | - | 5 | Pseudomonas aeruginosa |
| algL | NP\_252237 | (algL) poly(beta-d-mannuronate) lyase precursor AlgL [Alginate (VF0091)] [Pseudomonas aeruginosa PAO1] | 84.64 | 91.67 | NaN | 312058 | 313074 | - | VFDB | 006 | paeruginosa | - | 2 | Pseudomonas aeruginosa |
| flhA | NP\_250143 | (flhA) flagellar biosynthesis protein FlhA [Flagella (VF0273)] [Pseudomonas aeruginosa PAO1] | 85.48 | 99.91 | NaN | 951401 | 953522 | + | VFDB | 007 | - | - | 1 | Unknown |
| fleN | NP\_250145 | (fleN) flagellar synthesis regulator FleN [Flagella (VF0273)] [Pseudomonas aeruginosa PAO1] | 83.21 | 98.93 | NaN | 954962 | 955795 | + | VFDB | 007 | - | - | 1 | Unknown |
| fliA | NP\_250146 | (fliA) flagellar biosynthesis sigma factor FliA [Deoxyhexose linking sugar 209 Da capping structure (AI138)] [Pseudomonas aeruginosa PAO1] | 81.33 | 96.77 | NaN | 955809 | 956531 | + | VFDB | 007 | - | - | 1 | Unknown |
| pilR | NP\_253237 | (pilR) two-component response regulator PilR [Type IV pili (VF0082)] [Pseudomonas aeruginosa PAO1] | 82.43 | 99.85 | NaN | 208353 | 209693 | + | VFDB | 007 | - | - | 2 | Unknown |
| xcpA/pilD | NP\_253218 | (xcpA/pilD) type 4 prepilin peptidase PilD [Type IV pili (VF0082)] [Pseudomonas aeruginosa PAO1] | 80.66 | 99.66 | NaN | 246985 | 247856 | - | VFDB | 007 | - | - | 2 | Unknown |
| algW | NP\_253136 | (algW) AlgW protein [Alginate regulation (CVF523)] [Pseudomonas aeruginosa PAO1] | 81.52 | 95.98 | NaN | 357364 | 358490 | - | VFDB | 007 | - | - | 2 | Unknown |
| algU | NP\_249453 | (algU) alginate biosynthesis protein AlgZ/FimS [Alginate (VF0091)] [Pseudomonas aeruginosa PAO1] | 83.85 | 100.00 | NaN | 781635 | 782216 | + | VFDB | 007 | - | - | 2 | Unknown |
| hsiB1/vipA | NP\_248773 | (hsiB1/vipA) type VI secretion system tubule-forming protein VipA [HSI-I (VF0334)] [Pseudomonas aeruginosa PAO1] | 88.25 | 99.42 | NaN | 393326 | 393841 | + | VFDB | 007 | - | - | 3 | Unknown |
| dotU1 | NP\_248768 | (dotU1) type VI secretion system protein DotU [HSI-I (VF0334)] [Pseudomonas aeruginosa PAO1] | 82.02 | 99.11 | NaN | 386852 | 388193 | - | VFDB | 007 | - | - | 3 | Unknown |
| hsiJ1 | NP\_248769 | (hsiJ1) type VI secretion system hcp secretion island protein HsiJ1 [HSI-I (VF0334)] [Pseudomonas aeruginosa PAO1] | 82.17 | 99.40 | NaN | 388207 | 389534 | - | VFDB | 007 | - | - | 3 | Unknown |
| fliR | NP\_250139 | (fliR) flagellar biosynthetic protein FliR [Flagella (VF0273)] [Pseudomonas aeruginosa PAO1] | 80.59 | 98.97 | NaN | 949275 | 950045 | + | VFDB | 007 | - | - | 1 | Unknown |
| hsiC1/vipB | NP\_248774 | (hsiC1/vipB) type VI secretion system tubule-forming protein VipB [HSI-I (VF0334)] [Pseudomonas aeruginosa PAO1] | 91.40 | 99.80 | NaN | 393854 | 395350 | + | VFDB | 007 | - | - | 3 | Unknown |
| hcp1 | NP\_248775 | (hcp1) type VI secretion system substrate Hcp1 [HSI-I (VF0334)] [Pseudomonas aeruginosa PAO1] | 88.14 | 100.00 | NaN | 395425 | 395913 | + | VFDB | 007 | - | - | 3 | Unknown |
| hsiF1 | NP\_248777 | (hsiF1) type VI secretion system hcp secretion island protein HsiF1 a gp25-like protein but not exhibit lysozyme activity [HSI-I (VF0334)] [Pseudomonas aeruginosa PAO1] | 82.16 | 94.51 | NaN | 397683 | 398164 | + | VFDB | 007 | - | - | 3 | Unknown |
| hsiG1 | NP\_248778 | (hsiG1) type VI secretion system hcp secretion island protein HsiG1 [HSI-I (VF0334)] [Pseudomonas aeruginosa PAO1] | 83.39 | 99.25 | NaN | 398195 | 400050 | + | VFDB | 007 | - | - | 3 | Unknown |
| icmF1/tssM1 | NP\_248767 | (icmF1/tssM1) type VI secretion system protein IcmF1 [HSI-I (VF0334)] [Pseudomonas aeruginosa PAO1] | 82.43 | 98.67 | NaN | 383335 | 386602 | - | VFDB | 007 | - | - | 3 | Unknown |
| fliQ | NP\_250138 | (fliQ) flagellar biosynthetic protein FliQ [Flagella (VF0273)] [Pseudomonas aeruginosa PAO1] | 82.29 | 99.63 | NaN | 949004 | 949273 | + | VFDB | 007 | - | - | 1 | Unknown |
| fliI | NP\_249795 | (fliI) flagellum-specific ATP synthase FliI [Flagella (VF0273)] [Pseudomonas aeruginosa PAO1] | 84.22 | 95.65 | NaN | 939876 | 941173 | + | VFDB | 007 | - | - | 1 | Unknown |
| fliN | NP\_250135 | (fliN) flagellar motor switch protein FliN [Flagella (VF0273)] [Pseudomonas aeruginosa PAO1] | 83.23 | 98.10 | NaN | 947294 | 947767 | + | VFDB | 007 | - | - | 1 | Unknown |
| xcpQ | NP\_251795 | (xcpQ) general secretion pathway protein D [xcp secretion system (VF0084)] [Pseudomonas aeruginosa PAO1] | 80.34 | 90.34 | NaN | 753545 | 755347 | - | VFDB | 007 | - | - | 1 | Unknown |
| xcpR | NP\_251793 | (xcpR) general secretion pathway protein E [xcp secretion system (VF0084)] [Pseudomonas aeruginosa PAO1] | 85.69 | 97.55 | NaN | 756375 | 757849 | + | VFDB | 007 | - | - | 1 | Unknown |
| xcpS | NP\_251792 | (xcpS) general secretion pathway protein F [xcp secretion system (VF0084)] [Pseudomonas aeruginosa PAO1] | 84.28 | 99.75 | NaN | 757850 | 759067 | + | VFDB | 007 | - | - | 1 | Unknown |
| xcpT | NP\_251791 | (xcpT) general secretion pathway protein G [xcp secretion system (VF0084)] [Pseudomonas aeruginosa PAO1] | 90.12 | 92.84 | NaN | 759089 | 759503 | + | VFDB | 007 | - | - | 1 | Unknown |
| flgB | NP\_249768 | (flgB) flagellar basal body rod protein FlgB [Deoxyhexose linking sugar 209 Da capping structure (AI138)] [Pseudomonas aeruginosa PAO1] | 80.20 | 99.75 | NaN | 900470 | 900877 | + | VFDB | 007 | - | - | 1 | Unknown |
| flgC | NP\_249769 | (flgC) flagellar basal-body rod protein FlgC [Flagella (VF0273)] [Pseudomonas aeruginosa PAO1] | 83.44 | 100.00 | NaN | 900889 | 901341 | + | VFDB | 007 | - | - | 1 | Unknown |
| flgF | NP\_249772 | (flgF) flagellar basal-body rod protein FlgF [Flagella (VF0273)] [Pseudomonas aeruginosa PAO1] | 80.93 | 98.80 | NaN | 903604 | 904344 | + | VFDB | 007 | - | - | 1 | Unknown |
| flgG | NP\_249773 | (flgG) flagellar basal-body rod protein FlgG [Flagella (VF0273)] [Pseudomonas aeruginosa PAO1] | 88.47 | 94.91 | NaN | 904398 | 905143 | + | VFDB | 007 | - | - | 1 | Unknown |
| clpV1 | NP\_248780 | (clpV1) type VI secretion system AAA+ family ATPase [HSI-I (VF0334)] [Pseudomonas aeruginosa PAO1] | 88.04 | 97.01 | NaN | 401061 | 403694 | + | VFDB | 007 | - | - | 3 | Unknown |
| flgI | NP\_249775 | (flgI) flagellar P-ring protein precursor FlgI [Flagella (VF0273)] [Pseudomonas aeruginosa PAO1] | 84.81 | 94.86 | NaN | 905973 | 907025 | + | VFDB | 007 | - | - | 1 | Unknown |
| fleQ | NP\_249788 | (fleQ) transcriptional regulator FleQ [Flagella (VF0273)] [Pseudomonas aeruginosa PAO1] | 85.20 | 100.00 | NaN | 931514 | 932986 | + | VFDB | 007 | - | - | 1 | Unknown |
| fliE | NP\_249791 | (fliE) flagellar hook-basal body complex protein FliE [Flagella (VF0273)] [Pseudomonas aeruginosa PAO1] | 83.73 | 98.79 | NaN | 935919 | 936250 | + | VFDB | 007 | - | - | 1 | Unknown |
| fliF | NP\_249792 | (fliF) flagellar M-ring protein FliF [Flagella (VF0273)] [Pseudomonas aeruginosa PAO1] | 81.20 | 94.49 | NaN | 936339 | 938055 | + | VFDB | 007 | - | - | 1 | Unknown |
| fliG | NP\_249793 | (fliG) flagellar motor switch protein G [Flagella (VF0273)] [Pseudomonas aeruginosa PAO1] | 88.40 | 100.00 | NaN | 938050 | 939066 | + | VFDB | 007 | - | - | 1 | Unknown |
| fliM | NP\_250134 | (fliM) flagellar motor switch protein FliM [Flagella (VF0273)] [Pseudomonas aeruginosa PAO1] | 88.39 | 99.90 | NaN | 946287 | 947258 | + | VFDB | 007 | - | - | 1 | Unknown |
| fliP | NP\_250137 | (fliP) flagellar biosynthetic protein FliP [Flagella (VF0273)] [Pseudomonas aeruginosa PAO1] | 85.32 | 93.75 | NaN | 948273 | 949001 | + | VFDB | 007 | - | - | 1 | Unknown |
| flgH | NP\_249774 | (flgH) flagellar L-ring protein precursor FlgH [Flagella (VF0273)] [Pseudomonas aeruginosa PAO1] | 82.29 | 99.57 | NaN | 905197 | 905910 | + | VFDB | 007 | - | - | 1 | Unknown |
| pvdA | NP\_251076 | (pvdA) L-ornithine N5-oxygenase PvdA [Pyoverdine (VF0094)] [Pseudomonas aeruginosa PAO1] | 80.74 | 93.99 | NaN | 551570 | 552830 | - | VFDB | 007 | - | - | 3 | Unknown |
| pilU | NP\_249087 | (pilU) twitching motility protein PilU [Type IV pili (VF0082)] [Pseudomonas aeruginosa PAO1] | 88.77 | 99.74 | NaN | 141757 | 142902 | - | VFDB | 007 | - | - | 4 | Unknown |
| algR | NP\_253948 | (algR) alginate biosynthesis regulatory protein AlgR [Alginate (VF0091)] [Pseudomonas aeruginosa PAO1] | 82.00 | 99.60 | NaN | 332624 | 333370 | - | VFDB | 007 | - | - | 4 | Unknown |
| pvdM | NP\_251083 | (pvdM) dipeptidase precursor [pyoverdine (IA001)] [Pseudomonas aeruginosa PAO1] | 81.84 | 96.36 | NaN | 468053 | 469357 | + | VFDB | 007 | - | - | 3 | Unknown |
| pvdN | NP\_251084 | (pvdN) pyoverdine biosynthesis protein PvdN [pyoverdine (IA001)] [Pseudomonas aeruginosa PAO1] | 80.26 | 99.45 | NaN | 469397 | 470686 | + | VFDB | 007 | - | - | 3 | Unknown |
| pvdO | NP\_251085 | (pvdO) pyoverdine biosynthesis protein PvdO [pyoverdine (IA001)] [Pseudomonas aeruginosa PAO1] | 80.77 | 96.96 | NaN | 470726 | 471558 | + | VFDB | 007 | - | - | 3 | Unknown |
| motA | NP\_253641 | (motA) flagellar motor protein [Deoxyhexose linking sugar 209 Da capping structure (AI138)] [Pseudomonas aeruginosa PAO1] | 81.45 | 100.00 | NaN | 329938 | 330789 | + | VFDB | 007 | - | - | 6 | Unknown |
| waaA | NP\_253675 | (waaA) lipopolysaccharide core biosynthesis protein WaaP [LPS (VF0085)] [Pseudomonas aeruginosa PAO1] | 80.83 | 97.73 | NaN | 276845 | 278097 | + | VFDB | 007 | - | - | 6 | Unknown |
| motB | NP\_253640 | (motB) flagellar motor protein [Deoxyhexose linking sugar 209 Da capping structure (AI138)] [Pseudomonas aeruginosa PAO1] | 81.61 | 82.66 | NaN | 330803 | 331687 | + | VFDB | 007 | - | - | 6 | Unknown |
| waaG | NP\_253697 | (waaG) B-band O-antigen polymerase [LPS (VF0085)] [Pseudomonas aeruginosa PAO1] | 83.14 | 98.48 | NaN | 250960 | 252066 | + | VFDB | 007 | - | - | 6 | Unknown |
| waaF | NP\_253699 | (waaF) heptosyltransferase I [LPS (VF0085)] [Pseudomonas aeruginosa PAO1] | 80.64 | 100.00 | NaN | 248859 | 249896 | + | VFDB | 007 | - | - | 6 | Unknown |
| algC | NP\_254009 | (algC) phosphomannomutase AlgC [Alginate biosynthesis (CVF522)] [Pseudomonas aeruginosa PAO1] | 88.65 | 94.97 | NaN | 414540 | 415861 | + | VFDB | 007 | - | - | 4 | Unknown |
| pilJ | NP\_249102 | (pilJ) twitching motility protein PilJ [Type IV pili (VF0082)] [Pseudomonas aeruginosa PAO1] | 86.60 | 99.71 | NaN | 128858 | 130903 | - | VFDB | 007 | - | - | 4 | Unknown |
| pilI | NP\_249101 | (pilI) twitching motility protein PilI [Type IV pili (VF0082)] [Pseudomonas aeruginosa PAO1] | 82.28 | 99.81 | NaN | 131098 | 131633 | - | VFDB | 007 | - | - | 4 | Unknown |
| pilH | NP\_249100 | (pilH) twitching motility protein PilH [Type IV pili (VF0082)] [Pseudomonas aeruginosa PAO1] | 90.44 | 100.00 | NaN | 131677 | 132042 | - | VFDB | 007 | - | - | 4 | Unknown |
| pilG | NP\_249099 | (pilG) twitching motility protein PilG [Type IV pili (VF0082)] [Pseudomonas aeruginosa PAO1] | 90.13 | 96.81 | NaN | 132103 | 132497 | - | VFDB | 007 | - | - | 4 | Unknown |
| waaC | NP\_253698 | (waaC) 3-deoxy-D-manno-octulosonic-acid (KDO) transferase [LPS (VF0085)] [Pseudomonas aeruginosa PAO1] | 81.85 | 99.44 | NaN | 249893 | 250956 | + | VFDB | 007 | - | - | 6 | Unknown |
| algA | NP\_252241 | (algA) phosphomannose isomerase / guanosine 5'-diphospho-D-mannose pyrophosphorylase [Alginate (VF0091)] [Pseudomonas aeruginosa PAO1] | 87.11 | 96.68 | NaN | 199511 | 200915 | - | VFDB | 007 | - | - | 9 | Unknown |
| vgrG1b | NP\_248785 | (vgrG1b) type VI secretion system substrate VgrG1b [HSI-1 (Hcp-secretion island 1) (SS178)] [Pseudomonas aeruginosa PAO1] | 83.11 | 98.65 | NaN | 414297 | 416500 | + | VFDB | 007 | - | - | 3 | Unknown |
| pvdH | NP\_251103 | (pvdH) diaminobutyrate-2-oxoglutarate aminotransferase PvdH [pyoverdine (IA001)] [Pseudomonas aeruginosa PAO1] | 84.53 | 98.79 | NaN | 502232 | 503626 | - | VFDB | 007 | - | - | 3 | Unknown |
| vgrG1a | NP\_248781 | (vgrG1a) type VI secretion system substrate VgrG1 [HSI-I (VF0334)] [Pseudomonas aeruginosa PAO1] | 80.99 | 99.59 | NaN | 403871 | 405800 | + | VFDB | 007 | - | - | 3 | Unknown |
| pvdL | NP\_251114 | (pvdL) peptide synthase PvdL [pyoverdine (IA001)] [Pseudomonas aeruginosa PAO1] | 80.31 | 99.23 | NaN | 23093 | 36111 | + | VFDB | 007 | - | - | 11 | Unknown |
| algF | NP\_252240 | (algF) alginate o-acetyltransferase AlgF [Alginate (VF0091)] [Pseudomonas aeruginosa PAO1] | 83.64 | 91.71 | NaN | 201065 | 201662 | - | VFDB | 007 | - | - | 9 | Unknown |
| pvdS | NP\_251116 | (pvdS) extracytoplasmic-function sigma-70 factor [Pyoverdine (VF0094)] [Pseudomonas aeruginosa PAO1] | 83.20 | 91.31 | NaN | 21114 | 21631 | - | VFDB | 007 | - | - | 11 | Unknown |
| mbtH-like | NP\_251102 | (mbtH-like) MbtH-like protein from the pyoverdine cluster [pyoverdine (IA001)] [Pseudomonas aeruginosa PAO1] | 85.00 | 91.32 | NaN | 22033 | 22232 | + | VFDB | 007 | - | - | 11 | Unknown |
| algB | NP\_254170 | (algB) two-component response regulator AlgB [Alginate (VF0091)] [Pseudomonas aeruginosa PAO1] | 84.47 | 98.22 | NaN | 251847 | 253172 | - | VFDB | 007 | - | - | 10 | Unknown |
| algD | NP\_252230 | (algD) GDP-mannose 6-dehydrogenase AlgD [Alginate (VF0091)] [Pseudomonas aeruginosa PAO1] | 84.87 | 99.54 | NaN | 214415 | 215724 | - | VFDB | 007 | - | - | 9 | Unknown |
| alg8 | NP\_252231 | (alg8) alginate-c5-mannuronan-epimerase AlgG [Alginate (VF0091)] [Pseudomonas aeruginosa PAO1] | 85.18 | 99.06 | NaN | 212822 | 214292 | - | VFDB | 007 | - | - | 9 | Unknown |
| algI | NP\_252238 | (algI) alginate o-acetyltransferase AlgI [Alginate (VF0091)] [Pseudomonas aeruginosa PAO1] | 87.08 | 99.55 | NaN | 202902 | 204458 | - | VFDB | 007 | - | - | 9 | Unknown |
| pilT | NP\_249086 | (pilT) twitching motility protein PilT [Type IV pili (VF0082)] [Pseudomonas aeruginosa PAO1] | 89.16 | 99.81 | NaN | 143013 | 144045 | - | VFDB | 007 | - | - | 4 | Unknown |
| fepA | NP\_752600 | (fepA) ferrienterobactin outer membrane transporter [Enterobactin (VF0228)] [Escherichia coli CFT073] | 96.74 | 100.00 | NaN | 1031 | 3271 | - | VFDB | 008 | ecoli\_achtman\_4 | 4981 | 14 | Escherichia coli |
| fes | NP\_752602 | (fes) enterobactin/ferric enterobactin esterase [enterobactin (IA019)] [Escherichia coli CFT073] | 96.67 | 100.00 | NaN | 3514 | 4716 | + | VFDB | 008 | ecoli\_achtman\_4 | 4981 | 14 | Escherichia coli |
| entF | NP\_752604 | (entF) enterobactin synthase multienzyme complex component ATP-dependent [Enterobactin (VF0228)] [Escherichia coli CFT073] | 95.69 | 99.28 | NaN | 4934 | 8787 | + | VFDB | 008 | ecoli\_achtman\_4 | 4981 | 14 | Escherichia coli |
| espX4 | NP\_290672 | (espX4) Type III secretion system effector EspX4 [LEE encoded T3SS (SS020)] [Escherichia coli O157:H7 str. EDL933] | 93.30 | 99.94 | NaN | 164372 | 165952 | - | VFDB | 008 | ecoli\_achtman\_4 | 4981 | 7 | Escherichia coli |
| espX5 | NP\_290699 | (espX5) Type III secretion system effector EspX5 [LEE encoded T3SS (SS020)] [Escherichia coli O157:H7 str. EDL933] | 97.14 | 100.00 | NaN | 133581 | 134873 | + | VFDB | 008 | ecoli\_achtman\_4 | 4981 | 7 | Escherichia coli |
| espX1 | NP\_285716 | (espX1) Type III secretion system effector EspX1 [LEE encoded T3SS (SS020)] [Escherichia coli O157:H7 str. EDL933] | 95.15 | 100.00 | NaN | 203438 | 204859 | + | VFDB | 008 | ecoli\_achtman\_4 | 4981 | 5 | Escherichia coli |
| espY1 | NP\_285753 | (espY1) Type III secretion system effector EspY1 [LEE encoded T3SS (SS020)] [Escherichia coli O157:H7 str. EDL933] | 86.96 | 99.87 | NaN | 164483 | 165294 | - | VFDB | 008 | ecoli\_achtman\_4 | 4981 | 5 | Escherichia coli |
| espL1 | NP\_288154 | (espL1) Type III secretion system effector espL1 [LEE encoded T3SS (SS020)] [Escherichia coli O157:H7 str. EDL933] | 97.53 | 100.00 | NaN | 211020 | 212919 | + | VFDB | 008 | ecoli\_achtman\_4 | 4981 | 3 | Escherichia coli |
| ompA | AAF37887 | (ompA) outer membrane protein A [OmpA (VF0236)] [Escherichia coli O18:K1:H7 str. RS218] | 98.08 | 100.00 | NaN | 73759 | 74799 | + | VFDB | 008 | ecoli\_achtman\_4 | 4981 | 1 | Escherichia coli |
| entD | NP\_752599 | (entD) phosphopantetheinyl transferase component of enterobactin synthase multienzyme complex [Enterobactin (VF0228)] [Escherichia coli CFT073] | 95.20 | 100.00 | NaN | 236 | 1006 | - | VFDB | 008 | ecoli\_achtman\_4 | 4981 | 14 | Escherichia coli |
| fepB | NP\_752610 | (fepB) ferrienterobactin ABC transporter periplasmic binding protein [Enterobactin (VF0228)] [Escherichia coli CFT073] | 97.49 | 100.00 | NaN | 14331 | 15287 | - | VFDB | 008 | ecoli\_achtman\_4 | 4981 | 14 | Escherichia coli |
| fepC | NP\_752606 | (fepC) ferrienterobactin ABC transporter ATPase [Enterobactin (VF0228)] [Escherichia coli CFT073] | 97.30 | 100.00 | NaN | 10161 | 10976 | - | VFDB | 008 | ecoli\_achtman\_4 | 4981 | 14 | Escherichia coli |
| entC | NP\_752611 | (entC) isochorismate synthase 1 [Enterobactin (VF0228)] [Escherichia coli CFT073] | 97.90 | 100.00 | NaN | 15650 | 16837 | + | VFDB | 008 | ecoli\_achtman\_4 | 4981 | 14 | Escherichia coli |
| entE | NP\_752612 | (entE) 23-dihydroxybenzoate-AMP ligase component of enterobactin synthase multienzyme complex [Enterobactin (VF0228)] [Escherichia coli CFT073] | 95.47 | 100.00 | NaN | 16847 | 18457 | + | VFDB | 008 | ecoli\_achtman\_4 | 4981 | 14 | Escherichia coli |
| entB | NP\_752613 | (entB) isochorismatase [Enterobactin (VF0228)] [Escherichia coli CFT073] | 98.02 | 100.00 | NaN | 18471 | 19328 | + | VFDB | 008 | ecoli\_achtman\_4 | 4981 | 14 | Escherichia coli |
| entA | NP\_752614 | (entA) 23-dihydro-23-dihydroxybenzoate dehydrogenase [Enterobactin (VF0228)] [Escherichia coli CFT073] | 95.72 | 100.00 | NaN | 19328 | 20074 | + | VFDB | 008 | ecoli\_achtman\_4 | 4981 | 14 | Escherichia coli |
| fyuA | NP\_405467 | (fyuA) pesticin/yersiniabactin receptor protein [Yersiniabactin (VF0136)] [Yersinia pestis CO92] | 99.90 | 100.00 | NaN | 18220 | 20241 | - | VFDB | 008 | ecoli\_achtman\_4 | 4981 | 16 | Escherichia coli |
| ybtE | NP\_405468 | (ybtE) yersiniabactin siderophore biosynthetic protein [Yersiniabactin (VF0136)] [Yersinia pestis CO92] | 99.81 | 100.00 | NaN | 20372 | 21949 | - | VFDB | 008 | ecoli\_achtman\_4 | 4981 | 16 | Escherichia coli |
| ybtT | NP\_405469 | (ybtT) yersiniabactin biosynthetic protein YbtT [Yersiniabactin (VF0136)] [Yersinia pestis CO92] | 99.75 | 100.00 | NaN | 21953 | 22756 | - | VFDB | 008 | ecoli\_achtman\_4 | 4981 | 16 | Escherichia coli |
| fepD | NP\_752608 | (fepD) ferrienterobactin ABC transporter permease [Enterobactin (VF0228)] [Escherichia coli CFT073] | 96.07 | 100.00 | NaN | 11962 | 12978 | - | VFDB | 008 | ecoli\_achtman\_4 | 4981 | 14 | Escherichia coli |
| irp1 | NP\_405471 | (irp1) yersiniabactin biosynthetic protein Irp1 [Yersiniabactin (VF0136)] [Yersinia pestis CO92] | 99.27 | 100.00 | NaN | 23850 | 33341 | - | VFDB | 008 | ecoli\_achtman\_4 | 4981 | 16 | Escherichia coli |
| irp2 | NP\_405472 | (irp2) yersiniabactin biosynthetic protein Irp2 [Yersiniabactin (VF0136)] [Yersinia pestis CO92] | 99.75 | 100.00 | NaN | 33429 | 39536 | - | VFDB | 008 | ecoli\_achtman\_4 | 4981 | 16 | Escherichia coli |
| ybtA | NP\_405473 | (ybtA) transcriptional regulator YbtA [Yersiniabactin (VF0136)] [Yersinia pestis CO92] | 99.48 | 100.00 | NaN | 39727 | 40686 | - | VFDB | 008 | ecoli\_achtman\_4 | 4981 | 16 | Escherichia coli |
| ybtP | NP\_405474 | (ybtP) lipoprotein inner membrane ABC-transporter [Yersiniabactin (VF0136)] [Yersinia pestis CO92] | 99.61 | 100.00 | NaN | 40853 | 42655 | + | VFDB | 008 | ecoli\_achtman\_4 | 4981 | 16 | Escherichia coli |
| ybtQ | NP\_405475 | (ybtQ) inner membrane ABC-transporter YbtQ [Yersiniabactin (VF0136)] [Yersinia pestis CO92] | 99.67 | 100.00 | NaN | 42642 | 44444 | + | VFDB | 008 | ecoli\_achtman\_4 | 4981 | 16 | Escherichia coli |
| ybtX | NP\_405476 | (ybtX) putative signal transducer [Yersiniabactin (VF0136)] [Yersinia pestis CO92] | 99.69 | 100.00 | NaN | 44437 | 45717 | + | VFDB | 008 | ecoli\_achtman\_4 | 4981 | 16 | Escherichia coli |
| entS | NP\_752609 | (entS) enterobactin exporter iron-regulated [enterobactin (IA019)] [Escherichia coli CFT073] | 95.44 | 100.00 | NaN | 13077 | 14327 | + | VFDB | 008 | ecoli\_achtman\_4 | 4981 | 14 | Escherichia coli |
| ybtS | NP\_405477 | (ybtS) salicylate synthase Irp9 [Yersiniabactin (VF0136)] [Yersinia pestis CO92] | 99.92 | 100.00 | NaN | 45745 | 47049 | + | VFDB | 008 | ecoli\_achtman\_4 | 4981 | 16 | Escherichia coli |
| aslA | AAG10151 | (aslA) putative arylsulfatase [AslA (VF0238)] [Escherichia coli O18:K1:H7 str. RS218] | 93.56 | 100.00 | NaN | 49193 | 50621 | + | VFDB | 008 | ecoli\_achtman\_4 | 4981 | 18 | Escherichia coli |
| fepG | NP\_752607 | (fepG) iron-enterobactin ABC transporter permease [Enterobactin (VF0228)] [Escherichia coli CFT073] | 94.06 | 100.00 | NaN | 10973 | 11965 | - | VFDB | 008 | ecoli\_achtman\_4 | 4981 | 14 | Escherichia coli |
| fdeC | YP\_002390132 | (fdeC) adhesin FdeC [FdeC (VF0506)] [Escherichia coli O45:K1:H7 str. S88] | 94.21 | 99.88 | NaN | 19806 | 24054 | + | VFDB | 008 | ecoli\_achtman\_4 | 4981 | 51 | Escherichia coli |
| ykgK/ecpR | NP\_286011 | (ykgK/ecpR) regulator protein EcpR [ECP (VF0404)] [Escherichia coli O157:H7 str. EDL933] | 96.95 | 100.00 | NaN | 16194 | 16784 | - | VFDB | 008 | ecoli\_achtman\_4 | 4981 | 51 | Escherichia coli |
| yagZ/ecpA | NP\_286010 | (yagZ/ecpA) E. coli common pilus structural subunit EcpA [ECP (VF0404)] [Escherichia coli O157:H7 str. EDL933] | 99.15 | 100.00 | NaN | 15532 | 16119 | - | VFDB | 008 | ecoli\_achtman\_4 | 4981 | 51 | Escherichia coli |
| yagY/ecpB | NP\_286009 | (yagY/ecpB) E. coli common pilus chaperone EcpB [ECP (VF0404)] [Escherichia coli O157:H7 str. EDL933] | 98.06 | 100.00 | NaN | 14806 | 15474 | - | VFDB | 008 | ecoli\_achtman\_4 | 4981 | 51 | Escherichia coli |
| yagX/ecpC | NP\_286008 | (yagX/ecpC) E. coli common pilus usher EcpC [ECP (VF0404)] [Escherichia coli O157:H7 str. EDL933] | 98.77 | 100.00 | NaN | 12255 | 14780 | - | VFDB | 008 | ecoli\_achtman\_4 | 4981 | 51 | Escherichia coli |
| espR4 | NP\_288396 | (espR4) Type III secretion system effector espR4 [LEE encoded T3SS (SS020)] [Escherichia coli O157:H7 str. EDL933] | 84.31 | 99.56 | NaN | 80159 | 81294 | + | VFDB | 008 | ecoli\_achtman\_4 | 4981 | 16 | Escherichia coli |
| yagW/ecpD | NP\_286007 | (yagW/ecpD) polymerized tip adhesin of ECP fibers [ECP (VF0404)] [Escherichia coli O157:H7 str. EDL933] | 99.09 | 100.00 | NaN | 10622 | 12265 | - | VFDB | 008 | ecoli\_achtman\_4 | 4981 | 51 | Escherichia coli |
| espL4 | NP\_290644 | (espL4) Type III secretion system effector EspL4 [LEE encoded T3SS (SS020)] [Escherichia coli O157:H7 str. EDL933] | 97.30 | 100.00 | NaN | 19492 | 21678 | + | VFDB | 008 | ecoli\_achtman\_4 | 4981 | 48 | Escherichia coli |
| espR1 | NP\_287686 | (espR1) Type III secretion system effector espR1 [LEE encoded T3SS (SS020)] [Escherichia coli O157:H7 str. EDL933] | 93.84 | 83.65 | NaN | 15337 | 16390 | - | VFDB | 008 | ecoli\_achtman\_4 | 4981 | 40 | Escherichia coli |
| csgG | NP\_460110 | (csgG) curli production assembly/transport protein CsgG [Agf (VF0103)] [Salmonella enterica subsp. enterica serovar Typhimurium str. LT2] | 83.45 | 100.00 | NaN | 43333 | 44166 | + | VFDB | 008 | ecoli\_achtman\_4 | 4981 | 34 | Escherichia coli |
| csgF | NP\_460111 | (csgF) curli production assembly/transport protein CsgF [Agf (VF0103)] [Salmonella enterica subsp. enterica serovar Typhimurium str. LT2] | 81.23 | 99.04 | NaN | 42890 | 43306 | + | VFDB | 008 | ecoli\_achtman\_4 | 4981 | 34 | Escherichia coli |
| csgD | NP\_460113 | (csgD) DNA-binding transcriptional regulator CsgD [curli fibers/thin aggregative fimbriae (AGF) (AI094)] [Salmonella enterica subsp. enterica serovar Typhimurium str. LT2] | 81.11 | 100.00 | NaN | 41821 | 42471 | + | VFDB | 008 | ecoli\_achtman\_4 | 4981 | 34 | Escherichia coli |
| csgB | NP\_460114 | (csgB) minor curlin subunit precursor curli nucleator protein CsgB [Agf (VF0103)] [Salmonella enterica subsp. enterica serovar Typhimurium str. LT2] | 83.81 | 99.78 | NaN | 40611 | 41066 | - | VFDB | 008 | ecoli\_achtman\_4 | 4981 | 34 | Escherichia coli |
| yagV/ecpE | NP\_286006 | (yagV/ecpE) E. coli common pilus chaperone EcpE [ECP (VF0404)] [Escherichia coli O157:H7 str. EDL933] | 97.08 | 99.74 | NaN | 9945 | 10698 | - | VFDB | 008 | ecoli\_achtman\_4 | 4981 | 51 | Escherichia coli |
| ybtU | NP\_405470 | (ybtU) yersiniabactin biosynthetic protein YbtU [Yersiniabactin (VF0136)] [Yersinia pestis CO92] | 100.00 | 100.00 | NaN | 22753 | 23853 | - | VFDB | 008 | ecoli\_achtman\_4 | 4981 | 16 | Escherichia coli |
| sgrA | ZP\_00602747 | (sgrA) cell wall anchored protein SgrA [SgrA (VF0540)] [Enterococcus faecium DO] | 100.00 | 100.00 | NaN | 17698 | 18672 | - | VFDB | 009 | efaecium | 80 | 42 | Enterococcus faecium |
| acm | AAN12397 | (acm) collagen adhesin precursor Acm [Acm (VF0419)] [Enterococcus faecium str. TX2555] | 100.00 | 100.00 | NaN | 3344 | 5509 | + | VFDB | 009 | efaecium | 80 | 17 | Enterococcus faecium |
| fss3 | NP\_815578 | (fss3) Enterococcus faecalis surface protein Fss3 fibrinogen binding protein [Fibrinogen binding protein (AI273)] [Enterococcus faecalis V583] | 90.92 | 98.27 | NaN | 635 | 3806 | + | VFDB | 009 | efaecium | 80 | 44 | Enterococcus faecium |
| acm | AAN12397 | (acm) collagen adhesin precursor Acm [Acm (VF0419)] [Enterococcus faecium str. TX2555] | 100.00 | 100.00 | NaN | 73689 | 75854 | - | VFDB | 010 | efaecium | 117 | 2 | Enterococcus faecium |
| fss3 | NP\_815578 | (fss3) Enterococcus faecalis surface protein Fss3 fibrinogen binding protein [Fibrinogen binding protein (AI273)] [Enterococcus faecalis V583] | 90.70 | 98.27 | NaN | 119193 | 122364 | + | VFDB | 010 | efaecium | 117 | 4 | Enterococcus faecium |
| scm | ZP\_00604835 | (scm) collagen adhesin protein Scm [Scm (VF0418)] [Enterococcus faecium DO] | 96.17 | 80.38 | NaN | 16927 | 18520 | - | VFDB | 010 | efaecium | 117 | 13 | Enterococcus faecium |
| sgrA | ZP\_00602747 | (sgrA) cell wall anchored protein SgrA [SgrA (VF0540)] [Enterococcus faecium DO] | 99.90 | 100.00 | NaN | 3907 | 4881 | + | VFDB | 010 | efaecium | 117 | 26 | Enterococcus faecium |
| ecbA | ZP\_00603098 | (ecbA) Collagen binding MSCRAMM EcbA [EcbA (VF0539)] [Enterococcus faecium DO] | 100.00 | 100.00 | NaN | 5800 | 9027 | - | VFDB | 010 | efaecium | 117 | 34 | Enterococcus faecium |
| ecbA | ZP\_00603098 | (ecbA) Collagen binding MSCRAMM EcbA [EcbA (VF0539)] [Enterococcus faecium DO] | 100.00 | 100.00 | NaN | 5800 | 9027 | - | VFDB | 011 | efaecium | 117 | 69 | Enterococcus faecium |
| sgrA | ZP\_00602747 | (sgrA) cell wall anchored protein SgrA [SgrA (VF0540)] [Enterococcus faecium DO] | 99.90 | 100.00 | NaN | 3910 | 4884 | + | VFDB | 011 | efaecium | 117 | 26 | Enterococcus faecium |
| acm | AAN12397 | (acm) collagen adhesin precursor Acm [Acm (VF0419)] [Enterococcus faecium str. TX2555] | 100.00 | 100.00 | NaN | 73689 | 75854 | - | VFDB | 011 | efaecium | 117 | 12 | Enterococcus faecium |
| fss3 | NP\_815578 | (fss3) Enterococcus faecalis surface protein Fss3 fibrinogen binding protein [Fibrinogen binding protein (AI273)] [Enterococcus faecalis V583] | 90.70 | 98.27 | NaN | 119799 | 122970 | + | VFDB | 011 | efaecium | 117 | 2 | Enterococcus faecium |
| ecbA | ZP\_00603098 | (ecbA) Collagen binding MSCRAMM EcbA [EcbA (VF0539)] [Enterococcus faecium DO] | 100.00 | 100.00 | NaN | 5800 | 9027 | - | VFDB | 012 | efaecium | 117 | 36 | Enterococcus faecium |
| scm | ZP\_00604835 | (scm) collagen adhesin protein Scm [Scm (VF0418)] [Enterococcus faecium DO] | 96.17 | 80.38 | NaN | 16927 | 18520 | - | VFDB | 012 | efaecium | 117 | 12 | Enterococcus faecium |
| sgrA | ZP\_00602747 | (sgrA) cell wall anchored protein SgrA [SgrA (VF0540)] [Enterococcus faecium DO] | 99.90 | 100.00 | NaN | 3907 | 4881 | + | VFDB | 012 | efaecium | 117 | 28 | Enterococcus faecium |
| acm | AAN12397 | (acm) collagen adhesin precursor Acm [Acm (VF0419)] [Enterococcus faecium str. TX2555] | 100.00 | 100.00 | NaN | 73689 | 75854 | - | VFDB | 012 | efaecium | 117 | 2 | Enterococcus faecium |
| fss3 | NP\_815578 | (fss3) Enterococcus faecalis surface protein Fss3 fibrinogen binding protein [Fibrinogen binding protein (AI273)] [Enterococcus faecalis V583] | 90.70 | 98.27 | NaN | 66441 | 69612 | + | VFDB | 012 | efaecium | 117 | 11 | Enterococcus faecium |
| ykgK/ecpR | NP\_286011 | (ykgK/ecpR) regulator protein EcpR [ECP (VF0404)] [Escherichia coli O157:H7 str. EDL933] | 97.29 | 100.00 | NaN | 10053 | 10643 | + | VFDB | 014 | ecoli\_achtman\_4 | 90 | 22 | Escherichia coli |
| csgG | NP\_460110 | (csgG) curli production assembly/transport protein CsgG [Agf (VF0103)] [Salmonella enterica subsp. enterica serovar Typhimurium str. LT2] | 83.09 | 100.00 | NaN | 3395 | 4228 | - | VFDB | 014 | ecoli\_achtman\_4 | 90 | 9 | Escherichia coli |
| csgF | NP\_460111 | (csgF) curli production assembly/transport protein CsgF [Agf (VF0103)] [Salmonella enterica subsp. enterica serovar Typhimurium str. LT2] | 81.23 | 99.04 | NaN | 4255 | 4671 | - | VFDB | 014 | ecoli\_achtman\_4 | 90 | 9 | Escherichia coli |
| csgD | NP\_460113 | (csgD) DNA-binding transcriptional regulator CsgD [curli fibers/thin aggregative fimbriae (AGF) (AI094)] [Salmonella enterica subsp. enterica serovar Typhimurium str. LT2] | 81.11 | 100.00 | NaN | 5090 | 5740 | - | VFDB | 014 | ecoli\_achtman\_4 | 90 | 9 | Escherichia coli |
| csgB | NP\_460114 | (csgB) minor curlin subunit precursor curli nucleator protein CsgB [Agf (VF0103)] [Salmonella enterica subsp. enterica serovar Typhimurium str. LT2] | 83.81 | 99.78 | NaN | 6494 | 6949 | + | VFDB | 014 | ecoli\_achtman\_4 | 90 | 9 | Escherichia coli |
| fdeC | YP\_002390132 | (fdeC) adhesin FdeC [FdeC (VF0506)] [Escherichia coli O45:K1:H7 str. S88] | 94.21 | 99.88 | NaN | 2785 | 7033 | - | VFDB | 014 | ecoli\_achtman\_4 | 90 | 22 | Escherichia coli |
| yagZ/ecpA | NP\_286010 | (yagZ/ecpA) E. coli common pilus structural subunit EcpA [ECP (VF0404)] [Escherichia coli O157:H7 str. EDL933] | 99.32 | 100.00 | NaN | 10718 | 11305 | + | VFDB | 014 | ecoli\_achtman\_4 | 90 | 22 | Escherichia coli |
| gspC | YP\_404599 | (gspC) general secretion pathway protein C [T2SS (VF0333)] [Shigella dysenteriae Sd197] | 94.58 | 100.00 | NaN | 68530 | 69360 | + | VFDB | 014 | ecoli\_achtman\_4 | 90 | 2 | Escherichia coli |
| yagX/ecpC | NP\_286008 | (yagX/ecpC) E. coli common pilus usher EcpC [ECP (VF0404)] [Escherichia coli O157:H7 str. EDL933] | 98.73 | 100.00 | NaN | 12057 | 14582 | + | VFDB | 014 | ecoli\_achtman\_4 | 90 | 22 | Escherichia coli |
| yagW/ecpD | NP\_286007 | (yagW/ecpD) polymerized tip adhesin of ECP fibers [ECP (VF0404)] [Escherichia coli O157:H7 str. EDL933] | 98.66 | 100.00 | NaN | 14572 | 16215 | + | VFDB | 014 | ecoli\_achtman\_4 | 90 | 22 | Escherichia coli |
| yagV/ecpE | NP\_286006 | (yagV/ecpE) E. coli common pilus chaperone EcpE [ECP (VF0404)] [Escherichia coli O157:H7 str. EDL933] | 96.68 | 99.74 | NaN | 16139 | 16892 | + | VFDB | 014 | ecoli\_achtman\_4 | 90 | 22 | Escherichia coli |
| espR1 | NP\_287686 | (espR1) Type III secretion system effector espR1 [LEE encoded T3SS (SS020)] [Escherichia coli O157:H7 str. EDL933] | 94.22 | 83.73 | NaN | 25238 | 26292 | + | VFDB | 014 | ecoli\_achtman\_4 | 90 | 25 | Escherichia coli |
| gspE | YP\_404601 | (gspE) general secretion pathway protein E [T2SS (VF0333)] [Shigella dysenteriae Sd197] | 96.65 | 100.00 | NaN | 71450 | 72943 | + | VFDB | 014 | ecoli\_achtman\_4 | 90 | 2 | Escherichia coli |
| gspD | YP\_404600 | (gspD) general secretion pathway protein D [T2SS (VF0333)] [Shigella dysenteriae Sd197] | 97.46 | 100.00 | NaN | 69600 | 71450 | + | VFDB | 014 | ecoli\_achtman\_4 | 90 | 2 | Escherichia coli |
| espX5 | NP\_290699 | (espX5) Type III secretion system effector EspX5 [LEE encoded T3SS (SS020)] [Escherichia coli O157:H7 str. EDL933] | 95.51 | 100.00 | NaN | 77765 | 79057 | - | VFDB | 014 | ecoli\_achtman\_4 | 90 | 7 | Escherichia coli |
| yagY/ecpB | NP\_286009 | (yagY/ecpB) E. coli common pilus chaperone EcpB [ECP (VF0404)] [Escherichia coli O157:H7 str. EDL933] | 98.80 | 100.00 | NaN | 11363 | 12031 | + | VFDB | 014 | ecoli\_achtman\_4 | 90 | 22 | Escherichia coli |
| espX4 | NP\_290672 | (espX4) Type III secretion system effector EspX4 [LEE encoded T3SS (SS020)] [Escherichia coli O157:H7 str. EDL933] | 93.05 | 99.94 | NaN | 46647 | 48227 | + | VFDB | 014 | ecoli\_achtman\_4 | 90 | 7 | Escherichia coli |
| gspF | YP\_404602 | (gspF) general secretion pathway protein F [T2SS (VF0333)] [Shigella dysenteriae Sd197] | 96.67 | 100.00 | NaN | 72943 | 74142 | + | VFDB | 014 | ecoli\_achtman\_4 | 90 | 2 | Escherichia coli |
| fimH | NP\_757248 | (fimH) FimH protein precursor [Type 1 fimbriae (VF0221)] [Escherichia coli CFT073] | 97.15 | 100.00 | NaN | 37887 | 38798 | + | VFDB | 014 | ecoli\_achtman\_4 | 90 | 6 | Escherichia coli |
| espL1 | NP\_288154 | (espL1) Type III secretion system effector espL1 [LEE encoded T3SS (SS020)] [Escherichia coli O157:H7 str. EDL933] | 98.53 | 100.00 | NaN | 190656 | 192554 | + | VFDB | 014 | ecoli\_achtman\_4 | 90 | 3 | Escherichia coli |
| gspM | YP\_404609 | (gspM) general secretion pathway protein M [T2SS (VF0333)] [Shigella dysenteriae Sd197] | 97.16 | 100.00 | NaN | 78440 | 78862 | + | VFDB | 014 | ecoli\_achtman\_4 | 90 | 2 | Escherichia coli |
| gspL | YP\_404608 | (gspL) general secretion pathway protein L [T2SS (VF0333)] [Shigella dysenteriae Sd197] | 96.86 | 100.00 | NaN | 77464 | 78324 | + | VFDB | 014 | ecoli\_achtman\_4 | 90 | 2 | Escherichia coli |
| gspK | YP\_404607 | (gspK) general secretion pathway protein K [T2SS (VF0333)] [Shigella dysenteriae Sd197] | 97.65 | 100.00 | NaN | 76172 | 77149 | + | VFDB | 014 | ecoli\_achtman\_4 | 90 | 2 | Escherichia coli |
| gspJ | YP\_404606 | (gspJ) general secretion pathway protein J [T2SS (VF0333)] [Shigella dysenteriae Sd197] | 97.19 | 100.00 | NaN | 75606 | 76175 | + | VFDB | 014 | ecoli\_achtman\_4 | 90 | 2 | Escherichia coli |
| gspI | YP\_404605 | (gspI) general secretion pathway protein I [T2SS (VF0333)] [Shigella dysenteriae Sd197] | 95.16 | 100.00 | NaN | 75202 | 75573 | + | VFDB | 014 | ecoli\_achtman\_4 | 90 | 2 | Escherichia coli |
| gspH | YP\_404604 | (gspH) general secretion pathway protein H [T2SS (VF0333)] [Shigella dysenteriae Sd197] | 95.48 | 100.00 | NaN | 74675 | 75205 | + | VFDB | 014 | ecoli\_achtman\_4 | 90 | 2 | Escherichia coli |
| espX1 | NP\_285716 | (espX1) Type III secretion system effector EspX1 [LEE encoded T3SS (SS020)] [Escherichia coli O157:H7 str. EDL933] | 94.58 | 99.93 | NaN | 148566 | 149986 | - | VFDB | 014 | ecoli\_achtman\_4 | 90 | 6 | Escherichia coli |
| entE | NP\_752612 | (entE) 23-dihydroxybenzoate-AMP ligase component of enterobactin synthase multienzyme complex [Enterobactin (VF0228)] [Escherichia coli CFT073] | 95.78 | 100.00 | NaN | 101260 | 102870 | - | VFDB | 014 | ecoli\_achtman\_4 | 90 | 4 | Escherichia coli |
| entA | NP\_752614 | (entA) 23-dihydro-23-dihydroxybenzoate dehydrogenase [Enterobactin (VF0228)] [Escherichia coli CFT073] | 95.98 | 100.00 | NaN | 99643 | 100389 | - | VFDB | 014 | ecoli\_achtman\_4 | 90 | 4 | Escherichia coli |
| gspG | YP\_404603 | (gspG) general secretion pathway protein G [T2SS (VF0333)] [Shigella dysenteriae Sd197] | 96.27 | 100.00 | NaN | 74183 | 74638 | + | VFDB | 014 | ecoli\_achtman\_4 | 90 | 2 | Escherichia coli |
| entC | NP\_752611 | (entC) isochorismate synthase 1 [Enterobactin (VF0228)] [Escherichia coli CFT073] | 97.47 | 100.00 | NaN | 102880 | 104067 | - | VFDB | 014 | ecoli\_achtman\_4 | 90 | 4 | Escherichia coli |
| fepB | NP\_752610 | (fepB) ferrienterobactin ABC transporter periplasmic binding protein [Enterobactin (VF0228)] [Escherichia coli CFT073] | 97.39 | 100.00 | NaN | 104430 | 105386 | + | VFDB | 014 | ecoli\_achtman\_4 | 90 | 4 | Escherichia coli |
| entS | NP\_752609 | (entS) enterobactin exporter iron-regulated [enterobactin (IA019)] [Escherichia coli CFT073] | 95.60 | 100.00 | NaN | 105390 | 106640 | - | VFDB | 014 | ecoli\_achtman\_4 | 90 | 4 | Escherichia coli |
| fepD | NP\_752608 | (fepD) ferrienterobactin ABC transporter permease [Enterobactin (VF0228)] [Escherichia coli CFT073] | 96.36 | 100.00 | NaN | 106739 | 107755 | + | VFDB | 014 | ecoli\_achtman\_4 | 90 | 4 | Escherichia coli |
| entB | NP\_752613 | (entB) isochorismatase [Enterobactin (VF0228)] [Escherichia coli CFT073] | 97.90 | 100.00 | NaN | 100389 | 101246 | - | VFDB | 014 | ecoli\_achtman\_4 | 90 | 4 | Escherichia coli |
| fepC | NP\_752606 | (fepC) ferrienterobactin ABC transporter ATPase [Enterobactin (VF0228)] [Escherichia coli CFT073] | 97.30 | 100.00 | NaN | 108741 | 109556 | + | VFDB | 014 | ecoli\_achtman\_4 | 90 | 4 | Escherichia coli |
| fimG | NP\_757247 | (fimG) FimG protein precursor [Type 1 fimbriae (VF0221)] [Escherichia coli CFT073] | 98.02 | 100.00 | NaN | 37373 | 37876 | + | VFDB | 014 | ecoli\_achtman\_4 | 90 | 6 | Escherichia coli |
| entF | NP\_752604 | (entF) enterobactin synthase multienzyme complex component ATP-dependent [Enterobactin (VF0228)] [Escherichia coli CFT073] | 95.48 | 98.66 | NaN | 110954 | 114783 | - | VFDB | 014 | ecoli\_achtman\_4 | 90 | 4 | Escherichia coli |
| fes | NP\_752602 | (fes) enterobactin/ferric enterobactin esterase [enterobactin (IA019)] [Escherichia coli CFT073] | 96.43 | 100.00 | NaN | 115001 | 116203 | - | VFDB | 014 | ecoli\_achtman\_4 | 90 | 4 | Escherichia coli |
| fepA | NP\_752600 | (fepA) ferrienterobactin outer membrane transporter [Enterobactin (VF0228)] [Escherichia coli CFT073] | 97.50 | 100.00 | NaN | 116446 | 118686 | + | VFDB | 014 | ecoli\_achtman\_4 | 90 | 4 | Escherichia coli |
| entD | NP\_752599 | (entD) phosphopantetheinyl transferase component of enterobactin synthase multienzyme complex [Enterobactin (VF0228)] [Escherichia coli CFT073] | 95.07 | 100.00 | NaN | 118711 | 119481 | + | VFDB | 014 | ecoli\_achtman\_4 | 90 | 4 | Escherichia coli |
| fimF | NP\_757245 | (fimF) FimF protein precursor [Type 1 fimbriae (VF0221)] [Escherichia coli CFT073] | 97.38 | 100.00 | NaN | 36827 | 37360 | + | VFDB | 014 | ecoli\_achtman\_4 | 90 | 6 | Escherichia coli |
| fimD | NP\_757244 | (fimD) Outer membrane usher protein fimD precursor [Type 1 fimbriae (VF0221)] [Escherichia coli CFT073] | 98.22 | 100.00 | NaN | 34184 | 36820 | + | VFDB | 014 | ecoli\_achtman\_4 | 90 | 6 | Escherichia coli |
| fimC | NP\_757243 | (fimC) Chaperone protein fimC precursor [Type 1 fimbriae (VF0221)] [Escherichia coli CFT073] | 98.48 | 100.00 | NaN | 33392 | 34117 | + | VFDB | 014 | ecoli\_achtman\_4 | 90 | 6 | Escherichia coli |
| fimI | NP\_757242 | (fimI) Fimbrin-like protein fimI precursor [Type 1 fimbriae (VF0221)] [Escherichia coli CFT073] | 98.33 | 100.00 | NaN | 32816 | 33355 | + | VFDB | 014 | ecoli\_achtman\_4 | 90 | 6 | Escherichia coli |
| fimA | NP\_757241 | (fimA) Type-1 fimbrial protein A chain precursor [Type 1 fimbriae (VF0221)] [Escherichia coli CFT073] | 92.57 | 100.00 | NaN | 32146 | 32751 | + | VFDB | 014 | ecoli\_achtman\_4 | 90 | 6 | Escherichia coli |
| fimE | NP\_757240 | (fimE) Type 1 fimbriae Regulatory protein fimE [Type 1 fimbriae (VF0221)] [Escherichia coli CFT073] | 98.66 | 100.00 | NaN | 31126 | 31722 | + | VFDB | 014 | ecoli\_achtman\_4 | 90 | 6 | Escherichia coli |
| fepG | NP\_752607 | (fepG) iron-enterobactin ABC transporter permease [Enterobactin (VF0228)] [Escherichia coli CFT073] | 94.46 | 100.00 | NaN | 107752 | 108744 | + | VFDB | 014 | ecoli\_achtman\_4 | 90 | 4 | Escherichia coli |
| fimB | NP\_757239 | (fimB) Type 1 fimbriae Regulatory protein fimB [Type 1 fimbriae (VF0221)] [Escherichia coli CFT073] | 98.18 | 100.00 | NaN | 30046 | 30648 | + | VFDB | 014 | ecoli\_achtman\_4 | 90 | 6 | Escherichia coli |
| ompA | AAF37887 | (ompA) outer membrane protein A [OmpA (VF0236)] [Escherichia coli O18:K1:H7 str. RS218] | 94.49 | 100.00 | NaN | 298764 | 299816 | - | VFDB | 014 | ecoli\_achtman\_4 | 90 | 5 | Escherichia coli |
| lip1 | NP\_248770 | (lip1) lipoprotein [HSI-I (VF0334)] [Pseudomonas aeruginosa PAO1] | 99.57 | 100.00 | NaN | 193165 | 193629 | + | VFDB | 015 | paeruginosa | - | 15 | Pseudomonas aeruginosa |
| icmF1/tssM1 | NP\_248767 | (icmF1/tssM1) type VI secretion system protein IcmF1 [HSI-I (VF0334)] [Pseudomonas aeruginosa PAO1] | 99.79 | 100.00 | NaN | 196557 | 199862 | + | VFDB | 015 | paeruginosa | - | 15 | Pseudomonas aeruginosa |
| dotU1 | NP\_248768 | (dotU1) type VI secretion system protein DotU [HSI-I (VF0334)] [Pseudomonas aeruginosa PAO1] | 99.78 | 100.00 | NaN | 194986 | 196335 | + | VFDB | 015 | paeruginosa | - | 15 | Pseudomonas aeruginosa |
| tagF/pppB | NP\_248766 | (tagF/pppB) Pseudomonas protein phosphatase PppB [HSI-I (VF0334)] [Pseudomonas aeruginosa PAO1] | 100.00 | 100.00 | NaN | 199859 | 200539 | + | VFDB | 015 | paeruginosa | - | 15 | Pseudomonas aeruginosa |
| pppA | NP\_248765 | (pppA) Pseudomonas protein phosphatase PppA [HSI-I (VF0334)] [Pseudomonas aeruginosa PAO1] | 99.73 | 100.00 | NaN | 200549 | 201277 | + | VFDB | 015 | paeruginosa | - | 15 | Pseudomonas aeruginosa |
| phzB1 | NP\_252900 | (phzB1) phenazine biosynthesis protein PhzB [Phenazines biosynthesis (CVF536)] [Pseudomonas aeruginosa PAO1] | 98.77 | 100.00 | NaN | 289 | 777 | - | VFDB | 015 | paeruginosa | - | 16 | Pseudomonas aeruginosa |
| phzA1 | NP\_252899 | (phzA1) phenazine biosynthesis protein PhzA [Phenazines biosynthesis (CVF536)] [Pseudomonas aeruginosa PAO1] | 99.80 | 100.00 | NaN | 807 | 1295 | - | VFDB | 015 | paeruginosa | - | 16 | Pseudomonas aeruginosa |
| phzM | NP\_252898 | (phzM) phenazine-specific methyltransferase PhzM (adenosylmethionine dependent methyltransferase) [Pyocyanin (VF0100)] [Pseudomonas aeruginosa PAO1] | 99.70 | 100.00 | NaN | 1991 | 2995 | + | VFDB | 015 | paeruginosa | - | 16 | Pseudomonas aeruginosa |
| algW | NP\_253136 | (algW) AlgW protein [Alginate regulation (CVF523)] [Pseudomonas aeruginosa PAO1] | 100.00 | 99.83 | NaN | 37058 | 38225 | - | VFDB | 015 | paeruginosa | - | 17 | Pseudomonas aeruginosa |
| rhlI | NP\_252166 | (rhlI) autoinducer synthesis protein RhlL [Quorum sensing (VF0093)] [Pseudomonas aeruginosa PAO1] | 98.35 | 100.00 | NaN | 45304 | 45909 | - | VFDB | 015 | paeruginosa | - | 19 | Pseudomonas aeruginosa |
| rhlA | NP\_252169 | (rhlA) rhamnosyltransferase chain A [Rhamnolipid (VF0089)] [Pseudomonas aeruginosa PAO1] | 99.55 | 100.00 | NaN | 48285 | 49172 | - | VFDB | 015 | paeruginosa | - | 19 | Pseudomonas aeruginosa |
| hsiJ1 | NP\_248769 | (hsiJ1) type VI secretion system hcp secretion island protein HsiJ1 [HSI-I (VF0334)] [Pseudomonas aeruginosa PAO1] | 99.48 | 100.00 | NaN | 193645 | 194979 | + | VFDB | 015 | paeruginosa | - | 15 | Pseudomonas aeruginosa |
| rhlB | NP\_252168 | (rhlB) rhamnosyltransferase chain B [Rhamnolipid (VF0089)] [Pseudomonas aeruginosa PAO1] | 99.22 | 100.00 | NaN | 46939 | 48219 | - | VFDB | 015 | paeruginosa | - | 19 | Pseudomonas aeruginosa |
| vgrG1b | NP\_248785 | (vgrG1b) type VI secretion system substrate VgrG1b [HSI-1 (Hcp-secretion island 1) (SS178)] [Pseudomonas aeruginosa PAO1] | 99.28 | 100.00 | NaN | 173823 | 176048 | - | VFDB | 015 | paeruginosa | - | 15 | Pseudomonas aeruginosa |
| hsiA1 | NP\_248772 | (hsiA1) type VI secretion system hcp secretion island protein HsiA1 [HSI-I (VF0334)] [Pseudomonas aeruginosa PAO1] | 99.03 | 100.00 | NaN | 190189 | 191223 | - | VFDB | 015 | paeruginosa | - | 15 | Pseudomonas aeruginosa |
| tse3 | NP\_252174 | (tse3) type VI secretion system effector Tse3 glycoside hydrolase [HSI-1 (Hcp-secretion island 1) (SS178)] [Pseudomonas aeruginosa PAO1] | 99.51 | 100.00 | NaN | 54442 | 55668 | + | VFDB | 015 | paeruginosa | - | 19 | Pseudomonas aeruginosa |
| pilS | NP\_253236 | (pilS) two-component sensor PilS [Type IV pili (VF0082)] [Pseudomonas aeruginosa PAO1] | 99.25 | 100.00 | NaN | 75167 | 76759 | + | VFDB | 015 | paeruginosa | - | 13 | Pseudomonas aeruginosa |
| pilR | NP\_253237 | (pilR) two-component response regulator PilR [Type IV pili (VF0082)] [Pseudomonas aeruginosa PAO1] | 99.85 | 100.00 | NaN | 76774 | 78111 | + | VFDB | 015 | paeruginosa | - | 13 | Pseudomonas aeruginosa |
| fimT | NP\_253239 | (fimT) type 4 fimbrial biogenesis protein FimT [Type IV pili (VF0082)] [Pseudomonas aeruginosa PAO1] | 99.61 | 100.00 | NaN | 79386 | 79895 | + | VFDB | 015 | paeruginosa | - | 13 | Pseudomonas aeruginosa |
| fimU | NP\_253240 | (fimU) type 4 fimbrial biogenesis protein FimU [Type IV pili (VF0082)] [Pseudomonas aeruginosa PAO1] | 99.80 | 100.00 | NaN | 80001 | 80507 | + | VFDB | 015 | paeruginosa | - | 13 | Pseudomonas aeruginosa |
| pilV | NP\_253241 | (pilV) type IV pilus biogenesis protein PilV [Type IV pili (VF0082)] [Pseudomonas aeruginosa PAO1] | 99.64 | 100.00 | NaN | 80498 | 81055 | + | VFDB | 015 | paeruginosa | - | 13 | Pseudomonas aeruginosa |
| pilW | NP\_253242 | (pilW) type IV fimbrial biogenesis protein PilW [Type IV pili (VF0082)] [Pseudomonas aeruginosa PAO1] | 99.64 | 100.00 | NaN | 81052 | 81876 | + | VFDB | 015 | paeruginosa | - | 13 | Pseudomonas aeruginosa |
| pilX | NP\_253243 | (pilX) type 4 fimbrial biogenesis protein PilX [Type IV pili (VF0082)] [Pseudomonas aeruginosa PAO1] | 100.00 | 100.00 | NaN | 81873 | 82460 | + | VFDB | 015 | paeruginosa | - | 13 | Pseudomonas aeruginosa |
| pilY1 | NP\_253244 | (pilY1) type 4 fimbrial biogenesis protein PilY1 [Type IV pili (VF0082)] [Pseudomonas aeruginosa PAO1] | 91.95 | 99.66 | NaN | 82472 | 85963 | + | VFDB | 015 | paeruginosa | - | 13 | Pseudomonas aeruginosa |
| pilY2 | NP\_253245 | (pilY2) type 4 fimbrial biogenesis protein PilY2 [Type IV pili (VF0082)] [Pseudomonas aeruginosa PAO1] | 99.71 | 100.00 | NaN | 85965 | 86312 | + | VFDB | 015 | paeruginosa | - | 13 | Pseudomonas aeruginosa |
| pilE | NP\_253246 | (pilE) type 4 fimbrial biogenesis protein PilE [Type IV pili (VF0082)] [Pseudomonas aeruginosa PAO1] | 99.77 | 100.00 | NaN | 86309 | 86734 | + | VFDB | 015 | paeruginosa | - | 13 | Pseudomonas aeruginosa |
| tse2 | NP\_251392 | (tse2) type VI secretion system effector Tse2 [HSI-1 (Hcp-secretion island 1) (SS178)] [Pseudomonas aeruginosa PAO1] | 99.58 | 100.00 | NaN | 116750 | 117226 | - | VFDB | 015 | paeruginosa | - | 14 | Pseudomonas aeruginosa |
| vgrG1a | NP\_248781 | (vgrG1a) type VI secretion system substrate VgrG1 [HSI-I (VF0334)] [Pseudomonas aeruginosa PAO1] | 99.95 | 100.00 | NaN | 178440 | 180371 | - | VFDB | 015 | paeruginosa | - | 15 | Pseudomonas aeruginosa |
| clpV1 | NP\_248780 | (clpV1) type VI secretion system AAA+ family ATPase [HSI-I (VF0334)] [Pseudomonas aeruginosa PAO1] | 99.63 | 100.00 | NaN | 180418 | 183126 | - | VFDB | 015 | paeruginosa | - | 15 | Pseudomonas aeruginosa |
| hsiH1 | NP\_248779 | (hsiH1) type VI secretion system hcp secretion island protein HsiH1 [HSI-I (VF0334)] [Pseudomonas aeruginosa PAO1] | 99.90 | 100.00 | NaN | 183119 | 184165 | - | VFDB | 015 | paeruginosa | - | 15 | Pseudomonas aeruginosa |
| hsiG1 | NP\_248778 | (hsiG1) type VI secretion system hcp secretion island protein HsiG1 [HSI-I (VF0334)] [Pseudomonas aeruginosa PAO1] | 99.78 | 100.00 | NaN | 184129 | 185988 | - | VFDB | 015 | paeruginosa | - | 15 | Pseudomonas aeruginosa |
| hsiF1 | NP\_248777 | (hsiF1) type VI secretion system hcp secretion island protein HsiF1 a gp25-like protein but not exhibit lysozyme activity [HSI-I (VF0334)] [Pseudomonas aeruginosa PAO1] | 99.80 | 100.00 | NaN | 185985 | 186494 | - | VFDB | 015 | paeruginosa | - | 15 | Pseudomonas aeruginosa |
| hsiE1 | NP\_248776 | (hsiE1) type VI secretion system hcp secretion island protein HsiE1 interacting with HsiB1 to form a novel subcomplex of the T6SS [HSI-I (VF0334)] [Pseudomonas aeruginosa PAO1] | 99.76 | 100.00 | NaN | 186496 | 187341 | - | VFDB | 015 | paeruginosa | - | 15 | Pseudomonas aeruginosa |
| hcp1 | NP\_248775 | (hcp1) type VI secretion system substrate Hcp1 [HSI-I (VF0334)] [Pseudomonas aeruginosa PAO1] | 100.00 | 100.00 | NaN | 187509 | 187997 | - | VFDB | 015 | paeruginosa | - | 15 | Pseudomonas aeruginosa |
| hsiC1/vipB | NP\_248774 | (hsiC1/vipB) type VI secretion system tubule-forming protein VipB [HSI-I (VF0334)] [Pseudomonas aeruginosa PAO1] | 99.80 | 100.00 | NaN | 188073 | 189569 | - | VFDB | 015 | paeruginosa | - | 15 | Pseudomonas aeruginosa |
| hsiB1/vipA | NP\_248773 | (hsiB1/vipA) type VI secretion system tubule-forming protein VipA [HSI-I (VF0334)] [Pseudomonas aeruginosa PAO1] | 99.81 | 100.00 | NaN | 189582 | 190100 | - | VFDB | 015 | paeruginosa | - | 15 | Pseudomonas aeruginosa |
| fha1 | NP\_248771 | (fha1) type VI secretion system forkhead-associated protein Fha1 [HSI-I (VF0334)] [Pseudomonas aeruginosa PAO1] | 97.52 | 100.00 | NaN | 191591 | 193120 | + | VFDB | 015 | paeruginosa | - | 15 | Pseudomonas aeruginosa |
| motY | NP\_252216 | (motY) probable outer membrane protein precursor [Deoxyhexose linking sugar 209 Da capping structure (AI138)] [Pseudomonas aeruginosa PAO1] | 100.00 | 100.00 | NaN | 78489 | 79454 | - | VFDB | 015 | paeruginosa | - | 19 | Pseudomonas aeruginosa |
| pchI | NP\_252912 | (pchI) ABC transporter ATP-binding protein [Pyochelin (VF0095)] [Pseudomonas aeruginosa PAO1] | 99.71 | 100.00 | NaN | 60048 | 61772 | + | VFDB | 015 | paeruginosa | - | 23 | Pseudomonas aeruginosa |
| alg8 | NP\_252231 | (alg8) alginate-c5-mannuronan-epimerase AlgG [Alginate (VF0091)] [Pseudomonas aeruginosa PAO1] | 99.86 | 100.00 | NaN | 96768 | 98252 | + | VFDB | 015 | paeruginosa | - | 19 | Pseudomonas aeruginosa |
| flgA | NP\_252040 | (flgA) flagellar basal body P-ring biosynthesis protein FlgA [Deoxyhexose linking sugar 209 Da capping structure (AI138)] [Pseudomonas aeruginosa PAO1] | 99.86 | 100.00 | NaN | 63816 | 64514 | - | VFDB | 015 | paeruginosa | - | 12 | Pseudomonas aeruginosa |
| fptA | NP\_252911 | (fptA) Fe(III)-pyochelin receptor precursor [Pyochelin (VF0095)] [Pseudomonas aeruginosa PAO1] | 99.58 | 100.00 | NaN | 61864 | 64026 | + | VFDB | 015 | paeruginosa | - | 23 | Pseudomonas aeruginosa |
| phzS | NP\_252907 | (phzS) flavin dependent hydroxylase PhzS [Pyocyanin (VF0100)] [Pseudomonas aeruginosa PAO1] | 98.43 | 100.00 | NaN | 67156 | 68364 | - | VFDB | 015 | paeruginosa | - | 23 | Pseudomonas aeruginosa |
| phzG1 | NP\_252906 | (phzG1) phenazine biosynthesis protein PhzG pyridoxamine 5'-phosphate oxidase [Phenazines biosynthesis (CVF536)] [Pseudomonas aeruginosa PAO1] | 99.84 | 99.84 | NaN | 68602 | 69245 | - | VFDB | 015 | paeruginosa | - | 23 | Pseudomonas aeruginosa |
| ptxR | NP\_250948 | (ptxR) transcriptional regulator PtxR [pyoverdine (IA001)] [Pseudomonas aeruginosa PAO1] | 100.00 | 100.00 | NaN | 34159 | 35097 | + | VFDB | 015 | paeruginosa | - | 24 | Pseudomonas aeruginosa |
| pvcD | NP\_250947 | (pvcD) paerucumarin biosynthesis protein PvcD [pyoverdine (IA001)] [Pseudomonas aeruginosa PAO1] | 99.69 | 100.00 | NaN | 35330 | 35977 | - | VFDB | 015 | paeruginosa | - | 24 | Pseudomonas aeruginosa |
| pvcC | NP\_250946 | (pvcC) paerucumarin biosynthesis protein PvcC [pyoverdine (IA001)] [Pseudomonas aeruginosa PAO1] | 99.40 | 100.00 | NaN | 35970 | 37472 | - | VFDB | 015 | paeruginosa | - | 24 | Pseudomonas aeruginosa |
| pchH | NP\_252913 | (pchH) ABC transporter ATP-binding protein [Pyochelin (VF0095)] [Pseudomonas aeruginosa PAO1] | 99.36 | 100.00 | NaN | 58339 | 60051 | + | VFDB | 015 | paeruginosa | - | 23 | Pseudomonas aeruginosa |
| pvcB | NP\_250945 | (pvcB) paerucumarin biosynthesis protein PvcB [pyoverdine (IA001)] [Pseudomonas aeruginosa PAO1] | 99.66 | 100.00 | NaN | 37524 | 38399 | - | VFDB | 015 | paeruginosa | - | 24 | Pseudomonas aeruginosa |
| mucE | NP\_252722 | (mucE) small envelope protein MucE [Alginate regulation (CVF523)] [Pseudomonas aeruginosa PAO1] | 99.63 | 100.00 | NaN | 10416 | 10685 | + | VFDB | 015 | paeruginosa | - | 25 | Pseudomonas aeruginosa |
| tagT | NP\_248763 | (tagT) type six secretion associated protein TagT ATP-binding component of ABC transporter [HSI-I (VF0334)] [Pseudomonas aeruginosa PAO1] | 99.31 | 100.00 | NaN | 2141 | 2860 | + | VFDB | 015 | paeruginosa | - | 29 | Pseudomonas aeruginosa |
| tagS | NP\_248762 | (tagS) type IV secretion associated protein TagS forming a stable inner membrane complex with TagT [HSI-I (VF0334)] [Pseudomonas aeruginosa PAO1] | 99.75 | 100.00 | NaN | 2860 | 4059 | + | VFDB | 015 | paeruginosa | - | 29 | Pseudomonas aeruginosa |
| tagR | NP\_248761 | (tagR) type IV secretion associated protein TagR positively regulates PpkA [HSI-I (VF0334)] [Pseudomonas aeruginosa PAO1] | 99.83 | 100.00 | NaN | 4052 | 5764 | + | VFDB | 015 | paeruginosa | - | 29 | Pseudomonas aeruginosa |
| tagQ | NP\_248760 | (tagQ) type VI secretiona ssociated protein TagQ outer membrane lipoprotein [HSI-1 (Hcp-secretion island 1) (SS178)] [Pseudomonas aeruginosa PAO1] | 100.00 | 100.00 | NaN | 5826 | 6740 | + | VFDB | 015 | paeruginosa | - | 29 | Pseudomonas aeruginosa |
| phzH | NP\_248741 | (phzH) phenazine-modifying enzyme [Phenazines biosynthesis (CVF536)] [Pseudomonas aeruginosa PAO1] | 100.00 | 100.00 | NaN | 20291 | 22123 | - | VFDB | 015 | paeruginosa | - | 29 | Pseudomonas aeruginosa |
| exoT | NP\_248734 | (exoT) type III secretion system effector ExoT ADP ribosyltransferase activity and GTPase-activating protein activity [ExoT (VF0097)] [Pseudomonas aeruginosa PAO1] | 98.91 | 100.00 | NaN | 28266 | 29639 | - | VFDB | 015 | paeruginosa | - | 29 | Pseudomonas aeruginosa |
| pvcA | NP\_250944 | (pvcA) paerucumarin biosynthesis protein PvcA [pyoverdine (IA001)] [Pseudomonas aeruginosa PAO1] | 99.39 | 100.00 | NaN | 38417 | 39403 | - | VFDB | 015 | paeruginosa | - | 24 | Pseudomonas aeruginosa |
| pchG | NP\_252914 | (pchG) pyochelin biosynthetic protein PchG [Pyochelin (VF0095)] [Pseudomonas aeruginosa PAO1] | 99.43 | 100.00 | NaN | 57293 | 58342 | + | VFDB | 015 | paeruginosa | - | 23 | Pseudomonas aeruginosa |
| pchF | NP\_252915 | (pchF) pyochelin synthetase PchF [Pyochelin (VF0095)] [Pseudomonas aeruginosa PAO1] | 99.45 | 100.00 | NaN | 51867 | 57296 | + | VFDB | 015 | paeruginosa | - | 23 | Pseudomonas aeruginosa |
| pchE | NP\_252916 | (pchE) dihydroaeruginoic acid synthetase PchE [Pyochelin (VF0095)] [Pseudomonas aeruginosa PAO1] | 99.58 | 100.00 | NaN | 47554 | 51870 | + | VFDB | 015 | paeruginosa | - | 23 | Pseudomonas aeruginosa |
| alg44 | NP\_252232 | (alg44) alginate biosynthesis protein Alg8 [Alginate (VF0091)] [Pseudomonas aeruginosa PAO1] | 99.57 | 100.00 | NaN | 98335 | 99504 | + | VFDB | 015 | paeruginosa | - | 19 | Pseudomonas aeruginosa |
| algK | NP\_252233 | (algK) alginate biosynthesis protein Alg44 [Alginate (VF0091)] [Pseudomonas aeruginosa PAO1] | 99.86 | 100.00 | NaN | 99518 | 100945 | + | VFDB | 015 | paeruginosa | - | 19 | Pseudomonas aeruginosa |
| algE | NP\_252234 | (algE) alginate biosynthetic protein AlgK precursor [Alginate (VF0091)] [Pseudomonas aeruginosa PAO1] | 99.32 | 100.00 | NaN | 100942 | 102414 | + | VFDB | 015 | paeruginosa | - | 19 | Pseudomonas aeruginosa |
| algG | NP\_252235 | (algG) outer membrane protein AlgE [Alginate (VF0091)] [Pseudomonas aeruginosa PAO1] | 99.69 | 100.00 | NaN | 102435 | 104066 | + | VFDB | 015 | paeruginosa | - | 19 | Pseudomonas aeruginosa |
| algX | NP\_252236 | (algX) alginate biosynthesis protein AlgX [Alginate (VF0091)] [Pseudomonas aeruginosa PAO1] | 99.51 | 100.00 | NaN | 104079 | 105503 | + | VFDB | 015 | paeruginosa | - | 19 | Pseudomonas aeruginosa |
| algL | NP\_252237 | (algL) poly(beta-d-mannuronate) lyase precursor AlgL [Alginate (VF0091)] [Pseudomonas aeruginosa PAO1] | 99.82 | 100.00 | NaN | 105507 | 106610 | + | VFDB | 015 | paeruginosa | - | 19 | Pseudomonas aeruginosa |
| algI | NP\_252238 | (algI) alginate o-acetyltransferase AlgI [Alginate (VF0091)] [Pseudomonas aeruginosa PAO1] | 99.68 | 100.00 | NaN | 106852 | 108414 | + | VFDB | 015 | paeruginosa | - | 19 | Pseudomonas aeruginosa |
| algJ | NP\_252239 | (algJ) alginate o-acetyltransferase AlgJ [Alginate (VF0091)] [Pseudomonas aeruginosa PAO1] | 99.92 | 100.00 | NaN | 108429 | 109604 | + | VFDB | 015 | paeruginosa | - | 19 | Pseudomonas aeruginosa |
| algF | NP\_252240 | (algF) alginate o-acetyltransferase AlgF [Alginate (VF0091)] [Pseudomonas aeruginosa PAO1] | 99.39 | 100.00 | NaN | 109677 | 110327 | + | VFDB | 015 | paeruginosa | - | 19 | Pseudomonas aeruginosa |
| algA | NP\_252241 | (algA) phosphomannose isomerase / guanosine 5'-diphospho-D-mannose pyrophosphorylase [Alginate (VF0091)] [Pseudomonas aeruginosa PAO1] | 99.86 | 100.00 | NaN | 110524 | 111969 | + | VFDB | 015 | paeruginosa | - | 19 | Pseudomonas aeruginosa |
| pilB | NP\_253216 | (pilB) type 4 fimbrial biogenesis protein PilB [Type IV pili (VF0082)] [Pseudomonas aeruginosa PAO1] | 87.77 | 100.00 | NaN | 53550 | 55250 | + | VFDB | 015 | paeruginosa | - | 22 | Pseudomonas aeruginosa |
| xcpA/pilD | NP\_253218 | (xcpA/pilD) type 4 prepilin peptidase PilD [Type IV pili (VF0082)] [Pseudomonas aeruginosa PAO1] | 99.31 | 100.00 | NaN | 56472 | 57344 | + | VFDB | 015 | paeruginosa | - | 22 | Pseudomonas aeruginosa |
| pchA | NP\_252921 | (pchA) salicylate biosynthesis isochorismate synthase PchA [Pyochelin (VF0095)] [Pseudomonas aeruginosa PAO1] | 99.44 | 100.00 | NaN | 42113 | 43543 | - | VFDB | 015 | paeruginosa | - | 23 | Pseudomonas aeruginosa |
| pchB | NP\_252920 | (pchB) salicylate biosynthesis protein PchB [Pyochelin (VF0095)] [Pseudomonas aeruginosa PAO1] | 99.67 | 100.00 | NaN | 43540 | 43845 | - | VFDB | 015 | paeruginosa | - | 23 | Pseudomonas aeruginosa |
| pchC | NP\_252919 | (pchC) pyochelin biosynthetic protein PchC [Pyochelin (VF0095)] [Pseudomonas aeruginosa PAO1] | 99.60 | 100.00 | NaN | 43845 | 44600 | - | VFDB | 015 | paeruginosa | - | 23 | Pseudomonas aeruginosa |
| pchD | NP\_252918 | (pchD) pyochelin biosynthesis protein PchD [Pyochelin (VF0095)] [Pseudomonas aeruginosa PAO1] | 99.51 | 100.00 | NaN | 44597 | 46240 | - | VFDB | 015 | paeruginosa | - | 23 | Pseudomonas aeruginosa |
| pchR | NP\_252917 | (pchR) transcriptional regulator PchR [Pyochelin (VF0095)] [Pseudomonas aeruginosa PAO1] | 99.89 | 100.00 | NaN | 46470 | 47360 | + | VFDB | 015 | paeruginosa | - | 23 | Pseudomonas aeruginosa |
| algD | NP\_252230 | (algD) GDP-mannose 6-dehydrogenase AlgD [Alginate (VF0091)] [Pseudomonas aeruginosa PAO1] | 100.00 | 100.00 | NaN | 95318 | 96628 | + | VFDB | 015 | paeruginosa | - | 19 | Pseudomonas aeruginosa |
| flgM | NP\_252041 | (flgM) negative regulator of flagellin synthesis [Deoxyhexose linking sugar 209 Da capping structure (AI138)] [Pseudomonas aeruginosa PAO1] | 98.46 | 100.00 | NaN | 63348 | 63671 | - | VFDB | 015 | paeruginosa | - | 12 | Pseudomonas aeruginosa |
| pcrG | NP\_250396 | (pcrG) type III secretion system cytoplasmic regulator PcrG [TTSS (VF0083)] [Pseudomonas aeruginosa PAO1] | 100.00 | 100.00 | NaN | 221048 | 221344 | - | VFDB | 015 | paeruginosa | - | 3 | Pseudomonas aeruginosa |
| toxA | NP\_249839 | (toxA) exotoxin A precursor [ExoA (VF0086)] [Pseudomonas aeruginosa PAO1] | 99.64 | 100.00 | NaN | 253458 | 255374 | - | VFDB | 015 | paeruginosa | - | 11 | Pseudomonas aeruginosa |
| pscU | NP\_250381 | (pscU) type III secretion system protein PscU [TTSS (VF0083)] [Pseudomonas aeruginosa PAO1] | 99.52 | 100.00 | NaN | 231839 | 232888 | + | VFDB | 015 | paeruginosa | - | 3 | Pseudomonas aeruginosa |
| chpE | NP\_249108 | (chpE) probable chemotaxis protein [Type IV pili (VF0082)] [Pseudomonas aeruginosa PAO1] | 99.02 | 100.00 | NaN | 248313 | 248924 | - | VFDB | 015 | paeruginosa | - | 4 | Pseudomonas aeruginosa |
| chpD | NP\_249107 | (chpD) probable transcriptional regulator [Type IV pili (VF0082)] [Pseudomonas aeruginosa PAO1] | 99.25 | 100.00 | NaN | 249001 | 249795 | - | VFDB | 015 | paeruginosa | - | 4 | Pseudomonas aeruginosa |
| chpC | NP\_249106 | (chpC) probable chemotaxis protein [Type IV pili (VF0082)] [Pseudomonas aeruginosa PAO1] | 98.82 | 100.00 | NaN | 249803 | 250309 | - | VFDB | 015 | paeruginosa | - | 4 | Pseudomonas aeruginosa |
| chpB | NP\_249105 | (chpB) probable methylesterase [Type IV pili (VF0082)] [Pseudomonas aeruginosa PAO1] | 99.13 | 100.00 | NaN | 250306 | 251337 | - | VFDB | 015 | paeruginosa | - | 4 | Pseudomonas aeruginosa |
| chpA | NP\_249104 | (chpA) still frameshift probable component of chemotactic signal transduction system [Type IV pili (VF0082)] [Pseudomonas aeruginosa PAO1] | 99.66 | 100.00 | NaN | 251330 | 258748 | - | VFDB | 015 | paeruginosa | - | 4 | Pseudomonas aeruginosa |
| pilK | NP\_249103 | (pilK) methyltransferase PilK [Type IV pili (VF0082)] [Pseudomonas aeruginosa PAO1] | 99.77 | 100.00 | NaN | 258760 | 259635 | - | VFDB | 015 | paeruginosa | - | 4 | Pseudomonas aeruginosa |
| pilJ | NP\_249102 | (pilJ) twitching motility protein PilJ [Type IV pili (VF0082)] [Pseudomonas aeruginosa PAO1] | 99.85 | 100.00 | NaN | 259696 | 261744 | - | VFDB | 015 | paeruginosa | - | 4 | Pseudomonas aeruginosa |
| lasA | NP\_250562 | (lasA) LasA protease precursor [LasA (VF0088)] [Pseudomonas aeruginosa PAO1] | 99.12 | 100.00 | NaN | 39233 | 40489 | - | VFDB | 015 | paeruginosa | - | 3 | Pseudomonas aeruginosa |
| phzA1 | NP\_252899 | (phzA1) phenazine biosynthesis protein PhzA [Phenazines biosynthesis (CVF536)] [Pseudomonas aeruginosa PAO1] | 95.94 | 90.59 | NaN | 1273 | 1715 | - | VFDB | 015 | paeruginosa | - | 3 | Pseudomonas aeruginosa |
| phzB1 | NP\_252900 | (phzB1) phenazine biosynthesis protein PhzB [Phenazines biosynthesis (CVF536)] [Pseudomonas aeruginosa PAO1] | 91.95 | 88.96 | NaN | 749 | 1183 | - | VFDB | 015 | paeruginosa | - | 3 | Pseudomonas aeruginosa |
| fimV | NP\_251805 | (fimV) putative Type IV pili related protein [Type IV pili (VF0082)] [Pseudomonas aeruginosa PAO1] | 99.35 | 100.00 | NaN | 404527 | 407286 | - | VFDB | 015 | paeruginosa | - | 2 | Pseudomonas aeruginosa |
| xcpQ | NP\_251795 | (xcpQ) general secretion pathway protein D [xcp secretion system (VF0084)] [Pseudomonas aeruginosa PAO1] | 99.90 | 100.00 | NaN | 393435 | 395411 | + | VFDB | 015 | paeruginosa | - | 2 | Pseudomonas aeruginosa |
| xcpP | NP\_251794 | (xcpP) secretion protein XcpP [xcp secretion system (VF0084)] [Pseudomonas aeruginosa PAO1] | 99.86 | 100.00 | NaN | 392723 | 393430 | + | VFDB | 015 | paeruginosa | - | 2 | Pseudomonas aeruginosa |
| pscT | NP\_250382 | (pscT) type III secretion system protein PscT [TTSS (VF0083)] [Pseudomonas aeruginosa PAO1] | 99.37 | 100.00 | NaN | 231054 | 231842 | + | VFDB | 015 | paeruginosa | - | 3 | Pseudomonas aeruginosa |
| xcpR | NP\_251793 | (xcpR) general secretion pathway protein E [xcp secretion system (VF0084)] [Pseudomonas aeruginosa PAO1] | 100.00 | 100.00 | NaN | 390995 | 392503 | - | VFDB | 015 | paeruginosa | - | 2 | Pseudomonas aeruginosa |
| xcpT | NP\_251791 | (xcpT) general secretion pathway protein G [xcp secretion system (VF0084)] [Pseudomonas aeruginosa PAO1] | 99.78 | 100.00 | NaN | 389327 | 389773 | - | VFDB | 015 | paeruginosa | - | 2 | Pseudomonas aeruginosa |
| xcpU | NP\_251790 | (xcpU) general secretion pathway protein H [xcp secretion system (VF0084)] [Pseudomonas aeruginosa PAO1] | 99.81 | 100.00 | NaN | 388802 | 389320 | - | VFDB | 015 | paeruginosa | - | 2 | Pseudomonas aeruginosa |
| xcpV | NP\_251789 | (xcpV) general secretion pathway protein I [xcp secretion system (VF0084)] [Pseudomonas aeruginosa PAO1] | 99.23 | 100.00 | NaN | 388416 | 388805 | - | VFDB | 015 | paeruginosa | - | 2 | Pseudomonas aeruginosa |
| xcpW | NP\_251788 | (xcpW) general secretion pathway protein J [xcp secretion system (VF0084)] [Pseudomonas aeruginosa PAO1] | 99.44 | 100.00 | NaN | 387706 | 388419 | - | VFDB | 015 | paeruginosa | - | 2 | Pseudomonas aeruginosa |
| xcpX | NP\_251787 | (xcpX) general secretion pathway protein K [xcp secretion system (VF0084)] [Pseudomonas aeruginosa PAO1] | 99.70 | 100.00 | NaN | 386708 | 387709 | - | VFDB | 015 | paeruginosa | - | 2 | Pseudomonas aeruginosa |
| xcpY | NP\_251786 | (xcpY) general secretion pathway protein L [xcp secretion system (VF0084)] [Pseudomonas aeruginosa PAO1] | 99.65 | 100.00 | NaN | 385563 | 386711 | - | VFDB | 015 | paeruginosa | - | 2 | Pseudomonas aeruginosa |
| xcpZ | NP\_251785 | (xcpZ) general secretion pathway protein M [xcp secretion system (VF0084)] [Pseudomonas aeruginosa PAO1] | 99.43 | 100.00 | NaN | 385037 | 385561 | - | VFDB | 015 | paeruginosa | - | 2 | Pseudomonas aeruginosa |
| motB | NP\_253640 | (motB) flagellar motor protein [Deoxyhexose linking sugar 209 Da capping structure (AI138)] [Pseudomonas aeruginosa PAO1] | 99.33 | 100.00 | NaN | 500544 | 501587 | + | VFDB | 015 | paeruginosa | - | 1 | Pseudomonas aeruginosa |
| motA | NP\_253641 | (motA) flagellar motor protein [Deoxyhexose linking sugar 209 Da capping structure (AI138)] [Pseudomonas aeruginosa PAO1] | 98.00 | 100.00 | NaN | 499673 | 500524 | + | VFDB | 015 | paeruginosa | - | 1 | Pseudomonas aeruginosa |
| waaA | NP\_253675 | (waaA) lipopolysaccharide core biosynthesis protein WaaP [LPS (VF0085)] [Pseudomonas aeruginosa PAO1] | 99.92 | 100.00 | NaN | 454445 | 455722 | + | VFDB | 015 | paeruginosa | - | 1 | Pseudomonas aeruginosa |
| waaP | NP\_253696 | (waaP) UDP-glucose:(heptosyl) LPS alpha 13-glucosyltransferase WaaG [LPS (VF0085)] [Pseudomonas aeruginosa PAO1] | 100.00 | 100.00 | NaN | 428534 | 429340 | + | VFDB | 015 | paeruginosa | - | 1 | Pseudomonas aeruginosa |
| waaG | NP\_253697 | (waaG) B-band O-antigen polymerase [LPS (VF0085)] [Pseudomonas aeruginosa PAO1] | 99.47 | 100.00 | NaN | 427416 | 428537 | + | VFDB | 015 | paeruginosa | - | 1 | Pseudomonas aeruginosa |
| waaC | NP\_253698 | (waaC) 3-deoxy-D-manno-octulosonic-acid (KDO) transferase [LPS (VF0085)] [Pseudomonas aeruginosa PAO1] | 99.44 | 100.00 | NaN | 426352 | 427419 | + | VFDB | 015 | paeruginosa | - | 1 | Pseudomonas aeruginosa |
| waaF | NP\_253699 | (waaF) heptosyltransferase I [LPS (VF0085)] [Pseudomonas aeruginosa PAO1] | 99.71 | 100.00 | NaN | 425318 | 426355 | + | VFDB | 015 | paeruginosa | - | 1 | Pseudomonas aeruginosa |
| xcpS | NP\_251792 | (xcpS) general secretion pathway protein F [xcp secretion system (VF0084)] [Pseudomonas aeruginosa PAO1] | 99.92 | 100.00 | NaN | 389778 | 390995 | - | VFDB | 015 | paeruginosa | - | 2 | Pseudomonas aeruginosa |
| pilQ | NP\_253727 | (pilQ) type 4 fimbrial biogenesis protein PilQ [Type IV pili (VF0082)] [Pseudomonas aeruginosa PAO1] | 97.11 | 100.00 | NaN | 379350 | 381494 | + | VFDB | 015 | paeruginosa | - | 1 | Pseudomonas aeruginosa |
| pscS | NP\_250383 | (pscS) type III secretion system protein PscS [TTSS (VF0083)] [Pseudomonas aeruginosa PAO1] | 99.62 | 100.00 | NaN | 230791 | 231057 | + | VFDB | 015 | paeruginosa | - | 3 | Pseudomonas aeruginosa |
| pscQ | NP\_250385 | (pscQ) type III secretion system protein PscQ [TTSS (VF0083)] [Pseudomonas aeruginosa PAO1] | 99.14 | 100.00 | NaN | 229209 | 230138 | + | VFDB | 015 | paeruginosa | - | 3 | Pseudomonas aeruginosa |
| pcrV | NP\_250397 | (pcrV) type III secretion system hydrophilic translocator needle tip protein PcrV [TTSS (VF0083)] [Pseudomonas aeruginosa PAO1] | 98.87 | 100.00 | NaN | 220154 | 221038 | - | VFDB | 015 | paeruginosa | - | 3 | Pseudomonas aeruginosa |
| pcrH | NP\_250398 | (pcrH) type III secretion system regulatory protein PcrH [TTSS (VF0083)] [Pseudomonas aeruginosa PAO1] | 99.41 | 100.00 | NaN | 219639 | 220145 | - | VFDB | 015 | paeruginosa | - | 3 | Pseudomonas aeruginosa |
| popB | NP\_250399 | (popB) type III secretion system hydrophobic translocator pore protein PopB [TTSS (VF0083)] [Pseudomonas aeruginosa PAO1] | 99.66 | 100.00 | NaN | 218486 | 219658 | - | VFDB | 015 | paeruginosa | - | 3 | Pseudomonas aeruginosa |
| popD | NP\_250400 | (popD) type III secretion system hydrophobic translocator pore protein PopD [TTSS (VF0083)] [Pseudomonas aeruginosa PAO1] | 99.78 | 100.00 | NaN | 217587 | 218474 | - | VFDB | 015 | paeruginosa | - | 3 | Pseudomonas aeruginosa |
| exsC | NP\_250401 | (exsC) type III secretion system regulatory protein ExsC [TTSS (VF0083)] [Pseudomonas aeruginosa PAO1] | 99.09 | 100.00 | NaN | 217024 | 217461 | - | VFDB | 015 | paeruginosa | - | 3 | Pseudomonas aeruginosa |
| exsE | NP\_250402 | (exsE) type III secretion system regulatory protein ExsE [TTSS (VF0083)] [Pseudomonas aeruginosa PAO1] | 99.59 | 100.00 | NaN | 216770 | 217015 | - | VFDB | 015 | paeruginosa | - | 3 | Pseudomonas aeruginosa |
| exsB | NP\_250403 | (exsB) type III secretion system piolitin ExsB [TTSS (VF0083)] [Pseudomonas aeruginosa PAO1] | 99.76 | 100.00 | NaN | 216348 | 216761 | - | VFDB | 015 | paeruginosa | - | 3 | Pseudomonas aeruginosa |
| exsA | NP\_250404 | (exsA) type III secretion system regulatory protein ExsA [TTSS (VF0083)] [Pseudomonas aeruginosa PAO1] | 99.88 | 100.00 | NaN | 215214 | 216050 | - | VFDB | 015 | paeruginosa | - | 3 | Pseudomonas aeruginosa |
| exsD | NP\_250405 | (exsD) type III secretion system regulatory protein ExsD [TTSS (VF0083)] [Pseudomonas aeruginosa PAO1] | 99.88 | 100.00 | NaN | 214286 | 215116 | - | VFDB | 015 | paeruginosa | - | 3 | Pseudomonas aeruginosa |
| pscB | NP\_250406 | (pscB) type III secretion system protein PscB [TTSS (VF0083)] [Pseudomonas aeruginosa PAO1] | 99.29 | 100.00 | NaN | 213830 | 214252 | - | VFDB | 015 | paeruginosa | - | 3 | Pseudomonas aeruginosa |
| pscC | NP\_250407 | (pscC) type III secretion system secretin PscC [TTSS (VF0083)] [Pseudomonas aeruginosa PAO1] | 99.67 | 100.00 | NaN | 212028 | 213830 | - | VFDB | 015 | paeruginosa | - | 3 | Pseudomonas aeruginosa |
| pscD | NP\_250408 | (pscD) type III secretion system basal body protein PscD [TTSS (VF0083)] [Pseudomonas aeruginosa PAO1] | 99.69 | 100.00 | NaN | 210728 | 212026 | - | VFDB | 015 | paeruginosa | - | 3 | Pseudomonas aeruginosa |
| pscE | NP\_250409 | (pscE) type III secretion system cochaperone PscE for PscG [TTSS (VF0083)] [Pseudomonas aeruginosa PAO1] | 98.53 | 100.00 | NaN | 210562 | 210765 | - | VFDB | 015 | paeruginosa | - | 3 | Pseudomonas aeruginosa |
| pscF | NP\_250410 | (pscF) type III secretion system needle filament protein PscF [TTSS (VF0083)] [Pseudomonas aeruginosa PAO1] | 100.00 | 100.00 | NaN | 210302 | 210559 | - | VFDB | 015 | paeruginosa | - | 3 | Pseudomonas aeruginosa |
| pscR | NP\_250384 | (pscR) type III secretion system protein PscR [TTSS (VF0083)] [Pseudomonas aeruginosa PAO1] | 99.69 | 100.00 | NaN | 230135 | 230788 | + | VFDB | 015 | paeruginosa | - | 3 | Pseudomonas aeruginosa |
| pscG | NP\_250411 | (pscG) type III secretion system chaperone PscG for PscF [TTSS (VF0083)] [Pseudomonas aeruginosa PAO1] | 100.00 | 100.00 | NaN | 209952 | 210299 | - | VFDB | 015 | paeruginosa | - | 3 | Pseudomonas aeruginosa |
| pscI | NP\_250413 | (pscI) type III secretion system inner rod protein PscI [TTSS (VF0083)] [Pseudomonas aeruginosa PAO1] | 99.70 | 100.00 | NaN | 209186 | 209524 | - | VFDB | 015 | paeruginosa | - | 3 | Pseudomonas aeruginosa |
| pscJ | NP\_250414 | (pscJ) type III secretion system inner MS ring protein [TTSS (VF0083)] [Pseudomonas aeruginosa PAO1] | 99.87 | 100.00 | NaN | 208443 | 209189 | - | VFDB | 015 | paeruginosa | - | 3 | Pseudomonas aeruginosa |
| pscK | NP\_250415 | (pscK) type III secretion system protein PscK [TTSS (VF0083)] [Pseudomonas aeruginosa PAO1] | 98.72 | 99.04 | NaN | 207814 | 208434 | - | VFDB | 015 | paeruginosa | - | 3 | Pseudomonas aeruginosa |
| pscL | NP\_250416 | (pscL) type III secretion systemt protein PscL [TTSS (VF0083)] [Pseudomonas aeruginosa PAO1] | 98.92 | 100.00 | NaN | 207191 | 207835 | - | VFDB | 015 | paeruginosa | - | 3 | Pseudomonas aeruginosa |
| tse1 | NP\_250535 | (tse1) type VI secretion system effector Tse1 peptidoglycanhydrolase [HSI-1 (Hcp-secretion island 1) (SS178)] [Pseudomonas aeruginosa PAO1] | 99.78 | 100.00 | NaN | 68852 | 69316 | + | VFDB | 015 | paeruginosa | - | 3 | Pseudomonas aeruginosa |
| pcrD | NP\_250394 | (pcrD) type III secretion system protein PcrD [TTSS (VF0083)] [Pseudomonas aeruginosa PAO1] | 99.06 | 100.00 | NaN | 221803 | 223923 | - | VFDB | 015 | paeruginosa | - | 3 | Pseudomonas aeruginosa |
| pcr4 | NP\_250393 | (pcr4) type III secretion system protein Pcr4 [TTSS (VF0083)] [Pseudomonas aeruginosa PAO1] | 100.00 | 100.00 | NaN | 223920 | 224249 | - | VFDB | 015 | paeruginosa | - | 3 | Pseudomonas aeruginosa |
| pcr3 | NP\_250392 | (pcr3) type III secretion system protein Pcr3 [TTSS (VF0083)] [Pseudomonas aeruginosa PAO1] | 100.00 | 100.00 | NaN | 224254 | 224619 | - | VFDB | 015 | paeruginosa | - | 3 | Pseudomonas aeruginosa |
| pcr2 | NP\_250391 | (pcr2) type III secretion system protein Pcr2 [TTSS (VF0083)] [Pseudomonas aeruginosa PAO1] | 98.92 | 100.00 | NaN | 224616 | 224987 | - | VFDB | 015 | paeruginosa | - | 3 | Pseudomonas aeruginosa |
| pcr1 | NP\_250390 | (pcr1) type III secretion system protein Pcr1 [TTSS (VF0083)] [Pseudomonas aeruginosa PAO1] | 100.00 | 100.00 | NaN | 224974 | 225252 | - | VFDB | 015 | paeruginosa | - | 3 | Pseudomonas aeruginosa |
| popN | NP\_250389 | (popN) type III secretion system outer membrane protein PopN [TTSS (VF0083)] [Pseudomonas aeruginosa PAO1] | 99.08 | 100.00 | NaN | 225233 | 226099 | - | VFDB | 015 | paeruginosa | - | 3 | Pseudomonas aeruginosa |
| pscN | NP\_250388 | (pscN) type III secretion system ATPase PscN [TTSS (VF0083)] [Pseudomonas aeruginosa PAO1] | 99.09 | 100.00 | NaN | 226290 | 227612 | + | VFDB | 015 | paeruginosa | - | 3 | Pseudomonas aeruginosa |
| pscO | NP\_250387 | (pscO) type III secretion system protein PscO [TTSS (VF0083)] [Pseudomonas aeruginosa PAO1] | 99.79 | 100.00 | NaN | 227609 | 228085 | + | VFDB | 015 | paeruginosa | - | 3 | Pseudomonas aeruginosa |
| pscP | NP\_250386 | (pscP) type III secretion system protein PscP [TTSS (VF0083)] [Pseudomonas aeruginosa PAO1] | 96.49 | 100.00 | NaN | 228073 | 229212 | + | VFDB | 015 | paeruginosa | - | 3 | Pseudomonas aeruginosa |
| pscH | NP\_250412 | (pscH) type III secretion system protein PscH [TTSS (VF0083)] [Pseudomonas aeruginosa PAO1] | 100.00 | 100.00 | NaN | 209524 | 209955 | - | VFDB | 015 | paeruginosa | - | 3 | Pseudomonas aeruginosa |
| flgN | NP\_252042 | (flgN) flagella synthesis protein FlgN [Deoxyhexose linking sugar 209 Da capping structure (AI138)] [Pseudomonas aeruginosa PAO1] | 98.94 | 100.00 | NaN | 62823 | 63293 | - | VFDB | 015 | paeruginosa | - | 12 | Pseudomonas aeruginosa |
| pilP | NP\_253728 | (pilP) type IV pilus biogenesis protein PilP [Type IV pili (VF0082)] [Pseudomonas aeruginosa PAO1] | 99.81 | 100.00 | NaN | 378772 | 379296 | + | VFDB | 015 | paeruginosa | - | 1 | Pseudomonas aeruginosa |
| pilN | NP\_253730 | (pilN) type IV pilus inner membrane platform protein PilN [Type IV pili (VF0082)] [Pseudomonas aeruginosa PAO1] | 100.00 | 100.00 | NaN | 377559 | 378155 | + | VFDB | 015 | paeruginosa | - | 1 | Pseudomonas aeruginosa |
| fliR | NP\_250139 | (fliR) flagellar biosynthetic protein FliR [Flagella (VF0273)] [Pseudomonas aeruginosa PAO1] | 100.00 | 100.00 | NaN | 209722 | 210498 | + | VFDB | 015 | paeruginosa | - | 8 | Pseudomonas aeruginosa |
| flhB | NP\_250140 | (flhB) flagellar biosynthetic protein FlhB [Flagella (VF0273)] [Pseudomonas aeruginosa PAO1] | 99.74 | 100.00 | NaN | 210501 | 211637 | + | VFDB | 015 | paeruginosa | - | 8 | Pseudomonas aeruginosa |
| flhA | NP\_250143 | (flhA) flagellar biosynthesis protein FlhA [Flagella (VF0273)] [Pseudomonas aeruginosa PAO1] | 99.77 | 100.00 | NaN | 214484 | 216607 | + | VFDB | 015 | paeruginosa | - | 8 | Pseudomonas aeruginosa |
| flhF | NP\_250144 | (flhF) flagellar biosynthesis protein FlhF [Flagella (VF0273)] [Pseudomonas aeruginosa PAO1] | 99.61 | 100.00 | NaN | 216691 | 217980 | + | VFDB | 015 | paeruginosa | - | 8 | Pseudomonas aeruginosa |
| fleN | NP\_250145 | (fleN) flagellar synthesis regulator FleN [Flagella (VF0273)] [Pseudomonas aeruginosa PAO1] | 99.88 | 100.00 | NaN | 218119 | 218961 | + | VFDB | 015 | paeruginosa | - | 8 | Pseudomonas aeruginosa |
| fliA | NP\_250146 | (fliA) flagellar biosynthesis sigma factor FliA [Deoxyhexose linking sugar 209 Da capping structure (AI138)] [Pseudomonas aeruginosa PAO1] | 99.87 | 100.00 | NaN | 218958 | 219701 | + | VFDB | 015 | paeruginosa | - | 8 | Pseudomonas aeruginosa |
| motC | NP\_250151 | (motC) flagellar motor protein [Deoxyhexose linking sugar 209 Da capping structure (AI138)] [Pseudomonas aeruginosa PAO1] | 99.86 | 100.00 | NaN | 224696 | 225436 | + | VFDB | 015 | paeruginosa | - | 8 | Pseudomonas aeruginosa |
| motD | NP\_250152 | (motD) flagellar motor protein [Deoxyhexose linking sugar 209 Da capping structure (AI138)] [Pseudomonas aeruginosa PAO1] | 99.66 | 100.00 | NaN | 225449 | 226339 | + | VFDB | 015 | paeruginosa | - | 8 | Pseudomonas aeruginosa |
| algB | NP\_254170 | (algB) two-component response regulator AlgB [Alginate (VF0091)] [Pseudomonas aeruginosa PAO1] | 100.00 | 100.00 | NaN | 143131 | 144480 | - | VFDB | 015 | paeruginosa | - | 9 | Pseudomonas aeruginosa |
| exoY | NP\_250881 | (exoY) type III secretion system effector ExoY adenylate cyclase [ExoY (VF0099)] [Pseudomonas aeruginosa PAO1] | 99.56 | 100.00 | NaN | 29811 | 30947 | + | VFDB | 015 | paeruginosa | - | 10 | Pseudomonas aeruginosa |
| flgB | NP\_249768 | (flgB) flagellar basal body rod protein FlgB [Deoxyhexose linking sugar 209 Da capping structure (AI138)] [Pseudomonas aeruginosa PAO1] | 100.00 | 100.00 | NaN | 168213 | 168620 | + | VFDB | 015 | paeruginosa | - | 11 | Pseudomonas aeruginosa |
| flgC | NP\_249769 | (flgC) flagellar basal-body rod protein FlgC [Flagella (VF0273)] [Pseudomonas aeruginosa PAO1] | 100.00 | 100.00 | NaN | 168626 | 169066 | + | VFDB | 015 | paeruginosa | - | 11 | Pseudomonas aeruginosa |
| flgD | NP\_249770 | (flgD) flagellar basal-body rod modification protein FlgD [Flagella (VF0273)] [Pseudomonas aeruginosa PAO1] | 99.86 | 100.00 | NaN | 169079 | 169792 | + | VFDB | 015 | paeruginosa | - | 11 | Pseudomonas aeruginosa |
| flgE | NP\_249771 | (flgE) flagellar hook protein FlgE [Flagella (VF0273)] [Pseudomonas aeruginosa PAO1] | 99.71 | 100.00 | NaN | 169820 | 171208 | + | VFDB | 015 | paeruginosa | - | 11 | Pseudomonas aeruginosa |
| fliQ | NP\_250138 | (fliQ) flagellar biosynthetic protein FliQ [Flagella (VF0273)] [Pseudomonas aeruginosa PAO1] | 99.63 | 100.00 | NaN | 209453 | 209722 | + | VFDB | 015 | paeruginosa | - | 8 | Pseudomonas aeruginosa |
| flgF | NP\_249772 | (flgF) flagellar basal-body rod protein FlgF [Flagella (VF0273)] [Pseudomonas aeruginosa PAO1] | 99.87 | 100.00 | NaN | 171426 | 172175 | + | VFDB | 015 | paeruginosa | - | 11 | Pseudomonas aeruginosa |
| flgH | NP\_249774 | (flgH) flagellar L-ring protein precursor FlgH [Flagella (VF0273)] [Pseudomonas aeruginosa PAO1] | 99.57 | 100.00 | NaN | 173053 | 173748 | + | VFDB | 015 | paeruginosa | - | 11 | Pseudomonas aeruginosa |
| flgI | NP\_249775 | (flgI) flagellar P-ring protein precursor FlgI [Flagella (VF0273)] [Pseudomonas aeruginosa PAO1] | 99.73 | 100.00 | NaN | 173760 | 174869 | + | VFDB | 015 | paeruginosa | - | 11 | Pseudomonas aeruginosa |
| flgJ | NP\_249776 | (flgJ) flagellar rod assembly protein/muramidase FlgJ [Flagella (VF0273)] [Pseudomonas aeruginosa PAO1] | 99.83 | 100.00 | NaN | 174880 | 176082 | + | VFDB | 015 | paeruginosa | - | 11 | Pseudomonas aeruginosa |
| flgK | NP\_249777 | (flgK) flagellar hook-associated protein 1 FlgK [Flagella (VF0273)] [Pseudomonas aeruginosa PAO1] | 86.76 | 99.71 | NaN | 176101 | 178149 | + | VFDB | 015 | paeruginosa | - | 11 | Pseudomonas aeruginosa |
| fleQ | NP\_249788 | (fleQ) transcriptional regulator FleQ [Flagella (VF0273)] [Pseudomonas aeruginosa PAO1] | 99.19 | 100.00 | NaN | 200459 | 201931 | + | VFDB | 015 | paeruginosa | - | 11 | Pseudomonas aeruginosa |
| fleS | NP\_249789 | (fleS) two-component sensor [Deoxyhexose linking sugar 209 Da capping structure (AI138)] [Pseudomonas aeruginosa PAO1] | 99.50 | 100.00 | NaN | 202044 | 203252 | + | VFDB | 015 | paeruginosa | - | 11 | Pseudomonas aeruginosa |
| fleR | NP\_249790 | (fleR) two-component response regulator [Flagella (VF0273)] [Pseudomonas aeruginosa PAO1] | 99.44 | 100.00 | NaN | 203257 | 204678 | + | VFDB | 015 | paeruginosa | - | 11 | Pseudomonas aeruginosa |
| fliE | NP\_249791 | (fliE) flagellar hook-basal body complex protein FliE [Flagella (VF0273)] [Pseudomonas aeruginosa PAO1] | 99.39 | 100.00 | NaN | 204925 | 205254 | + | VFDB | 015 | paeruginosa | - | 11 | Pseudomonas aeruginosa |
| fliF | NP\_249792 | (fliF) flagellar M-ring protein FliF [Flagella (VF0273)] [Pseudomonas aeruginosa PAO1] | 99.83 | 100.00 | NaN | 205277 | 207073 | + | VFDB | 015 | paeruginosa | - | 11 | Pseudomonas aeruginosa |
| fliG | NP\_249793 | (fliG) flagellar motor switch protein G [Flagella (VF0273)] [Pseudomonas aeruginosa PAO1] | 99.70 | 100.00 | NaN | 207079 | 208095 | + | VFDB | 015 | paeruginosa | - | 11 | Pseudomonas aeruginosa |
| pcrR | NP\_250395 | (pcrR) type III secretion system regulatory protein PcrR [TTSS (VF0083)] [Pseudomonas aeruginosa PAO1] | 99.08 | 100.00 | NaN | 221372 | 221806 | - | VFDB | 015 | paeruginosa | - | 3 | Pseudomonas aeruginosa |
| fliI | NP\_249795 | (fliI) flagellum-specific ATP synthase FliI [Flagella (VF0273)] [Pseudomonas aeruginosa PAO1] | 99.63 | 100.00 | NaN | 208893 | 210248 | + | VFDB | 015 | paeruginosa | - | 11 | Pseudomonas aeruginosa |
| fliJ | NP\_249796 | (fliJ) flagellar protein FliJ [Flagella (VF0273)] [Pseudomonas aeruginosa PAO1] | 99.78 | 100.00 | NaN | 210262 | 210705 | + | VFDB | 015 | paeruginosa | - | 11 | Pseudomonas aeruginosa |
| rhlC | NP\_249821 | (rhlC) rhamnosyltransferase 2 [Rhamnolipid biosynthesis (CVF524)] [Pseudomonas aeruginosa PAO1] | 99.49 | 100.00 | NaN | 234970 | 235947 | - | VFDB | 015 | paeruginosa | - | 11 | Pseudomonas aeruginosa |
| flgG | NP\_249773 | (flgG) flagellar basal-body rod protein FlgG [Flagella (VF0273)] [Pseudomonas aeruginosa PAO1] | 99.75 | 100.00 | NaN | 172222 | 173007 | + | VFDB | 015 | paeruginosa | - | 11 | Pseudomonas aeruginosa |
| pilO | NP\_253729 | (pilO) type IV pilus inner membrane platform protein PilO [Type IV pili (VF0082)] [Pseudomonas aeruginosa PAO1] | 99.84 | 100.00 | NaN | 378152 | 378775 | + | VFDB | 015 | paeruginosa | - | 1 | Pseudomonas aeruginosa |
| fliP | NP\_250137 | (fliP) flagellar biosynthetic protein FliP [Flagella (VF0273)] [Pseudomonas aeruginosa PAO1] | 99.61 | 100.00 | NaN | 208641 | 209408 | + | VFDB | 015 | paeruginosa | - | 8 | Pseudomonas aeruginosa |
| fliN | NP\_250135 | (fliN) flagellar motor switch protein FliN [Flagella (VF0273)] [Pseudomonas aeruginosa PAO1] | 100.00 | 100.00 | NaN | 207717 | 208190 | + | VFDB | 015 | paeruginosa | - | 8 | Pseudomonas aeruginosa |
| pilM | NP\_253731 | (pilM) type IV pilus inner membrane platform protein PilM [Type IV pili (VF0082)] [Pseudomonas aeruginosa PAO1] | 99.81 | 100.00 | NaN | 376495 | 377559 | + | VFDB | 015 | paeruginosa | - | 1 | Pseudomonas aeruginosa |
| fliR | NP\_250139 | (fliR) flagellar biosynthetic protein FliR [Flagella (VF0273)] [Pseudomonas aeruginosa PAO1] | 98.33 | 100.00 | NaN | 254777 | 255553 | + | VFDB | 015 | paeruginosa | - | 1 | Pseudomonas aeruginosa |
| fliQ | NP\_250138 | (fliQ) flagellar biosynthetic protein FliQ [Flagella (VF0273)] [Pseudomonas aeruginosa PAO1] | 98.78 | 91.11 | NaN | 254529 | 254774 | + | VFDB | 015 | paeruginosa | - | 1 | Pseudomonas aeruginosa |
| fliP | NP\_250137 | (fliP) flagellar biosynthetic protein FliP [Flagella (VF0273)] [Pseudomonas aeruginosa PAO1] | 98.78 | 95.83 | NaN | 253749 | 254484 | + | VFDB | 015 | paeruginosa | - | 1 | Pseudomonas aeruginosa |
| algP/algR3 | NP\_253940 | (algP/algR3) alginate regulatory protein AlgP [Alginate (VF0091)] [Pseudomonas aeruginosa PAO1] | 99.53 | 100.00 | NaN | 123575 | 124633 | + | VFDB | 015 | paeruginosa | - | 1 | Pseudomonas aeruginosa |
| algQ | NP\_253942 | (algQ) Alginate regulatory protein AlgQ [Alginate (VF0091)] [Pseudomonas aeruginosa PAO1] | 99.59 | 100.00 | NaN | 122276 | 122758 | + | VFDB | 015 | paeruginosa | - | 1 | Pseudomonas aeruginosa |
| algR | NP\_253948 | (algR) alginate biosynthesis regulatory protein AlgR [Alginate (VF0091)] [Pseudomonas aeruginosa PAO1] | 100.00 | 100.00 | NaN | 116385 | 117131 | + | VFDB | 015 | paeruginosa | - | 1 | Pseudomonas aeruginosa |
| algZ | NP\_253949 | (algZ) sigma factor AlgU [Alginate (VF0091)] [Pseudomonas aeruginosa PAO1] | 99.91 | 100.00 | NaN | 115304 | 116380 | + | VFDB | 015 | paeruginosa | - | 1 | Pseudomonas aeruginosa |
| algC | NP\_254009 | (algC) phosphomannomutase AlgC [Alginate biosynthesis (CVF522)] [Pseudomonas aeruginosa PAO1] | 99.86 | 100.00 | NaN | 45880 | 47271 | - | VFDB | 015 | paeruginosa | - | 1 | Pseudomonas aeruginosa |
| pilI | NP\_249101 | (pilI) twitching motility protein PilI [Type IV pili (VF0082)] [Pseudomonas aeruginosa PAO1] | 99.81 | 100.00 | NaN | 261829 | 262365 | - | VFDB | 015 | paeruginosa | - | 4 | Pseudomonas aeruginosa |
| pilH | NP\_249100 | (pilH) twitching motility protein PilH [Type IV pili (VF0082)] [Pseudomonas aeruginosa PAO1] | 100.00 | 100.00 | NaN | 262416 | 262781 | - | VFDB | 015 | paeruginosa | - | 4 | Pseudomonas aeruginosa |
| pilG | NP\_249099 | (pilG) twitching motility protein PilG [Type IV pili (VF0082)] [Pseudomonas aeruginosa PAO1] | 100.00 | 100.00 | NaN | 262828 | 263235 | - | VFDB | 015 | paeruginosa | - | 4 | Pseudomonas aeruginosa |
| pilU | NP\_249087 | (pilU) twitching motility protein PilU [Type IV pili (VF0082)] [Pseudomonas aeruginosa PAO1] | 100.00 | 100.00 | NaN | 273949 | 275097 | - | VFDB | 015 | paeruginosa | - | 4 | Pseudomonas aeruginosa |
| fliO | NP\_250136 | (fliO) flagellar protein FliO [Flagella (VF0273)] [Pseudomonas aeruginosa PAO1] | 100.00 | 100.00 | NaN | 208192 | 208644 | + | VFDB | 015 | paeruginosa | - | 8 | Pseudomonas aeruginosa |
| pilT | NP\_249086 | (pilT) twitching motility protein PilT [Type IV pili (VF0082)] [Pseudomonas aeruginosa PAO1] | 99.61 | 100.00 | NaN | 275275 | 276309 | - | VFDB | 015 | paeruginosa | - | 4 | Pseudomonas aeruginosa |
| lasB | NP\_252413 | (lasB) elastase LasB [LasB (VF0087)] [Pseudomonas aeruginosa PAO1] | 99.80 | 100.00 | NaN | 160180 | 161676 | - | VFDB | 015 | paeruginosa | - | 5 | Pseudomonas aeruginosa |
| fliM | NP\_250134 | (fliM) flagellar motor switch protein FliM [Flagella (VF0273)] [Pseudomonas aeruginosa PAO1] | 99.90 | 100.00 | NaN | 206718 | 207689 | + | VFDB | 015 | paeruginosa | - | 8 | Pseudomonas aeruginosa |
| fliL | NP\_250133 | (fliL) flagellar basal body protein FliL [Deoxyhexose linking sugar 209 Da capping structure (AI138)] [Pseudomonas aeruginosa PAO1] | 100.00 | 100.00 | NaN | 206189 | 206710 | + | VFDB | 015 | paeruginosa | - | 8 | Pseudomonas aeruginosa |
| fliK | NP\_250132 | (fliK) flagellar hook-length control protein FliK [Deoxyhexose linking sugar 209 Da capping structure (AI138)] [Pseudomonas aeruginosa PAO1] | 98.91 | 100.00 | NaN | 204662 | 205945 | + | VFDB | 015 | paeruginosa | - | 8 | Pseudomonas aeruginosa |
| lasI | NP\_250123 | (lasI) autoinducer synthesis protein LasI [Quorum sensing (VF0093)] [Pseudomonas aeruginosa PAO1] | 100.00 | 100.00 | NaN | 193420 | 194025 | + | VFDB | 015 | paeruginosa | - | 8 | Pseudomonas aeruginosa |
| aprA | NP\_249940 | (aprA) alkaline metalloproteinase precursor [Alkaline protease (VF0090)] [Pseudomonas aeruginosa PAO1] | 99.38 | 100.00 | NaN | 15999 | 17438 | + | VFDB | 015 | paeruginosa | - | 8 | Pseudomonas aeruginosa |
| algU | NP\_249453 | (algU) alginate biosynthesis protein AlgZ/FimS [Alginate (VF0091)] [Pseudomonas aeruginosa PAO1] | 100.00 | 100.00 | NaN | 182612 | 183193 | - | VFDB | 015 | paeruginosa | - | 7 | Pseudomonas aeruginosa |
| mucP | NP\_252339 | (mucP) metalloprotease protease [Alginate regulation (CVF523)] [Pseudomonas aeruginosa PAO1] | 99.78 | 100.00 | NaN | 78730 | 80082 | - | VFDB | 015 | paeruginosa | - | 5 | Pseudomonas aeruginosa |
| mucA | NP\_249454 | (mucA) alkaline metalloproteinase precursor [Alginate (VF0091)] [Pseudomonas aeruginosa PAO1] | 99.66 | 99.83 | NaN | 181997 | 182580 | - | VFDB | 015 | paeruginosa | - | 7 | Pseudomonas aeruginosa |
| mucC | NP\_249456 | (mucC) negative regulator for alginate biosynthesis MucB [Alginate (VF0091)] [Pseudomonas aeruginosa PAO1] | 99.78 | 100.00 | NaN | 180586 | 181041 | - | VFDB | 015 | paeruginosa | - | 7 | Pseudomonas aeruginosa |
| mucD | NP\_249457 | (mucD) serine protease MucD precursor [Alginate regulation (CVF523)] [Pseudomonas aeruginosa PAO1] | 99.79 | 100.00 | NaN | 179122 | 180546 | - | VFDB | 015 | paeruginosa | - | 7 | Pseudomonas aeruginosa |
| plcH | NP\_249535 | (plcH) hemolytic phospholipase C precursor [PLC (VF0092)] [Pseudomonas aeruginosa PAO1] | 99.68 | 100.00 | NaN | 92948 | 95140 | + | VFDB | 015 | paeruginosa | - | 7 | Pseudomonas aeruginosa |
| exoS | NP\_252530 | (exoS) type III secretion system effector ExoS ADP ribosyltransferase activity and GTPase-activating protein activity [ExoS (VF0096)] [Pseudomonas aeruginosa PAO1] | 99.93 | 100.00 | NaN | 302756 | 304117 | - | VFDB | 015 | paeruginosa | - | 5 | Pseudomonas aeruginosa |
| pilF | NP\_252494 | (pilF) type 4 fimbrial biogenesis protein PilF [Type IV pili (VF0082)] [Pseudomonas aeruginosa PAO1] | 99.74 | 100.00 | NaN | 262547 | 263305 | - | VFDB | 015 | paeruginosa | - | 5 | Pseudomonas aeruginosa |
| mucB | NP\_249455 | (mucB) anti-sigma factor MucA inhibitor of alg gene expression [Alginate (VF0091)] [Pseudomonas aeruginosa PAO1] | 100.00 | 100.00 | NaN | 181038 | 181988 | - | VFDB | 015 | paeruginosa | - | 7 | Pseudomonas aeruginosa |
| fliH | NP\_249794 | (fliH) flagellar assembly protein H [Flagella (VF0273)] [Pseudomonas aeruginosa PAO1] | 99.75 | 100.00 | NaN | 208097 | 208903 | + | VFDB | 015 | paeruginosa | - | 11 | Pseudomonas aeruginosa |
| phzC1 | NP\_252901 | (phzC1) phenazine biosynthesis protein PhzC [Phenazines biosynthesis (CVF536)] [Pseudomonas aeruginosa PAO1] | 99.75 | 100.00 | NaN | 38 | 1255 | - | VFDB | 016 | paeruginosa | 395 | 51 | Pseudomonas aeruginosa |
| phzD1 | NP\_252902 | (phzD1) phenazine biosynthesis protein PhzD isochorismatase [Phenazines biosynthesis (CVF536)] [Pseudomonas aeruginosa PAO1] | 98.88 | 100.00 | NaN | 281 | 904 | - | VFDB | 016 | paeruginosa | 395 | 52 | Pseudomonas aeruginosa |
| algL | NP\_252237 | (algL) poly(beta-d-mannuronate) lyase precursor AlgL [Alginate (VF0091)] [Pseudomonas aeruginosa PAO1] | 99.82 | 100.00 | NaN | 170615 | 171718 | + | VFDB | 016 | paeruginosa | 395 | 2 | Pseudomonas aeruginosa |
| pilU | NP\_249087 | (pilU) twitching motility protein PilU [Type IV pili (VF0082)] [Pseudomonas aeruginosa PAO1] | 100.00 | 100.00 | NaN | 296593 | 297741 | - | VFDB | 016 | paeruginosa | 395 | 4 | Pseudomonas aeruginosa |
| pilG | NP\_249099 | (pilG) twitching motility protein PilG [Type IV pili (VF0082)] [Pseudomonas aeruginosa PAO1] | 100.00 | 100.00 | NaN | 285477 | 285884 | - | VFDB | 016 | paeruginosa | 395 | 4 | Pseudomonas aeruginosa |
| pilH | NP\_249100 | (pilH) twitching motility protein PilH [Type IV pili (VF0082)] [Pseudomonas aeruginosa PAO1] | 100.00 | 100.00 | NaN | 285065 | 285430 | - | VFDB | 016 | paeruginosa | 395 | 4 | Pseudomonas aeruginosa |
| pilI | NP\_249101 | (pilI) twitching motility protein PilI [Type IV pili (VF0082)] [Pseudomonas aeruginosa PAO1] | 99.81 | 100.00 | NaN | 284478 | 285014 | - | VFDB | 016 | paeruginosa | 395 | 4 | Pseudomonas aeruginosa |
| pilJ | NP\_249102 | (pilJ) twitching motility protein PilJ [Type IV pili (VF0082)] [Pseudomonas aeruginosa PAO1] | 99.85 | 100.00 | NaN | 282345 | 284393 | - | VFDB | 016 | paeruginosa | 395 | 4 | Pseudomonas aeruginosa |
| pilK | NP\_249103 | (pilK) methyltransferase PilK [Type IV pili (VF0082)] [Pseudomonas aeruginosa PAO1] | 99.66 | 100.00 | NaN | 281409 | 282284 | - | VFDB | 016 | paeruginosa | 395 | 4 | Pseudomonas aeruginosa |
| chpA | NP\_249104 | (chpA) still frameshift probable component of chemotactic signal transduction system [Type IV pili (VF0082)] [Pseudomonas aeruginosa PAO1] | 99.73 | 100.00 | NaN | 273979 | 281397 | - | VFDB | 016 | paeruginosa | 395 | 4 | Pseudomonas aeruginosa |
| chpB | NP\_249105 | (chpB) probable methylesterase [Type IV pili (VF0082)] [Pseudomonas aeruginosa PAO1] | 99.13 | 100.00 | NaN | 272955 | 273986 | - | VFDB | 016 | paeruginosa | 395 | 4 | Pseudomonas aeruginosa |
| chpC | NP\_249106 | (chpC) probable chemotaxis protein [Type IV pili (VF0082)] [Pseudomonas aeruginosa PAO1] | 98.82 | 100.00 | NaN | 272452 | 272958 | - | VFDB | 016 | paeruginosa | 395 | 4 | Pseudomonas aeruginosa |
| chpD | NP\_249107 | (chpD) probable transcriptional regulator [Type IV pili (VF0082)] [Pseudomonas aeruginosa PAO1] | 99.25 | 100.00 | NaN | 271650 | 272444 | - | VFDB | 016 | paeruginosa | 395 | 4 | Pseudomonas aeruginosa |
| chpE | NP\_249108 | (chpE) probable chemotaxis protein [Type IV pili (VF0082)] [Pseudomonas aeruginosa PAO1] | 99.18 | 100.00 | NaN | 270962 | 271573 | - | VFDB | 016 | paeruginosa | 395 | 4 | Pseudomonas aeruginosa |
| algU | NP\_249453 | (algU) alginate biosynthesis protein AlgZ/FimS [Alginate (VF0091)] [Pseudomonas aeruginosa PAO1] | 100.00 | 100.00 | NaN | 299900 | 300481 | - | VFDB | 016 | paeruginosa | 395 | 3 | Pseudomonas aeruginosa |
| mucA | NP\_249454 | (mucA) alkaline metalloproteinase precursor [Alginate (VF0091)] [Pseudomonas aeruginosa PAO1] | 99.49 | 100.00 | NaN | 299284 | 299868 | - | VFDB | 016 | paeruginosa | 395 | 3 | Pseudomonas aeruginosa |
| mucB | NP\_249455 | (mucB) anti-sigma factor MucA inhibitor of alg gene expression [Alginate (VF0091)] [Pseudomonas aeruginosa PAO1] | 100.00 | 100.00 | NaN | 298325 | 299275 | - | VFDB | 016 | paeruginosa | 395 | 3 | Pseudomonas aeruginosa |
| mucC | NP\_249456 | (mucC) negative regulator for alginate biosynthesis MucB [Alginate (VF0091)] [Pseudomonas aeruginosa PAO1] | 99.56 | 100.00 | NaN | 297873 | 298328 | - | VFDB | 016 | paeruginosa | 395 | 3 | Pseudomonas aeruginosa |
| pilT | NP\_249086 | (pilT) twitching motility protein PilT [Type IV pili (VF0082)] [Pseudomonas aeruginosa PAO1] | 99.90 | 100.00 | NaN | 297919 | 298953 | - | VFDB | 016 | paeruginosa | 395 | 4 | Pseudomonas aeruginosa |
| xcpZ | NP\_251785 | (xcpZ) general secretion pathway protein M [xcp secretion system (VF0084)] [Pseudomonas aeruginosa PAO1] | 99.81 | 100.00 | NaN | 379659 | 380183 | - | VFDB | 016 | paeruginosa | 395 | 5 | Pseudomonas aeruginosa |
| xcpY | NP\_251786 | (xcpY) general secretion pathway protein L [xcp secretion system (VF0084)] [Pseudomonas aeruginosa PAO1] | 99.65 | 100.00 | NaN | 380185 | 381333 | - | VFDB | 016 | paeruginosa | 395 | 5 | Pseudomonas aeruginosa |
| xcpX | NP\_251787 | (xcpX) general secretion pathway protein K [xcp secretion system (VF0084)] [Pseudomonas aeruginosa PAO1] | 99.50 | 100.00 | NaN | 381330 | 382331 | - | VFDB | 016 | paeruginosa | 395 | 5 | Pseudomonas aeruginosa |
| pvdP | NP\_251082 | (pvdP) tyrosinase required for pyoverdine maturation [pyoverdine (IA001)] [Pseudomonas aeruginosa PAO1] | 99.45 | 100.00 | NaN | 17231 | 18865 | + | VFDB | 016 | paeruginosa | 395 | 6 | Pseudomonas aeruginosa |
| pvdM | NP\_251083 | (pvdM) dipeptidase precursor [pyoverdine (IA001)] [Pseudomonas aeruginosa PAO1] | 99.85 | 100.00 | NaN | 15669 | 17015 | - | VFDB | 016 | paeruginosa | 395 | 6 | Pseudomonas aeruginosa |
| pvdN | NP\_251084 | (pvdN) pyoverdine biosynthesis protein PvdN [pyoverdine (IA001)] [Pseudomonas aeruginosa PAO1] | 99.92 | 100.00 | NaN | 14363 | 15646 | - | VFDB | 016 | paeruginosa | 395 | 6 | Pseudomonas aeruginosa |
| pvdO | NP\_251085 | (pvdO) pyoverdine biosynthesis protein PvdO [pyoverdine (IA001)] [Pseudomonas aeruginosa PAO1] | 99.30 | 100.00 | NaN | 13480 | 14334 | - | VFDB | 016 | paeruginosa | 395 | 6 | Pseudomonas aeruginosa |
| pvdF | NP\_251086 | (pvdF) pyoverdine synthetase F [pyoverdine (IA001)] [Pseudomonas aeruginosa PAO1] | 99.28 | 100.00 | NaN | 12584 | 13411 | + | VFDB | 016 | paeruginosa | 395 | 6 | Pseudomonas aeruginosa |
| pvdE | NP\_251087 | (pvdE) pyoverdine biosynthesis protein PvdE [Pyoverdine (VF0094)] [Pseudomonas aeruginosa PAO1] | 99.76 | 100.00 | NaN | 10557 | 12206 | - | VFDB | 016 | paeruginosa | 395 | 6 | Pseudomonas aeruginosa |
| fpvA | NP\_251088 | (fpvA) ferripyoverdine receptor FpvA [Pyoverdine (VF0094)] [Pseudomonas aeruginosa PAO1] | 99.71 | 100.00 | NaN | 8007 | 10454 | - | VFDB | 016 | paeruginosa | 395 | 6 | Pseudomonas aeruginosa |
| mucD | NP\_249457 | (mucD) serine protease MucD precursor [Alginate regulation (CVF523)] [Pseudomonas aeruginosa PAO1] | 99.79 | 100.00 | NaN | 296409 | 297833 | - | VFDB | 016 | paeruginosa | 395 | 3 | Pseudomonas aeruginosa |
| pvdD | NP\_251089 | (pvdD) pyoverdine synthetase D [Pyoverdine (VF0094)] [Pseudomonas aeruginosa PAO1] | 99.25 | 100.00 | NaN | 497 | 7843 | + | VFDB | 016 | paeruginosa | 395 | 6 | Pseudomonas aeruginosa |
| xcpP | NP\_251794 | (xcpP) secretion protein XcpP [xcp secretion system (VF0084)] [Pseudomonas aeruginosa PAO1] | 100.00 | 100.00 | NaN | 387345 | 388052 | + | VFDB | 016 | paeruginosa | 395 | 5 | Pseudomonas aeruginosa |
| xcpR | NP\_251793 | (xcpR) general secretion pathway protein E [xcp secretion system (VF0084)] [Pseudomonas aeruginosa PAO1] | 99.60 | 100.00 | NaN | 385617 | 387125 | - | VFDB | 016 | paeruginosa | 395 | 5 | Pseudomonas aeruginosa |
| xcpS | NP\_251792 | (xcpS) general secretion pathway protein F [xcp secretion system (VF0084)] [Pseudomonas aeruginosa PAO1] | 99.51 | 100.00 | NaN | 384400 | 385617 | - | VFDB | 016 | paeruginosa | 395 | 5 | Pseudomonas aeruginosa |
| xcpT | NP\_251791 | (xcpT) general secretion pathway protein G [xcp secretion system (VF0084)] [Pseudomonas aeruginosa PAO1] | 99.78 | 100.00 | NaN | 383949 | 384395 | - | VFDB | 016 | paeruginosa | 395 | 5 | Pseudomonas aeruginosa |
| xcpU | NP\_251790 | (xcpU) general secretion pathway protein H [xcp secretion system (VF0084)] [Pseudomonas aeruginosa PAO1] | 100.00 | 100.00 | NaN | 383424 | 383942 | - | VFDB | 016 | paeruginosa | 395 | 5 | Pseudomonas aeruginosa |
| xcpV | NP\_251789 | (xcpV) general secretion pathway protein I [xcp secretion system (VF0084)] [Pseudomonas aeruginosa PAO1] | 100.00 | 100.00 | NaN | 383038 | 383427 | - | VFDB | 016 | paeruginosa | 395 | 5 | Pseudomonas aeruginosa |
| xcpW | NP\_251788 | (xcpW) general secretion pathway protein J [xcp secretion system (VF0084)] [Pseudomonas aeruginosa PAO1] | 99.58 | 100.00 | NaN | 382328 | 383041 | - | VFDB | 016 | paeruginosa | 395 | 5 | Pseudomonas aeruginosa |
| xcpQ | NP\_251795 | (xcpQ) general secretion pathway protein D [xcp secretion system (VF0084)] [Pseudomonas aeruginosa PAO1] | 99.59 | 100.00 | NaN | 388057 | 390033 | + | VFDB | 016 | paeruginosa | 395 | 5 | Pseudomonas aeruginosa |
| pvdA | NP\_251076 | (pvdA) L-ornithine N5-oxygenase PvdA [Pyoverdine (VF0094)] [Pseudomonas aeruginosa PAO1] | 99.47 | 100.00 | NaN | 25383 | 26714 | + | VFDB | 016 | paeruginosa | 395 | 6 | Pseudomonas aeruginosa |
| plcH | NP\_249535 | (plcH) hemolytic phospholipase C precursor [PLC (VF0092)] [Pseudomonas aeruginosa PAO1] | 99.68 | 100.00 | NaN | 168521 | 170713 | + | VFDB | 016 | paeruginosa | 395 | 3 | Pseudomonas aeruginosa |
| pilF | NP\_252494 | (pilF) type 4 fimbrial biogenesis protein PilF [Type IV pili (VF0082)] [Pseudomonas aeruginosa PAO1] | 99.74 | 100.00 | NaN | 468777 | 469535 | - | VFDB | 016 | paeruginosa | 395 | 2 | Pseudomonas aeruginosa |
| algZ | NP\_253949 | (algZ) sigma factor AlgU [Alginate (VF0091)] [Pseudomonas aeruginosa PAO1] | 99.91 | 100.00 | NaN | 699853 | 700929 | - | VFDB | 016 | paeruginosa | 395 | 1 | Pseudomonas aeruginosa |
| algR | NP\_253948 | (algR) alginate biosynthesis regulatory protein AlgR [Alginate (VF0091)] [Pseudomonas aeruginosa PAO1] | 99.47 | 100.00 | NaN | 699102 | 699848 | - | VFDB | 016 | paeruginosa | 395 | 1 | Pseudomonas aeruginosa |
| algQ | NP\_253942 | (algQ) Alginate regulatory protein AlgQ [Alginate (VF0091)] [Pseudomonas aeruginosa PAO1] | 98.55 | 100.00 | NaN | 693475 | 693957 | - | VFDB | 016 | paeruginosa | 395 | 1 | Pseudomonas aeruginosa |
| algP/algR3 | NP\_253940 | (algP/algR3) alginate regulatory protein AlgP [Alginate (VF0091)] [Pseudomonas aeruginosa PAO1] | 97.17 | 97.73 | NaN | 691624 | 692658 | - | VFDB | 016 | paeruginosa | 395 | 1 | Pseudomonas aeruginosa |
| pilM | NP\_253731 | (pilM) type IV pilus inner membrane platform protein PilM [Type IV pili (VF0082)] [Pseudomonas aeruginosa PAO1] | 99.81 | 100.00 | NaN | 410648 | 411712 | - | VFDB | 016 | paeruginosa | 395 | 1 | Pseudomonas aeruginosa |
| pilN | NP\_253730 | (pilN) type IV pilus inner membrane platform protein PilN [Type IV pili (VF0082)] [Pseudomonas aeruginosa PAO1] | 99.83 | 100.00 | NaN | 410052 | 410648 | - | VFDB | 016 | paeruginosa | 395 | 1 | Pseudomonas aeruginosa |
| pilO | NP\_253729 | (pilO) type IV pilus inner membrane platform protein PilO [Type IV pili (VF0082)] [Pseudomonas aeruginosa PAO1] | 99.84 | 100.00 | NaN | 409432 | 410055 | - | VFDB | 016 | paeruginosa | 395 | 1 | Pseudomonas aeruginosa |
| pilP | NP\_253728 | (pilP) type IV pilus biogenesis protein PilP [Type IV pili (VF0082)] [Pseudomonas aeruginosa PAO1] | 100.00 | 100.00 | NaN | 408911 | 409435 | - | VFDB | 016 | paeruginosa | 395 | 1 | Pseudomonas aeruginosa |
| pilQ | NP\_253727 | (pilQ) type 4 fimbrial biogenesis protein PilQ [Type IV pili (VF0082)] [Pseudomonas aeruginosa PAO1] | 97.06 | 100.00 | NaN | 406713 | 408857 | - | VFDB | 016 | paeruginosa | 395 | 1 | Pseudomonas aeruginosa |
| waaF | NP\_253699 | (waaF) heptosyltransferase I [LPS (VF0085)] [Pseudomonas aeruginosa PAO1] | 99.90 | 100.00 | NaN | 361852 | 362889 | - | VFDB | 016 | paeruginosa | 395 | 1 | Pseudomonas aeruginosa |
| waaC | NP\_253698 | (waaC) 3-deoxy-D-manno-octulosonic-acid (KDO) transferase [LPS (VF0085)] [Pseudomonas aeruginosa PAO1] | 99.72 | 100.00 | NaN | 360788 | 361855 | - | VFDB | 016 | paeruginosa | 395 | 1 | Pseudomonas aeruginosa |
| waaG | NP\_253697 | (waaG) B-band O-antigen polymerase [LPS (VF0085)] [Pseudomonas aeruginosa PAO1] | 99.73 | 100.00 | NaN | 359670 | 360791 | - | VFDB | 016 | paeruginosa | 395 | 1 | Pseudomonas aeruginosa |
| waaP | NP\_253696 | (waaP) UDP-glucose:(heptosyl) LPS alpha 13-glucosyltransferase WaaG [LPS (VF0085)] [Pseudomonas aeruginosa PAO1] | 99.75 | 100.00 | NaN | 358867 | 359673 | - | VFDB | 016 | paeruginosa | 395 | 1 | Pseudomonas aeruginosa |
| waaA | NP\_253675 | (waaA) lipopolysaccharide core biosynthesis protein WaaP [LPS (VF0085)] [Pseudomonas aeruginosa PAO1] | 99.92 | 100.00 | NaN | 332483 | 333760 | - | VFDB | 016 | paeruginosa | 395 | 1 | Pseudomonas aeruginosa |
| motA | NP\_253641 | (motA) flagellar motor protein [Deoxyhexose linking sugar 209 Da capping structure (AI138)] [Pseudomonas aeruginosa PAO1] | 99.41 | 100.00 | NaN | 287683 | 288534 | - | VFDB | 016 | paeruginosa | 395 | 1 | Pseudomonas aeruginosa |
| algC | NP\_254009 | (algC) phosphomannomutase AlgC [Alginate biosynthesis (CVF522)] [Pseudomonas aeruginosa PAO1] | 100.00 | 100.00 | NaN | 768970 | 770361 | + | VFDB | 016 | paeruginosa | 395 | 1 | Pseudomonas aeruginosa |
| rhlI | NP\_252166 | (rhlI) autoinducer synthesis protein RhlL [Quorum sensing (VF0093)] [Pseudomonas aeruginosa PAO1] | 98.52 | 100.00 | NaN | 110445 | 111050 | - | VFDB | 016 | paeruginosa | 395 | 2 | Pseudomonas aeruginosa |
| rhlB | NP\_252168 | (rhlB) rhamnosyltransferase chain B [Rhamnolipid (VF0089)] [Pseudomonas aeruginosa PAO1] | 99.22 | 100.00 | NaN | 112080 | 113360 | - | VFDB | 016 | paeruginosa | 395 | 2 | Pseudomonas aeruginosa |
| rhlA | NP\_252169 | (rhlA) rhamnosyltransferase chain A [Rhamnolipid (VF0089)] [Pseudomonas aeruginosa PAO1] | 99.55 | 100.00 | NaN | 113426 | 114313 | - | VFDB | 016 | paeruginosa | 395 | 2 | Pseudomonas aeruginosa |
| lasB | NP\_252413 | (lasB) elastase LasB [LasB (VF0087)] [Pseudomonas aeruginosa PAO1] | 100.00 | 100.00 | NaN | 366385 | 367881 | - | VFDB | 016 | paeruginosa | 395 | 2 | Pseudomonas aeruginosa |
| mucP | NP\_252339 | (mucP) metalloprotease protease [Alginate regulation (CVF523)] [Pseudomonas aeruginosa PAO1] | 99.93 | 100.00 | NaN | 284910 | 286262 | - | VFDB | 016 | paeruginosa | 395 | 2 | Pseudomonas aeruginosa |
| algA | NP\_252241 | (algA) phosphomannose isomerase / guanosine 5'-diphospho-D-mannose pyrophosphorylase [Alginate (VF0091)] [Pseudomonas aeruginosa PAO1] | 99.93 | 100.00 | NaN | 175632 | 177077 | + | VFDB | 016 | paeruginosa | 395 | 2 | Pseudomonas aeruginosa |
| algF | NP\_252240 | (algF) alginate o-acetyltransferase AlgF [Alginate (VF0091)] [Pseudomonas aeruginosa PAO1] | 99.54 | 100.00 | NaN | 174785 | 175435 | + | VFDB | 016 | paeruginosa | 395 | 2 | Pseudomonas aeruginosa |
| algJ | NP\_252239 | (algJ) alginate o-acetyltransferase AlgJ [Alginate (VF0091)] [Pseudomonas aeruginosa PAO1] | 99.15 | 100.00 | NaN | 173537 | 174712 | + | VFDB | 016 | paeruginosa | 395 | 2 | Pseudomonas aeruginosa |
| algI | NP\_252238 | (algI) alginate o-acetyltransferase AlgI [Alginate (VF0091)] [Pseudomonas aeruginosa PAO1] | 99.68 | 100.00 | NaN | 171960 | 173522 | + | VFDB | 016 | paeruginosa | 395 | 2 | Pseudomonas aeruginosa |
| hsiJ1 | NP\_248769 | (hsiJ1) type VI secretion system hcp secretion island protein HsiJ1 [HSI-I (VF0334)] [Pseudomonas aeruginosa PAO1] | 99.62 | 100.00 | NaN | 7366 | 8700 | - | VFDB | 016 | paeruginosa | 395 | 13 | Pseudomonas aeruginosa |
| exoS | NP\_252530 | (exoS) type III secretion system effector ExoS ADP ribosyltransferase activity and GTPase-activating protein activity [ExoS (VF0096)] [Pseudomonas aeruginosa PAO1] | 99.34 | 100.00 | NaN | 512452 | 513813 | - | VFDB | 016 | paeruginosa | 395 | 2 | Pseudomonas aeruginosa |
| algX | NP\_252236 | (algX) alginate biosynthesis protein AlgX [Alginate (VF0091)] [Pseudomonas aeruginosa PAO1] | 99.93 | 100.00 | NaN | 169187 | 170611 | + | VFDB | 016 | paeruginosa | 395 | 2 | Pseudomonas aeruginosa |
| algE | NP\_252234 | (algE) alginate biosynthetic protein AlgK precursor [Alginate (VF0091)] [Pseudomonas aeruginosa PAO1] | 99.32 | 100.00 | NaN | 166050 | 167522 | + | VFDB | 016 | paeruginosa | 395 | 2 | Pseudomonas aeruginosa |
| algK | NP\_252233 | (algK) alginate biosynthesis protein Alg44 [Alginate (VF0091)] [Pseudomonas aeruginosa PAO1] | 99.86 | 100.00 | NaN | 164626 | 166053 | + | VFDB | 016 | paeruginosa | 395 | 2 | Pseudomonas aeruginosa |
| alg44 | NP\_252232 | (alg44) alginate biosynthesis protein Alg8 [Alginate (VF0091)] [Pseudomonas aeruginosa PAO1] | 99.57 | 100.00 | NaN | 163443 | 164612 | + | VFDB | 016 | paeruginosa | 395 | 2 | Pseudomonas aeruginosa |
| alg8 | NP\_252231 | (alg8) alginate-c5-mannuronan-epimerase AlgG [Alginate (VF0091)] [Pseudomonas aeruginosa PAO1] | 99.86 | 100.00 | NaN | 161876 | 163360 | + | VFDB | 016 | paeruginosa | 395 | 2 | Pseudomonas aeruginosa |
| algD | NP\_252230 | (algD) GDP-mannose 6-dehydrogenase AlgD [Alginate (VF0091)] [Pseudomonas aeruginosa PAO1] | 99.92 | 100.00 | NaN | 160426 | 161736 | + | VFDB | 016 | paeruginosa | 395 | 2 | Pseudomonas aeruginosa |
| motY | NP\_252216 | (motY) probable outer membrane protein precursor [Deoxyhexose linking sugar 209 Da capping structure (AI138)] [Pseudomonas aeruginosa PAO1] | 100.00 | 100.00 | NaN | 143597 | 144562 | - | VFDB | 016 | paeruginosa | 395 | 2 | Pseudomonas aeruginosa |
| tse3 | NP\_252174 | (tse3) type VI secretion system effector Tse3 glycoside hydrolase [HSI-1 (Hcp-secretion island 1) (SS178)] [Pseudomonas aeruginosa PAO1] | 99.59 | 100.00 | NaN | 119583 | 120809 | + | VFDB | 016 | paeruginosa | 395 | 2 | Pseudomonas aeruginosa |
| algG | NP\_252235 | (algG) outer membrane protein AlgE [Alginate (VF0091)] [Pseudomonas aeruginosa PAO1] | 99.94 | 100.00 | NaN | 167543 | 169174 | + | VFDB | 016 | paeruginosa | 395 | 2 | Pseudomonas aeruginosa |
| pvdQ | NP\_251075 | (pvdQ) 3-oxo-C12-homoserine lactone acylase PvdQ [pyoverdine (IA001)] [Pseudomonas aeruginosa PAO1] | 99.61 | 100.00 | NaN | 26837 | 29125 | + | VFDB | 016 | paeruginosa | 395 | 6 | Pseudomonas aeruginosa |
| ptxR | NP\_250948 | (ptxR) transcriptional regulator PtxR [pyoverdine (IA001)] [Pseudomonas aeruginosa PAO1] | 99.57 | 100.00 | NaN | 176272 | 177210 | + | VFDB | 016 | paeruginosa | 395 | 6 | Pseudomonas aeruginosa |
| pvcD | NP\_250947 | (pvcD) paerucumarin biosynthesis protein PvcD [pyoverdine (IA001)] [Pseudomonas aeruginosa PAO1] | 99.85 | 100.00 | NaN | 177444 | 178091 | - | VFDB | 016 | paeruginosa | 395 | 6 | Pseudomonas aeruginosa |
| fliI | NP\_249795 | (fliI) flagellum-specific ATP synthase FliI [Flagella (VF0273)] [Pseudomonas aeruginosa PAO1] | 99.26 | 100.00 | NaN | 143983 | 145338 | - | VFDB | 016 | paeruginosa | 395 | 11 | Pseudomonas aeruginosa |
| fliJ | NP\_249796 | (fliJ) flagellar protein FliJ [Flagella (VF0273)] [Pseudomonas aeruginosa PAO1] | 99.78 | 100.00 | NaN | 143526 | 143969 | - | VFDB | 016 | paeruginosa | 395 | 11 | Pseudomonas aeruginosa |
| rhlC | NP\_249821 | (rhlC) rhamnosyltransferase 2 [Rhamnolipid biosynthesis (CVF524)] [Pseudomonas aeruginosa PAO1] | 99.90 | 100.00 | NaN | 118284 | 119261 | + | VFDB | 016 | paeruginosa | 395 | 11 | Pseudomonas aeruginosa |
| toxA | NP\_249839 | (toxA) exotoxin A precursor [ExoA (VF0086)] [Pseudomonas aeruginosa PAO1] | 99.58 | 99.95 | NaN | 98857 | 100772 | + | VFDB | 016 | paeruginosa | 395 | 11 | Pseudomonas aeruginosa |
| tse2 | NP\_251392 | (tse2) type VI secretion system effector Tse2 [HSI-1 (Hcp-secretion island 1) (SS178)] [Pseudomonas aeruginosa PAO1] | 100.00 | 100.00 | NaN | 32473 | 32949 | - | VFDB | 016 | paeruginosa | 395 | 10 | Pseudomonas aeruginosa |
| fimV | NP\_251805 | (fimV) putative Type IV pili related protein [Type IV pili (VF0082)] [Pseudomonas aeruginosa PAO1] | 99.49 | 100.00 | NaN | 276648 | 279407 | + | VFDB | 016 | paeruginosa | 395 | 9 | Pseudomonas aeruginosa |
| flgA | NP\_252040 | (flgA) flagellar basal body P-ring biosynthesis protein FlgA [Deoxyhexose linking sugar 209 Da capping structure (AI138)] [Pseudomonas aeruginosa PAO1] | 99.86 | 100.00 | NaN | 19821 | 20519 | - | VFDB | 016 | paeruginosa | 395 | 9 | Pseudomonas aeruginosa |
| flgM | NP\_252041 | (flgM) negative regulator of flagellin synthesis [Deoxyhexose linking sugar 209 Da capping structure (AI138)] [Pseudomonas aeruginosa PAO1] | 99.69 | 100.00 | NaN | 19353 | 19676 | - | VFDB | 016 | paeruginosa | 395 | 9 | Pseudomonas aeruginosa |
| flgN | NP\_252042 | (flgN) flagella synthesis protein FlgN [Deoxyhexose linking sugar 209 Da capping structure (AI138)] [Pseudomonas aeruginosa PAO1] | 100.00 | 100.00 | NaN | 18828 | 19298 | - | VFDB | 016 | paeruginosa | 395 | 9 | Pseudomonas aeruginosa |
| algB | NP\_254170 | (algB) two-component response regulator AlgB [Alginate (VF0091)] [Pseudomonas aeruginosa PAO1] | 100.00 | 100.00 | NaN | 179482 | 180831 | - | VFDB | 016 | paeruginosa | 395 | 8 | Pseudomonas aeruginosa |
| exoT | NP\_248734 | (exoT) type III secretion system effector ExoT ADP ribosyltransferase activity and GTPase-activating protein activity [ExoT (VF0097)] [Pseudomonas aeruginosa PAO1] | 99.20 | 100.00 | NaN | 28176 | 29549 | - | VFDB | 016 | paeruginosa | 395 | 8 | Pseudomonas aeruginosa |
| phzH | NP\_248741 | (phzH) phenazine-modifying enzyme [Phenazines biosynthesis (CVF536)] [Pseudomonas aeruginosa PAO1] | 99.84 | 100.00 | NaN | 20203 | 22035 | - | VFDB | 016 | paeruginosa | 395 | 8 | Pseudomonas aeruginosa |
| tagQ | NP\_248760 | (tagQ) type VI secretiona ssociated protein TagQ outer membrane lipoprotein [HSI-1 (Hcp-secretion island 1) (SS178)] [Pseudomonas aeruginosa PAO1] | 98.14 | 100.00 | NaN | 5737 | 6651 | + | VFDB | 016 | paeruginosa | 395 | 8 | Pseudomonas aeruginosa |
| tagR | NP\_248761 | (tagR) type IV secretion associated protein TagR positively regulates PpkA [HSI-I (VF0334)] [Pseudomonas aeruginosa PAO1] | 99.94 | 100.00 | NaN | 3963 | 5675 | + | VFDB | 016 | paeruginosa | 395 | 8 | Pseudomonas aeruginosa |
| tagS | NP\_248762 | (tagS) type IV secretion associated protein TagS forming a stable inner membrane complex with TagT [HSI-I (VF0334)] [Pseudomonas aeruginosa PAO1] | 98.92 | 100.00 | NaN | 2771 | 3970 | + | VFDB | 016 | paeruginosa | 395 | 8 | Pseudomonas aeruginosa |
| dotU1 | NP\_248768 | (dotU1) type VI secretion system protein DotU [HSI-I (VF0334)] [Pseudomonas aeruginosa PAO1] | 99.70 | 100.00 | NaN | 6010 | 7359 | - | VFDB | 016 | paeruginosa | 395 | 13 | Pseudomonas aeruginosa |
| fliH | NP\_249794 | (fliH) flagellar assembly protein H [Flagella (VF0273)] [Pseudomonas aeruginosa PAO1] | 99.26 | 100.00 | NaN | 145328 | 146134 | - | VFDB | 016 | paeruginosa | 395 | 11 | Pseudomonas aeruginosa |
| fliG | NP\_249793 | (fliG) flagellar motor switch protein G [Flagella (VF0273)] [Pseudomonas aeruginosa PAO1] | 99.80 | 100.00 | NaN | 146136 | 147152 | - | VFDB | 016 | paeruginosa | 395 | 11 | Pseudomonas aeruginosa |
| fliF | NP\_249792 | (fliF) flagellar M-ring protein FliF [Flagella (VF0273)] [Pseudomonas aeruginosa PAO1] | 99.78 | 100.00 | NaN | 147158 | 148954 | - | VFDB | 016 | paeruginosa | 395 | 11 | Pseudomonas aeruginosa |
| pppA | NP\_248765 | (pppA) Pseudomonas protein phosphatase PppA [HSI-I (VF0334)] [Pseudomonas aeruginosa PAO1] | 99.59 | 100.00 | NaN | 1068 | 1796 | - | VFDB | 016 | paeruginosa | 395 | 13 | Pseudomonas aeruginosa |
| algW | NP\_253136 | (algW) AlgW protein [Alginate regulation (CVF523)] [Pseudomonas aeruginosa PAO1] | 99.66 | 99.83 | NaN | 186213 | 187380 | + | VFDB | 016 | paeruginosa | 395 | 12 | Pseudomonas aeruginosa |
| flgB | NP\_249768 | (flgB) flagellar basal body rod protein FlgB [Deoxyhexose linking sugar 209 Da capping structure (AI138)] [Pseudomonas aeruginosa PAO1] | 100.00 | 100.00 | NaN | 185610 | 186017 | - | VFDB | 016 | paeruginosa | 395 | 11 | Pseudomonas aeruginosa |
| flgC | NP\_249769 | (flgC) flagellar basal-body rod protein FlgC [Flagella (VF0273)] [Pseudomonas aeruginosa PAO1] | 100.00 | 100.00 | NaN | 185164 | 185604 | - | VFDB | 016 | paeruginosa | 395 | 11 | Pseudomonas aeruginosa |
| flgD | NP\_249770 | (flgD) flagellar basal-body rod modification protein FlgD [Flagella (VF0273)] [Pseudomonas aeruginosa PAO1] | 100.00 | 100.00 | NaN | 184438 | 185151 | - | VFDB | 016 | paeruginosa | 395 | 11 | Pseudomonas aeruginosa |
| flgE | NP\_249771 | (flgE) flagellar hook protein FlgE [Flagella (VF0273)] [Pseudomonas aeruginosa PAO1] | 99.57 | 100.00 | NaN | 183022 | 184410 | - | VFDB | 016 | paeruginosa | 395 | 11 | Pseudomonas aeruginosa |
| flgF | NP\_249772 | (flgF) flagellar basal-body rod protein FlgF [Flagella (VF0273)] [Pseudomonas aeruginosa PAO1] | 99.87 | 100.00 | NaN | 182055 | 182804 | - | VFDB | 016 | paeruginosa | 395 | 11 | Pseudomonas aeruginosa |
| tagT | NP\_248763 | (tagT) type six secretion associated protein TagT ATP-binding component of ABC transporter [HSI-I (VF0334)] [Pseudomonas aeruginosa PAO1] | 99.31 | 100.00 | NaN | 2052 | 2771 | + | VFDB | 016 | paeruginosa | 395 | 8 | Pseudomonas aeruginosa |
| flgG | NP\_249773 | (flgG) flagellar basal-body rod protein FlgG [Flagella (VF0273)] [Pseudomonas aeruginosa PAO1] | 99.87 | 100.00 | NaN | 181223 | 182008 | - | VFDB | 016 | paeruginosa | 395 | 11 | Pseudomonas aeruginosa |
| flgI | NP\_249775 | (flgI) flagellar P-ring protein precursor FlgI [Flagella (VF0273)] [Pseudomonas aeruginosa PAO1] | 99.64 | 100.00 | NaN | 179361 | 180470 | - | VFDB | 016 | paeruginosa | 395 | 11 | Pseudomonas aeruginosa |
| flgJ | NP\_249776 | (flgJ) flagellar rod assembly protein/muramidase FlgJ [Flagella (VF0273)] [Pseudomonas aeruginosa PAO1] | 99.75 | 100.00 | NaN | 178148 | 179350 | - | VFDB | 016 | paeruginosa | 395 | 11 | Pseudomonas aeruginosa |
| flgK | NP\_249777 | (flgK) flagellar hook-associated protein 1 FlgK [Flagella (VF0273)] [Pseudomonas aeruginosa PAO1] | 86.57 | 99.71 | NaN | 176081 | 178129 | - | VFDB | 016 | paeruginosa | 395 | 11 | Pseudomonas aeruginosa |
| fleQ | NP\_249788 | (fleQ) transcriptional regulator FleQ [Flagella (VF0273)] [Pseudomonas aeruginosa PAO1] | 99.19 | 100.00 | NaN | 152300 | 153772 | - | VFDB | 016 | paeruginosa | 395 | 11 | Pseudomonas aeruginosa |
| fleS | NP\_249789 | (fleS) two-component sensor [Deoxyhexose linking sugar 209 Da capping structure (AI138)] [Pseudomonas aeruginosa PAO1] | 99.34 | 100.00 | NaN | 150979 | 152187 | - | VFDB | 016 | paeruginosa | 395 | 11 | Pseudomonas aeruginosa |
| fleR | NP\_249790 | (fleR) two-component response regulator [Flagella (VF0273)] [Pseudomonas aeruginosa PAO1] | 99.30 | 100.00 | NaN | 149553 | 150974 | - | VFDB | 016 | paeruginosa | 395 | 11 | Pseudomonas aeruginosa |
| fliE | NP\_249791 | (fliE) flagellar hook-basal body complex protein FliE [Flagella (VF0273)] [Pseudomonas aeruginosa PAO1] | 99.39 | 100.00 | NaN | 148977 | 149306 | - | VFDB | 016 | paeruginosa | 395 | 11 | Pseudomonas aeruginosa |
| flgH | NP\_249774 | (flgH) flagellar L-ring protein precursor FlgH [Flagella (VF0273)] [Pseudomonas aeruginosa PAO1] | 99.28 | 100.00 | NaN | 180482 | 181177 | - | VFDB | 016 | paeruginosa | 395 | 11 | Pseudomonas aeruginosa |
| pscL | NP\_250416 | (pscL) type III secretion systemt protein PscL [TTSS (VF0083)] [Pseudomonas aeruginosa PAO1] | 99.69 | 100.00 | NaN | 224545 | 225189 | + | VFDB | 016 | paeruginosa | 395 | 7 | Pseudomonas aeruginosa |
| pscK | NP\_250415 | (pscK) type III secretion system protein PscK [TTSS (VF0083)] [Pseudomonas aeruginosa PAO1] | 98.72 | 99.04 | NaN | 223946 | 224566 | + | VFDB | 016 | paeruginosa | 395 | 7 | Pseudomonas aeruginosa |
| pscJ | NP\_250414 | (pscJ) type III secretion system inner MS ring protein [TTSS (VF0083)] [Pseudomonas aeruginosa PAO1] | 99.47 | 100.00 | NaN | 223191 | 223937 | + | VFDB | 016 | paeruginosa | 395 | 7 | Pseudomonas aeruginosa |
| pcr4 | NP\_250393 | (pcr4) type III secretion system protein Pcr4 [TTSS (VF0083)] [Pseudomonas aeruginosa PAO1] | 99.70 | 100.00 | NaN | 208131 | 208460 | + | VFDB | 016 | paeruginosa | 395 | 7 | Pseudomonas aeruginosa |
| pcr3 | NP\_250392 | (pcr3) type III secretion system protein Pcr3 [TTSS (VF0083)] [Pseudomonas aeruginosa PAO1] | 100.00 | 100.00 | NaN | 207761 | 208126 | + | VFDB | 016 | paeruginosa | 395 | 7 | Pseudomonas aeruginosa |
| pcr2 | NP\_250391 | (pcr2) type III secretion system protein Pcr2 [TTSS (VF0083)] [Pseudomonas aeruginosa PAO1] | 98.92 | 100.00 | NaN | 207393 | 207764 | + | VFDB | 016 | paeruginosa | 395 | 7 | Pseudomonas aeruginosa |
| pcr1 | NP\_250390 | (pcr1) type III secretion system protein Pcr1 [TTSS (VF0083)] [Pseudomonas aeruginosa PAO1] | 100.00 | 100.00 | NaN | 207128 | 207406 | + | VFDB | 016 | paeruginosa | 395 | 7 | Pseudomonas aeruginosa |
| popN | NP\_250389 | (popN) type III secretion system outer membrane protein PopN [TTSS (VF0083)] [Pseudomonas aeruginosa PAO1] | 99.08 | 100.00 | NaN | 206281 | 207147 | + | VFDB | 016 | paeruginosa | 395 | 7 | Pseudomonas aeruginosa |
| pscN | NP\_250388 | (pscN) type III secretion system ATPase PscN [TTSS (VF0083)] [Pseudomonas aeruginosa PAO1] | 99.17 | 100.00 | NaN | 204768 | 206090 | - | VFDB | 016 | paeruginosa | 395 | 7 | Pseudomonas aeruginosa |
| pscO | NP\_250387 | (pscO) type III secretion system protein PscO [TTSS (VF0083)] [Pseudomonas aeruginosa PAO1] | 99.16 | 100.00 | NaN | 204295 | 204771 | - | VFDB | 016 | paeruginosa | 395 | 7 | Pseudomonas aeruginosa |
| pcrD | NP\_250394 | (pcrD) type III secretion system protein PcrD [TTSS (VF0083)] [Pseudomonas aeruginosa PAO1] | 99.48 | 100.00 | NaN | 208457 | 210577 | + | VFDB | 016 | paeruginosa | 395 | 7 | Pseudomonas aeruginosa |
| pscP | NP\_250386 | (pscP) type III secretion system protein PscP [TTSS (VF0083)] [Pseudomonas aeruginosa PAO1] | 95.62 | 97.84 | NaN | 203213 | 204307 | - | VFDB | 016 | paeruginosa | 395 | 7 | Pseudomonas aeruginosa |
| pscR | NP\_250384 | (pscR) type III secretion system protein PscR [TTSS (VF0083)] [Pseudomonas aeruginosa PAO1] | 99.54 | 100.00 | NaN | 201637 | 202290 | - | VFDB | 016 | paeruginosa | 395 | 7 | Pseudomonas aeruginosa |
| pscS | NP\_250383 | (pscS) type III secretion system protein PscS [TTSS (VF0083)] [Pseudomonas aeruginosa PAO1] | 99.62 | 100.00 | NaN | 201368 | 201634 | - | VFDB | 016 | paeruginosa | 395 | 7 | Pseudomonas aeruginosa |
| pscT | NP\_250382 | (pscT) type III secretion system protein PscT [TTSS (VF0083)] [Pseudomonas aeruginosa PAO1] | 99.24 | 100.00 | NaN | 200583 | 201371 | - | VFDB | 016 | paeruginosa | 395 | 7 | Pseudomonas aeruginosa |
| pscU | NP\_250381 | (pscU) type III secretion system protein PscU [TTSS (VF0083)] [Pseudomonas aeruginosa PAO1] | 99.24 | 100.00 | NaN | 199537 | 200586 | - | VFDB | 016 | paeruginosa | 395 | 7 | Pseudomonas aeruginosa |
| pvcA | NP\_250944 | (pvcA) paerucumarin biosynthesis protein PvcA [pyoverdine (IA001)] [Pseudomonas aeruginosa PAO1] | 99.49 | 100.00 | NaN | 180531 | 181517 | - | VFDB | 016 | paeruginosa | 395 | 6 | Pseudomonas aeruginosa |
| pvcB | NP\_250945 | (pvcB) paerucumarin biosynthesis protein PvcB [pyoverdine (IA001)] [Pseudomonas aeruginosa PAO1] | 99.66 | 100.00 | NaN | 179638 | 180513 | - | VFDB | 016 | paeruginosa | 395 | 6 | Pseudomonas aeruginosa |
| pvcC | NP\_250946 | (pvcC) paerucumarin biosynthesis protein PvcC [pyoverdine (IA001)] [Pseudomonas aeruginosa PAO1] | 99.27 | 100.00 | NaN | 178084 | 179586 | - | VFDB | 016 | paeruginosa | 395 | 6 | Pseudomonas aeruginosa |
| pscQ | NP\_250385 | (pscQ) type III secretion system protein PscQ [TTSS (VF0083)] [Pseudomonas aeruginosa PAO1] | 99.36 | 100.00 | NaN | 202287 | 203216 | - | VFDB | 016 | paeruginosa | 395 | 7 | Pseudomonas aeruginosa |
| motB | NP\_253640 | (motB) flagellar motor protein [Deoxyhexose linking sugar 209 Da capping structure (AI138)] [Pseudomonas aeruginosa PAO1] | 99.71 | 100.00 | NaN | 286620 | 287663 | - | VFDB | 016 | paeruginosa | 395 | 1 | Pseudomonas aeruginosa |
| pcrR | NP\_250395 | (pcrR) type III secretion system regulatory protein PcrR [TTSS (VF0083)] [Pseudomonas aeruginosa PAO1] | 99.77 | 100.00 | NaN | 210574 | 211008 | + | VFDB | 016 | paeruginosa | 395 | 7 | Pseudomonas aeruginosa |
| pcrV | NP\_250397 | (pcrV) type III secretion system hydrophilic translocator needle tip protein PcrV [TTSS (VF0083)] [Pseudomonas aeruginosa PAO1] | 99.89 | 100.00 | NaN | 211342 | 212226 | + | VFDB | 016 | paeruginosa | 395 | 7 | Pseudomonas aeruginosa |
| pscI | NP\_250413 | (pscI) type III secretion system inner rod protein PscI [TTSS (VF0083)] [Pseudomonas aeruginosa PAO1] | 99.70 | 100.00 | NaN | 222856 | 223194 | + | VFDB | 016 | paeruginosa | 395 | 7 | Pseudomonas aeruginosa |
| pscH | NP\_250412 | (pscH) type III secretion system protein PscH [TTSS (VF0083)] [Pseudomonas aeruginosa PAO1] | 99.77 | 100.00 | NaN | 222425 | 222856 | + | VFDB | 016 | paeruginosa | 395 | 7 | Pseudomonas aeruginosa |
| pscG | NP\_250411 | (pscG) type III secretion system chaperone PscG for PscF [TTSS (VF0083)] [Pseudomonas aeruginosa PAO1] | 99.14 | 100.00 | NaN | 222081 | 222428 | + | VFDB | 016 | paeruginosa | 395 | 7 | Pseudomonas aeruginosa |
| pscF | NP\_250410 | (pscF) type III secretion system needle filament protein PscF [TTSS (VF0083)] [Pseudomonas aeruginosa PAO1] | 99.61 | 100.00 | NaN | 221821 | 222078 | + | VFDB | 016 | paeruginosa | 395 | 7 | Pseudomonas aeruginosa |
| pscE | NP\_250409 | (pscE) type III secretion system cochaperone PscE for PscG [TTSS (VF0083)] [Pseudomonas aeruginosa PAO1] | 98.04 | 100.00 | NaN | 221615 | 221818 | + | VFDB | 016 | paeruginosa | 395 | 7 | Pseudomonas aeruginosa |
| pscD | NP\_250408 | (pscD) type III secretion system basal body protein PscD [TTSS (VF0083)] [Pseudomonas aeruginosa PAO1] | 99.77 | 100.00 | NaN | 220354 | 221652 | + | VFDB | 016 | paeruginosa | 395 | 7 | Pseudomonas aeruginosa |
| pscC | NP\_250407 | (pscC) type III secretion system secretin PscC [TTSS (VF0083)] [Pseudomonas aeruginosa PAO1] | 99.39 | 100.00 | NaN | 218550 | 220352 | + | VFDB | 016 | paeruginosa | 395 | 7 | Pseudomonas aeruginosa |
| pcrG | NP\_250396 | (pcrG) type III secretion system cytoplasmic regulator PcrG [TTSS (VF0083)] [Pseudomonas aeruginosa PAO1] | 99.33 | 100.00 | NaN | 211036 | 211332 | + | VFDB | 016 | paeruginosa | 395 | 7 | Pseudomonas aeruginosa |
| pscB | NP\_250406 | (pscB) type III secretion system protein PscB [TTSS (VF0083)] [Pseudomonas aeruginosa PAO1] | 99.76 | 100.00 | NaN | 218128 | 218550 | + | VFDB | 016 | paeruginosa | 395 | 7 | Pseudomonas aeruginosa |
| exsA | NP\_250404 | (exsA) type III secretion system regulatory protein ExsA [TTSS (VF0083)] [Pseudomonas aeruginosa PAO1] | 99.76 | 100.00 | NaN | 216330 | 217166 | + | VFDB | 016 | paeruginosa | 395 | 7 | Pseudomonas aeruginosa |
| exsB | NP\_250403 | (exsB) type III secretion system piolitin ExsB [TTSS (VF0083)] [Pseudomonas aeruginosa PAO1] | 99.52 | 100.00 | NaN | 215619 | 216032 | + | VFDB | 016 | paeruginosa | 395 | 7 | Pseudomonas aeruginosa |
| exsE | NP\_250402 | (exsE) type III secretion system regulatory protein ExsE [TTSS (VF0083)] [Pseudomonas aeruginosa PAO1] | 99.19 | 100.00 | NaN | 215365 | 215610 | + | VFDB | 016 | paeruginosa | 395 | 7 | Pseudomonas aeruginosa |
| exsC | NP\_250401 | (exsC) type III secretion system regulatory protein ExsC [TTSS (VF0083)] [Pseudomonas aeruginosa PAO1] | 98.86 | 100.00 | NaN | 214919 | 215356 | + | VFDB | 016 | paeruginosa | 395 | 7 | Pseudomonas aeruginosa |
| popD | NP\_250400 | (popD) type III secretion system hydrophobic translocator pore protein PopD [TTSS (VF0083)] [Pseudomonas aeruginosa PAO1] | 99.78 | 100.00 | NaN | 213906 | 214793 | + | VFDB | 016 | paeruginosa | 395 | 7 | Pseudomonas aeruginosa |
| popB | NP\_250399 | (popB) type III secretion system hydrophobic translocator pore protein PopB [TTSS (VF0083)] [Pseudomonas aeruginosa PAO1] | 99.57 | 100.00 | NaN | 212722 | 213894 | + | VFDB | 016 | paeruginosa | 395 | 7 | Pseudomonas aeruginosa |
| pcrH | NP\_250398 | (pcrH) type III secretion system regulatory protein PcrH [TTSS (VF0083)] [Pseudomonas aeruginosa PAO1] | 98.82 | 100.00 | NaN | 212235 | 212741 | + | VFDB | 016 | paeruginosa | 395 | 7 | Pseudomonas aeruginosa |
| exsD | NP\_250405 | (exsD) type III secretion system regulatory protein ExsD [TTSS (VF0083)] [Pseudomonas aeruginosa PAO1] | 99.52 | 100.00 | NaN | 217264 | 218094 | + | VFDB | 016 | paeruginosa | 395 | 7 | Pseudomonas aeruginosa |
| tagF/pppB | NP\_248766 | (tagF/pppB) Pseudomonas protein phosphatase PppB [HSI-I (VF0334)] [Pseudomonas aeruginosa PAO1] | 100.00 | 100.00 | NaN | 1806 | 2486 | - | VFDB | 016 | paeruginosa | 395 | 13 | Pseudomonas aeruginosa |
| icmF1/tssM1 | NP\_248767 | (icmF1/tssM1) type VI secretion system protein IcmF1 [HSI-I (VF0334)] [Pseudomonas aeruginosa PAO1] | 99.64 | 100.00 | NaN | 2483 | 5788 | - | VFDB | 016 | paeruginosa | 395 | 13 | Pseudomonas aeruginosa |
| pilX | NP\_253243 | (pilX) type 4 fimbrial biogenesis protein PilX [Type IV pili (VF0082)] [Pseudomonas aeruginosa PAO1] | 100.00 | 100.00 | NaN | 60679 | 61266 | + | VFDB | 016 | paeruginosa | 395 | 17 | Pseudomonas aeruginosa |
| fliQ | NP\_250138 | (fliQ) flagellar biosynthetic protein FliQ [Flagella (VF0273)] [Pseudomonas aeruginosa PAO1] | 99.63 | 100.00 | NaN | 133519 | 133788 | + | VFDB | 016 | paeruginosa | 395 | 14 | Pseudomonas aeruginosa |
| fliR | NP\_250139 | (fliR) flagellar biosynthetic protein FliR [Flagella (VF0273)] [Pseudomonas aeruginosa PAO1] | 99.61 | 100.00 | NaN | 133788 | 134564 | + | VFDB | 016 | paeruginosa | 395 | 14 | Pseudomonas aeruginosa |
| flhB | NP\_250140 | (flhB) flagellar biosynthetic protein FlhB [Flagella (VF0273)] [Pseudomonas aeruginosa PAO1] | 99.74 | 100.00 | NaN | 134567 | 135703 | + | VFDB | 016 | paeruginosa | 395 | 14 | Pseudomonas aeruginosa |
| flhA | NP\_250143 | (flhA) flagellar biosynthesis protein FlhA [Flagella (VF0273)] [Pseudomonas aeruginosa PAO1] | 99.86 | 100.00 | NaN | 138550 | 140673 | + | VFDB | 016 | paeruginosa | 395 | 14 | Pseudomonas aeruginosa |
| flhF | NP\_250144 | (flhF) flagellar biosynthesis protein FlhF [Flagella (VF0273)] [Pseudomonas aeruginosa PAO1] | 99.61 | 100.00 | NaN | 140757 | 142046 | + | VFDB | 016 | paeruginosa | 395 | 14 | Pseudomonas aeruginosa |
| fleN | NP\_250145 | (fleN) flagellar synthesis regulator FleN [Flagella (VF0273)] [Pseudomonas aeruginosa PAO1] | 100.00 | 100.00 | NaN | 142185 | 143027 | + | VFDB | 016 | paeruginosa | 395 | 14 | Pseudomonas aeruginosa |
| fliP | NP\_250137 | (fliP) flagellar biosynthetic protein FliP [Flagella (VF0273)] [Pseudomonas aeruginosa PAO1] | 99.74 | 100.00 | NaN | 132707 | 133474 | + | VFDB | 016 | paeruginosa | 395 | 14 | Pseudomonas aeruginosa |
| fliA | NP\_250146 | (fliA) flagellar biosynthesis sigma factor FliA [Deoxyhexose linking sugar 209 Da capping structure (AI138)] [Pseudomonas aeruginosa PAO1] | 99.87 | 100.00 | NaN | 143024 | 143767 | + | VFDB | 016 | paeruginosa | 395 | 14 | Pseudomonas aeruginosa |
| motD | NP\_250152 | (motD) flagellar motor protein [Deoxyhexose linking sugar 209 Da capping structure (AI138)] [Pseudomonas aeruginosa PAO1] | 99.78 | 100.00 | NaN | 149515 | 150405 | + | VFDB | 016 | paeruginosa | 395 | 14 | Pseudomonas aeruginosa |
| pilS | NP\_253236 | (pilS) two-component sensor PilS [Type IV pili (VF0082)] [Pseudomonas aeruginosa PAO1] | 100.00 | 100.00 | NaN | 53973 | 55565 | + | VFDB | 016 | paeruginosa | 395 | 17 | Pseudomonas aeruginosa |
| pilR | NP\_253237 | (pilR) two-component response regulator PilR [Type IV pili (VF0082)] [Pseudomonas aeruginosa PAO1] | 100.00 | 100.00 | NaN | 55580 | 56917 | + | VFDB | 016 | paeruginosa | 395 | 17 | Pseudomonas aeruginosa |
| fimT | NP\_253239 | (fimT) type 4 fimbrial biogenesis protein FimT [Type IV pili (VF0082)] [Pseudomonas aeruginosa PAO1] | 100.00 | 100.00 | NaN | 58192 | 58701 | + | VFDB | 016 | paeruginosa | 395 | 17 | Pseudomonas aeruginosa |
| fimU | NP\_253240 | (fimU) type 4 fimbrial biogenesis protein FimU [Type IV pili (VF0082)] [Pseudomonas aeruginosa PAO1] | 100.00 | 100.00 | NaN | 58807 | 59313 | + | VFDB | 016 | paeruginosa | 395 | 17 | Pseudomonas aeruginosa |
| pilV | NP\_253241 | (pilV) type IV pilus biogenesis protein PilV [Type IV pili (VF0082)] [Pseudomonas aeruginosa PAO1] | 100.00 | 100.00 | NaN | 59304 | 59861 | + | VFDB | 016 | paeruginosa | 395 | 17 | Pseudomonas aeruginosa |
| motC | NP\_250151 | (motC) flagellar motor protein [Deoxyhexose linking sugar 209 Da capping structure (AI138)] [Pseudomonas aeruginosa PAO1] | 100.00 | 100.00 | NaN | 148762 | 149502 | + | VFDB | 016 | paeruginosa | 395 | 14 | Pseudomonas aeruginosa |
| pilW | NP\_253242 | (pilW) type IV fimbrial biogenesis protein PilW [Type IV pili (VF0082)] [Pseudomonas aeruginosa PAO1] | 100.00 | 100.00 | NaN | 59858 | 60682 | + | VFDB | 016 | paeruginosa | 395 | 17 | Pseudomonas aeruginosa |
| fliO | NP\_250136 | (fliO) flagellar protein FliO [Flagella (VF0273)] [Pseudomonas aeruginosa PAO1] | 100.00 | 100.00 | NaN | 132258 | 132710 | + | VFDB | 016 | paeruginosa | 395 | 14 | Pseudomonas aeruginosa |
| fliM | NP\_250134 | (fliM) flagellar motor switch protein FliM [Flagella (VF0273)] [Pseudomonas aeruginosa PAO1] | 99.90 | 100.00 | NaN | 130784 | 131755 | + | VFDB | 016 | paeruginosa | 395 | 14 | Pseudomonas aeruginosa |
| hsiB1/vipA | NP\_248773 | (hsiB1/vipA) type VI secretion system tubule-forming protein VipA [HSI-I (VF0334)] [Pseudomonas aeruginosa PAO1] | 99.81 | 100.00 | NaN | 12191 | 12709 | + | VFDB | 016 | paeruginosa | 395 | 13 | Pseudomonas aeruginosa |
| hsiC1/vipB | NP\_248774 | (hsiC1/vipB) type VI secretion system tubule-forming protein VipB [HSI-I (VF0334)] [Pseudomonas aeruginosa PAO1] | 99.80 | 100.00 | NaN | 12722 | 14218 | + | VFDB | 016 | paeruginosa | 395 | 13 | Pseudomonas aeruginosa |
| hcp1 | NP\_248775 | (hcp1) type VI secretion system substrate Hcp1 [HSI-I (VF0334)] [Pseudomonas aeruginosa PAO1] | 100.00 | 100.00 | NaN | 14294 | 14782 | + | VFDB | 016 | paeruginosa | 395 | 13 | Pseudomonas aeruginosa |
| hsiE1 | NP\_248776 | (hsiE1) type VI secretion system hcp secretion island protein HsiE1 interacting with HsiB1 to form a novel subcomplex of the T6SS [HSI-I (VF0334)] [Pseudomonas aeruginosa PAO1] | 99.53 | 100.00 | NaN | 14950 | 15795 | + | VFDB | 016 | paeruginosa | 395 | 13 | Pseudomonas aeruginosa |
| hsiF1 | NP\_248777 | (hsiF1) type VI secretion system hcp secretion island protein HsiF1 a gp25-like protein but not exhibit lysozyme activity [HSI-I (VF0334)] [Pseudomonas aeruginosa PAO1] | 99.41 | 100.00 | NaN | 15797 | 16306 | + | VFDB | 016 | paeruginosa | 395 | 13 | Pseudomonas aeruginosa |
| hsiG1 | NP\_248778 | (hsiG1) type VI secretion system hcp secretion island protein HsiG1 [HSI-I (VF0334)] [Pseudomonas aeruginosa PAO1] | 99.73 | 100.00 | NaN | 16303 | 18162 | + | VFDB | 016 | paeruginosa | 395 | 13 | Pseudomonas aeruginosa |
| fliN | NP\_250135 | (fliN) flagellar motor switch protein FliN [Flagella (VF0273)] [Pseudomonas aeruginosa PAO1] | 100.00 | 100.00 | NaN | 131783 | 132256 | + | VFDB | 016 | paeruginosa | 395 | 14 | Pseudomonas aeruginosa |
| hsiH1 | NP\_248779 | (hsiH1) type VI secretion system hcp secretion island protein HsiH1 [HSI-I (VF0334)] [Pseudomonas aeruginosa PAO1] | 99.90 | 100.00 | NaN | 18126 | 19172 | + | VFDB | 016 | paeruginosa | 395 | 13 | Pseudomonas aeruginosa |
| vgrG1a | NP\_248781 | (vgrG1a) type VI secretion system substrate VgrG1 [HSI-I (VF0334)] [Pseudomonas aeruginosa PAO1] | 99.84 | 100.00 | NaN | 21920 | 23851 | + | VFDB | 016 | paeruginosa | 395 | 13 | Pseudomonas aeruginosa |
| vgrG1b | NP\_248785 | (vgrG1b) type VI secretion system substrate VgrG1b [HSI-1 (Hcp-secretion island 1) (SS178)] [Pseudomonas aeruginosa PAO1] | 99.69 | 100.00 | NaN | 26243 | 28468 | + | VFDB | 016 | paeruginosa | 395 | 13 | Pseudomonas aeruginosa |
| aprA | NP\_249940 | (aprA) alkaline metalloproteinase precursor [Alkaline protease (VF0090)] [Pseudomonas aeruginosa PAO1] | 99.58 | 100.00 | NaN | 7884 | 9323 | + | VFDB | 016 | paeruginosa | 395 | 14 | Pseudomonas aeruginosa |
| lasI | NP\_250123 | (lasI) autoinducer synthesis protein LasI [Quorum sensing (VF0093)] [Pseudomonas aeruginosa PAO1] | 100.00 | 100.00 | NaN | 117486 | 118091 | + | VFDB | 016 | paeruginosa | 395 | 14 | Pseudomonas aeruginosa |
| fliK | NP\_250132 | (fliK) flagellar hook-length control protein FliK [Deoxyhexose linking sugar 209 Da capping structure (AI138)] [Pseudomonas aeruginosa PAO1] | 98.83 | 100.00 | NaN | 128728 | 130011 | + | VFDB | 016 | paeruginosa | 395 | 14 | Pseudomonas aeruginosa |
| fliL | NP\_250133 | (fliL) flagellar basal body protein FliL [Deoxyhexose linking sugar 209 Da capping structure (AI138)] [Pseudomonas aeruginosa PAO1] | 99.81 | 100.00 | NaN | 130255 | 130776 | + | VFDB | 016 | paeruginosa | 395 | 14 | Pseudomonas aeruginosa |
| clpV1 | NP\_248780 | (clpV1) type VI secretion system AAA+ family ATPase [HSI-I (VF0334)] [Pseudomonas aeruginosa PAO1] | 99.48 | 100.00 | NaN | 19165 | 21873 | + | VFDB | 016 | paeruginosa | 395 | 13 | Pseudomonas aeruginosa |
| lip1 | NP\_248770 | (lip1) lipoprotein [HSI-I (VF0334)] [Pseudomonas aeruginosa PAO1] | 99.78 | 100.00 | NaN | 8716 | 9180 | - | VFDB | 016 | paeruginosa | 395 | 13 | Pseudomonas aeruginosa |
| pilY1 | NP\_253244 | (pilY1) type 4 fimbrial biogenesis protein PilY1 [Type IV pili (VF0082)] [Pseudomonas aeruginosa PAO1] | 92.21 | 99.66 | NaN | 61278 | 64769 | + | VFDB | 016 | paeruginosa | 395 | 17 | Pseudomonas aeruginosa |
[truncated: 2,400,485 more chars]
